# Mechanochemical Aerobic Activation of Metallic Copper for the Synthesis of 1,4‐Allenynes

**DOI:** 10.1002/cssc.202500211

**Published:** 2025-05-07

**Authors:** Ana M. Constantin, Francesco Mele, Matteo Lanzi, Giovanni Maestri, Raimondo Maggi, Nicola Della Ca’, Luca Capaldo

**Affiliations:** ^1^ Department of ChemistryLife Sciences and Environmental Sustainability University of Parma Parco Area delle Scienze 17/A 43124 Parma Italy; ^2^ CIRCC (Interuniversity Consortium Chemical Reactivity and Catalysis) via Celso Ulpiani 27 70126 Bari Italy

**Keywords:** ball milling, copper allenylidenes, copper catalysis, mechanochemistry, propargylic esters

## Abstract

Mechanical activation of zero‐valent metals is emerging as a new paradigm in sustainable synthesis, opening up a broad swath of chemical space under more practical conditions than solution‐phase methods. Contrary to its neighboring elements in the periodic table, copper has been significantly overlooked in this domain, especially for C—C bond formation. Herein, a mechanochemical method for synthesizing >25 previously unreported conjugated allenynes—a valuable scaffold in organic synthesis is presented—through copper‐catalyzed homocoupling of propargylic esters. This approach utilizes ball milling to activate inexpensive zero‐valent copper under solvent‐minimized conditions, sourced from both powders and recycled waste materials, including wires and bolts, into catalytically active Cu^I^ species under aerobic conditions. The synthetic utility of the synthesized compounds is showcased through an unprecedented rhodium‐catalyzed cyclocarbonylation and a gold‐catalyzed cyclization reaction.

## Graphical abstract

In a copper of hours. A sustainable mechanochemical approach to synthesize conjugated allenynes via copper‐catalyzed homocoupling of propargylic esters is reported. Using ball milling, cheap Cu^0^ from powders and recycled materials to generate catalytically active Cu^I^ species under aerobic conditions is activated. This method minimizes solvents and avoids the use of sensitive Cu^I^ salts.

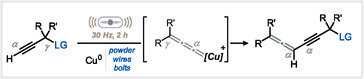

## 1 Introduction

Mechanochemistry is having a tremendous impact as a transformative and sustainable approach in the synthetic community.^[^ ^1^, ^2^, ^3^, ^4^, ^5^, ^6^, ^7^ ^]^ It utilizes mechanical forces to drive chemical reactions, thus complementing thermal, electrical, and photochemical activation modes, and offers advantages such as reduced waste, shorter reaction times, and alternative selectivity.

**Figure 1:**
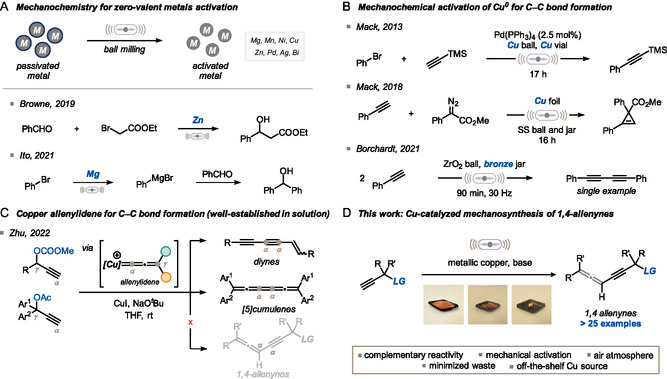
Context and state of the art. A) Mechanochemistry can be used for the activation of zero‐valent metals as an alternative to experimental tricks. B) Precedents of mechanochemical activation of copper for C—C bond formation. C) Precedent for the exploitation of the copper‐allenylidene species for the formation of C—C bond *in solution*. D) This work.

Recently, ball milling has been successfully applied for the activation of zero‐valent metals^[^ ^8^ ^]^ including Mg,^[^ ^9^, ^10^, ^11^, ^12^, ^13^ ^]^ Zn,^[^ ^14^, ^15^, ^16^, ^17^ ^]^ Mn,^[^ ^18^, ^19^ ^]^ Ni,^[^ ^20^ ^]^ Ag,^[^ ^21^, ^22^ ^]^ and Bi,^[^ ^23^ ^]^ and their use in C—C bond forming reactions has been extensively documented in mechanochemistry. In fact, the comminution of these elements can be exploited to remove the outer passivated layer (typically made of metal oxides) and expose more active metal surface (**Figure** **1**A). This generally allows for milder reaction conditions and eliminates the need for additives to trigger reactivity. Magnesium^[^ ^9^, ^10^, ^11^, ^12^, ^13^ ^]^ and zinc^[^ ^14^, ^15^, ^16^, ^17^, ^24^, ^25^ ^]^ undoubtedly stand out as the most investigated metals in this realm (Figure 1A).

Despite its low cost and abundance, copper has been largely underutilized in the development of mechanochemical methodologies for C—C bond formation, particularly when compared to its neighboring elements in the periodic table. In contrast, the formation of C—N bonds was more widely explored.^[^ ^26^, ^27^, ^28^, ^29^, ^30^, ^31^ ^]^ Notable examples of C—C bond formation include a mechanocatalytic Sonogashira coupling^[^ ^32^ ^]^ and a cyclopropenation of terminal alkynes^[^ ^21^ ^]^ reported by Mack and colleagues (Figure 1B). More recently, Borchardt and co‐workers developed a Cu^0^‐based mechanochemical Glaser coupling (one single example).^[^ ^33^ ^]^ In these reports, high‐speed ball milling in a copper jar equipped with a copper bead was exploited to generate the catalytic amount of metal needed for the C(sp)—H bond activation step (Figure 1B). Moreover, the same group has reported the development of direct mechanocatalysis, activating Pd alloys with Ni and Cu for Suzuki and Sonogashira couplings.^[^ ^34^ ^]^

Building upon our previous work on copper catalysis^[^ ^35^ ^]^ and set to expand the scope of copper‐catalyzed mechanochemical transformations for building molecular complexity, we were triggered by the promiscuous reactivity of copper with alkynes shown in solution.^[^ ^36^, ^37^ ^]^ Indeed, on one side, Cu can activate terminal acetylenes under basic conditions to form copper acetylides; on the other, it can facilitate the formation of copper allenylidenes when propargylic alcohol derivatives are employed. This peculiarity has been very recently exploited in solution^[^ ^38^, ^39^, ^40^ ^]^ relying on Cu^I^ complexes and inert conditions.

In one of these cases, Zhu and co‐workers reported the copper‐catalyzed synthesis of diynes and [5]cumulenes (Figure 1C) via a copper allenylidene intermediate by leveraging a base‐assisted proton transfer (BAPT) mechanism.^[^ ^40^ ^]^ Unfortunately, the occurrence of BAPT prevented the isolation of conjugated allenynes,^[^ ^41^, ^42^, ^43^, ^44^ ^]^ a highly sought‐after scaffold in natural products^[^ ^45^, ^46^, ^47^ ^]^ and molecular materials.^[^ ^48^ ^]^

Motivated by the underexplored potential of mechanochemical synthesis via zero‐valent copper, we decided to exploit this synthetic opportunity. Such an approach would unlock copper catalysis without protective atmospheres and offer a cost‐effective, readily available alternative to air‐sensitive Cu^I^ salts for this type of catalysis. However, it remained uncertain whether the transient copper species mentioned above (e.g., Cu allenylidene) could be harnessed under mechanochemical conditions.

Herein, we present the successful development of a copper‐catalyzed synthesis of 1,4‐allenynes under ball milling conditions (Figure 1D). Readily available forms of copper, including powder, wires, and bolts, were shown to effectively promote the desired reactivity. Our mechanistic investigation proved the critical role of mechanochemistry in enabling the controlled, on demand‐generation of the Cu^I^ species for efficient catalysis under aerobic conditions.

## 2 Results and Discussion

We started by optimizing the homocoupling of propargylic ester **1a** on a 0.2 mmol scale in the presence of cheap copper powder (5.1 mg, 40 mol%) and diisopropylethylamine (DIPEA, 1 eq.) as the base to promote deprotonation of the alkyne proton. The reaction mixture was milled in a stainless steel jar with one stainless steel ball (Ø = 10 mm) for 2 h at 30 Hz at room temperature, in the presence of CH_3_CN as a liquid‐assisted grinding (LAG) agent (*η* = 1), under air‐equilibrated conditions. Gratifyingly, under these conditions, the expected allenyne (**2a**) was formed in 65% ^1^H‐NMR yield with no major byproducts observed (**Table** **1**, entry 1). Increasing or decreasing the loading of copper did not improve this result (entries 2 and 3). Thus, we opted to double the milling time to 4 h; however, we found that **2a** undergoes decomposition upon prolonged milling (40%, entry 4; see also Section 7, Supporting Information). When attempting a gentler grinding (10 Hz), only 5% yield was obtained (entry 5). Replacing DIPEA with inorganic bases did not prove beneficial either (entries 6 and 7). More specifically, the use of K_2_CO_3_, used for a copper‐based Glaser coupling,^[^ ^38^ ^]^ did not produce the expected product. Similarly, when using KO*t*Bu as the base, the product was detected only in traces, with no consumption of the starting **1a** whatsoever.

**Table 1:** Optimization of reaction conditions.

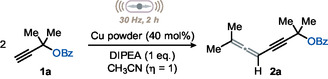		
Entry	Variation from conditionsa)	Yield of **2a** [%]b)
1	None	65
2	Cu (50 mol%)	62
3	Cu (20 mol%)	33
4	Milling time: 4 h	40
5	Milling frequency: 10 Hz	5
6	K_2_CO_3_ instead of DIPEA	n.d.
7	KO*t*Bu instead of DIPEA	<5
8	ZnO or NaCl as grinding agents	10
**9**	**DIPEA (2 eq.), CTAB (1 eq.)**	**84 (82)**
10	As entry 9, no LAG (neat)	59
11	As entry 9, no copper	n.d.
12	As entry 9, no DIPEA	n.d.
13	As entry 9, no milling	n.d.

a) General procedure: In a stainless steel jar (V = 5 mL) equipped with one stainless steel bead (Ø = 10 mm), alkyne (0.2 mmol), copper (40 mol%), additives, and LAG (*η* = 1) are sequentially added. The jar is sealed, mounted on a ball mill, and shaken as indicated. Bz: benzoyl.

b) ^1^H‐NMR yield (CH_2_Br_2_ as internal standard).

Next, with the aim of affecting the texture and heat capacity of the reaction mixture, we screened inert solid grinding agents like ZnO and NaCl (entry 8). Unfortunately, neither attempt led to improved yields. After extensive screening (see Section 4, Supporting Information), we found that using an excess of the base and a bromide source significantly improved reaction yield. In particular, among the bromide sources tested, cetyltrimethylammonium bromide (CTAB) proved the best, delivering **2a** in 84% yield (82% after isolation). Interestingly, the reaction delivers **2a** in satisfying yield, also in the absence of LAG (59%, entry 10).

Finally, control experiments were conducted to ascertain the crucial role of all the components: the removal of copper or DIPEA led to no conversion (entries 11 and 12), as did static conditions (i.e., no milling, entry 13). It is worth mentioning that no Glaser byproduct was observed under the investigated conditions.

**Figure 2:**
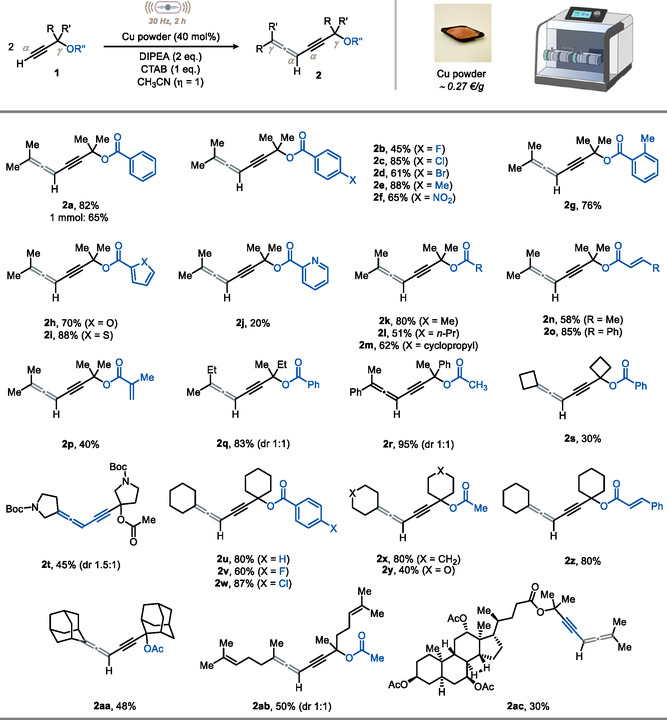
Scope of the copper‐catalyzed mechanochemical synthesis of 1,4‐allenynes. Reaction conditions: in a stainless steel jar (V = 5 mL) equipped with one stainless steel bead (Ø = 10 mm), alkyne (0.2 mmol), copper (40 mol%), DIPEA (2 eq.), CTAB (1 eq.), and CH_3_CN (*η* = 1) are sequentially added. The jar is sealed, mounted on a ball mill, and shaken as indicated.

With optimized conditions in hand, we turned to evaluate the generality of our approach (**Figure** **2**). First, we focused on the carboxylate group. Several substituents, including halogens, alkyl, and strongly electron‐withdrawing groups (e.g., NO_2_), were well tolerated and the expected products **2a–2f** were isolated in serviceable to excellent yields (45–88%). Gratifyingly, substituents on the ortho position of the aromatic ring did not hamper the reaction (**2g**, 76%). Other electron‐rich aromatics such as furan and thiophene (**2h** and **2i**) were also well tolerated; however, electron‐poor pyridine did not work well (**2j**, 20%) under optimized conditions. We attribute this result to the well‐known tendency of pyridine to coordinate the metal center and thus hamper the activation of **1**.

Next, we wondered if we could replace the aroyl group with an acyl one. Gratifyingly, we observed that the reaction remained efficient when replacing the phenyl ring with a methyl (**2k**), *n*‐propyl (**2l**), or cyclopropyl group (**2m**), which delivered the expected products in 80, 51, and 62%, respectively. Similarly, crotonates (**2n**), cinnamate (**2o**), and methacrylate (**2p**) allowed to obtain the corresponding allenynes to be obtained in modest‐to‐very good yields (Figure 2).

Next, we turned our attention to the carbon atom bearing the leaving group and assessed the impact of its substitution pattern on the overall transformation: we found that quaternary centers are uniquely suited for this method. Mixed alkyl substituents were well tolerated (**2q**, **2r**, 83% and 95%, respectively), while the use of 1‐ethynylcyclobutyl benzoate allowed to obtain the corresponding spirocyclic homocoupling product to be obtained only in modest yield (**2s**, 30%). *N*‐Boc pyrrolidine was also tolerated under the optimized conditions, and the corresponding spiroallenyne **2t** was obtained in 45% yield. Interestingly, less strained six‐membered rings on the quaternary carbon typically afforded the desired products **2u–2z** in higher yields. Finally, a very congested alkyne underwent homocoupling under our mechanochemical conditions (**2aa**) too, delivering the expected product in 48% yield. Notably, a citronellal and a cholic acid derivatives underwent the desired transformation in acceptable yields (**2b‐2ac**, >50%). It is important to note that alcohols and amines require protection to be compatible with our protocol (see **2t** and **2ac**). Additionally, alkynes bearing a less hindered tertiary carbon at the γ‐position represented a limitation, as they were fully recovered unreacted at the end of the reaction.

**Figure 3:**
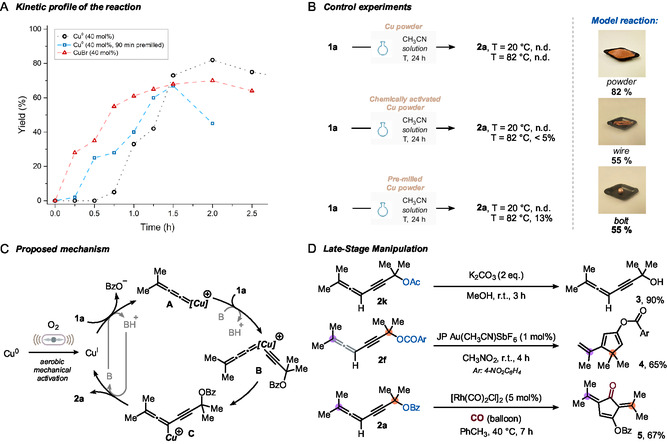
A) Kinetic profile of the reaction. B) Control experiments. C) Proposed mechanism. D) Late‐stage manipulation of **2a**. JP: JohnPhos.

Subsequently, we examined this mechanochemical transformation from a mechanistic perspective and started by recording the reaction profile over time in optimized conditions. Interestingly, the analysis revealed an induction period (**Figure** **3**A, black dotted line), with no **2a** observed until 45 min of milling time. To account for this observation, we formulated two hypotheses: (1) milling under aerobic conditions facilitates comminution of the metal, increasing its surface–to–volume ratio and promoting the formation of oxidized copper species that drive the observed reactivity; or (2) similarly to other metals, the milling process is needed to remove the copper basic oxide patina, thereby exposing fresh zero‐valent copper, which serves as the active species in the reaction.

To test our first hypothesis, we surmised that a copper bromide species could be formed under our conditions and be the active catalytic species. Specifically, Cu^I^ species may be stabilized by the additives (amine and bromide) as CuBr. Accordingly, we replaced Cu^0^ powder with CuBr (40 mol%) and monitored the reaction profile under otherwise standard conditions (Figure 3A, red dotted line). The induction period disappeared. We also tracked the reaction profile using premilled copper (90 min at 30 Hz) and found that the induction period was significantly reduced compared to standard conditions (Figure 3A, see blue and black dotted lines). To put our second hypothesis to the test, we started by adding off‐the‐shelf copper powder to a stirring CH_3_CN solution of propargyl ester **1a**, DIPEA (2 eq.), and CTAB (1 eq.) at room temperature: unsurprisingly, no product was formed even after 24 h (Figure 3B). A similar outcome was observed when we used chemically activated copper (via HNO_3_ etching) or mechanically activated copper (ball milling, 90 min at 30 Hz). In both cases, allenyne **2a** was not formed, and the starting material **1a** was largely recovered (≈90%).

Furthermore, in any of the cases mentioned above, increasing the reaction temperature had a positive impact (Figure 3B). Based on these findings, we excluded a pivotal role for zero‐valent copper in this reaction. It is important to mention here that we excluded Cu_2_O, CuO, and CuBr_2_ as putative catalysts in our reaction conditions (see Section 4, Supporting Information).

Moreover, when the model reaction was performed under an inert atmosphere (Ar), we obtained little amount of the product (15% via ^1^H‐NMR), which identifies molecular oxygen as the terminal oxidant to form the active Cu^I^ species (see Section 7, Supporting Information).^[^ ^33^ ^]^ Overall, we propose that the initial milling time of copper is needed for the in situ generation of Cu^I^, which effectively starts catalysis.

To evaluate the robustness of our reaction, we tested alternative copper sources, including electrical wires and bolts purchased at a local electrical supply store. We found that, although copper powder delivers the best yield in the series, other forms can be utilized to convert **1a** into **2a** in serviceable yields. However, in these cases, a significant abrasion of the metal was observed.^[^ ^33^ ^]^

Considering the above findings and drawing from solution‐phase precedents,^[^ ^37^, ^40^ ^]^ we propose the mechanistic pathway illustrated in Figure 3C. Specifically, mechanical activation of zero‐valent copper under aerobic conditions generates the catalytically active Cu^I^ species, which is stabilized by the base and additive. In the presence of DIPEA as the base, an alkynyl copper intermediate is formed, and subsequent expulsion of the carboxylate group yields copper allenylidene intermediate (**A**). This is followed by the involvement of an additional equivalent of **1a**, leading to the formation of the mixed copper species (**B**) and, upon migratory insertion, of **C**. Finally, protodecupration of **C** leads to product **2a**. Notably, unlike the solution‐phase mechanism reported by Zhu and coworkers, we did not observe the formation of diynes or cumulenes.^[^ ^40^, ^49^ ^]^

Finally, to prove the versatility of the 1,4‐allenynes synthesized in this work, we explored diverse conditions for the late‐stage manipulation of **2a** (Figure 3D). First, **2a** could be easily deprotected under basic conditions, yielding propargylic alcohol **3** in 90% yield, with no need for column chromatography. The allenyne core remained untouched. Second, intrigued by the well‐known reactivity of allenes with transition metals,^[^ ^50^ ^]^ we attempted a gold‐catalyzed cascade cyclization.^[^ ^51^, ^52^ ^]^ When using JohnPhos as the ligand, cyclopentadiene **4** was isolated in 65% yield after stirring in CH_3_NO_2_ for 4 h at room temperature. Finally, a low‐pressure Rh‐catalyzed cyclocarbonylation was carried out to deliver cyclopentenone **5** in 67% yield after 7 h of stirring at 40 °C in toluene.^[^ ^53^ ^]^ [Correction added on 14 May 2025, after first online publication: Reference 49 has been added in Section 2.]

## 3 Conclusion

In conclusion, we have reported the first mechanochemical approach for the synthesis of conjugated allenynes. We successfully exploited the reactivity of copper allenylidene under solvent‐minimized conditions by activating zero‐valent, inexpensive copper metal from diverse sources, including low‐cost feedstock items. While there is still room to enhance the catalytic efficiency, we demonstrated that ball milling is essential for generating catalytically active Cu^I^ species under aerobic conditions, thereby eliminating the need for air‐ and moisture‐sensitive Cu^I^ salts. Finally, the synthetic utility of the allenynes synthesized herein was demonstrated through their catalytic transformation into cyclopentenones via a novel rhodium‐catalyzed carbonylation and into cyclopentenes through a gold‐catalyzed rearrangement.

## Conflict of Interest

The authors declare no conflict of interest.

## Author Contributions

**Ana M. Constantin**: data curation (lead); investigation (equal); methodology (equal); writing—review editing (supporting). **Francesco Mele**: data curation (supporting); investigation (equal); methodology (equal; validation (lead); writing—review editing (supporting). **Matteo Lanzi**: writing—review editing (supporting); **giovanni maestri**: writing—review editing (supporting); **Raimondo Maggi**: resources (equal); writing—review editing (supporting). **Nicola Della Ca’**: funding acquisition (equal); resources (equal); supervision (equal); writing—review editing (supporting). **Luca Capaldo**: conceptualization (lead); funding acquisition (supporting); project administration (lead); supervision (lead); writing—original draft (lead); writing—review editing (lead). **Ana M. Constantin** and **Francesco Mele** contributed equally to this work.

## Supporting information

Supplementary Material

## Data Availability

The data that support the findings of this study are available from the corresponding author upon reasonable request.

## References

[1] J. L. Howard , Q. Cao , D. L. Browne , Chem. Sci. 2018, 9, 3080. 29780455 10.1039/c7sc05371a PMC5933221

[2] S. L. James , C. J. Adams , C. Bolm , D. Braga , P. Collier , T. Friščić , F. Grepioni , K. D. M. Harris , G. Hyett , W. Jones , A. Krebs , J. Mack , L. Maini , A. G. Orpen , I. P. Parkin , W. C. Shearouse , J. W. Steed , D. C. Waddell , Chem. Soc. Rev. 2012, 41, 413. 21892512 10.1039/c1cs15171a

[3] F. Cuccu , L. De Luca , F. Delogu , E. Colacino , N. Solin , R. Mocci , A. Porcheddu , ChemSusChem 2022, 15, e202200362. 35867602 10.1002/cssc.202200362 PMC9542358

[4] J.‐L. Do , T. Friščić , ACS Cent. Sci. 2017, 3, 13. 28149948 10.1021/acscentsci.6b00277 PMC5269651

[5] V. Martinez , T. Stolar , B. Karadeniz , I. Brekalo , K. Užarević , Nat. Rev. Chem. 2022, 7, 51. 37117822 10.1038/s41570-022-00442-1

[6] G.‐W. Wang , Chem. Soc. Rev. 2013, 42, 7668. 23660585 10.1039/c3cs35526h

[7] F. Mele , A. M. Constantin , A. Porcheddu , R. Maggi G. Maestri , N. D. Ca’ , L. Capaldo , Beilstein J. Org. Chem. 2025, 21, 458. 40041196 10.3762/bjoc.21.33 PMC11878148

[8] A. C. Jones , J. A. Leitch , S. E. Raby‐Buck , D. L. Browne , Nat. Synth. 2022, 1, 763F775.

[9] V. S. Pfennig , R. C. Villella , J. Nikodemus , C. Bolm , Angew. Chem. Int. Ed. 2022, 61, e202116514. 10.1002/anie.202116514 PMC9306648 34942056

[10] J. M. Harrowfield , R. J. Hart , C. R. Whitaker , Aust. J. Chem. 2001, 54, 423.

[11] I. R. Speight , T. P. Hanusa , Molecules 2020, 25, 570. 32012963 10.3390/molecules25030570 PMC7037680

[12] R. Takahashi , A. Hu , P. Gao , Y. Gao , Y. Pang , T. Seo , J. Jiang , S. Maeda , H. Takaya , K. Kubota , H. Ito , Nat. Commun. 2021, 12, 6691. 34795265 10.1038/s41467-021-26962-w PMC8602241

[13] C. Wu , T. Ying , X. Yang , W. Su , A. V. Dushkin , J. Yu , Org. Lett. 2021, 23, 6423. 34351160 10.1021/acs.orglett.1c02241

[14] G.‐W. Wang , Y. Murata , K. Komatsu , T. S. M. Wan , Chem. Commun. 1996, 2059.

[15] Q. Cao , R. T. Stark , I. A. Fallis , D. L. Browne , ChemSusChem 2019, 12, 2554. 31033237 10.1002/cssc.201900886 PMC6619031

[16] J. Yin , R. T. Stark , I. A. Fallis , D. L. Browne , J. Org. Chem. 2020, 85, 2347. 31913623 10.1021/acs.joc.9b02876

[17] Q. Cao , J. L. Howard , E. Wheatley , D. L. Browne , Angew. Chem. Int. Ed. 2018, 57, 11339. 10.1002/anie.201806480 PMC6220771 30015403

[18] S. Wu , W. Shi , G. Zou , New J. Chem. 2021, 45, 11269.

[19] W. I. Nicholson , J. L. Howard , G. Magri , A. C. Seastram , A. Khan , R. R. A. Bolt , L. C. Morrill , E. Richards , D. L. Browne , Angew. Chem. Int. Ed. 2021, 60, 23128. 10.1002/anie.202108752 PMC8596600 34405513

[20] R. A. Haley , A. R. Zellner , J. A. Krause , H. Guan , J. Mack , ACS Sustainable Chem. Eng. 2016, 4, 2464.

[21] L. Chen , D. Leslie , M. G. Coleman , J. Mack , Chem. Sci. 2018, 9, 4650. 29899959 10.1039/c8sc00443a PMC5969500

[22] L. Chen , M. O. Bovee , B. E. Lemma , K. S. Keithley , S. L. Pilson , M. G. Coleman , J. Mack , Angew. Chem. Int. Ed. 2015, 54, 11084. 10.1002/anie.201504236 26352021

[23] S. Wada , N. Hayashi , H. Suzuki , Org. Biomol. Chem. 2003, 1, 2160. 12945908 10.1039/b303783e

[24] L. Pontini , J. A. Leitch , D. L. Browne , Green Chem. 2023, 25, 4319.

[25] A. C. Jones , W. I. Nicholson , J. A. Leitch , D. L. Browne , Org. Lett. 2021, 23, 6337. 34342468 10.1021/acs.orglett.1c02096

[26] L. Rinaldi , K. Martina , F. Baricco , L. Rotolo , G. Cravotto , Molecules 2015, 20, 2837. 25671367 10.3390/molecules20022837 PMC6272186

[27] T. L. Cook , J. A. Walker , J. Mack , Green Chem. 2013, 15, 617.

[28] M. Tireli , S. Maracic , S. Lukin , M. J. Kulcsar , D. Zilic , M. Cetina , I. Halasz , S. Raic‐Malic , K. Uzarevic , Beilstein J. Org. Chem. 2017, 13, 2352. 29181115 10.3762/bjoc.13.232 PMC5687011

[29] D. Tan , V. Strukil , C. Mottillo , T. Friscic , Chem. Commun. 2014, 50, 5248. 10.1039/c3cc47905f 24256886

[30] P. Wu , L. Ling , Y. Hu , S. Pan , C. Bolm , ACS Sustainable Chem. Eng. 2024, 12, 15875.

[31] W. Shi , Y. Shi , M. Xü , G. Zou , X.‐Y. Wu , Molecular Catalysis 2022, 528, 112472.

[32] D. A. Fulmer , W. C. Shearouse , S. T. Medonza , J. Mack , Green Chem. 2009, 11, 1821.

[33] C. G. Vogt , M. Oltermann , W. Pickhardt , S. Grätz , L. Borchardt , Adv. Energy Sustainability Res. 2021, 2, 2100011.

[34] W. Pickhardt , F. J. L. Kraus , M. Wohlgemuth , M. Etter , M. Spallek , M. Schwensow , Z. Genç , S. Grätz , L. Borchardt , ChemCatChem 2024, 16, e202400491.

[35] F. Mele , A. M. Constantin , F. Sacchelli , D. Schiroli , P. P. Mazzeo , G. Maestri , E. Motti , R. Maggi , R. Mancuso , B. Gabriele , F. Pancrazzi , N. Della Ca’ , Green Chem. 2024, 26, 6429.

[36] A. Delgado , P. Orlando , M. Lanzi , J. Benet‐Buchholz , D. Passarella , A. W. Kleij , Org. Lett. 2024, 26, 7596. 39213514 10.1021/acs.orglett.4c02682 PMC11406568

[37] G. Hattori , K. Sakata , H. Matsuzawa , Y. Tanabe , Y. Miyake , Y. Nishibayashi , J. Am. Chem. Soc. 2010, 132, 10592. 20617844 10.1021/ja1047494

[38] K. Zhong , C. Shan , L. Zhu , S. Liu , T. Zhang , F. Liu , B. Shen , Y. Lan , R. Bai , J. Am. Chem. Soc. 2019, 141, 5772. 30887803 10.1021/jacs.8b13055

[39] Z. Y. Wang , R. Zhu , J. Am. Chem. Soc. 2023, 145, 23755. 37853723 10.1021/jacs.3c08290

[40] Q. Yao , B. Liu , T. Cao , S. Zhu , Chem. Commun. 2022, 58, 4969. 10.1039/d2cc00681b 35353104

[41] J. Wang , H. Qian , S. Ma , Org. Chem. Front. 2024, 11, 437.

[42] G. Wu , Y. Yao , G. Li , X. Zhang , H. Qian , S. Ma , Angew. Chem. Int. Ed. 2022, 61, e202112427. 10.1002/anie.202112427 34734475

[43] X. Duan , Q. Jin , K. Wang , Y. Sun , Y. Wei , Z. Wang , J.‐K. Qiu , K. Guo , X. Bao , X. Wu , ACS Catal. 2024, 14, 13219.

[44] S. Yu , S. Ma , Angew. Chem. Int. Ed. 2012, 51, 3074. 10.1002/anie.201101460 22271630

[45] A. Hoffmann‐Röder , N. Krause , Angew. Chem. Int. Ed. 2004, 43, 1196. 10.1002/anie.200300628 14991780

[46] J. G. Ondeyka , D. L. Zink , K. Young , R. Painter , S. Kodali , A. Galgoci , J. Collado , J. R. Tormo , A. Basilio , F. Vicente , J. Wang , S. B. Singh , J. Nat. Prod. 2006, 69, 377. 16562839 10.1021/np050416w

[47] R. E. Bew , J. R. Chapman , E. R. H. Jones , B. E. Lowe , G. Lowe , J. Chem. Soc. C 1966, 129.

[48] P. Rivera‐Fuentes , F. Diederich , Angew. Chem. Int. Ed. 2012, 51, 2818. 10.1002/anie.201108001 22308109

[49] K. J. Ardila‐Fierro , C. Bolm , J. G. Hernández , Angew. Chem. Int. Ed. 2019, 58, 12945. 10.1002/anie.201905670 PMC6773223 31265746

[50] A. S. K. Hashmi , Angew. Chem. Int. Ed. 2000, 39, 3590. 11091408

[51] G. Lemière , V. Gandon , N. Agenet , J.‐P. Goddard , A. de Kozak , C. Aubert , L. Fensterbank , M. Malacria , Angew. Chem. Int. Ed. 2006, 45, 7596. 10.1002/anie.200602189 17051571

[52] W. Yang , A. S. K. Hashmi , Chem. Soc. Rev. 2014, 43, 2941. 24671183 10.1039/c3cs60441a

[53] Z. Y. Tian , Q. Cui , C. H. Liu , Z. X. Yu , Angew. Chem. Int. Ed. 2018, 57, 15544. 10.1002/anie.201805908 30102822

