# Supplementary material for: Mechanochemical Aerobic Activation of Metallic Copper for the Synthesis of 1,4‐Allenynes
Source: ChemSusChem. 2025 May 7;18(13):e202500211. doi: 10.1002/cssc.202500211 (PMC12231817; doi:10.1002/cssc.202500211)
Supplement: Supplementary file 1 — Supplementary Material [file CSSC-18-e202500211-s001.pdf]

Supporting Information

Mechanochemical Aerobic Activation of Metallic Copper for the Synthesis of 1,4-Allenynes

Ana M. Constantin,[a],‡ Francesco Mele,[a],‡, Matteo Lanzi,[b],[c] Giovanni Maestri,[b],[c] Raimondo Maggi,[a],[c] Nicola Della Ca',[a],[c],* Luca Capaldo[a],[c],*

[a] SynCat Lab, Department of Chemistry, Life Sciences and Environmental Sustainability, University of Parma, Parco Area delle Scienze 17/A, 43124 Parma, Italy. E-mail: luca.capaldo@unipr.it; nicola.dellaca@unipr.it

[b] Lab052, Department of Chemistry, Life Sciences and Environmental Sustainability, University of Parma, Parco Area delle Scienze 17/A, 43124 Parma, Italy.

[c] CIRCC (Interuniversity Consortium Chemical Reactivity and Catalysis), via Celso Ulpiani 27, 70126 Bari (Italy).

‡ These authors contributed equally to this work.

Contents

1. GENERAL INFORMATION S3
2. MECHANOCHEMISTRY EQUIPMENT S4
3. CHART OF STARTING MATERIALS S5
4. OPTIMIZATION FOR THE MECHANOSYNTHESIS OF 1,4-ALLENYNES S6
General procedure for the optimization of reaction parameters S6
Optimization of the catalyst loading S6
Optimization of the base S7
Optimization of milling frequency S7
Optimization of additives and texture agents S8
Control experiments S8
Control experiments using different oxidants S9
Optimization of the LAG S9
5. GENERAL PREPARATIVE PROCEDURES S10
5.1 General procedures for the synthesis of starting materials S10
General Procedure 1 (GP1) S10
General Procedure 2 (GP2) S10
General Procedure 3 (GP3) S10
5.2 General procedure for the mechanochemical synthesis of 1,4 allenynes S11
General Procedure 4 (GP4) S11
5.3 Late-stage manipulation of 1,4-allenynes S11
Deprotection of **2k** S11
Gold-mediated cyclization of **2f** S11
Rhodium-catalyzed carbonylative cyclization of **2a** S12
6. CHARACTERIZATION OF COMPOUNDS S13
Substrates 1a-ac S13
Products 2a-ac S22
Products 3-5 .... S31
Elemental analysis of unknown compounds S33
7. MECHANISTIC INVESTIGATION S34
Use of zero-valent copper in solution S34
Studies on the mechanochemical degradation of 2a S35
Influence of O_2 on the mechanochemical process S35
Influence of CuBr percentages on the mechanochemical process S36
Kinetic experiments S37
Kinetic profile under optimized conditions S37
Kinetic profile with CuBr (40 mol%) S37
Kinetic profile with pre-milled copper S38
8. REFERENCES S39
9. COPIES OF NMR SPECTRA S40
Substrates 1a-ac S41
Products 2a-ac S72

1. General Information

Reagents and consumables. All reagents and solvents were bought from Sigma Aldrich, TCI, Fluorochem, and BLDPharm and used as received unless otherwise specified. The solvents employed in this work were purchased from Carlo Erba or Sigma Aldrich and used as received. Disposable syringes were purchased from B. Braun. TLC analysis was performed using Silica on aluminum foils TLC plates (F254, Merck) with visualization under ultraviolet light (254 nm and 365 nm) or appropriate TLC staining (potassium permanganate).

NMR spectroscopy. ^{1}H and ^{13}C NMR spectra were recorded on a Bruker AVANCE 400 or a JEOL 600MHz ECZ600R spectrometers in deuterated chloroform, using the solvent signals as internal reference (7.26 and 77.16 ppm for ^{1}H and ^{13}C, respectively). For ^{19}F JEOL 600MHz ECZ600R spectrometer was used. The terms m, s, d, t, q and quint represent multiplet, singlet, doublet, triplet, quadruplet and quintuplet, respectively. NMR data were processed using the MestReNova 14 software package. The identity of known compounds was confirmed by comparing the corresponding ^{1}H NMR and ^{13}C NMR with those reported in the literature retrieved via the Reaxys Database.

Ball milling apparatus. Mechanochemical experiments described in this work were conducted in a Retsch MM400 or in a Fritsch pulverisette P23 mixer mill. Stainless steel with stainless-steel bearings were used, unless otherwise indicated.

Melting point. Melting points were measured with an Electrothermal apparatus and are uncorrected.

Elemental Analysis. A FlashSmart CHNS instrument from ThermoFisher Scientific equipped with a Thermal Conductivity Detector was used.

2. Mechanochemistry Equipment

Most mechanochemical experiments described in this work were conducted in a Retsch MM400 mixer mill (Figure S1 left), using a stainless steel milling jar (V = 5 mL) and one 10-mm hardened steel milling ball (hardened chrome steel with <12% chromium content; RETSCH 05.368.0032) at a frequency of 30 Hz under ambient conditions without external heating.

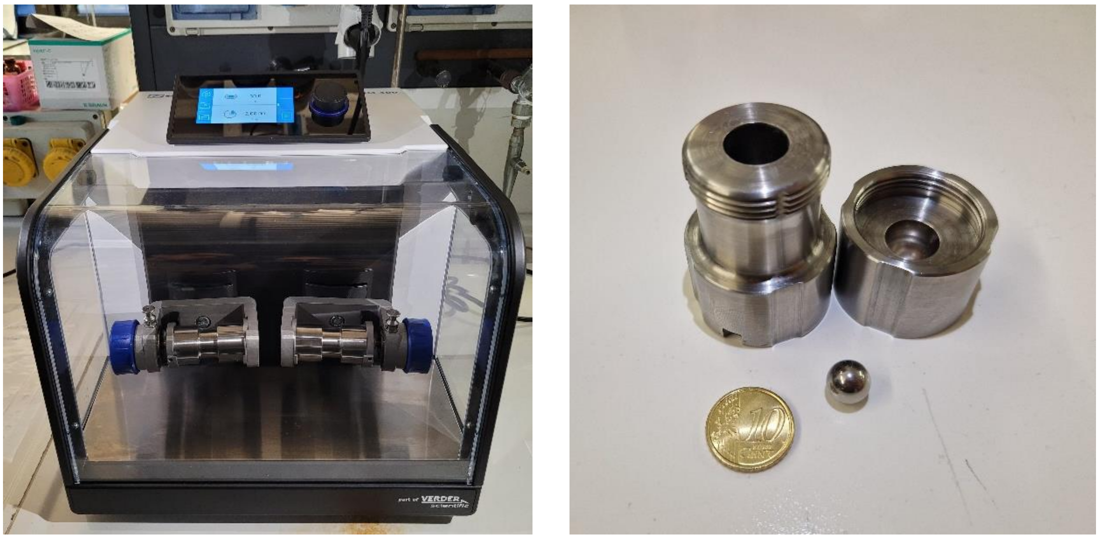

Figure S1. MM400 mixer mill used in this work for explorative and preparative experiments (left). Close-up of the stainless-steel jar and bead used; a 0.1 € coin for comparison.

In some instances (see Section 7), a Fritsch pulverisette P23 vertical mill was used (Figure S2). In this case, a stainless-steel jar with one 15-mm stainless-steel bead was used.

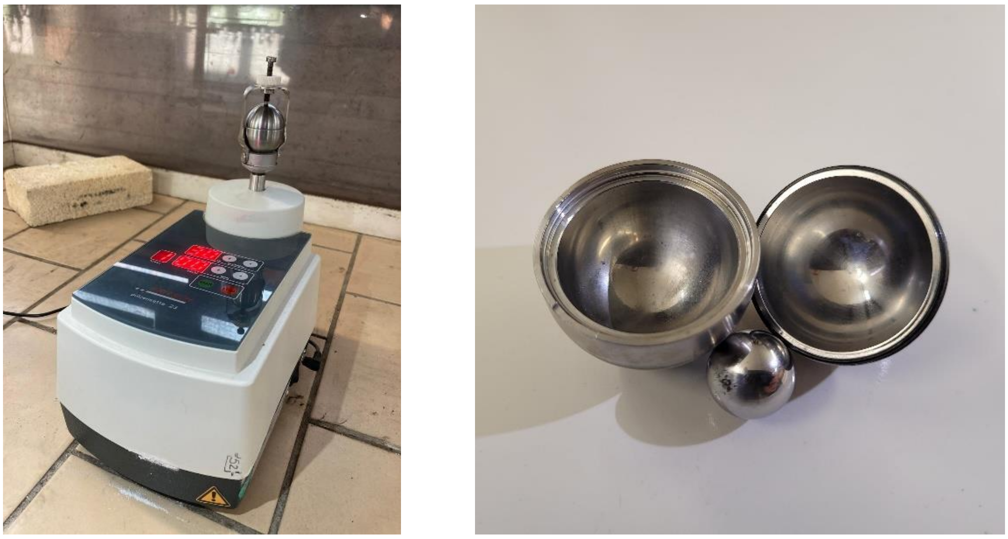

Figure S2. Fritsch pulverisette P23 vertical mixer mill used for mechanistic experiments (left). MM400 mixer mill used in this work for explorative and preparative experiments (left). Close-up of the stainless-steel jar and bead used.

3. Chart of starting materials

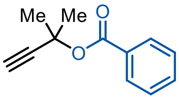

1a, 80%

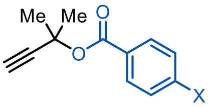

1b, 75% (X = F)
1c, 75% (X = Cl)
1d, 65% (X = Br)
1e, 75% (X = CH_3)
1f, 85% (X = NO_2)

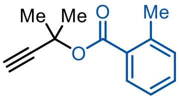

1g, 65%

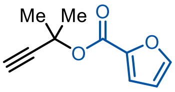

1h, 10%

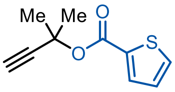

1i, 29%

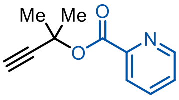

1j, 30%

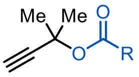

1k, 90% (X = CH_3)
1l, 61% (X = *n*-Pr)

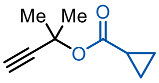

1m, 65%

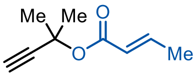

1n, 58%

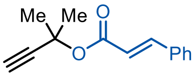

1o, 80%

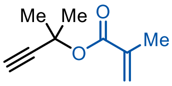

1p, 13%

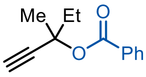

1q, 35%

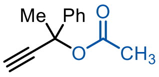

1r, 98%

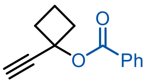

1s, 70%

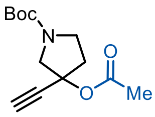

1t, 60%

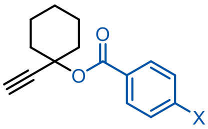

1u, 30% (X = H)
1v, 18% (X = F)
1w, 26% (X = Cl)

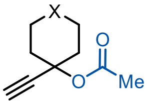

1x, 88% (X = CH_2)
1y, 65% (X = O)

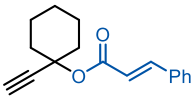

1z, 40%

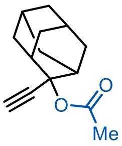

1aa, 85%

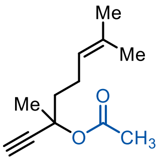

1ab, 55%

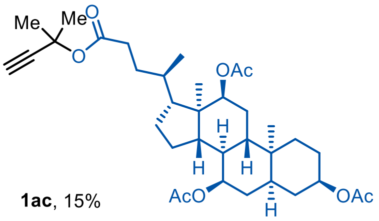

1ac, 15%

Figure S3. Propargyl esters synthesized in this work.

4. Optimization for the mechanosynthesis of 1,4-allenynes

General procedure for the optimization of reaction parameters

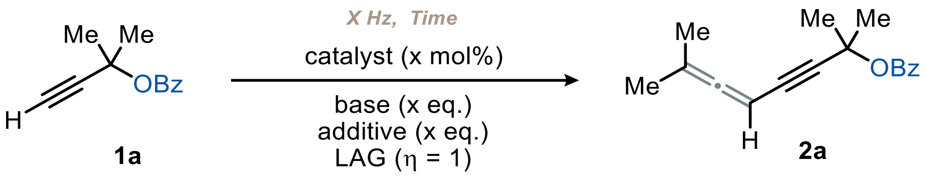

2-Methyl-but-3-yn-2-yl benzoate (**1a**) (37.6 mg, 0.2 mmol, 1 eq.), copper catalyst, additive, base and LAG were placed in a 5 mL stainless-steel milling jar, equipped with one 10 mm hardened stainless-steel ball. The vessel was sealed and mounted on a MM400 mixer mill. The desired frequency was set on the instrument, and milling was carried out for the specified time. After milling, the reaction crude was recovered with EtOAc and filtered over a short plug of silica gel. The solvent was removed, and the yield of **2a** was determined via ^1H NMR ($CDCl_3$) using CH_2Br_2 as internal standard.

NB: After each use, stainless steel jars were rigorously cleaned with a 30% ammonium hydroxide solution to ensure complete removal of copper traces.

Optimization of the catalyst loading

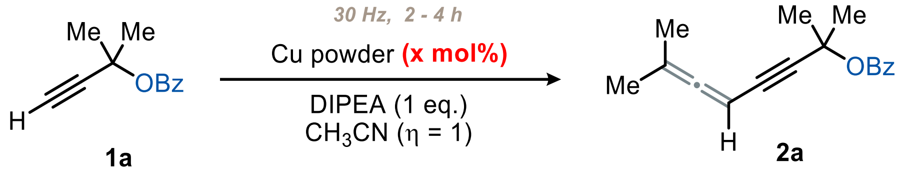

Table S1. Optimization of the catalyst loading.

Entry	*Cu (x mol%)*	*Milling time*	*Yield (%)*[a]
1	100 mol%	2 h	62 (60)[b]
2	50 mol%	2 h	62
3	40 mol%	2 h	65
4	20 mol%	2 h	33
5	10 mol%	2 h	21
6	40 mol%	4 h	40

[a] *Yields were calculated using CH_2Br_2 as internal standard.* [b] *Yield after isolation.*

Optimization of the base

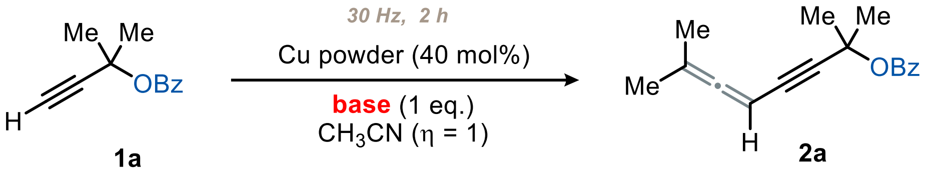

Table S2. Optimization of the base.

Entry	*Base*	*Yield (%)*[a]
1	K_2CO_3	n.d.
2	KO*t*Bu	< 5
3	TBD	20
4	DMAP	n.d.
5	DABCO	10
6	TEA	49
7	DIPEA	65

[a] *Yields were calculated using CH_2Br_2 as internal standard. TBD: 1,5,7-triazabicyclo[4.4.0]dec-5-ene. DMAP: N,N-dimethylaminopyridine. DABCO: 1,4-diazabicyclo[2.2.2]octane. TEA: triethylamine. DIPEA: diisopropylethylamine.*

Optimization of milling frequency

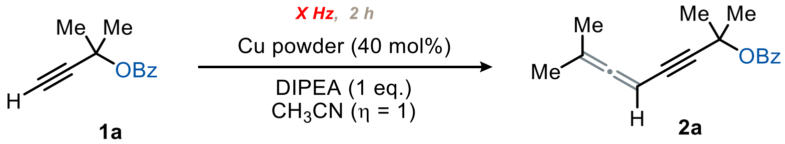

Table S3. Optimization of milling frequency.

Entry	*Frequency (Hz)*	*Yield (%)*[a]
1	0	-
2	10	5
3	20	10
4	25	20
5	30	65
6[b]	30	30

[a] *Yields were calculated using CH_2Br_2 as internal standard.* [b] *Two 10-mm stainless-steel balls were used.*

Optimization of additives and texture agents

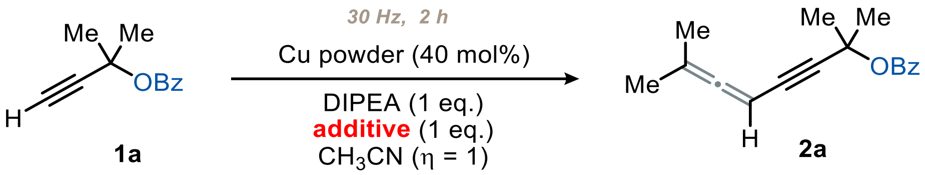

Table S4. Screening of additives and texture agents.

Entry	*Additive*	*Yield (%)*[a]
1	ZnO	10
2	NaCl	10
3	NH_4Cl	60
4	9-octadecenylamine	48[b]
5	TBAB	35
6	NH_4Br	60
7	CTAB	32
8	TBAI	46
9	NH_4I	35

[a] Yields were calculated using CH_2Br_2 as internal standard. [b]Product was isolated in 46% yield after column chromatography. CTAB: cetylammonium bromide. TBAB: tetrabutylammonium bromide. TBAI: tetrabutylammonium iodide.

Control experiments

Table S5. Control experiments.

Entry	*Cat (40 mol%)*	*DIPEA (2 eq.)*	*CTAB*	*CH_3CN (η=1)*	*Yield (%)*[a]
1	-	2 eq.	1 eq.	yes	n.d.
2	Cu powder	-	1 eq.	yes	n.d.
3	Cu powder	2 eq.	-	no	20
4[b]	Cu powder	2 eq.	1 eq.	yes	n.d.
5[c]	Cu powder	2 eq.	1 eq.	yes	41
6	Cu_2O	2 eq.	1 eq.	yes	7
7	$CuBr_2$	2 eq.	1 eq.	yes	18
8[d]	Cu powder	2 eq.	1 eq.	yes	40
9	Cu powder	1 eq.	2 eq.	yes	55
10	Cu powder	2 eq.	2 eq.	yes	65
11	**Cu powder**	**2 eq.**	**1 eq.**	**yes**	**84 (82)**

[a] Yields were calculated using CH_2Br_2 as internal standard. [b] The reaction mixture was left in the jar without milling for 2 h. [c] The copper powder was pre-milled for 90 minutes at 30 Hz and then used. [d] The reaction was conducted in a zirconia jar (V = 15 mL) equipped with one ZrO_2 ball using general procedure.

Control experiments using different oxidants

Table S6. Screening of oxidants.

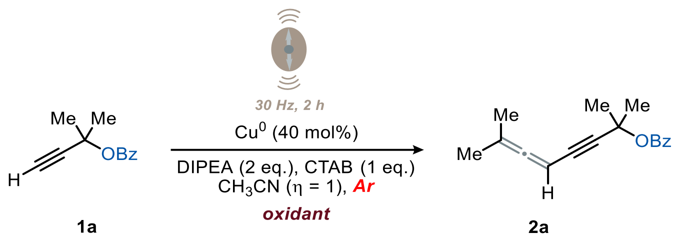

Entry	Oxidant	Yield (%)[a]
1	**air**	50%
2	H_2O_2 (1 eq)	63%[b]
3	BQ (1 eq)	n.d.

*Reactions performed with a Fritsch P23 vertical ball mill, in a stainless-steel jar of 15 mL and one 10 mm milling ball [a] Yields were calculated using CH_2Br_2 as internal standard. [b] 19% of **1a** was detected.*

Optimization of the LAG

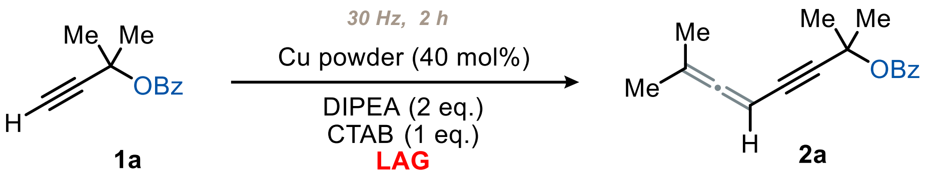

Table S7. Screening of different LAGs.

Entry	LAG (η = 1)	Yield (%)[a]
1	H_2O	7
2	2-Me-THF	64
3	Toluene	75
4	DMF	n.d.
5	CH_3NO_2	n.d.
6	HFIP	n.d.
7	CH_3CN	84
8	Neat (η = 0)	59

[a] Yields were calculated using CH_2Br_2 as internal standard.

5. General preparative procedures

5.1 General procedures for the synthesis of starting materials

General Procedure 1 (GP1)

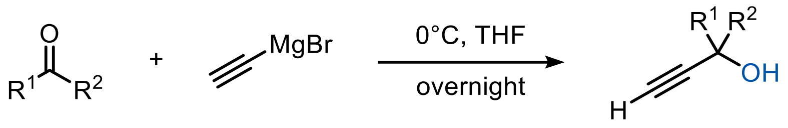

According to the literature procedure,[1] ethynylmagnesium bromide (0.50 M solution in THF, 3.6 mmol, 1.2 eq.) was added dropwise to a stirring solution of ketone (3 mmol, 1 eq.) in 3 mL anhydrous THF at 0 °C under N_2. The resulting solution was stirred at 0 °C for 10 min, then warmed up to room temperature and stirred until completion, as judged by TLC. The reaction mixture was carefully quenched with aqueous NH_4Cl solution (10 mL), extracted with EtOAc (10 m × 2) and subsequently dried over anhydrous Na_2SO_4. Finally, the solvent was removed under reduced pressure. The crude product was used without any further purification for the next steps. This procedure was used for the synthesis of alcohols precursors of **1s**, **1t, 1y** and **1aa**.

General Procedure 2 (GP2)

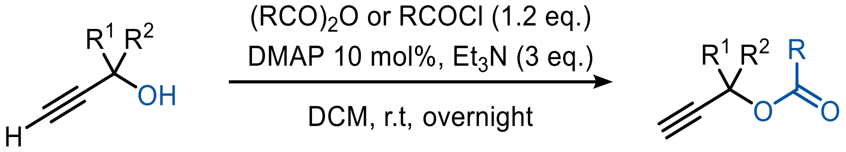

According to the literature procedure,[2] triethylamine (3 mmol, 1.5 eq.) was added dropwise to a solution of propargyl alcohol (2 mmol, 1 eq.) and DMAP (0.2 mmol, 10 mol%) in 3 mL anhydrous DCM. The resulting solution was stirred at 0°C for 10 min. After that, acyl chloride (for the synthesis of **1a-j**, **1l-q**, **1s**, **1u-w**,**1z, 1ac**) or anhydride acetic (2.4 mmol, 1.2 eq., for the synthesis of **1r**, **1t, 1y**, **1aa-ab**) was slowly added over 10 min and the reaction mixture was stirred at 25 °C overnight. The resulting solution was quenched with aqueous $NaHCO_3$ solution (10 mL), extracted with DCM (5 mL × 3) and dried over anhydrous Na_2SO_4. After the solvent was evaporated, the crude product was purified by column chromatography (hexane/ethyl acetate) to give the desired product.

General Procedure 3 (GP3)

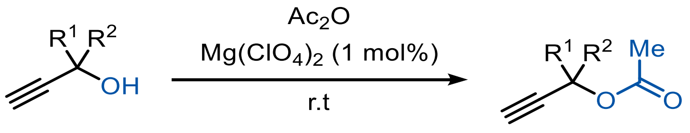

According to the literature procedure,[3] propargyl alcohol (2.5 mmol, 1 eq.) was added dropwise to a stirred solution of $Mg(ClO_4)_2$ (5.6 mg, 0.025 mmol, 1 mol%) in Ac_2O (0.24 mL, 2.5 mmol). After the starting material was consumed, as judged by TLC, the reaction crude was diluted with a solution of $NaHCO_3$ (0.1M) and extracted with Et_2O. For substrate **1k** the solvent was evaporated, and the crude product was used without further purification. For substrate **1x**, after solvent evaporation the crude product was purified by column chromatography (hexane/ethyl acetate) to give the desired product.

5.2 General procedure for the mechanochemical synthesis of 1,4 allenynes

General Procedure 4 (GP4)

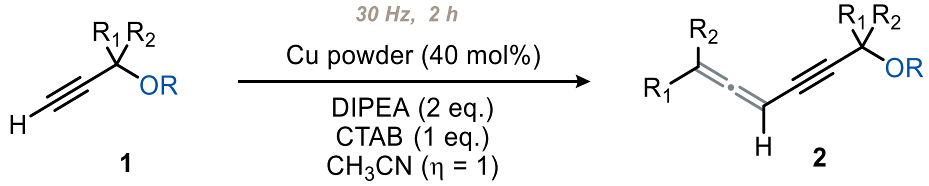

In a stainless-steel jar (V = 5 mL), copper powder (5,1 mg, 0.08 mmol, 40 mol%), CTAB (72.8 mg, 0.2 mmol, 1 eq.), CH_3CN (η = 1), DIPEA (70 µL, 0.4 mmol, 2 eq.) and substrate **1** (0.2 mmol, 1 eq.) were added together with one 10-mm hardened stainless-steel ball. The vessel was closed under air and was placed in the vibratory ball mill (Retsch MM400) set at 30 Hz. After 2 h, the jar was opened, and the mixture was recovered with EtOAc. The crude mixture was directly purified via flash column chromatography (eluent: Hexane/EtOAc gradient) to give compound **2**.

NB: After each use, stainless steel jars were rigorously cleaned with a 30% ammonium hydroxide solution to ensure complete removal of copper traces.

5.3 Late-stage manipulation of 1,4-allenynes

Deprotection of **2k**

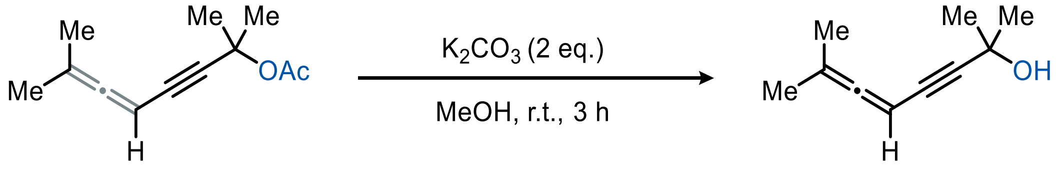

2k **3**, 90%

In a 10 mL tube open to air, K_2CO_3 (0.4 mmol, 2 eq.) was suspended in methanol (2 mL) and subsequently allenyne **2k** (0.2 mmol) was added dropwise at room temperature under stirring. The reaction was monitored via TLC and judged complete after 3 h. Then, the solvent was removed, and the reaction mixture was diluted with brine and extracted with Et_2O to afford pure product **3** in 90 % yield.

Gold-mediated cyclization of **2f**

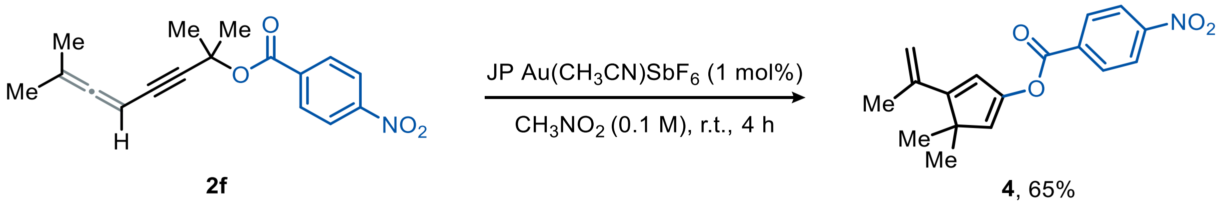

2f **4**, 65%

In a 10 mL Schlenk tube under N_2, JohnPhos Au(CH_3CN)SbF_6 (1 mol%), **2a** (0.2 mmol) and CH_3NO_2 (0.1 M) were sequentially added at room temperature under stirring. The tube was purged with N_2. The reaction tube was capped, and the reaction mixture was stirred for 4 h. After completion, the reaction crude material was purified via flash chromatography (silica gel, Hex:EA = 60:1), to give the title product **4** in 65% yield.

Rhodium-catalyzed carbonylative cyclization of **2a**

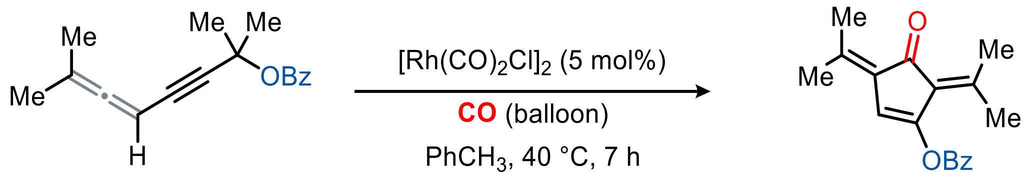

2a

5, 67%

To a 10 mL Schlenk tube under N_2, $[Rh(CO)_2Cl]_2$ (5 mol%) and allenyne **2a** (50.8 mg, 0.2 mmol) were dissolved in toluene (0.1 M). The tube was evacuated and filled with CO two times; a CO ballon (1 atm) was then mounted on the tube and the mixture was stirred for 4 h at 40 °C. After completion, the reaction crude was purified via flash chromatography (silica gel, Hex:EA = 20:1), to give the title product **5** in 67% yield.

6. Characterization of compounds

Substrates **1a-ac**

2-methylbut-3-yn-2-yl benzoate (1a)

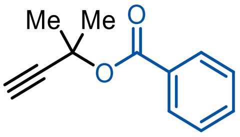

Prepared via GP2. Column chromatography: Hex:EtOAc 15:1 to yield 301.1 mg (80%) of the title compound as a colorless oil.

^{1}H NMR (400 MHz, $CDCl_3$) δ 8.02 – 7.97 (m, 2H), 7.54 – 7.45 (m, 1H), 7.37 (td, *J* = 7.6, 1.4 Hz, 2H), 2.58 (s, 1H), 1.79 (s, 6H). **^{13}C NMR** (101 MHz, $CDCl_3$) δ 165.0, 133.0, 130.9, 129.8, 128.4, 84.8, 72.7, 72.4, 29.2. Spectroscopic data are in accordance with the literature.[2]

2-methylbut-3-yn-2-yl 4-fluorobenzoate (1b)

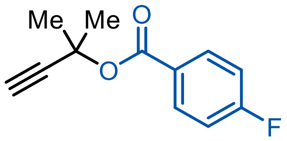

Prepared via GP2. Column chromatography: Hex:EtOAc, 17:1 to yield 309.3 mg (75%) of the title compound as a colorless oil.

^{1}H NMR (400 MHz, $CDCl_3$) δ 8.02 (dd, *J* = 8.8, 5.6 Hz, 2H), 7.11 – 7.05 (m, 2H), 2.58 (s, 1H), 1.81 (s, 6H). **^{13}C NMR** (101 MHz, $CDCl_3$) δ 165.8 (d, J = 254.0 Hz), 164.0, 132.3 (d, J = 9.4 Hz), 127.1 (d, J = 2.9 Hz), 115.5 (d, J = 22.2 Hz), 84.7, 72.8, 72.5, 29.1. **^{19}F NMR** (565 MHz, $CDCl_3$) δ -105.7 (tt, *J* = 8.5, 5.4 Hz). Spectroscopic data are in accordance with the literature.[4]

2-methylbut-3-yn-2-yl 4-chlorobenzoate (1c)

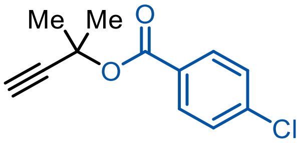

Prepared via GP2. Column chromatography: Hex:EtOAc 20:1 to yield 333.9 mg (75%) of the title compound as a colorless oil.

^{1}H NMR (400 MHz, $CDCl_3$) δ 7.97 – 7.88 (m, 2H), 7.40 – 7.32 (m, 2H), 2.58 (s, 1H), 1.79 (s, 6H). **^{13}C NMR** (101 MHz, $CDCl_3$) δ 164.0, 139.3, 131.0, 129.3, 128.7, 84.5, 72.9, 72.6, 29.0. Spectroscopic data are in accordance with the literature.[2]

2-methylbut-3-yn-2-yl 4-bromobenzoate (1d)

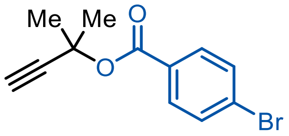

Prepared via GP2. Column chromatography: Hex:EtOAc, 15:1 to yield 347.2 mg (65%) of the title compound as a white solid.

^{1}H NMR (400 MHz, CDCl3) δ 7.91 – 7.83 (m, 2H), 7.60 – 7.52 (m, 2H), 2.59 (s, 1H), 1.81 (s, 6H).**^{13}C NMR** (101 MHz, $CDCl_3$) δ 164.2, 132.7, 131.7, 131.3, 129.8, 128.1, 84.5, 72.9, 29.1. Data is in accordance with literature.[5]

2-methylbut-3-yn-2-yl 4-methylbenzoate (1e)

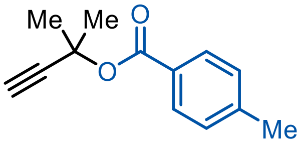

Prepared via GP2. Column chromatography: Hex:EtOAc, 20:1 to yield 303.3 mg (75%) of the title compound as a colorless oil.

^{1}H NMR (400 MHz, $CDCl_3$) δ 7.96 – 7.87 (m, 2H), 7.22 (dd, J = 8.6, 0.7 Hz, 2H), 2.58 (s, 3H), 2.40 (s, 1H), 1.81 (s, 6H).**^{13}C NMR** (101 MHz, $CDCl_3$) δ 165.1, 143.6, 129.8, 129.2, 128.2, 85.0, 72.5, 72.1, 29.2, 21.8. Spectroscopic data are in accordance with the literature.[4]

2-methylbut-3-yn-2-yl 4-nitrobenzoate (1f)

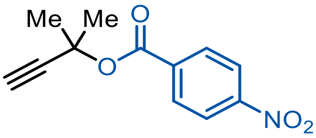

Prepared via GP2. Column chromatography: Hex:EtOAc, 60:1 to yield 396.4 mg (85%) of the title compound as a white solid.

^{1}H NMR (400 MHz, $CDCl_3$) δ 8.37 – 8.22 (m, 2H), 8.22 – 8.13 (m, 2H), 2.62 (s, 1H), 1.84 (s, 6H).**^{13}C NMR** (101 MHz, $CDCl_3$) δ 163.0, 150.6, 136.3, 130.8, 123.6, 84.1, 73.7, 73.3, 29.2. Spectroscopic data are in accordance with the literature.[6]

2-methylbut-3-yn-2-yl 2-methylbenzoate (1g)

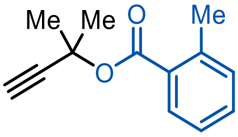

Prepared via GP2. Column chromatography: Hex:EtOAc 15:1 to yield 262.8 mg (65%) of the title compound as a colorless oil.

^{1}H NMR (400 MHz, $CDCl_3$) δ 7.87 (dd, *J* = 8.1, 1.7 Hz, 1H), 7.38 (t, *J* = 8.4 Hz, 1H), 7.25 – 7.19 (m, 2H), 2.62 (s, 3H), 2.59 (s, 1H), 1.82 (s, 6H). **^{13}C NMR** (101 MHz, $CDCl_3$) δ 166.1, 140.2, 132.0, 131.8, 130.7, 130.3, 125.8, 84.9, 72.6, 72.2, 29.2, 21.9.

2-methylbut-3-yn-2-yl furan-2-carboxylate (1h)

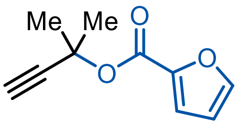

Prepared, following the general procedure GP2. The crude product was purified *via* column chromatography (Hex:EtOAc, 12:1) to yield 302.9 mg (85%) of the title compound as a colorless oil.

^{1}H NMR (400 MHz, $CDCl_3$) δ 7.55 (dd, *J* = 1.8, 0.9 Hz, 1H), 7.14 (dd, *J* = 3.5, 0.9 Hz, 1H), 6.48 (dd, *J* = 3.5, 1.7 Hz, 1H), 2.58 (s, 1H), 1.79 (d, *J* = 1.1 Hz, 6H). **^{13}C NMR** (101 MHz, $CDCl_3$) δ 156.8, 146.0, 144.8, 117.7, 111.6, 84.1, 72.7, 72.7, 28.9. Spectroscopic data are in accordance with the literature.[2]

2-methylbut-3-yn-2-yl thiophene-2-carboxylate (1i)

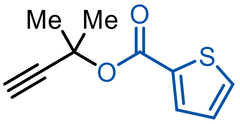

Prepared via GP2. Column chromatography: Hex:EtOAc, 30:1 to yield 112.6 mg (29%) of the title compound as a colorless oil.

^{1}H NMR (400 MHz, $CDCl_3$) δ 7.76 (dd, *J* = 3.7, 1.3 Hz, 1H), 7.52 (dd, *J* = 5.0, 1.3 Hz, 1H), 7.07 (dd, *J* = 5.0, 3.7 Hz, 1H), 2.58 (s, 1H), 1.78 (s, 6H). **^{13}C NMR** (101 MHz, $CDCl_3$) δ 160.6, 134.5, 133.7, 132.4, 127.7, 84.5, 72.9, 72.8, 29.2. Spectroscopic data are in accordance with the literature.[2]

2-methylbut-3-yn-2-yl picolinate (1j)

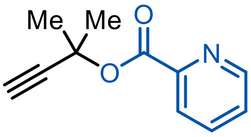

Prepared via GP2. Column chromatography: Hex:EtOAc, 25:1 to yield 113.5 mg (30%) of the title compound as a white solid.

^{1}H NMR (400 MHz, $CDCl_3$) δ 8.72 (d, *J* = 4.6 Hz, 1H), 8.07 (d, *J* = 7.8 Hz, 1H), 7.79 (td, *J* = 7.7, 1.8 Hz, 1H), 7.42 (ddd, *J* = 7.7, 4.7, 1.3 Hz, 1H), 2.58 (s, 1H), 1.84 (s, 6H). **^{13}C NMR** (101 MHz, $CDCl_3$) δ 163.5, 149.9, 148.7, 137.0, 126.8, 125.2, 84.4, 73.4, 73.1, 29.0.

2-methylbut-3-yn-2-yl acetate (1k)

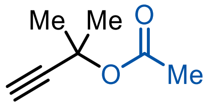

Prepared via GP3. The crude product yielded 249 mg of the title compound as a volatile colorless oil. Traces of AcOH were detected.

^{1}H NMR (400 MHz, $CDCl_3$) δ 2.45 (s, 1H), 1.92 (s, 3H), 1.56 (s, 6H).**^{13}C NMR** (101 MHz, $CDCl_3$) δ 176.7, 84.6, 72.3, 71.5, 29.0, 21.7. Spectroscopic data are in accordance with the literature.[3]

2-methylbut-3-yn-2-yl butyrate (1l)

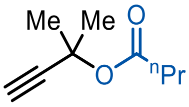

Prepared via GP2. Column chromatography: Hex:EtOAc, 20:1 to yield 185.0 mg (60%) of the title compound as a volatile colorless oil.

^{1}H NMR (400 MHz, $CDCl_3$) δ 2.50 (s, 1H), 2.23 (t, J = 7.4 Hz, 2H), 1.75 – 1.54 (m, 8H), 0.93 (t, J = 7.5 Hz, 3H).**^{13}C NMR** (101 MHz, $CDCl_3$) δ 172.1, 85.0, 72.2, 71.4, 36.9, 29.0, 18.5, 13.7. Spectroscopic data are in accordance with the literature.

2-methylbut-3-yn-2-yl cyclopropanecarboxylate (1m)

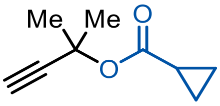

Prepared via GP2. Column chromatography: Hex:EtOAc 20:1 to yield 197.8 mg (65%) of the title compound as a volatile colorless oil.

^{1}H NMR (400 MHz, $CDCl_3$) δ 2.45 (d, J = 3.4 Hz, 1H), 1.59 (d, J = 5.0 Hz, 6H), 1.53 – 1.41 (m, 1H), 0.94 – 0.85 (m, 2H), 0.81 – 0.69 (m, 2H).**^{13}C NMR** (101 MHz, $CDCl_3$) δ 173.0, 84.8, 72.2, 71.4, 29.1, 13.3, 8.2.

2-methylbut-3-yn-2-yl (E)-but-2-enoate (1n)

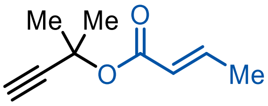

Prepared via GP2. Column chromatography: Hex:EtOAc 60:1 to yield 258.7 mg (85%) of the title compound as a volatile colorless oil.

^{1}H NMR (400 MHz, $CDCl_3$) δ 6.95 (dq, J = 15.0, 6.6 Hz, 1H), 5.85 – 5.72 (m, 1H), 2.53 (d, J = 4.1 Hz, 1H), 1.91 – 1.80 (m, 3H), 1.69 (s, 6H).**^{13}C NMR** (101 MHz, $CDCl_3$) δ 165.0, 144.8, 123.4, 85.0, 72.3, 71.5, 29.1, 18.0.

2-methylbut-3-yn-2-yl cinnamate (1o)

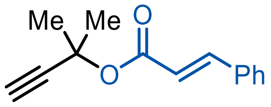

Prepared via GP2. Column chromatography: Hex:EtOAc 60:1 to yield 364.1 mg (85%) of the title compound as a colorless oil.

^{1}H NMR (400 MHz, CDCl3) δ 7.64 (d, J = 16.0 Hz, 1H), 7.52 – 7.42 (m, 2H), 7.39 – 7.29 (m, 3H), 6.36 (d, J = 16.0 Hz, 1H), 2.57 (s, 1H), 1.74 (s, 6H).**^{13}C NMR** (101 MHz, $CDCl_3$) δ 165.2, 144.7, 134.3, 130.2, 128.8, 128.0, 118.7, 84.7, 72.5, 71.7, 29.0.

2-methylbut-3-yn-2-yl methacrylate (1p)

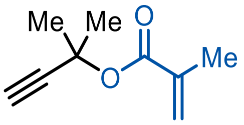

Prepared via GP2. Column chromatography: Hex:EtOAc 10:1 to yield 39.5 mg 13% of the title compound as a volatile colorless oil.

^{1}H NMR (400 MHz, $CDCl_3$) δ 6.09 – 6.04 (m, 1H), 5.57 – 5.51 (m, 1H), 2.53 (s, 1H), 1.92 (s, 3H), 1.71 (s, 6H).**^{13}C NMR** (101 MHz, $CDCl_3$) δ 165.8, 137.1, 125.5, 84.8, 72.4, 71.8, 29.0, 18.2.

3-methylpent-1-yn-3-yl benzoate (1q)

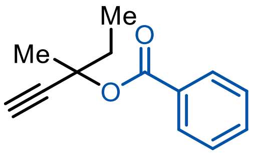

Prepared via GP2. Column chromatography: Hex:EtOAc 15:1 to yield 141.5 mg (35%) of the title compound as a colorless oil.

^{1}H NMR (400 MHz, $CDCl_3$) δ 8.07 – 7.99 (m, 2H), 7.54 (ddt, *J* = 9.2, 6.7, 1.4 Hz, 1H), 7.42 (td, *J* = 7.6, 1.1 Hz, 2H), 2.60 (s, 1H), 2.13 (dt, *J* = 14.6, 7.2 Hz, 1H), 2.05 – 1.94 (m, 1H), 1.81 (d, *J* = 0.8 Hz, 3H), 1.17 – 1.09 (m, 3H).**^{13}C NMR** (101 MHz, $CDCl_3$) δ 164.9, 133.0, 131.0, 129.7, 128.4, 83.8, 75.9, 73.6, 34.8, 26.2, 8.6.

2-phenylbut-3-yn-2-yl acetate (1r)

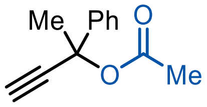

Prepared via GP2. Column chromatography: Hex:EtOAc 5:1 to yield 368.8 mg (98%) of the title compound as a yellow oil.

^{1}H NMR (400 MHz, $CDCl_3$) δ 7.63 – 7.56 (m, 2H), 7.42 – 7.34 (m, 1H), 7.33 – 7.27 (m, 2H), 2.83 (s, 1H), 2.08 (s, 3H), 1.91 (s, 3H). **^{13}C NMR** (101 MHz, $CDCl_3$) δ 168.6, 142.1, 128.4, 127.9, 124.7, 82.9, 75.6, 75.3, 31.8, 22.1. Spectroscopic data are in accordance with the literature.[2]

1-ethynylcyclobutyl benzoate (1s)

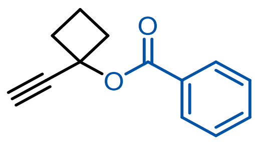

Prepared via GP2 from propargyl alcohol prepared via GP1. Column chromatography: Hex:EtOAc 20:1 to yield 280.2 mg (70%) of the title compound as a colorless oil.

^{1}H NMR (400 MHz, $CDCl_3$) δ 8.08 – 8.01 (m, 2H), 7.60 – 7.51 (m, 1H), 7.43 (t, *J* = 7.9 Hz, 2H), 2.78 – 2.66 (m, 2H), 2.62 (s, 1H), 2.56 (dt, *J* = 12.6, 9.7 Hz, 2H), 2.14 – 1.89 (m, 2H). **^{13}C NMR** (101 MHz, $CDCl_3$) δ 164.9, 133.2, 130.2, 130.1, 128.4, 84.0, 72.9, 72.0, 36.8, 14.5. Spectroscopic data are in accordance with the literature.[7]

tert-butyl 3-acetoxy-3-ethynylpyrrolidine-1-carboxylate (1t)

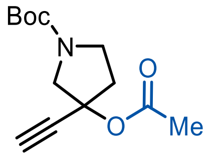

Prepared via GP2. Column chromatography: Hex:EtOAc 4:1 to yield 304.1 mg (60%) of the title compound as a colorless oil as a mixture of rotamers.

^{1}H NMR (400 MHz, $CDCl_3$) δ 3.94 – 3.69 (m, 2H), 3.62 – 3.30 (m, 2H), 2.65 (s, 1H), 2.51 (dd, *J* = 13.2, 6.5 Hz, 1H), 2.35 (td, *J* = 14.7, 8.1 Hz, 1H), 2.09 (s, 3H), 1.48 (s, 9H). **^{13}C NMR** (101 MHz, $CDCl_3$) δ 169.6 (M+m), 154.3 (M+m), 80.6 (M+m), 80.1 (M), 80.0 (m), 75.9 (M+m), 74.8 (M+m), 57.0 (M+m), 44.0 (m), 43.5 (M), 38.2 (m), 37.8 (M), 28.6 (M+m), 21.6 (M+m).

1-ethynylcyclohexyl benzoate (1u)

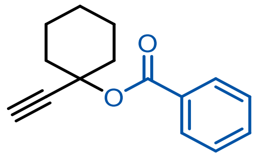

Prepared via GP2. Column chromatography: Hex:EtOAc 10:1 to yield 136.9 mg (30%) of the title compound as a waxy solid.

^{1}H NMR (400 MHz, $CDCl_3$) δ 8.04 (dd, *J* = 8.4, 1.4 Hz, 2H), 7.60 – 7.51 (m, 1H), 7.48 – 7.38 (m, 2H), 2.65 (s, 1H), 2.30 – 2.19 (m, 2H), 2.10 (p, *J* = 6.7 Hz, 2H), 1.70 (p, *J* = 6.1 Hz, 4H), 1.57 – 1.49 (m, 1H), 1.44 (s, 1H). **^{13}C NMR** (101 MHz, $CDCl_3$) δ 164.8, 133.0, 131.1, 129.8, 128.4, 83.9, 75.6, 74.5, 37.2, 25.3, 22.6. Spectroscopic data are in accordance with the literature.[1]

1-ethynylcyclohexyl 4-fluorobenzoate (1v)

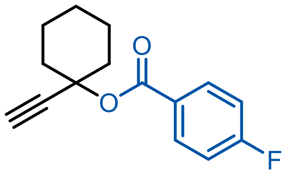

Prepared via GP2. Column chromatography: Hex:EtOAc 15:1 to yield 88.6 mg (18%) of the title compound as a colorless oil.

^{1}H NMR (400 MHz, $CDCl_3$) δ 8.09 – 7.99 (m, 2H), 7.15 – 7.04 (m, 2H), 2.65 (s, 1H), 2.22 (dt, *J* = 11.7, 5.5 Hz, 2H), 2.06 (p, *J* = 6.9 Hz, 2H), 1.69 (p, *J* = 6.3 Hz, 4H), 1.54 (dp, *J* = 10.9, 5.6 Hz, 1H), 1.41 (dq, *J* = 12.8, 6.7 Hz, 1H). **^{13}C NMR** (101 MHz, $CDCl_3$) δ 165.8 (d, *J* = 253.6 Hz), 132.3 (d, *J* = 9.1 Hz), 127.3 (d, *J* = 3.3 Hz), 115.5 (d, *J* = 21.8 Hz), 83.7, 75.9, 74.6, 37.1, 25.2, 22.6. **^{19}F** (565 MHz, $CDCl_3$) δ -105.89.

1-ethynylcyclohexyl 4-chlorobenzoate (1w)

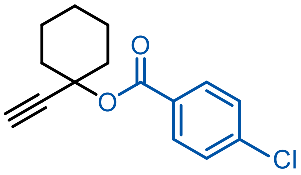

Prepared via GP2. Column chromatography: Hex:EtOAc 20:1 to yield 136.6 mg (26%) of the title compound as a colorless oil.

^{1}H NMR (400 MHz, $CDCl_3$) δ 8.00 – 7.91 (m, 2H), 7.43 – 7.35 (m, 2H), 2.64 (s, 1H), 2.22 (dt, *J* = 12.1, 5.6 Hz, 2H), 2.05 (p, *J* = 6.7 Hz, 2H), 1.68 (p, *J* = 6.2 Hz, 4H), 1.60 – 1.47 (m, 1H), 1.40 (dq, *J* = 13.1, 6.7 Hz, 1H). **^{13}C NMR** (101 MHz, $CDCl_3$) δ 163.8, 139.4, 130.8, 129.7, 128.4, 83.6, 76.0, 74.7, 37.2, 25.2, 22.5.

1-ethynylcyclohexyl acetate (1x)

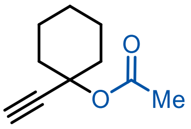

Prepared via GP3. Column chromatography: Hex:EtOAc 17:1 to yield 292.5 mg (88%) of the title compound as a colorless oil.

^{1}H NMR (400 MHz, $CDCl_3$) δ 2.51 (s, 1H), 2.06 – 1.96 (m, 2H), 1.96 – 1.89 (m, 3H), 1.74 (q, *J* = 6.2 Hz, 2H), 1.52 (d, *J* = 7.0 Hz, 4H), 1.46 – 1.07 (m, 2H). **^{13}C NMR** (101 MHz, $CDCl_3$) δ 168.9, 83.4, 74.9, 74.2, 36.9, 24.8, 22.3, 21.7. Spectroscopic data are in accordance with the literature.[1]

4-ethynyltetrahydro-2H-pyran-4-yl acetate (1y)

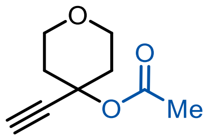

Prepared via GP2. Column chromatography: Hex:EtOAc 4:1 to yield 218.6 mg (65%) of the title compound as a colorless oil.

^{1}H NMR (400 MHz, $CDCl_3$) δ 3.87 (dt, *J* = 12.1, 4.4 Hz, 2H), 3.75 (ddd, *J* = 12.0, 9.3, 2.8 Hz, 2H), 2.69 (s, 1H), 2.25 (dddd, *J* = 13.0, 4.8, 2.9, 1.7 Hz, 2H), 2.09 (s, 3H), 2.03 (ddd, *J* = 13.3, 9.3, 4.1 Hz, 2H). **^{13}C NMR** (101 MHz, $CDCl_3$) δ 169.2, 82.3, 75.2, 72.2, 64.3, 37.4, 21.8.

1-ethynylcyclohexyl cinnamate (1z)

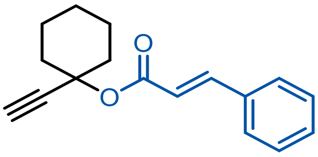

Prepared via GP2. Column chromatography: Hex:EtOAc 20:1 to yield 203.4 mg (40%) of the title compound as a yellow oil.

^{1}H NMR (400 MHz, $CDCl_3$) δ 7.67 (d, *J* = 16.0 Hz, 1H), 7.52 (dd, *J* = 6.7, 3.2 Hz, 2H), 7.43 – 7.34 (m, 3H), 6.42 (d, *J* = 16.0 Hz, 1H), 2.64 (s, 1H), 2.21 (dt, *J* = 11.7, 5.5 Hz, 2H), 1.98 (q, *J* = 7.1 Hz, 2H), 1.67 (h, *J* = 4.7 Hz, 4H), 1.61 – 1.48 (m, 1H), 1.38 (dp, *J* = 13.3, 6.7 Hz, 1H).**^{13}C NMR** (101 MHz, $CDCl_3$) δ 165.3, 144.9, 134.6, 130.4, 129.1, 128.2, 119.0, 83.9, 75.4, 74.4, 37.2, 25.3, 22.6.

2-ethynyladamantan-2-yl acetate (1aa)

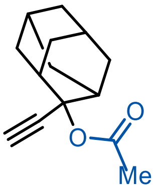

Prepared via GP2 from propargyl alcohol prepared via GP1. Column chromatography Hex:EtOAc 10:1 to yield 370.9 mg (85%) of the title compound as a white solid. **mp**: 61.8-63.4°C.

^{1}H NMR (400 MHz, $CDCl_3$) δ 2.64 (s, 1H), 2.44 (t, *J* = 3.3 Hz, 2H), 2.24 – 2.15 (m, 2H), 2.07 (s, 3H), 2.00 – 1.92 (m, 2H), 1.86 – 1.67 (m, 6H), 1.64 – 1.55 (m, 2H).**^{13}C NMR** (101 MHz, $CDCl_3$) δ 169.3, 83.9, 79.8, 75.1, 37.5, 35.9, 34.7, 31.9, 26.8, 26.4, 21.9. Spectroscopic data are in accordance with the literature.[8]

3,7-dimethyloct-6-en-1-yn-3-yl acetate (1ab)

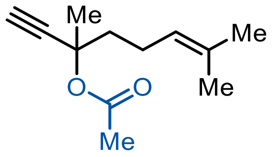

Prepared via GP2. Column chromatography: Hex:EtOAc 20:1 to yield 213.6 mg (55%) of the title compound as a colorless oil.

^{1}H NMR (400 MHz, $CDCl_3$) δ 5.14 – 5.04 (m, 1H), 2.54 (s, 1H), 2.21 – 2.07 (m, 2H), 2.00 (s, 3H), 1.93 (ddd, *J* = 13.6, 10.0, 6.5 Hz, 1H), 1.85 – 1.72 (m, 1H), 1.66 (s, 6H), 1.60 (s, 3H). **^{13}C NMR** (101 MHz, $CDCl_3$) δ 169.4, 132.3, 123.2, 83.9, 75.0, 73.4, 41.4, 26.4, 25.7, 23.0, 22.0, 17.7. Spectroscopic data are in accordance with the literature.[9]

(3R,5S,7R,8R,9S,10S,12S,13R,14S,17R)-10,13-dimethyl-17-((R)-5-((2-methylbut-3-yn-2-yl)oxy)-5-oxopentan-2-yl)hexadecahydro-1H-cyclopenta[a]phenanthrene-3,7,12-triyl triacetate (1ac)

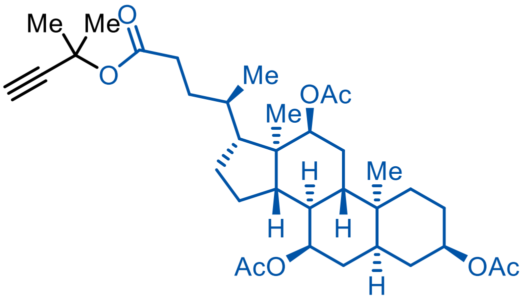

Prepared via GP2. Column chromatography: Hex:EtOAc 4:1 gradient to 3:1 to yield 180 mg (15%) of the title compound as a white solid. **mp**: 221.6 - 223.8°C.

^{1}H NMR (400 MHz, $CDCl_3$) δ 5.13 – 4.99 (m, 1H), 4.85 (q, *J* = 3.0 Hz, 1H), 4.51 (tt, *J* = 11.3, 4.3 Hz, 1H), 2.48 (s, 1H), 2.25 (ddd, *J* = 14.9, 9.4, 5.2 Hz, 1H), 2.14 (d, *J* = 2.0 Hz, 1H), 2.08 (s, 3H), 2.02 (s, 3H), 1.98 (d, *J* = 2.2 Hz, 3H), 1.95 (d, *J* = 5.0 Hz, 2H), 1.91 – 1.77 (m, 3H), 1.60 (s, 9H), 1.58 – 1.52 (m, 3H), 1.45 (s, 2H), 1.35 (qd, *J* = 6.9, 4.1 Hz, 2H), 1.29 – 1.15 (m, 5H), 1.09 – 0.96 (m, 2H), 0.86 (s, 3H), 0.76 (d, *J* = 6.5 Hz, 3H), 0.67 (s, 3H). **^{13}C NMR** (101 MHz, $CDCl_3$) δ 172.2, 170.4 (2C), 170.2, 84.7, 77.4, 75.3, 74.0, 72.2, 71.3, 70.8, 47.3, 45.0, 43.3, 40.9, 37.7, 34.6, 34.6, 34.4, 34.3, 31.7, 31.2, 30.5, 28.9, 28.8, 27.1, 26.8, 25.5, 22.7, 22.5, 21.5, 21.4, 21.4, 17.5, 12.2.

Products **2a-ac**

2,7-dimethylocta-5,6-dien-3-yn-2-yl benzoate (2a)

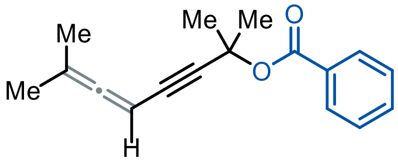

Prepared via GP4. Column chromatography: Hex:EtOAc 39:1 to yield 20.8 mg (82%) of the title compound as a colorless oil. The reaction was also repeated on a 1 mmol scale (of **1a**) with the very same experimental setup (milling time: 3 h) and obtained 83 mg (65%) of the title compound.

^{1}H NMR (400 MHz, $CDCl_3$) δ 8.06 – 7.97 (m, 2H), 7.58 – 7.48 (m, 1H), 7.42 (t, *J* = 7.6 Hz, 2H), 5.32 – 5.23 (p, *J* = 2.9 Hz, 1H), 1.82 (s, 6H), 1.71 (d, *J* = 2.9 Hz, 6H). **^{13}C NMR** (101 MHz, $CDCl_3$) δ 210.3, 164.9, 132.8, 131.3, 129.7, 128.3, 97.7, 89.4, 78.6, 73.5, 73.1, 29.4, 20.1.

2,7-dimethylocta-5,6-dien-3-yn-2-yl 4-fluorobenzoate (2b)

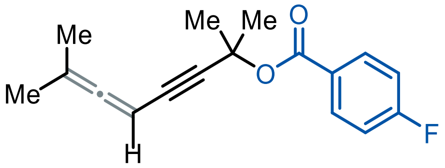

Prepared via GP4. Column chromatography: Hex:EtOAc 59:1 to yield 12.2 mg (45%) of the title compound as a waxy solid. **mp:** 43.2-44.4 °C.

^{1}H NMR (400 MHz, $CDCl_3$) δ 8.08 – 7.98 (m, 2H), 7.14 – 7.03 (m, 2H), 5.28 (p, *J* = 2.9 Hz, 1H), 1.81 (s, 6H), 1.72 (d, *J* = 2.9 Hz, 6H). **^{13}C NMR** (101 MHz, $CDCl_3$) δ 210.3, 165.8 (d, *J* = 253.6 Hz), 164.0, 132.3 (d, *J* = 9.4 Hz), 127.5 (d, *J* = 3.3 Hz), 115.5 (d, *J* = 21.8 Hz), 97.8, 89.3, 78.8, 73.7, 73.0, 29.4, 20.1. **^{19}F NMR** (565 MHz, $CDCl_3$) δ -106.5.

2,7-dimethylocta-5,6-dien-3-yn-2-yl 4-chlorobenzoate (2c)

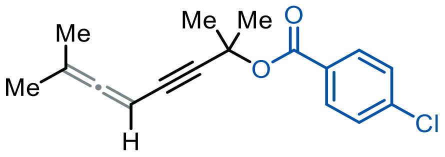

Prepared via GP4. Column chromatography: Hex:EtOAc 59:1 to yield 24.4 mg (85%) of the title compound as a waxy solid. **mp:** 48.7 -50.3°C.

^{1}H NMR (400 MHz, $CDCl_3$) δ 7.99 – 7.89 (m, 2H), 7.43 – 7.34 (m, 2H), 5.27 (p, *J* = 2.9 Hz, 1H), 1.81 (s, 6H), 1.70 (d, *J* = 2.9 Hz, 6H). **^{13}C NMR** (101 MHz, $CDCl_3$ δ 210.3, 164.0, 139.2, 130.8, 129.7, 128.6, 97.8, 89.1, 78.8, 73.3, 73.0, 29.3, 20.0.

2,7-dimethylocta-5,6-dien-3-yn-2-yl 4-bromobenzoate (2d)

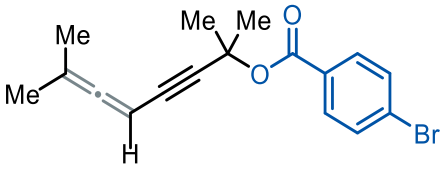

Prepared via GP4. Column chromatography: Hex:EtOAc 39:1 to yield 20.3 mg (61%) of the title compound as a waxy solid. **mp:** 40.3 -44.3 °C.

^{1}H NMR (400 MHz, $CDCl_3$) δ 7.93 – 7.82 (m, 2H), 7.61 – 7.52 (m, 2H), 5.27 (hept, *J* = 2.9 Hz, 1H), 1.81 (s, 6H), 1.72 (d, *J* = 2.9 Hz, 6H). **^{13}C NMR** (101 MHz, $CDCl_3$) δ 210.4, 164.2, 131.7, 131.3, 130.2, 127.9, 97.8, 89.1, 78.9, 73.9, 73.0, 29.3, 20.1.

2,7-dimethylocta-5,6-dien-3-yn-2-yl 4-methylbenzoate (2e)

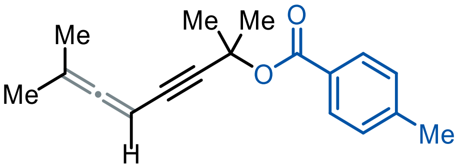

Prepared via GP4. Column chromatography: Hex:EtOAc 39:1 to yield 23.5 mg (88%) of the title compound as a colorless oil.

^{1}H NMR (400 MHz, $CDCl_3$) δ 7.96 – 7.86 (m, 2H), 7.25 – 7.18 (m, 2H), 5.28 (hept, *J* = 2.9 Hz, 1H), 2.40 (s, 3H), 1.81 (s, 6H), 1.71 (d, *J* = 2.9 Hz, 6H). **^{13}C NMR** (101 MHz, $CDCl_3$) δ 210.3, 165.0, 143.4, 129.8, 129.1, 128.6, 97.7, 89.6, 78.5, 73.3, 73.1, 29.4, 21.8, 20.1.

2,7-dimethylocta-5,6-dien-3-yn-2-yl 4-nitrobenzoate (2f)

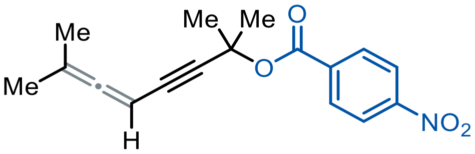

Prepared via GP4. Column chromatography: Hex:EtOAc 29:1 to yield 19.4 mg (65%) of the title compound as a white solid. **mp**: 94.2-96.0 °C.

^{1}H NMR (400 MHz, $CDCl_3$) δ 8.29 – 8.21 (m, 2H), 8.21 – 8.12 (m, 2H), 5.25 (h, *J* = 2.9 Hz, 1H), 1.83 (s, 6H), 1.70 (d, *J* = 2.9 Hz, 6H). **^{13}C NMR** (101 MHz, $CDCl_3$) δ 210.4, 162.9, 150.5, 136.7, 130.8, 123.5, 97.9, 88.4, 79.3, 74.9, 72.8, 29.2, 20.0.

An HMBC spectrum was also taken to ascertain the allenyne structure and is displayed in Section 9. The long range costant (*J*) was adjusted to 20 Hz in order to develop the coupling with the carbon at 210 ppm.

2,7-dimethylocta-5,6-dien-3-yn-2-yl 2-methylbenzoate (2g)

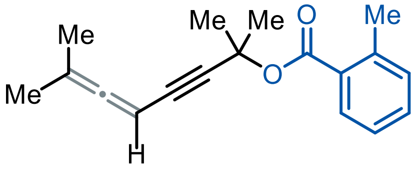

Prepared via GP4. Column chromatography: Hex:EtOAc 59:1 to yield 20.3 mg (76%) of the title compound as a colorless oil.

^{1}H NMR (400 MHz, $CDCl_3$) δ 7.87 (dd, *J* = 8.1, 1.7 Hz, 1H), 7.37 (td, *J* = 7.3, 1.6 Hz, 1H), 7.22 (t, *J* = 7.0 Hz, 2H), 5.29 (hept, *J* = 2.9 Hz, 1H), 2.60 (s, 3H), 1.82 (s, 6H), 1.72 (d, *J* = 2.9 Hz, 6H). **^{13}C NMR** (101 MHz, $CDCl_3$) δ 210.3, 166.1, 140.0, 131.8, 131.7, 130.8, 130.6, 125.7, 97.6, 89.5, 78.6, 73.3, 73.1, 29.4, 21.9, 20.0.

2,7-dimethylocta-5,6-dien-3-yn-2-yl furan-2-carboxylate (2h)

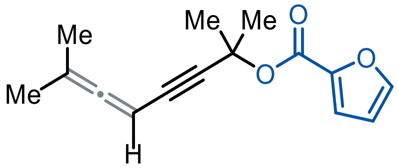

Prepared via GP4. Column chromatography: Hex:EtOAc 15:1 to yield 17.0 mg (70%) of the title compound as a waxy solid. **mp:** 60.3-62.5 °C.

^{1}H NMR (400 MHz, $CDCl_3$) δ 7.54 (dd, *J* = 1.8, 0.9 Hz, 1H), 7.11 (dd, *J* = 3.5, 0.9 Hz, 1H), 6.47 (dd, *J* = 3.4, 1.7 Hz, 1H), 5.25 (hept, *J* = 2.9 Hz, 1H), 1.79 (s, 6H), 1.70 (d, *J* = 3.1 Hz, 6H). **^{13}C NMR** (101 MHz, $CDCl_3$) δ 210.3, 157.2, 146.1, 145.3, 117.5, 111.8, 97.7, 89.0, 79.0, 74.1, 73.0, 29.3, 20.0.

2,7-dimethylocta-5,6-dien-3-yn-2-yl thiophene-2-carboxylate (2i)

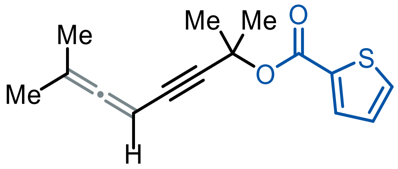

Prepared via GP4. Column chromatography: Hex:EtOAc 29:1 to yield 22.8 mg (88%) of the title compound as a waxy solid. **mp:** 43.1-45.7 °C.

^{1}H NMR (400 MHz, $CDCl_3$) δ 7.76 (dd, *J* = 3.7, 1.3 Hz, 1H), 7.52 (dd, *J* = 5.0, 1.3 Hz, 1H), 7.07 (dd, *J* = 5.0, 3.8 Hz, 1H), 5.27 (hept, *J* = 2.9 Hz, 1H), 1.80 (s, 6H), 1.71 (d, *J* = 2.9 Hz, 6H). **^{13}C NMR** (101 MHz, $CDCl_3$) δ 210.3, 160.7, 135.0, 133.4, 132.2, 127.7, 97.7, 89.2, 78.8, 74.2, 73.0, 29.4, 20.1.

2,7-dimethylocta-5,6-dien-3-yn-2-yl picolinate (2j)

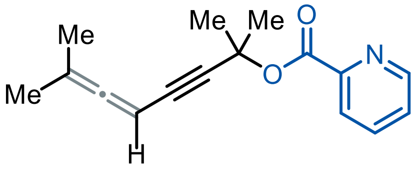

Prepared via GP4. Column chromatography: Hex:EtOAc 10:1 to yield 5.1 mg (20%) of the title compound as a colorless oil.

^{1}H NMR (400 MHz, $CDCl_3$) δ 8.8 (ddd, J = 4.8, 1.8, 0.9 Hz, 1H), 8.1 (dt, J = 7.8, 1.1 Hz, 1H), 7.8 (td, J = 7.8, 1.8 Hz, 1H), 7.5 (ddd, J = 7.6, 4.8, 1.2 Hz, 1H), 5.3 (hept, J = 2.9 Hz, 1H), 1.9 (s, 6H), 1.7 (d, J = 2.9 Hz, 6H). **^{13}C NMR** (101 MHz, $CDCl_3$) δ 210.4, 163.5, 150.0, 149.1, 137.0, 126.7, 125.2, 97.7, 89.0, 79.1, 74.7, 73.0, 29.3, 20.1.

2,7-dimethylocta-5,6-dien-3-yn-2-yl acetate (2k)

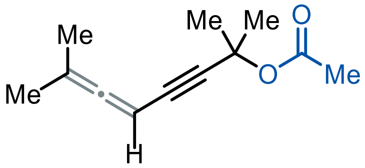

Prepared via GP4. Column chromatography: Hex:EtOAc 19:1 to yield 15.3 mg (80%) of the title compound as a colorless oil.

^{1}H NMR (400 MHz, $CDCl_3$) δ 5.17 (hept, *J* = 2.9 Hz, 1H), 1.93 (s, 3H), 1.64 (d, *J* = 3.1 Hz, 6H), 1.60 (s, 6H).**^{13}C NMR** (101 MHz, CDCl3) δ 210.1, 169.2, 97.2, 89.3, 78.1, 72.9, 72.7, 29.2, 21.9, 19.6.

2,7-dimethylocta-5,6-dien-3-yn-2-yl butyrate (2l)

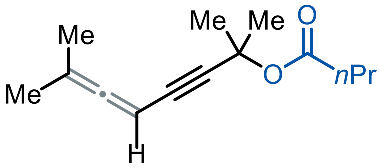

Prepared via GP4. Column chromatography: Hex:EtOAc 59:1 to yield 11.2 mg (51%) of the title compound as a colorless oil.

^{1}H NMR (400 MHz, $CDCl_3$) δ 5.24 (h, *J* = 3.0 Hz, 1H), 2.23 (t, *J* = 7.4 Hz, 2H), 1.71 (d, *J* = 2.9 Hz, 6H), 1.67 (s, 6H), 1.62 (q, *J* = 7.3 Hz, 2H), 0.94 (t, *J* = 7.4 Hz, 3H).**^{13}C NMR** (101 MHz, $CDCl_3$) δ 210.2, 172.2, 97.6, 89.6, 78.2, 73.1, 72.7, 37.1, 29.2, 20.1, 18.6, 13.7.

2,7-dimethylocta-5,6-dien-3-yn-2-yl cyclopropanecarboxylate (2m)

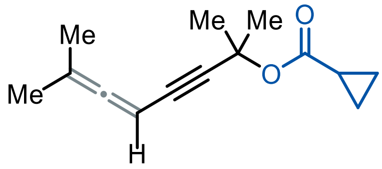

Prepared via GP4. Column chromatography: Hex:EtOAc 39:1 to yield 13.5 mg (62%) of the title compound as a colorless oil.

^{1}H NMR (400 MHz, $CDCl_3$) δ 5.24 (h, *J* = 2.9 Hz, 1H), 1.70 (d, *J* = 2.9 Hz, 6H), 1.66 (s, 6H), 1.53 (ddd, *J* = 12.8, 8.1, 4.7 Hz, 1H), 1.00 – 0.90 (m, 2H), 0.85 – 0.74 (m, 2H). **^{13}C NMR** (101 MHz, $CDCl_3$) δ 210.2, 173.1, 97.6, 89.6, 78.2, 73.1, 72.8, 29.2, 20.0, 13.7, 8.4.

2,7-dimethylocta-5,6-dien-3-yn-2-yl (E)-but-2-enoate (2n)

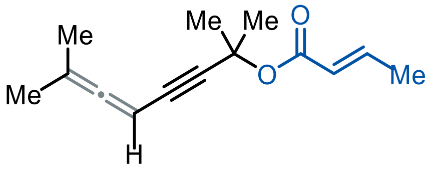

Prepared via GP4. Column chromatography: Hex:EtOAc 29:1 to yield 12.6 mg (58%) of the title compound as a colorless oil.

^{1}H NMR (400 MHz, $CDCl_3$) δ 6.93 (dq, *J* = 15.5, 6.8 Hz, 1H), 5.79 (dq, *J* = 15.5, 1.7 Hz, 1H), 5.26 (hept, *J* = 2.9 Hz, 1H), 1.85 (dd, *J* = 7.0, 1.7 Hz, 3H), 1.77 – 1.64 (m, 12H). **^{13}C NMR** (101 MHz, $CDCl_3$) δ 210.2, 165.0, 144.4, 123.7, 97.6, 89.6, 78.3, 73.1, 72.7, 29.3, 20.1, 18.0.

2,7-dimethylocta-5,6-dien-3-yn-2-yl cinnamate (2o)

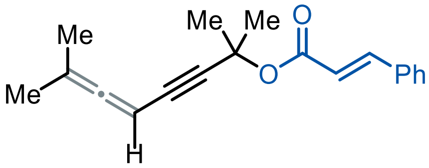

Prepared via GP4. Column chromatography: Hex:EtOAc 39:1 to yield 23.8 mg (85%) of the title compound as a yellowish oil.

^{1}H NMR (400 MHz, $CDCl_3$) δ 7.65 (d, *J* = 16.0 Hz, 1H), 7.62 – 7.47 (m, 2H), 7.37 (dd, *J* = 4.4, 2.1 Hz, 3H), 6.39 (d, *J* = 16.0 Hz, 1H), 5.28 (hept, *J* = 2.9 Hz, 1H), 1.77 (s, 6H), 1.72 (d, *J* = 2.9 Hz, 6H). **^{13}C NMR** (101 MHz, $CDCl_3$) δ 210.3, 165.4, 144.5, 134.6, 130.2, 128.7, 128.1, 119.5, 97.7, 89.5, 78.5, 73.1, 73.1, 29.3, 20.1.

2,7-dimethylocta-5,6-dien-3-yn-2-yl methacrylate (2p)

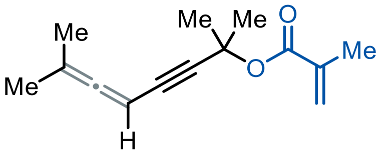

Prepared via GP4. Column chromatography: Hex:EtOAc 29:1 to yield 8.7 mg (40%) of the title compound as a colorless oil.

^{1}H NMR (400 MHz, $CDCl_3$) δ 6.05 (dq, *J* = 2.1, 1.1 Hz, 1H), 5.52 (p, *J* = 1.6 Hz, 1H), 5.26 (hept, *J* = 2.9 Hz, 1H), 1.92 (s, 3H), 1.75 – 1.65 (m, 12H).**^{13}C NMR** (101 MHz, CDCl3) δ 210.3, 165.8, 137.3, 125.2, 97.7, 89.5, 78.4, 73.1, 73.0, 29.2, 20.2, 18.5.

3,8-dimethyldeca-6,7-dien-4-yn-3-yl benzoate (2q)

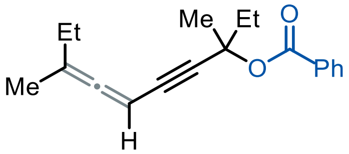

Prepared via GP4. Column chromatography: Hex:EtOAc 49:1 to yield 23.4 mg (dr 1:1, 83%) of the title compound as a colorless oil.

^{1}H NMR (400 MHz, $CDCl_3$) δ 8.08 – 7.97 (m, 2H), 7.60 – 7.49 (m, 1H), 7.48 – 7.37 (m, 2H), 5.37 (h, *J* = 3.1 Hz, 1H), 2.21 – 2.08 (m, 1H), 2.08 – 1.90 (m, 3H), 1.81 (s, 3H), 1.74 – 1.67 (m, 3H), 1.11 (t, *J* = 7.4 Hz, 3H), 1.01 (td, *J* = 7.5, 2.2 Hz, 3H).**^{13}C NMR** (101 MHz, $CDCl_3$) δ 209.8, 164.9, 133.0, 131.4, 129.6, 128.5, 103.8, 88.3, 79.8, 77.1, 74.9, 35.1, 26.9, 26.3, 18.5, 12.1, 8.9.

2,7-diphenylocta-5,6-dien-3-yn-2-yl acetate (2r)

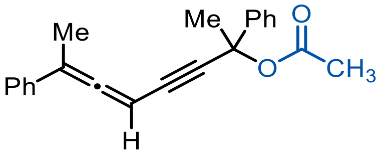

Prepared via GP4. Column chromatography: Hex:EtOAc 29:1 to yield 30.0 mg (dr 1:1, 95%) of the title compound as a colorless oil.

^{1}H NMR (400 MHz, $CDCl_3$) δ 7.58 (dd, *J* = 8.4, 1.3 Hz, 2H), 7.43 (dd, *J* = 8.5, 1.4 Hz, 2H), 7.38 – 7.32 (m, 4H), 7.31 – 7.26 (m, 2H), 5.88 – 5.82 (m, 1H), 2.17 (dd, *J* = 3.1, 1.3 Hz, 3H), 2.08 (s, 3H), 1.91 (s, 3H).**^{13}C NMR** (101 MHz, $CDCl_3$) δ 213.9, 168.8, 142.9, 135.5, 128.6, 128.5, 127.9, 127.5, 126.4, 125.0, 103.3, 89.0, 80.1, 77.3, 76.3, 32.2, 22.0, 16.8.

1-(4-cyclobutylidenebut-3-en-1-yn-1-yl)cyclobutyl benzoate (2s)

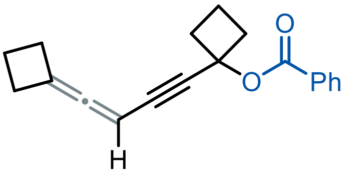

Prepared via GP4. Column chromatography: DCM:Hex 7:3 to yield 8.3 mg (30%) of the title compound as a colorless oil.

^{1}H NMR (400 MHz, CDCl3) δ 8.09 – 7.98 (m, 2H), 7.62 – 7.50 (m, 1H), 7.48 – 7.38 (m, 2H), 5.40 (p, *J* = 4.3 Hz, 1H), 3.04 – 2.90 (m, 2H), 2.93 – 2.80 (m, 2H), 2.72 (ddt, *J* = 10.6, 8.4, 3.2 Hz, 2H), 2.58 (qd, *J* = 9.6, 2.3 Hz, 2H), 2.12 – 1.87 (m, 4H). **^{13}C NMR** (101 MHz, $CDCl_3$) δ 203.7, 164.9, 133.1, 130.6, 129.9, 128.4, 103.1, 89.7, 79.0, 76.8, 73.2, 37.1, 29.7, 17.6, 14.8.

tert-butyl 3-acetoxy-3-(4-(1-(tert-butoxycarbonyl)pyrrolidin-3-ylidene)but-3-en-1-yn-1-yl)pyrrolidine-1-carboxylate (2t)

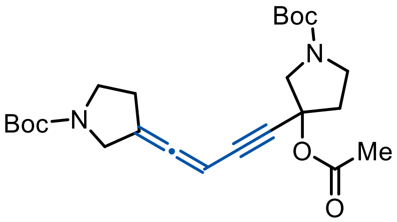

Prepared via GP4. Column chromatography: Hex:EtOAc 4:1 to yield 20.0 mg (45%) of the title compound as a colorless oil as a mixture of rotamers (both given).

^{1}H NMR (400 MHz, $CDCl_3$) δ 5.51 (p, *J* = 3.9 Hz, 1H), 4.10 – 4.05 (m, 1H), 4.01 (s, 1H), 3.85 (dd, *J* = 17.7, 12.3 Hz, 1H), 3.73 (d, *J* = 12.2 Hz, 1H), 3.49 (td, *J* = 22.1, 9.9 Hz, 4H), 2.72 (dd, *J* = 7.6, 4.2 Hz, 1H), 2.67 – 2.57 (m, 1H), 2.49 (dt, *J* = 12.3, 5.5 Hz, 1H), 2.30 (dt, *J* = 13.4, 8.4 Hz, 1H), 2.07 (s, 3H), 1.46 (d, *J* = 2.7 Hz, 18H). **^{13}C NMR** (101 MHz, $CDCl_3$) δ 204.6 (M+m), 169.2 (M+m), 154.2, (m) 154.1 (M), 102.2(M+m), 86.9 (M+m), 79.8 (m), 79.7 (M), 79.2 (M+m), 77.6 (M+m), 56.8 (M+m), 47.7 (M+m), 45.8 (M+m), 45.4 (M+m), 43.9 (M+m), 43.4 (M+m), 38.1 (m), 37.7 (M), 29.6 (M+m), 28.4 (M+m), 21.5 (M+m).

NB: We observed partial product degradation during column chromatography.

1-(4-cyclohexylidenebut-3-en-1-yn-1-yl)cyclohexyl benzoate (2u)

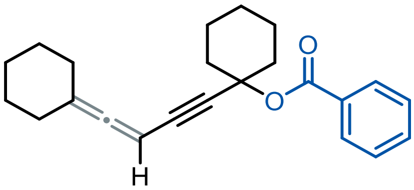

Prepared via GP4. Column chromatography: Hex:EtOAc 39:1 to yield 26.7 mg (80%) of the title compound as a colorless oil.

^{1}H NMR (400 MHz, $CDCl_3$) δ 8.04 (dd, *J* = 8.4, 1.4 Hz, 2H), 7.56 – 7.49 (m, 1H), 7.42 (t, *J* = 7.6 Hz, 2H), 5.30 (p, *J* = 2.2 Hz, 1H), 2.21 – 2.10 (m, 8H), 1.72 – 1.60 (m, 6H), 1.59 – 1.42 (m, 6H). **^{13}C NMR** (101 MHz, $CDCl_3$)

δ 207.1, 164.6, 133.0, 131.4, 129.7, 128.0, 104.3, 88.2, 80.8, 76.5, 73.2, 37.3, 30.8, 26.9, 26.0, 25.4, 22.8.

1-(4-cyclohexylidenebut-3-en-1-yn-1-yl)cyclohexyl 4-fluorobenzoate (2v)

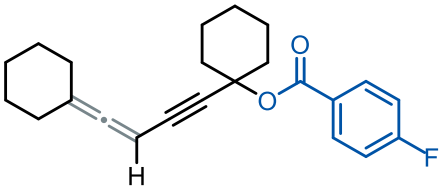

Prepared via GP4. Column chromatography: Hex:EtOAc 39:1 to yield 20.1 mg (60%) of the title compound as a colorless oil.

^{1}H NMR (400 MHz, $CDCl_3$) δ 8.09 – 7.99 (m, 2H), 7.15 – 7.04 (m, 2H), 5.29 (p, *J* = 2.2 Hz, 1H), 2.28 – 2.01 (m, 7H), 1.74 – 1.52 (m, 7H), 1.56 – 1.33 (m, 6H). **^{13}C NMR** (101 MHz, $CDCl_3$) δ 207.1, 165.7 (d, *J* = 253.6 Hz), 163.7, 132.2 (d, *J* = 9.1 Hz), 127.7 (d, *J* = 2.9 Hz), 115.5 (d, *J* = 22.2 Hz), 104.3, 88.0, 76.8, 72.8, 37.4, 30.8, 27.0, 26.0, 25.4, 25.4, 22.7. **^{19}F NMR** (565 MHz, $CDCl_3$) δ -106.4.

1-(4-cyclohexylidenebut-3-en-1-yn-1-yl)cyclohexyl 4-chlorobenzoate (2w)

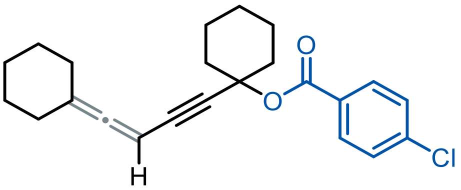

Prepared via GP4. Column chromatography: Hex:EtOAc 39:1 to yield 32.0 mg (87%) of the title compound as a colorless oil.

^{1}H NMR (400 MHz, $CDCl_3$) δ 8.00 – 7.91 (m, 2H), 7.44 – 7.35 (m, 2H), 5.29 (p, *J* = 2.1 Hz, 1H), 2.25 – 1.99 (m, 7H), 1.79 – 1.61 (m, 6H), 1.54 – 1.44 (m, 7H). **^{13}C NMR** (151 MHz, $CDCl_3$*D*) δ 207.2, 163.8, 139.2, 131.2, 129.9, 128.8, 128.7, 104.4, 87.9, 81.1, 74.7, 72.8, 37.4, 30.8, 27.0, 26.0, 25.4, 22.7.

1-(4-cyclohexylidenebut-3-en-1-yn-1-yl)cyclohexyl acetate (2x)

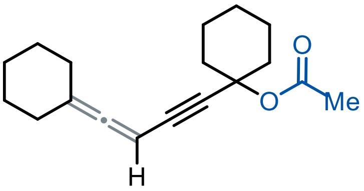

Prepared via GP4. Column chromatography: Hex:EtOAc 29:1 to yield 21.7 mg (80%) of the title compound as a colorless oil.

^{1}H NMR (400 MHz, $CDCl_3$) δ 5.26 (p, *J* = 2.2 Hz, 1H), 2.19 – 2.06 (m, 6H), 2.02 (s, 3H), 1.92 – 1.81 (m, 2H), 1.72 – 1.41 (m, 12H). **^{13}C NMR** (101 MHz, $CDCl_3$) δ 207.0, 169.4, 104.2, 88.1, 80.7, 76.2, 72.8, 37.2, 30.8, 27.0, 26.0, 25.3, 22.7, 22.2.

4-(4-(tetrahydro-4H-pyran-4-ylidene)but-3-en-1-yn-1-yl)tetrahydro-2H-pyran-4-yl acetate (2y)

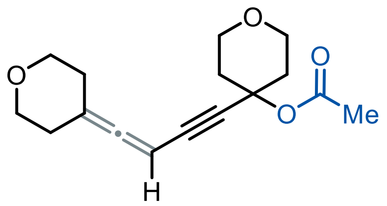

Prepared via GP4. Column chromatography: Hex:EtOAc 4:1 to yield 11.0 mg (40%) of the title compound as a white solid. **mp:** 145.3 – 146.7°C.

^{1}H NMR (400 MHz, $CDCl_3$) δ 5.41 (p, *J* = 2.2 Hz, 1H), 3.93 – 3.78 (m, 4H), 3.77 – 3.65 (m, 4H), 2.44 – 2.19 (m, 6H), 2.08 (s, 3H), 2.07 – 2.00 (m, 2H).**^{13}C NMR** (101 MHz, $CDCl_3$) δ 207.4, 169.3, 100.3, 87.5, 81.0, 74.2, 73.1, 68.3, 64.5, 37.7, 30.8, 22.0.

1-(4-cyclohexylidenebut-3-en-1-yn-1-yl)cyclohexyl cinnamate (2z)

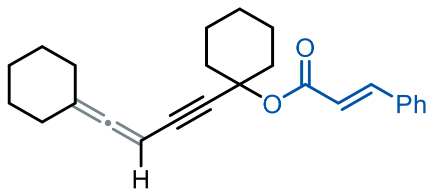

Prepared via GP4. Column chromatography: Hex:EtOAc 49:1 to yield 28.8 mg (80%) of the title compound as a white solid. **mp:** 71.8-73.2 °C.

^{1}H NMR (400 MHz, $CDCl_3$) δ 7.68 (d, *J* = 16.0 Hz, 1H), 7.59 – 7.49 (m, 2H), 7.40 (dd, *J* = 5.0, 1.9 Hz, 3H), 6.44 (d, *J* = 16.0 Hz, 1H), 5.33 (p, *J* = 2.2 Hz, 1H), 2.27 – 2.11 (m, 6H), 2.04 (p, *J* = 6.7 Hz, 2H), 1.71 – 1.43 (m, 12H).**^{13}C NMR** (101 MHz, $CDCl_3$) δ 207.1, 165.2, 144.4, 134.7, 130.2, 129.0, 128.2, 119.5, 104.3, 88.2, 80.9, 76.4, 72.9, 37.5, 30.8, 27.0, 26.0, 25.4, 22.7.

2-(4-(adamantan-2-ylidene)but-3-en-1-yn-1-yl)adamantan-2-yl acetate (2aa)

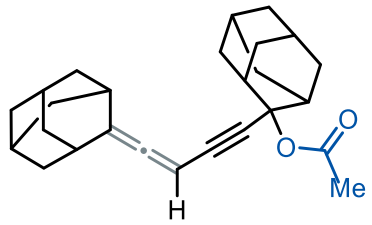

Prepared via GP4. Column chromatography: Hex:EtOAc 29:1 to yield 18.0 mg (48%) of the title compound as a white solid. **mp:** 84.3-86.7 °C.

^{1}H NMR (400 MHz, $CDCl_3$) δ 5.27 (s, 1H), 2.55 (t, *J* = 3.2 Hz, 2H), 2.45 (t, *J* = 3.1 Hz, 2H), 2.24 – 2.13 (m, 2H), 2.06 (s, 3H), 2.01 – 1.66 (m, 20H), 1.64 – 1.55 (m, 2H).**^{13}C NMR** (101 MHz, $CDCl_3$) δ 203.4, 169.3, 111.4, 88.0, 82.3, 80.7, 73.4, 38.7, 38.3, 37.7, 37.0, 36.3, 34.9, 34.3, 32.2, 31.7, 28.0, 27.9, 27.0, 26.5, 22.8, 22.0, 14.2.

2,6,11,15-tetramethylhexadeca-2,9,10,14-tetraen-7-yn-6-yl acetate (2ab)

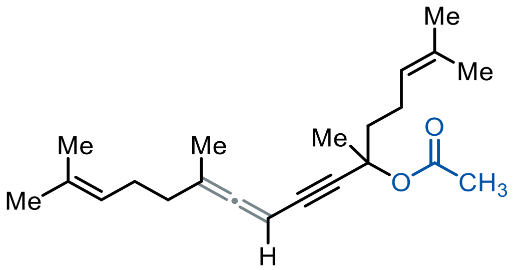

Prepared via GP4. Column chromatography: Hex:EtOAc 30:1 to yield 16.4 mg (dr 1:1, 50%) of the title compound as a colorless oil.

^{1}H NMR (400 MHz, $CDCl_3$) δ 5.31 (hept, *J* = 3.3 Hz, 1H), 5.11 (dtt, *J* = 8.4, 5.4, 1.4 Hz, 2H), 2.20 – 2.12 (m, 2H), 2.08 (t, *J* = 7.3 Hz, 2H), 2.00 (s, 3H), 1.95 (dd, *J* = 9.3, 7.4 Hz, 3H), 1.86 – 1.74 (m, 1H), 1.70 (d, *J* = 2.9 Hz, 3H), 1.67 (s, 9H), 1.60 (d, *J* = 5.8 Hz, 6H).**^{13}C NMR** (101 MHz, $CDCl_3$) δ 210.1, 169.5, 132.1, 132.1, 123.8, 123.6, 101.8, 88.6, 79.5, 76.1, 74.5, 41.7, 33.8, 26.6, 26.0, 25.8, 25.8, 23.2, 22.1, 18.6, 17.8, 17.6.

(3R,5S,7R,8R,9S,10S,12S,13R,14S,17R)-10,13-dimethyl-17-((R)-5-((2,7-dimethylocta-5,6-dien-3-yn-2-yl)oxy)-5-oxopentan-2-yl)hexadecahydro-1H-cyclopenta[a]phenanthrene-3,7,12-triyl triacetate (2ac)

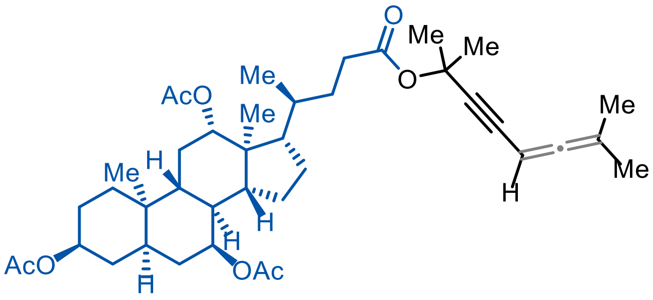

Prepared via GP4. Column chromatography: Hex:EtOAc 4:1 to yield 19.9 mg (30%) of the title compound as a white solid. **dec.**: 256.1°C.

^{1}H NMR (400 MHz, $CDCl_3$) δ 5.26 (hept, *J* = 3.0 Hz, 1H), 5.13 – 5.07 (m, 1H), 4.92 (q, *J* = 3.0 Hz, 1H), 4.59 (tt, *J* = 11.3, 4.3 Hz, 1H), 2.30 (ddt, *J* = 15.3, 10.8, 5.5 Hz, 1H), 2.19 – 2.15 (m, 4H), 2.07 – 2.02 (m, 7H), 2.03 – 1.76 (m, 7H), 1.73 (d, *J* = 2.9 Hz, 6H), 1.68 (s, 6H), 1.65 – 1.57 (m, 5H), 1.51 (td, *J* = 7.6, 2.8 Hz, 2H), 1.41 (ddt, *J* = 13.3, 7.1, 3.1 Hz, 2H), 1.35 – 1.21 (m, 3H), 1.10 (dddd, *J* = 17.5, 14.4, 10.7, 4.6 Hz, 2H), 0.93 (s, 3H), 0.82 (d, *J* = 6.5 Hz, 3H), 0.74 (s, 3H).**^{13}C NMR** (101 MHz, $CDCl_3$) δ 210.2, 172.5, 170.7 (2C), 170.5, 97.7, 89.5, 78.3, 75.6, 74.2, 73.0, 72.8, 70.8, 47.5, 45.2, 43.5, 41.1, 37.9, 34.8, 34.7, 34.6, 34.5, 32.0, 31.4, 30.8, 29.2, 29.2, 29.0, 27.3, 27.0, 25.7, 22.9, 22.7, 21.7, 21.6, 21.6, 20.1 (2C), 17.7, 12.3.

Products **3-5**

2,7-dimethylocta-5,6-dien-3-yn-2-ol (3)

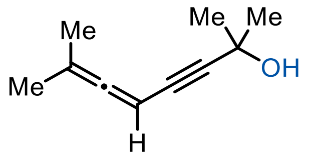

Prepared according to a procedure described above. The crude product yielded 27 mg (90%) of the title compound as a colorless oil.

^{1}H NMR (400 MHz, $CDCl_3$) δ 5.22 (p, *J* = 2.9 Hz, 1H), 1.73 (d, *J* = 3.0 Hz, 6H), 1.53 (s, 6H). **^{13}C NMR** (101 MHz, $CDCl_3$) δ 210.2, 97.7, 93.2, 76.4, 72.9, 65.7, 31.5, 20.0. Spectroscopic data are in accordance with the literature.[10]

3,3-dimethyl-4-(prop-1-en-2-yl)cyclopenta-1,4-dien-1-yl 4-nitrobenzoate (4)

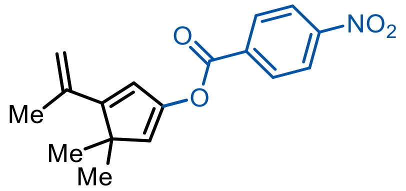

Prepared according to a procedure described above. The crude product was purified *via* column chromatography (Hex:EtOAc, 60:1) to yield 38.8 mg (65%) of the title compound as a yellowish solid. **mp:** 104.0- 105.1 °C.

^{1}H NMR (600 MHz, $CDCl_3$) δ 8.36 – 8.28 (m, 4H), 6.32 (d, *J* = 1.8 Hz, 1H), 6.19 (d, *J* = 1.8 Hz, 1H), 5.11 (s, 1H), 5.05 (s, 1H), 2.01 (d, *J* = 1.3 Hz, 3H), 1.33 (s, 6H). **^{13}C NMR** (101 MHz, $CDCl_3$) δ 162.4, 155.1, 150.9, 147.5, 136.6, 135.2, 131.2, 130.6, 123.8, 123.7, 114.2, 49.9, 23.9, 22.6.

4-oxo-3,5-di(propan-2-ylidene)cyclopent-1-en-1-yl benzoate (5)

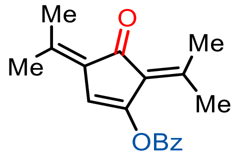

Prepared according to a procedure described above. The crude product was purified *via* column chromatography (Hex:EtOAc, 60:1) to yield 37.7 mg (67%) of the title compound as a colorless oil.

^{1}H NMR (400 MHz, $CDCl_3$) δ 8.13 (dd, *J* = 8.4, 1.4 Hz, 2H), 7.68 – 7.59 (m, 1H), 7.50 (dd, *J* = 8.5, 7.1 Hz, 2H), 6.62 (s, 1H), 2.36 (s, 3H), 2.33 (s, 3H), 2.09 (s, 3H), 1.93 (s, 3H).**^{13}C NMR** (101 MHz, $CDCl_3$) δ 191.5, 164.3, 149.8, 146.2, 142.7, 133.9, 130.8, 130.2, 129.3, 128.9, 127.7, 116.6, 23.7, 23.2, 22.5, 20.9.

Elemental analysis of unknown compounds

	Calculated				*Found*			
Compound	C	H	N	S	C	H	N	S
2a	80.28	7.13			79.95	6.99		
2b	74.98	6.29			74.66	6.18		
2c	70.71	5.93			70.10	6.16		
2d	61.28	5.14			61.50	4.95		
2e	80.56	7.51			80.36	7.41		
2f	68.22	5.72	4.68		68.44	5.80	4.59	
2g	80.56	7.51			80.87	7.72		
2h	73.75	6.6			72.80	6.58		
2i	69.2	6.19		12.31	69.15	5.94		12.18
2j	75.27	6.71	5.49		75.44	6.70	5.61	
2k	74.97	8.39			75.28	8.65		
2l	73.33	9.15			73.07	8.98		
2m	77.03	8.31			77.11	8.10		
2n	77.03	8.31			76.71	8.40		
2o	81.4	7.19			81.10	7.05		
2p	77.03	8.31			76.77	8.44		
2q	80.82	7.85			80.82	7.62		
2r	83.52	6.37			83.32	6.62		
2s	81.99	6.52			81.60	6.32		
2t	64.55	7.67	6.27		64.32	7.47	6.32	
2u	82.6	7.84			82.77	8.07		
2v	78.38	7.15			78.48	7.10		
2w	74.89	6.83			74.69	6.78		
2x	79.37	8.88			79.18	9.13		
2y	69.55	7.3			79.39	7.62		
2z	83.29	7.83			82.89	8.11		
2aa	82.94	8.57			82.91	8.49		
2ab	80.44	9.82			80.31	9.61		
2ac	72.04	8.77			72.09	8.84		
4	68.22	5.72	4.68		68.32	5.89	4.53	
5	76.57	6.43			76.74	6.32		

7. Mechanistic investigation

Use of zero-valent copper in solution

A 15 mL Schlenk tube was charged with copper powder (5.1 mg, 0.08 mmol, 0.4 equiv.), CTAB (72.8 mg, 0.2 mmol, 1 equiv.), DIPEA (70 µL, 0.4 mmol, 2 equiv.), substrate **1a** (37.6 mg, 0.2 mmol, 1 equiv.), and CH_3CN (2.0 mL, 0.1 M). The reaction mixture was stirred for the specified time and temperature. The mixture was quenched by dilution with EtOAc and filtered through a short silica plug to remove solids. The solvent was removed under reduced pressure, and the crude product was analyzed by ^1H NMR spectroscopy using CH_2Br_2 as an internal standard.

Where specified, copper was activated either chemically (etching by HNO_3) or mechanically (ball milling):

Procedure for chemical activation: a 5 mL vial was charged with 1 g of copper powder and 3 mL of HNO_3 0.1 N. The suspension was sonicated for 15 minutes. The copper was recovered via filtration over Buchner, rinsed with water, acetone, and diethyl ether. The so-obtained copper was directly used and stored in a Schlenk flask under argon atmosphere.

Procedure for mechanochemical activation: a stainless-steel jar was loaded with 500 mg of copper powder and a stainless-steel ball. The solid was milled at 30 Hz for 90 minutes. Note: sintering of the metal was macroscopically observed.

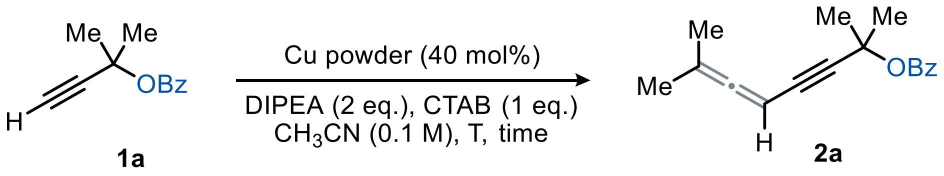

Table S8 Control experiments in solution with zero-valent copper.

Entry	*Time (h)*	*Temperature (° C)*	*Yield (%)*[a]
1	2	21	n.d.
2	24	21	n.d.
3	2	82	n.d.
4	24	82	n.d.
5[b]	24	21	n.d.
6[b]	24	82	traces
7	24	21	n.d.
8[c]	24	82	13[d]

[a] ^1H-NMR yields calculated using CH_2Br_2 as internal standard. [b] Chemically activated copper was used. [c] Mechanically activated copper was used. [d] major decomposition was observed.

The performed experiments showed that off-the-shelf copper powder is not able to promote the observed reactivity in solution, neither at room temperature nor in refluxing CH_3CN. Even upon prolonged reaction time (24 h), the expected product (**2a**) was not observed. When fresh surface of Cu(0) was exposed via chemical or mechanochemical activation, **2a** was not observed either. It should be noted that when the reaction was performed at high temperature (refluxing CH_3CN), major decomposition was observed in the case of mechanically activated copper.

NB: Following each use, stainless steel jars were rigorously cleaned with a 30% ammonium hydroxide solution to ensure complete removal of copper traces.

Studies on the mechanochemical degradation of **2a**

Reactions were performed following GP4. Thus, copper powder (5,1 mg, 0.08 mmol, 0.4 eq.), CTAB (72.8 mg, 0.2 mmol, 1 eq.), CH_3CN (η=1), DIPEA (70 µL, 0.4 mmol, 2 eq.) and product **2a** (50.8 mg, 0.2 mmol, 1 eq.) were added in a 5 mL stainless-steel jar equipped with one 10 mm hardened stainless-steel ball. The mixture was milled for the specified time and the crude was processed and analyzed as described above (^1H NMR, CH_2Br_2 as internal standard).

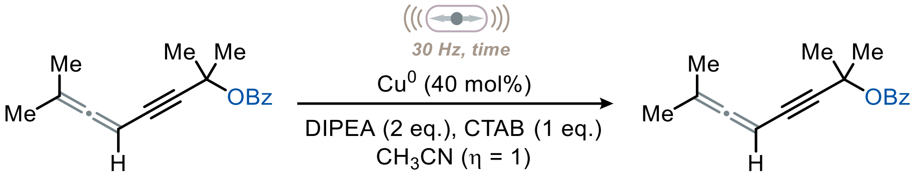

2a

Table S9. Study of the mechanochemical degradation of product ***2a***

Entry	Time (h)	Recovery (%)[a]
1	2	85
2	4	54
3[b]	4	95

[a] ^1H-NMR recoveries calculated using CH_2Br_2 as internal standard. [b] Reaction performed without copper.

These experiments show that **2a** is not stable under reaction conditions and well explain why a decrease in yield is recorded upon prolonged milling (> 2 h).

NB: Following each use, stainless steel jars were rigorously cleaned with a 30% ammonium hydroxide solution to ensure complete removal of copper traces.

Influence of O_2 on the mechanochemical process

The Fritsch instrument displayed in Figure S2 was used. Two experiments were performed: one under optimized conditions (Table S5, entry 11) and one under argon atmosphere to exclude molecular oxygen from the jar. Reaction mixtures were performed following GP4: copper powder (5,1 mg, 0.08 mmol, 0.4 eq.), CTAB (72.8 mg, 0.2 mmol, 1 eq.), CH_3CN (η=1), DIPEA (70 µL, 0.4 mmol, 2 eq.) and substrate **1a** (37.6 mg, 0.2 mmol, 1 eq.) were added in a 15 mL stainless-steel jar with one 10 mm hardened stainless-steel ball. For the test performed under argon, the inert gas was flowed into the jar for 10 minutes, and then the jar was sealed while the flow was still ongoing. The sealed vessel was mounted into the holding station of a P23 mixer mill. The mixture was milled for the specified time and the crude was processed and analyzed as described above (^1H NMR, CH_2Br_2 as internal standard).

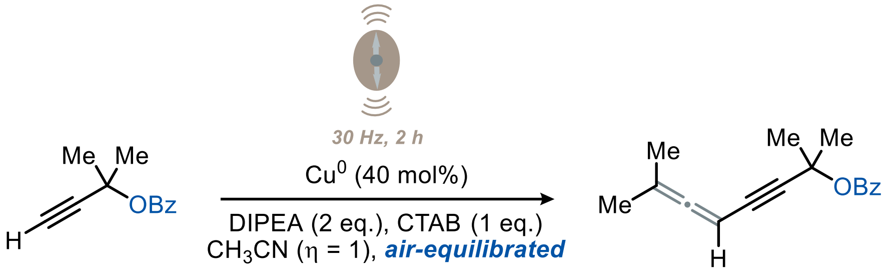

1a **2a**, 50%

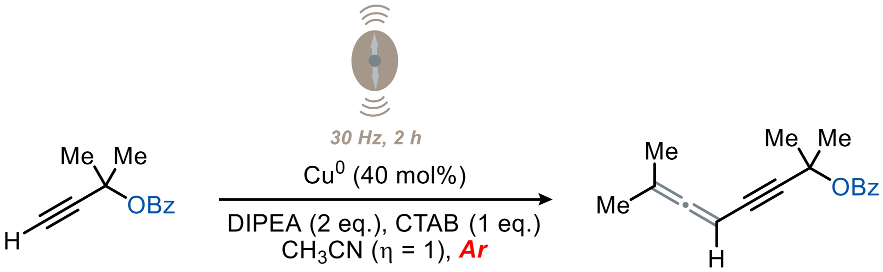

1a **2a,** traces

These experiments show that the presence of O_2 significantly boosts catalysis, suggesting the role of oxygen as a sacrificial oxidant. We attribute the lower yield obtained with the Fritsch pulverisette P23 vertical mill compared to the MM400 to the different volume of the jar and, thus, the different kinetic energy in the system.

NB: Following each use, stainless steel jars were rigorously cleaned with a 30% ammonium hydroxide solution to ensure complete removal of copper traces.

Influence of CuBr percentages on the mechanochemical process

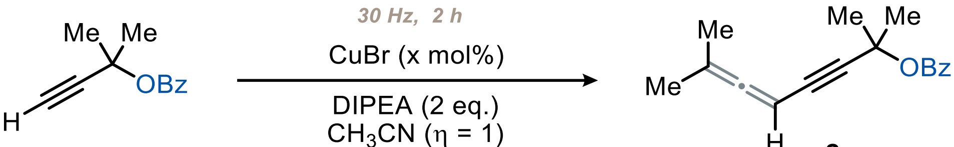

1a **2a**

Entry	*% CuBr*	*Yield (%)*[a]
1	5	74
2	20	45
3[b]	40	30

[a] ¹H-NMR recoveries calculated using CH_2Br_2 as internal standard. [b] Reaction already present in the SI (page S35).

Kinetic experiments

Kinetic profile was obtained by monitoring the model reaction for the conversion of propargyl ester**1a** to **2a** at different milling time. GP4 was followed. The reaction was stopped at the indicated time, the crude was processed and analyzed as described above (^{1}H NMR, CH_2Br_2 as internal standard).

NB: Following each use, stainless steel jars were rigorously cleaned with a 30% ammonium hydroxide solution to ensure complete removal of copper traces.

Kinetic profile under optimized conditions

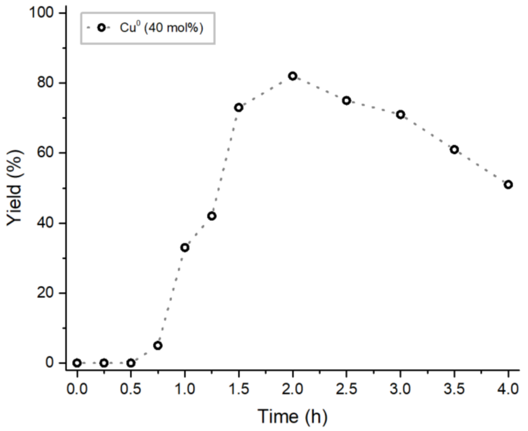

Entry	Milling time (h)	Yield (%)
**1**	0	n.d.
**2**	0.25	n.d.
**3**	0.5	traces
**4**	0.75	5
**5**	1	33
**6**	1.25	42
**7**	1.5	73
**8**	2	82
**9**	2.5	75
**10**	3	71
**11**	3.5	61
**12**	4	51

Kinetic profile with CuBr (40 mol%)

In this case, copper powder was replaced with CuBr. Two kinetic profiles were recorded, with and without texture agent (CTAB).

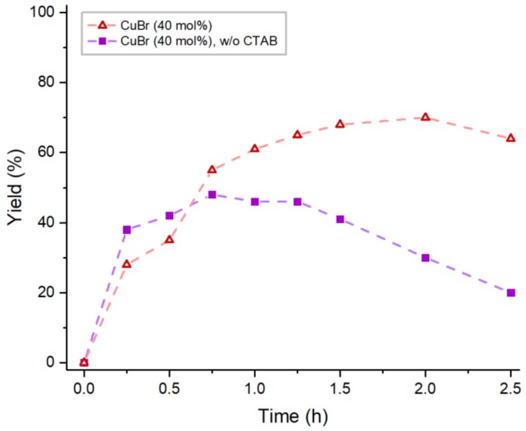

Entry	Milling time (h)	Yield, w/o CTAB	Yield, w/ CTAB
**1**	0	0	n.d.
**2**	0.25	38	28
**3**	0.5	42	35
**4**	0.75	48	55
**5**	1	46	61
**6**	1.25	46	65
**7**	1.5	41	68
**8**	2	30	70
**9**	2.5	20	64

Kinetic profile with pre-milled copper

*In this experiment, copper was pre-milled with DIPEA, CTAB and CH_3CN (η = 1) for 90 minutes at 30 Hz. Then, **1a** was added and the mixture was monitored in time.*

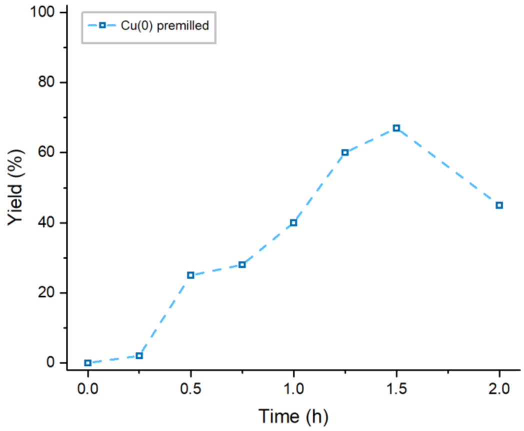

Entry	Milling time (h)	Yield (%)
**1**	0	n.d.
**2**	0.25	traces
**3**	0.5	25
**4**	0.75	28
**5**	1	40
**6**	1.25	60
**7**	1.5	67
**8**	2	52

8. References

[1] T. R. Pradhan, D. K. Mohapatra, *Adv. Synth. Catal.* **2019**, *361*, 3605-3611.

[2] V. V. Pagar, A. M. Jadhav, R.-S. Liu, *J. Am. Chem. Soc.* **2011**, *133*, 20728-20731.

[3] M. C. Nakhla, J. L. Wood, *J. Am. Chem. Soc.* **2017**, *139*, 18504-18507.

[4] L. C. Wilkins, Y. Soltani, J. R. Lawson, B. Slater, R. L. Melen, *Chem. Eur. J.* **2018**, *24*, 7364-7368.

[5] G. F. Hennion, S. O. Barrett, *J. Am. Chem. Soc.* **1957**, *79*, 2146-2148.

[6] B. C. Söderberg, S. N. O'Neil, A. C. Chisnell, J. Liu, *Tetrahedron* **2000**, *56*, 5037-5044.

[7] M.-B. Li, D. Posevins, A. Geoffroy, C. Zhu, J.-E. Bäckvall, *Angew. Chem. Int. Ed.* **2020**, *59*, 1992-1996.

[8] E. A. Dikusar, N. G. Kozlov, S. S. Koval'skaya, L. A. Popova, K. L. Moiseichuk, *Russ. J. Gen. Chem.* **2001**, *71*, 290-293.

[9] M. Gaydou, R. E. Miller, N. Delpont, J. Ceccon, A. M. Echavarren, *Angew. Chem. Int. Ed.* **2013**, *52*, 6396-6399.

[10] H. Jiang, W. Wang, B. Yin, W. Liu, *Eur. J. Org. Chem.* **2010**, *2010*, 4450-4453.

9. Copies of NMR spectra

Substrates **1a-ac**

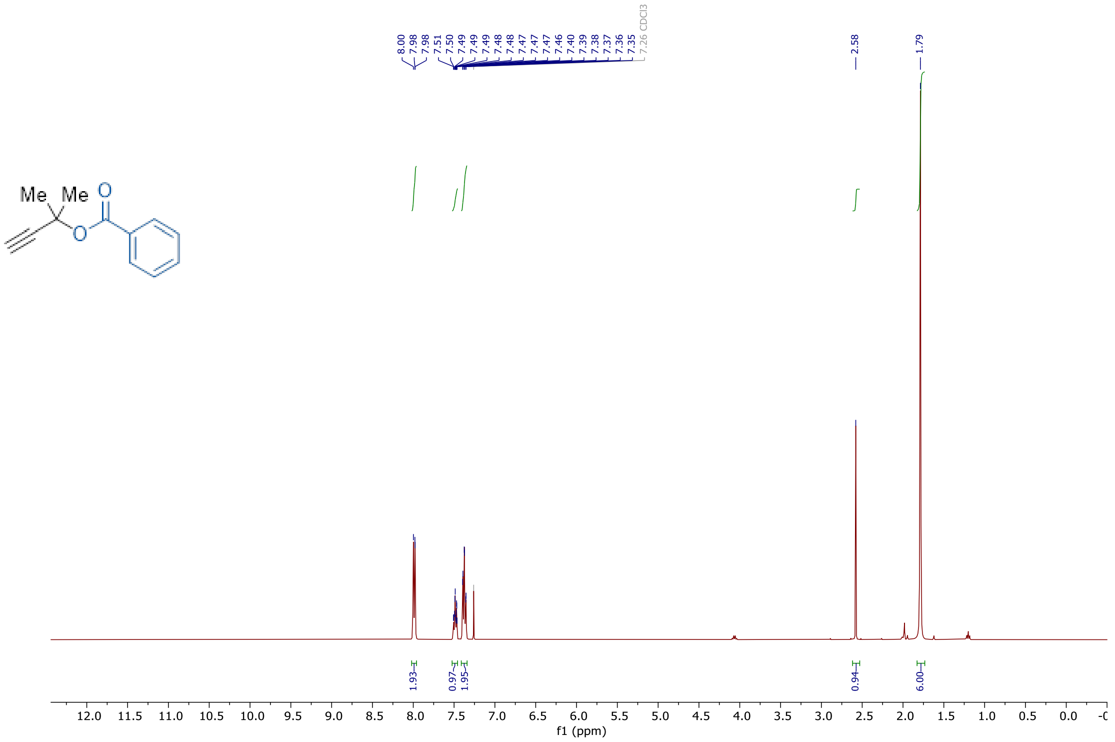

^{1}H NMR of compound **1a** (400 MHz, $CDCl_3$)

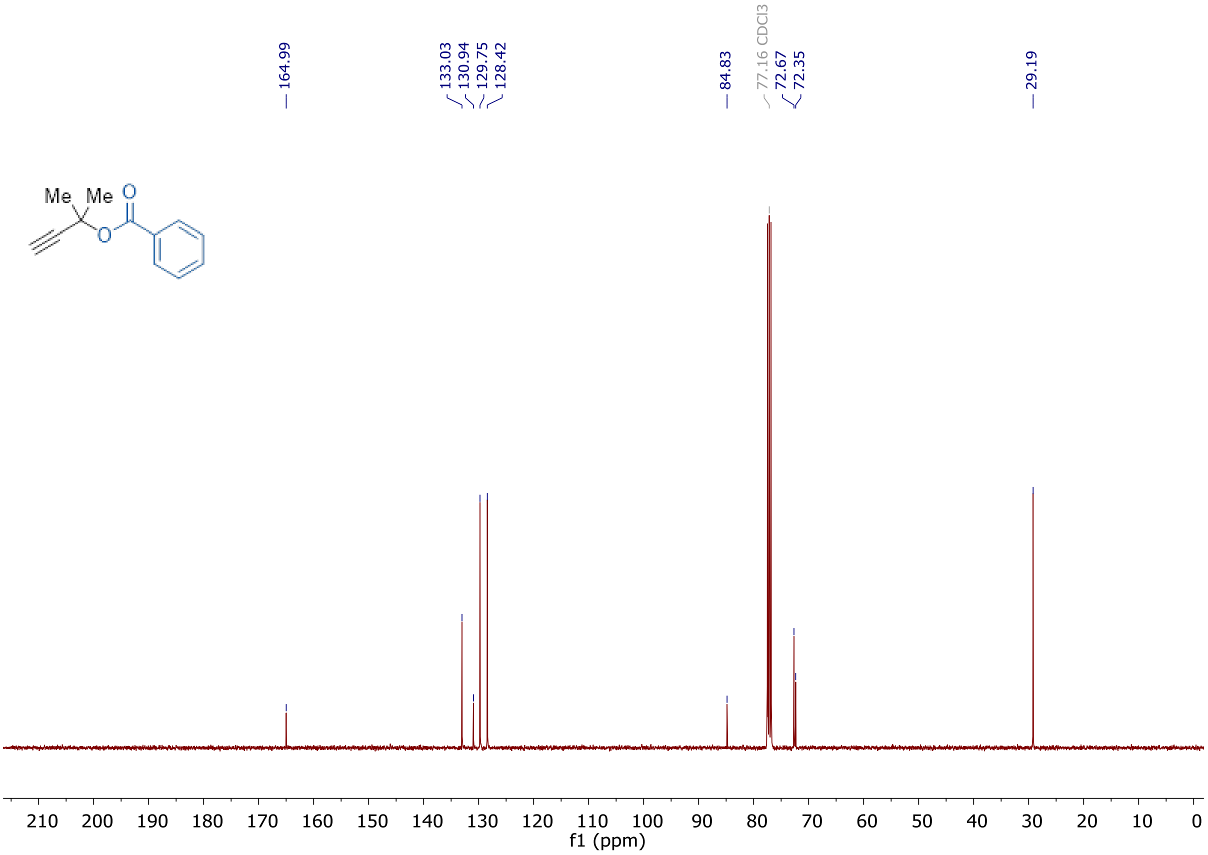

^{13}C NMR of compound **1a** (101 MHz, $CDCl_3$)

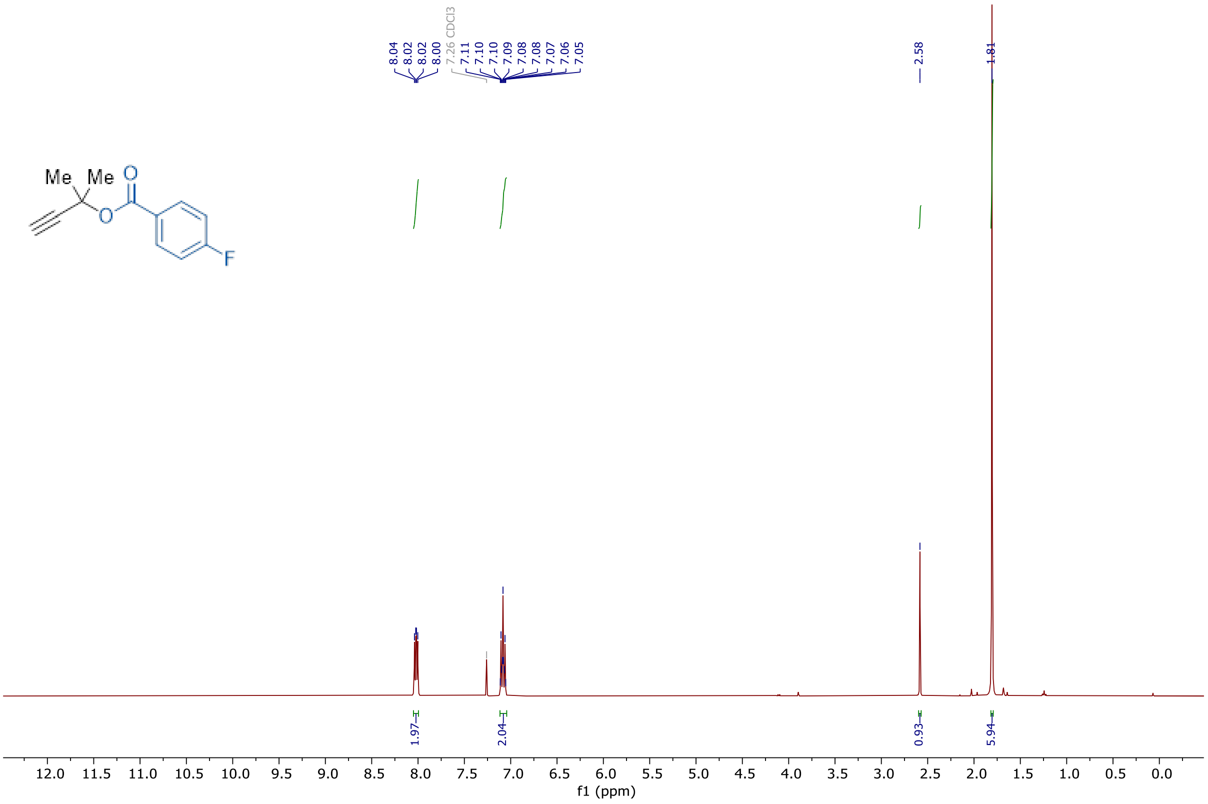

^{1}**H NMR** of compound **1b** (400 MHz, $CDCl_3$)

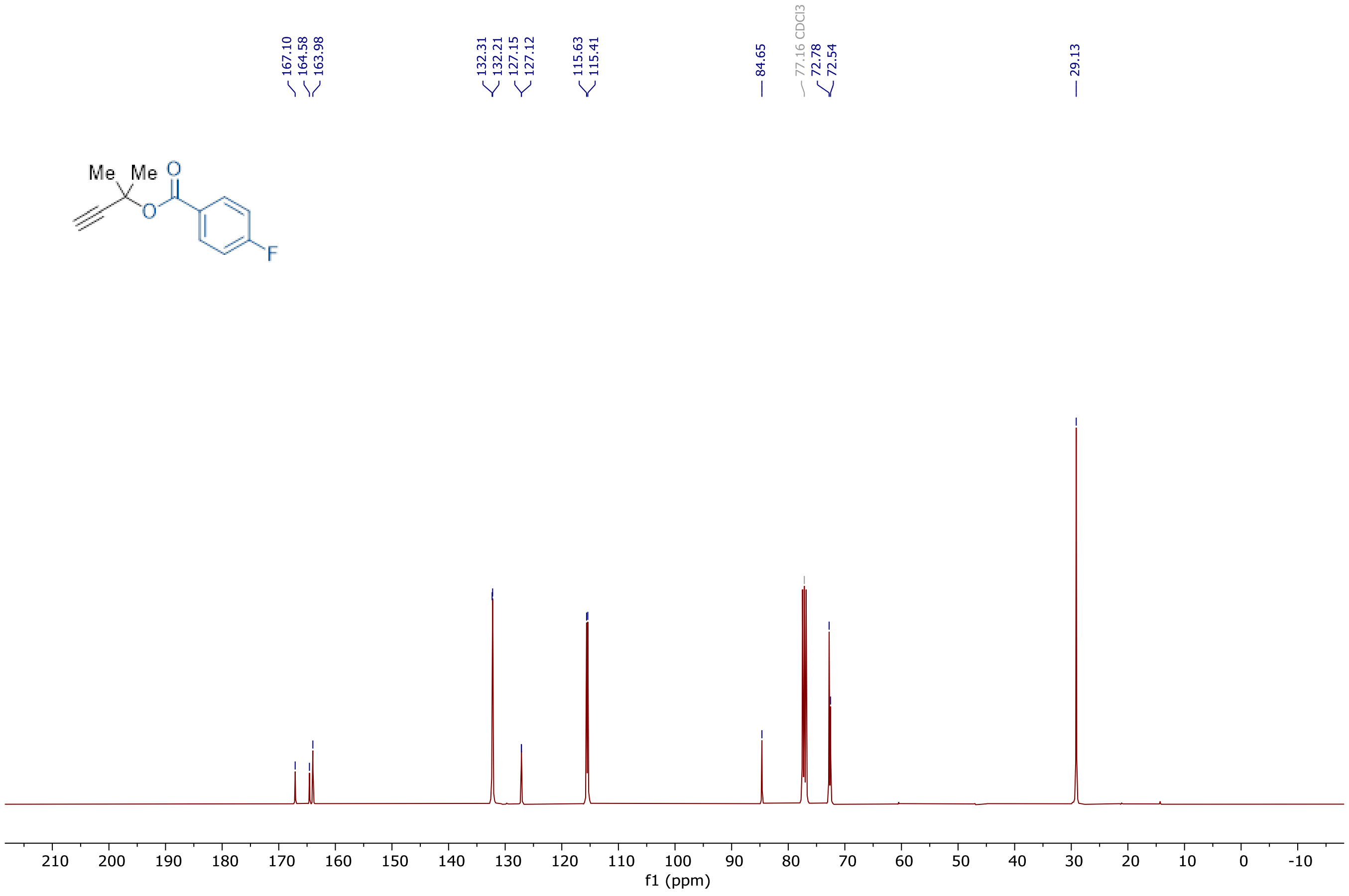

^{13}**C NMR** of compound **1b** (101 MHz, $CDCl_3$)

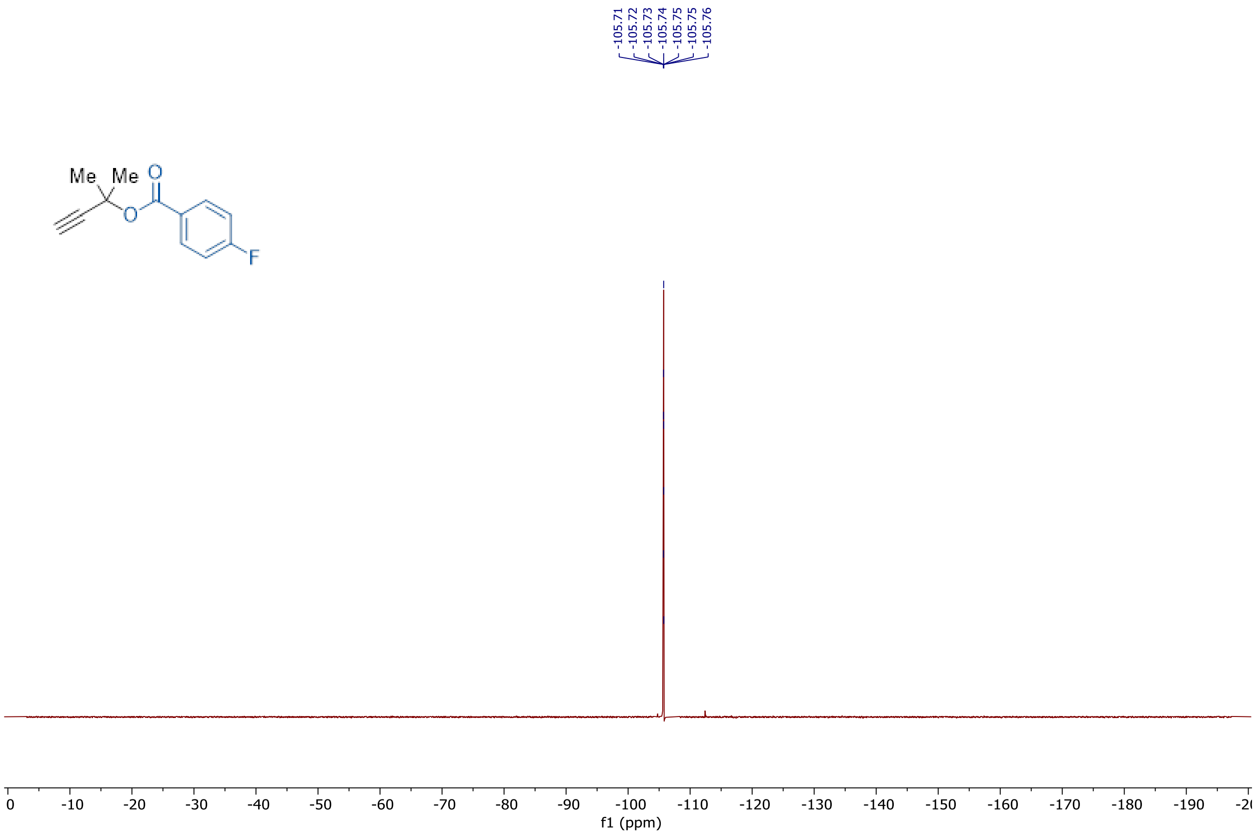

[19]**F NMR** of compound **1b** (565 MHz, $CDCl_3$)

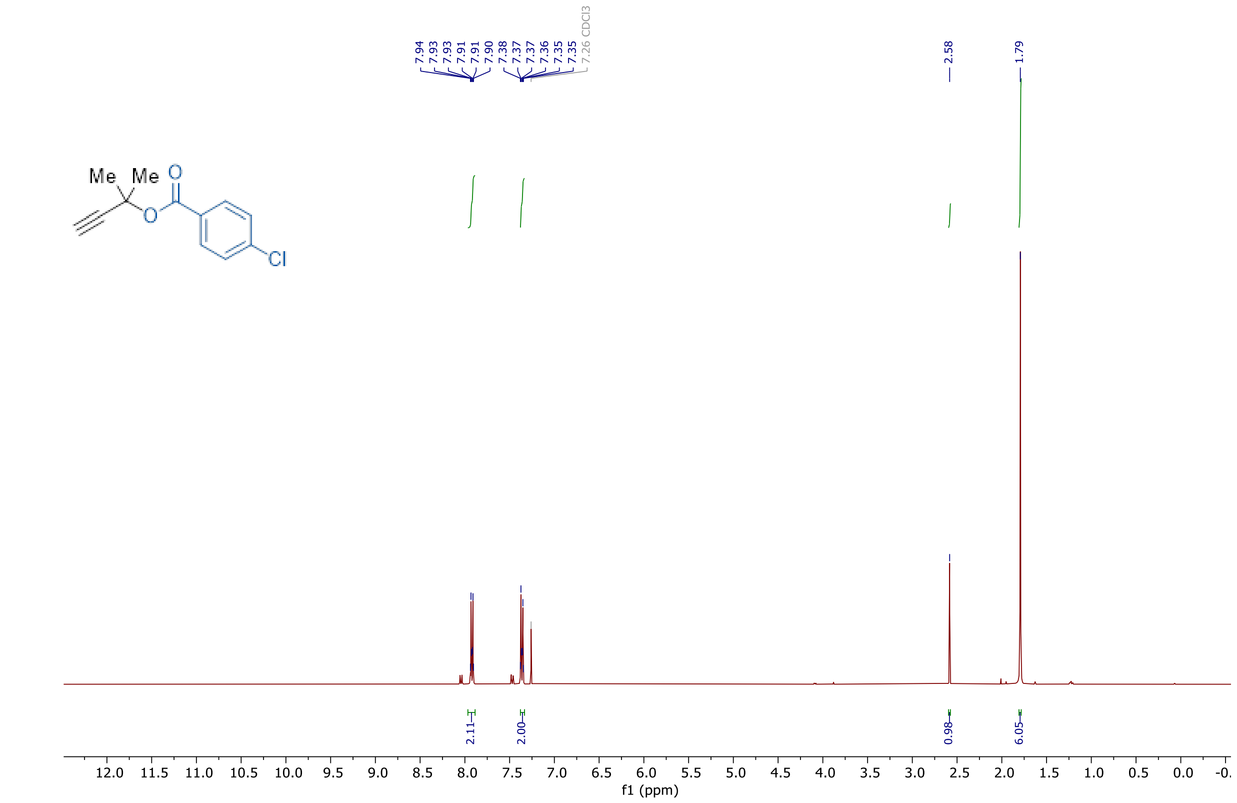

¹H NMR of compound **1c** (400 MHz, $CDCl_3$)

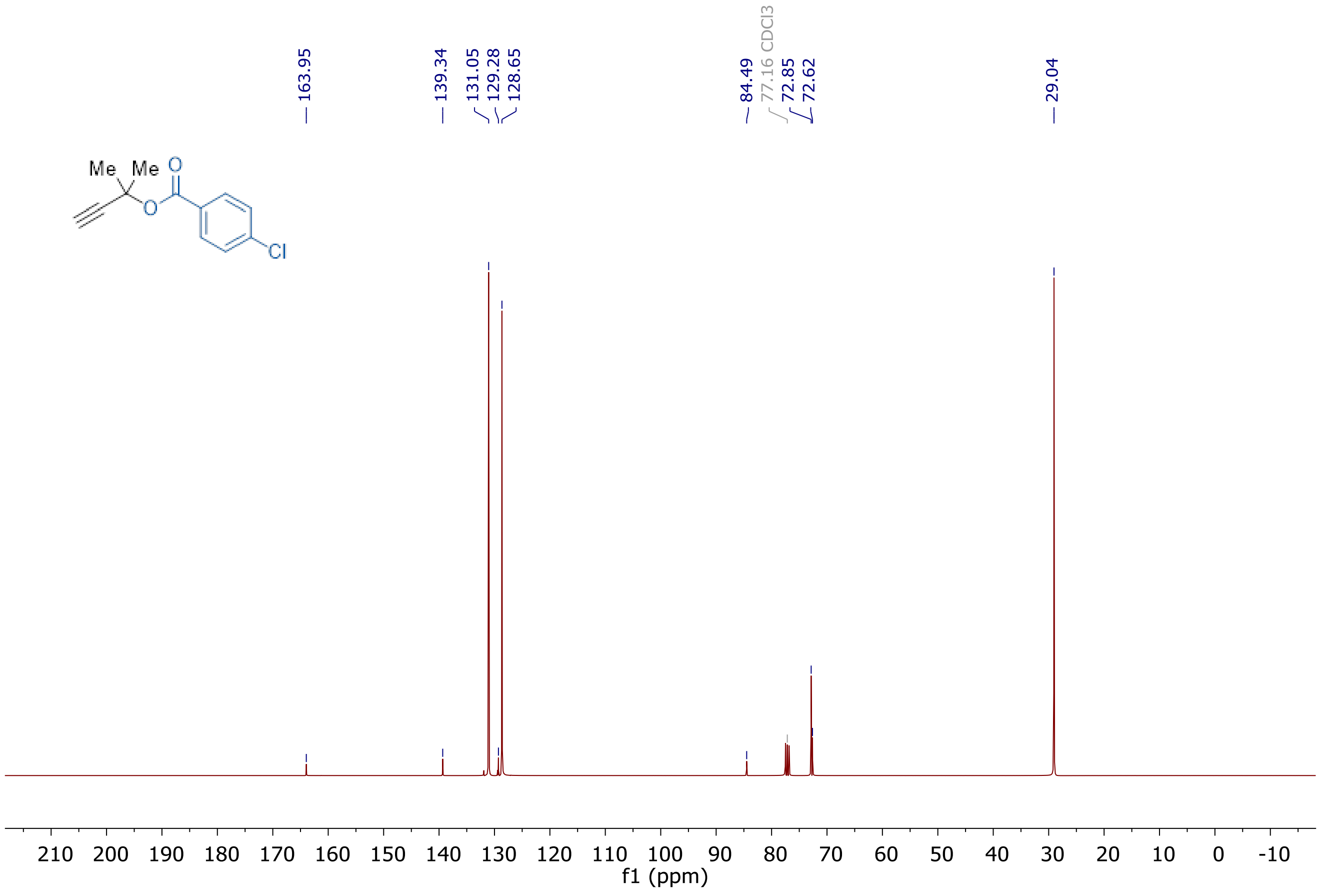

¹³C NMR of compound **1c** (101 MHz, $CDCl_3$)

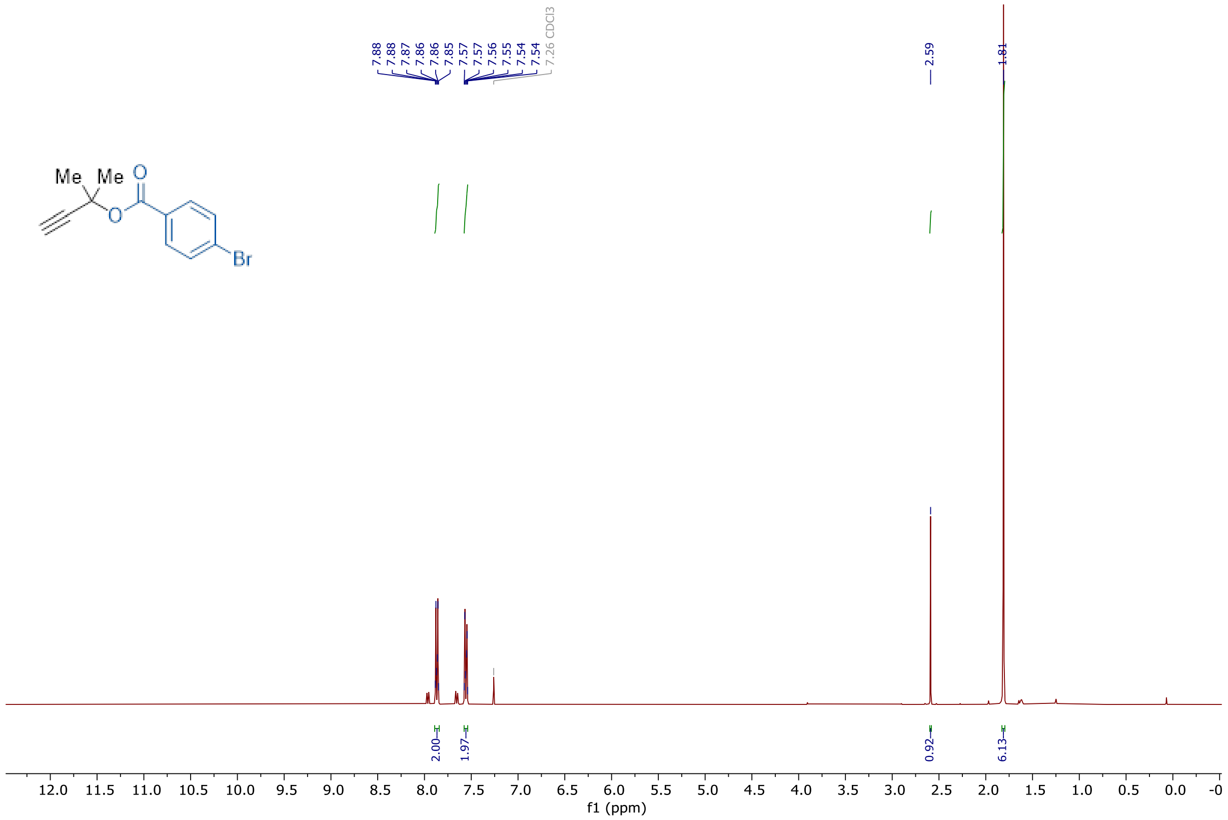

^{1}H NMR of compound **1d** (400 MHz, $CDCl_3$)

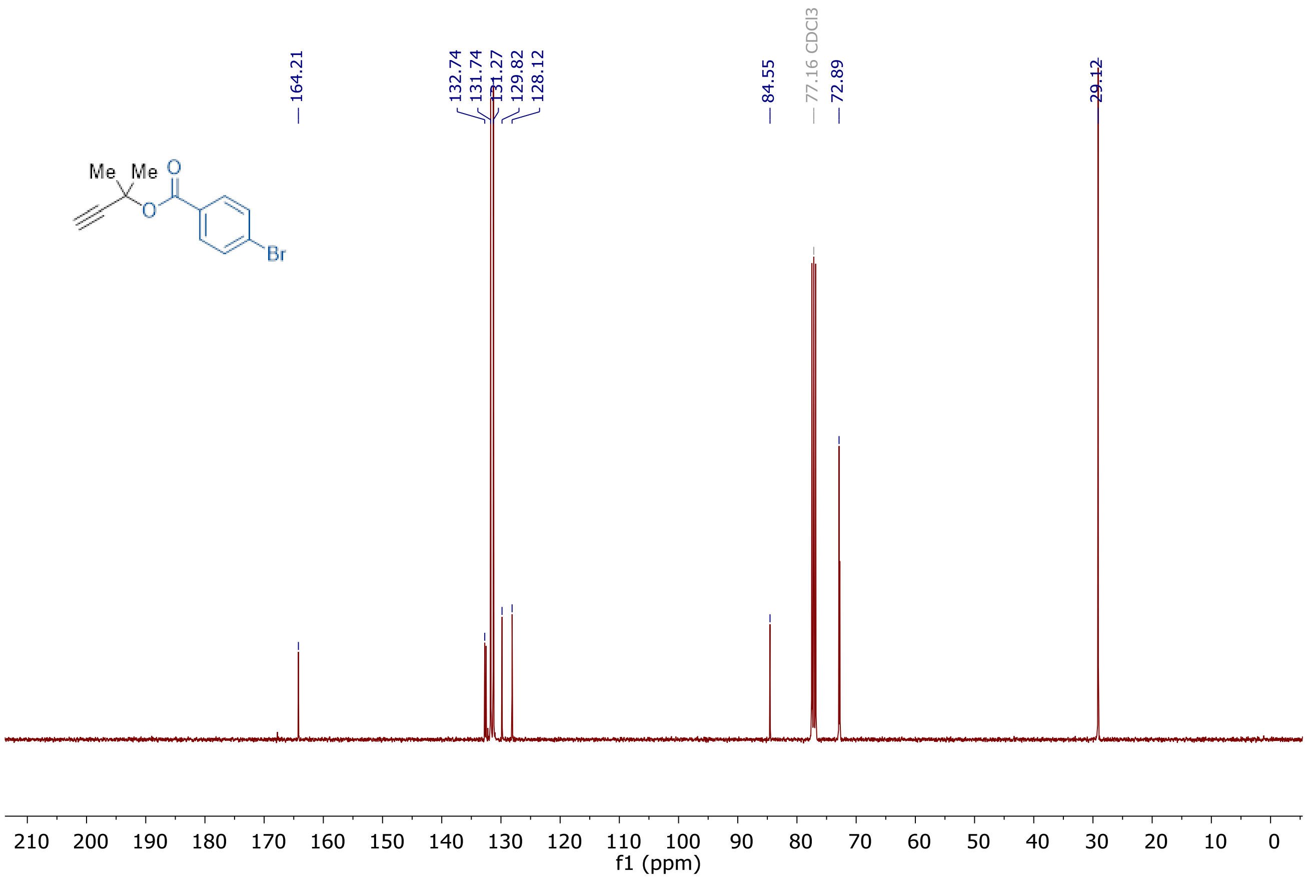

^{13}C NMR of compound **1d** (101 MHz, $CDCl_3$)

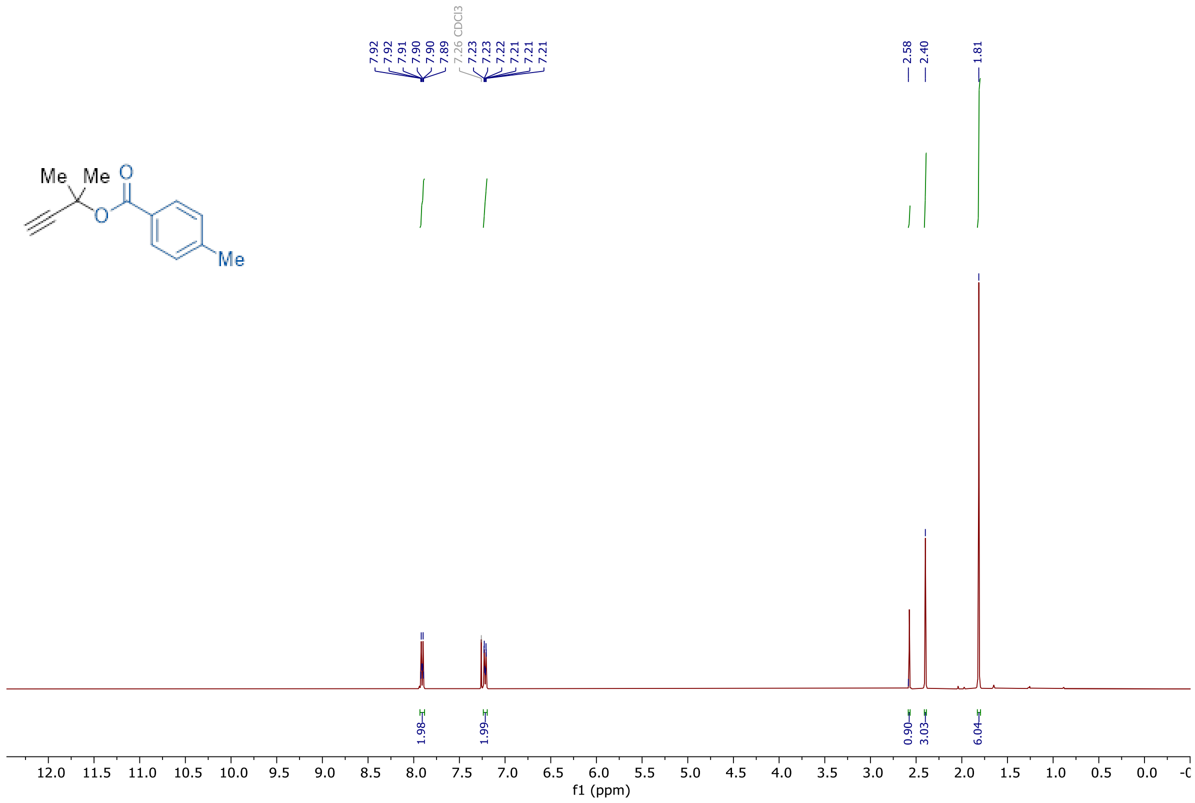

[1]**H NMR** of compound **1e** (400 MHz, $CDCl_3$)

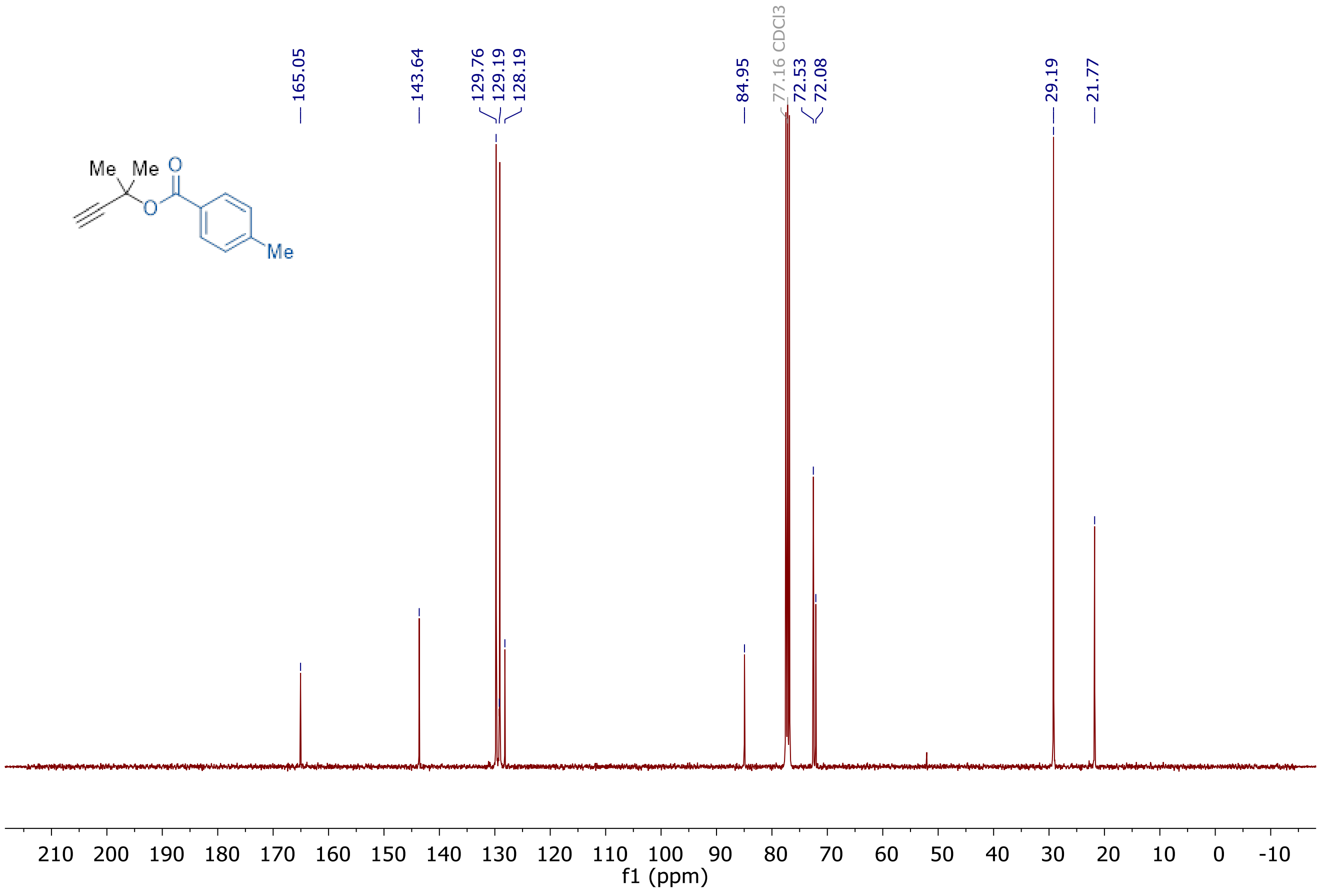

[13]**C NMR** of compound **1e** (101 MHz, $CDCl_3$)

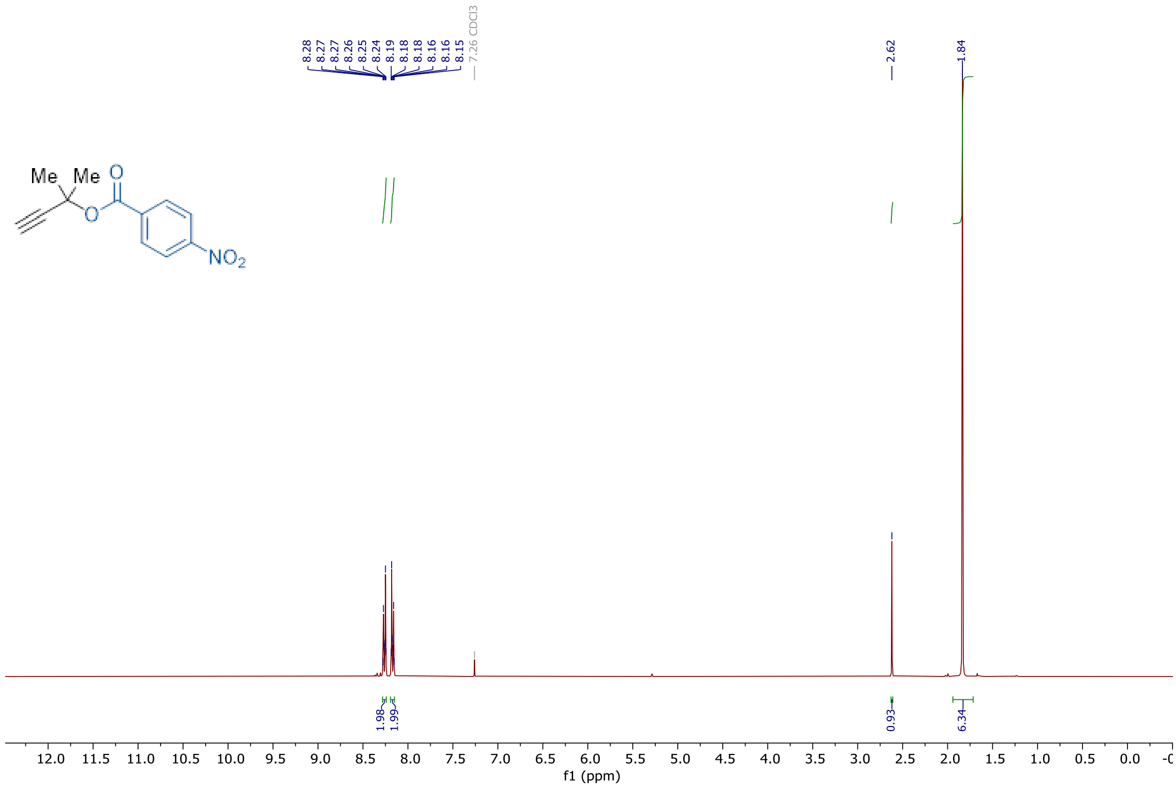

^{1}H NMR of compound 1f (400 MHz, $CDCl_3$)

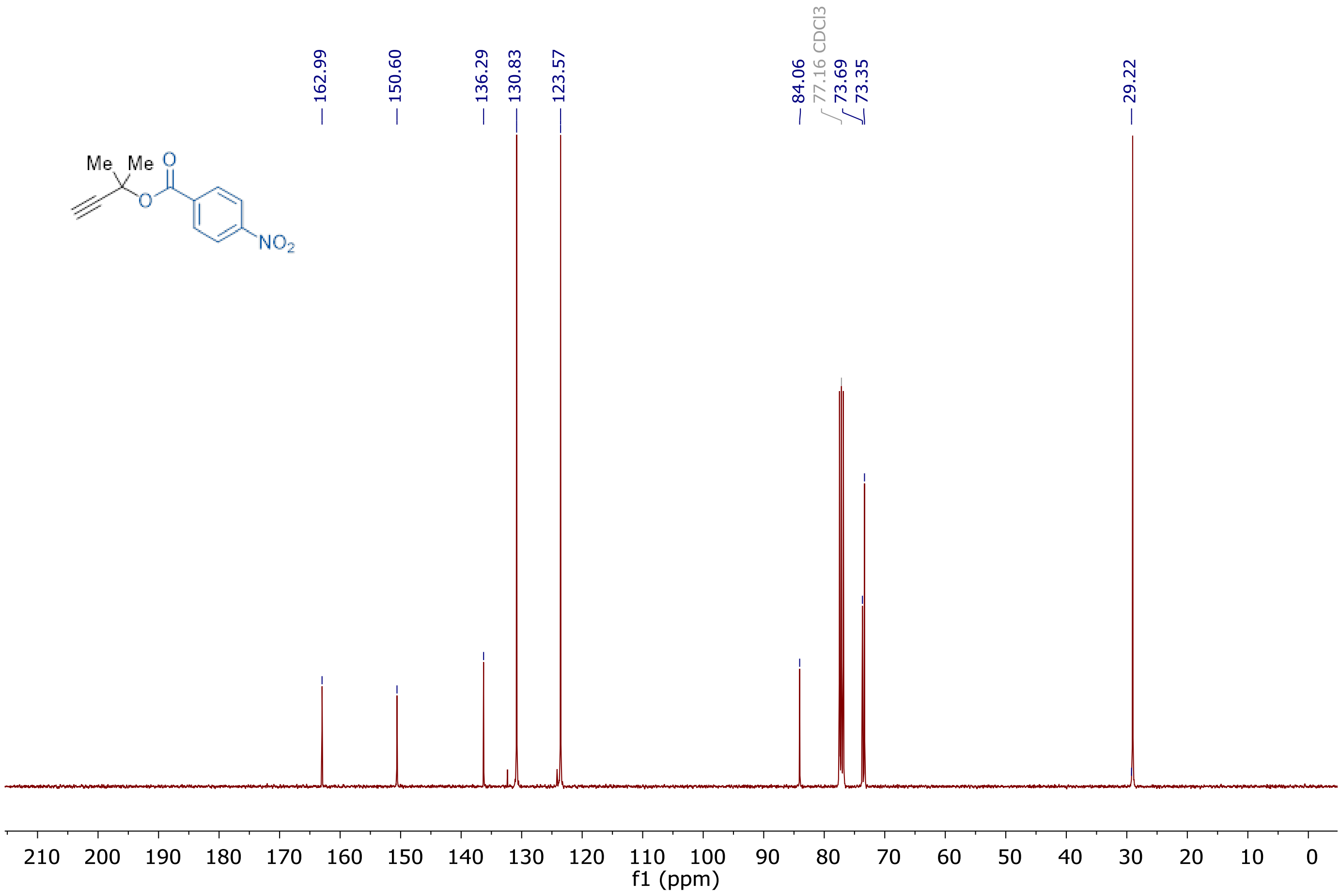

^{13}C NMR of compound 1f (101 MHz, $CDCl_3$)

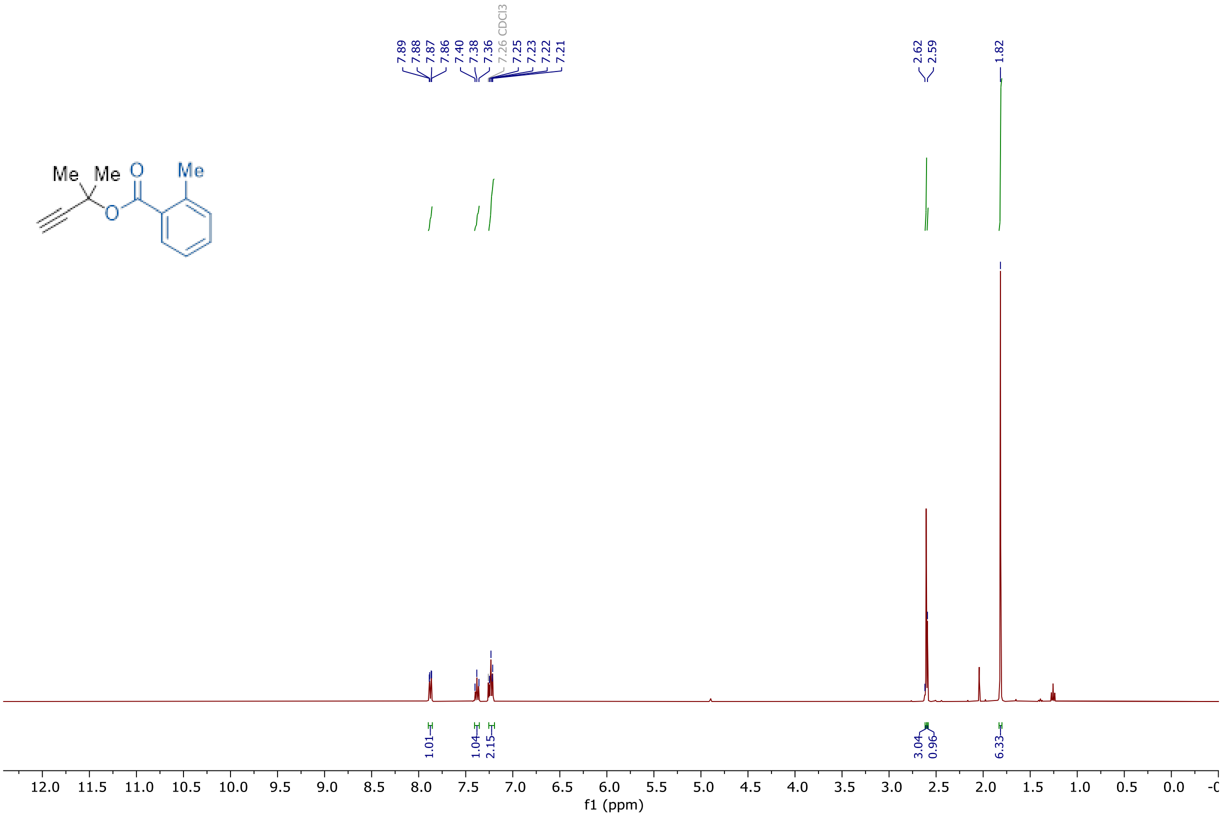

^{1}**H NMR** of compound **1g** (400 MHz, $CDCl_3$)

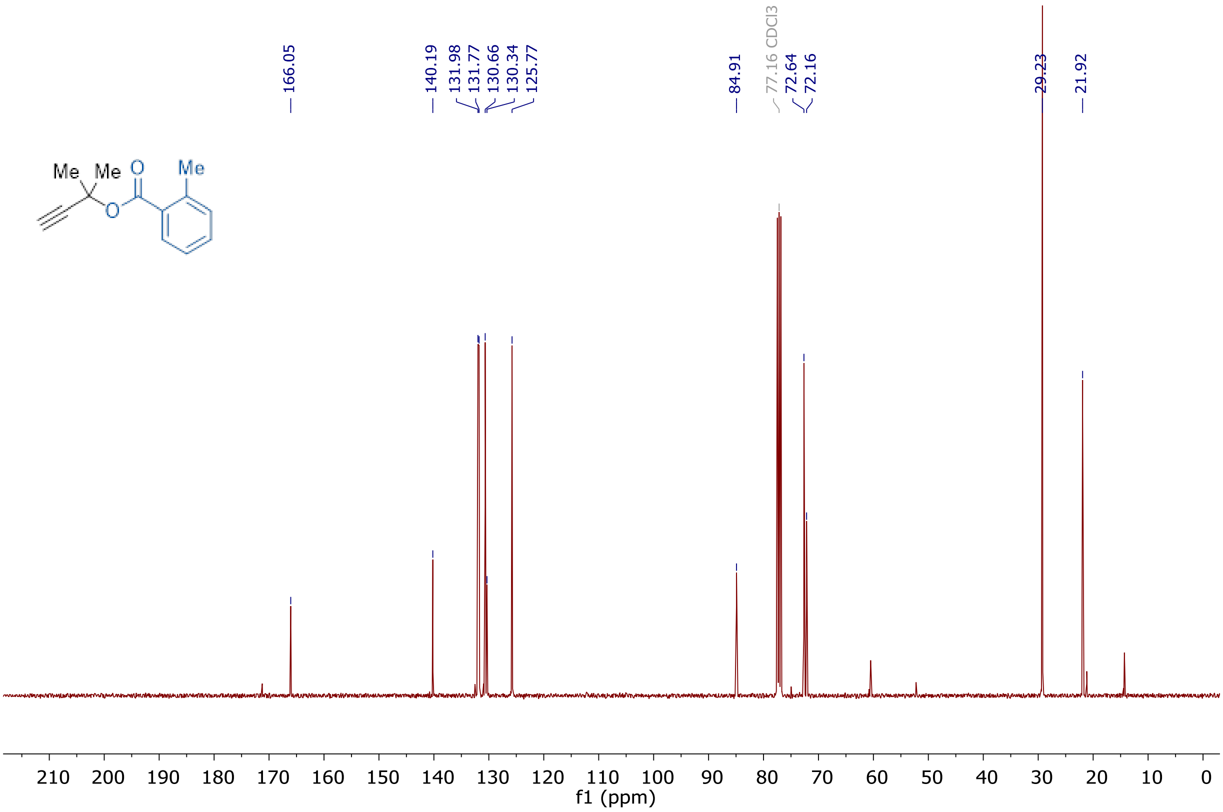

^{13}**C NMR** of compound **1g** (101 MHz, $CDCl_3$)

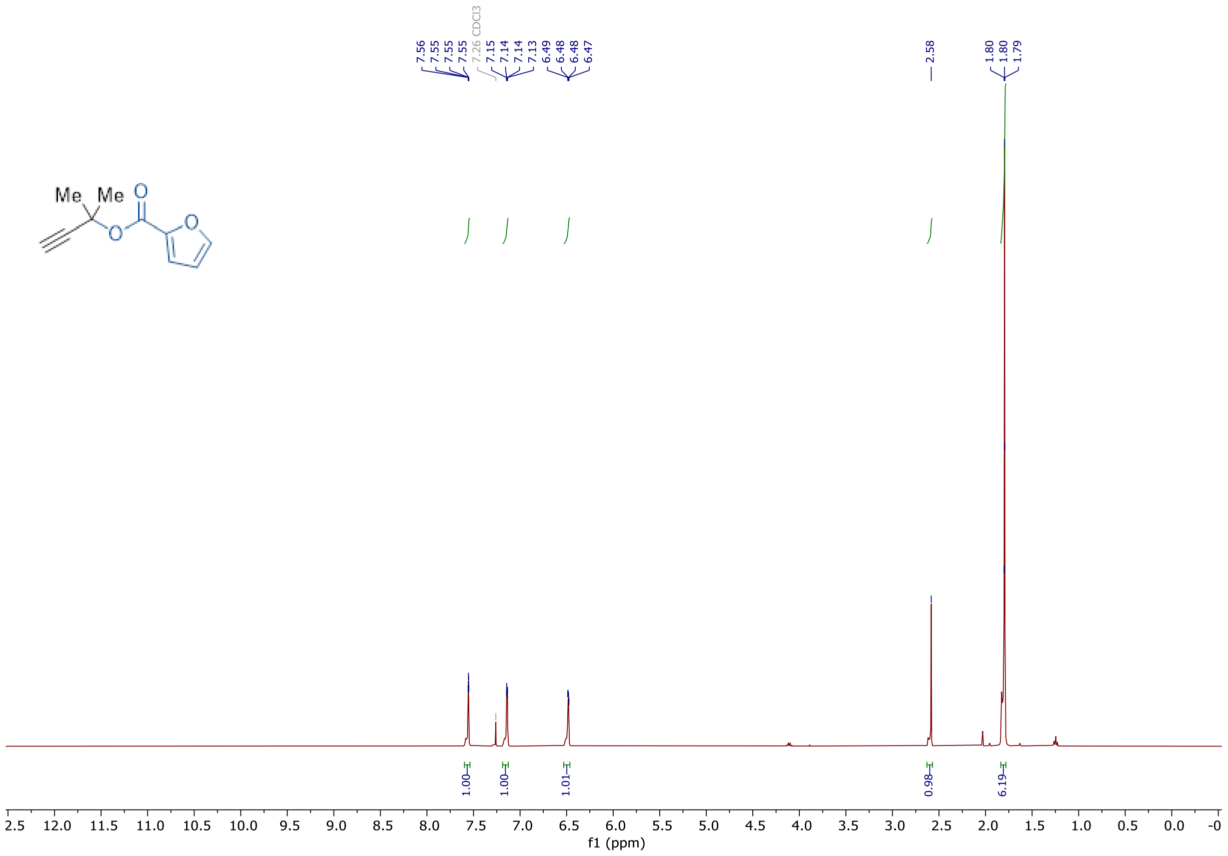

^{1}**H NMR** of compound **1h** (400 MHz, $CDCl_3$)

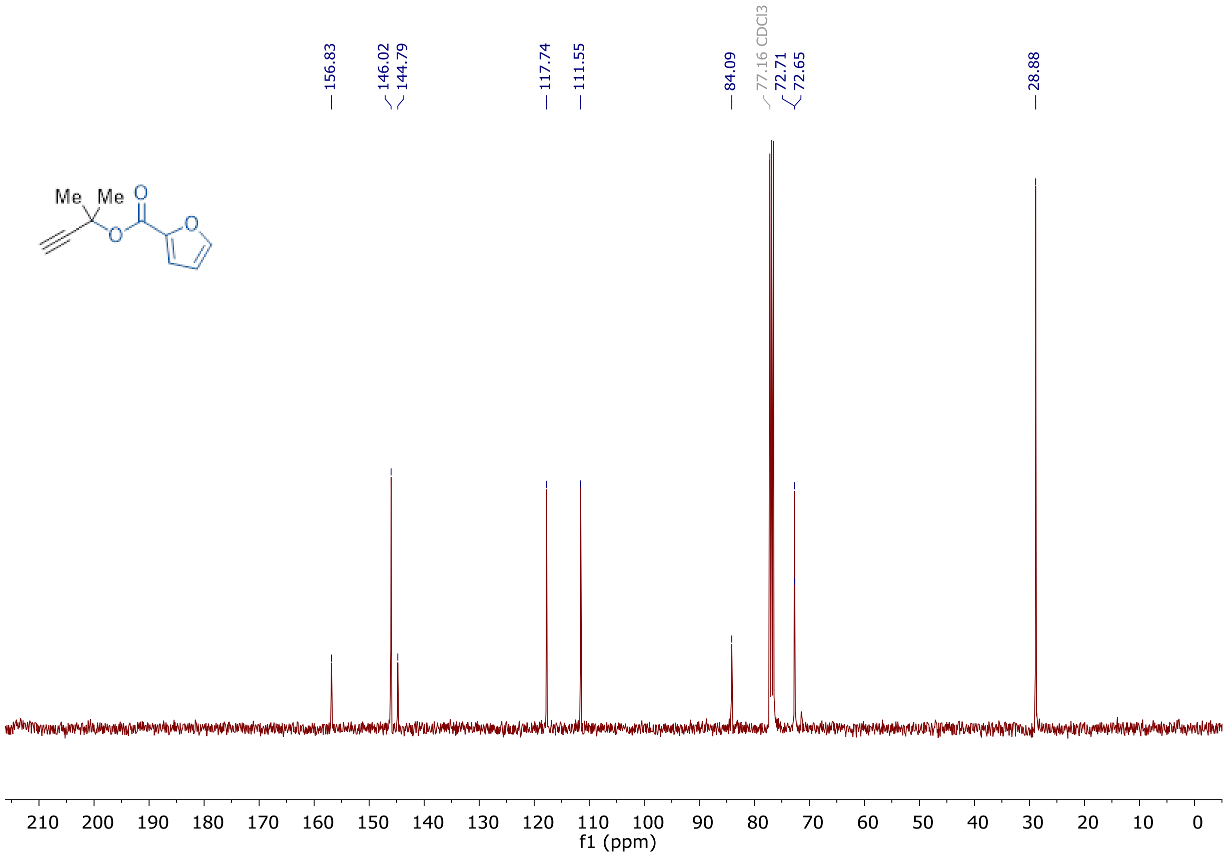

^{13}**C NMR** of compound **1h** (101 MHz, $CDCl_3$)

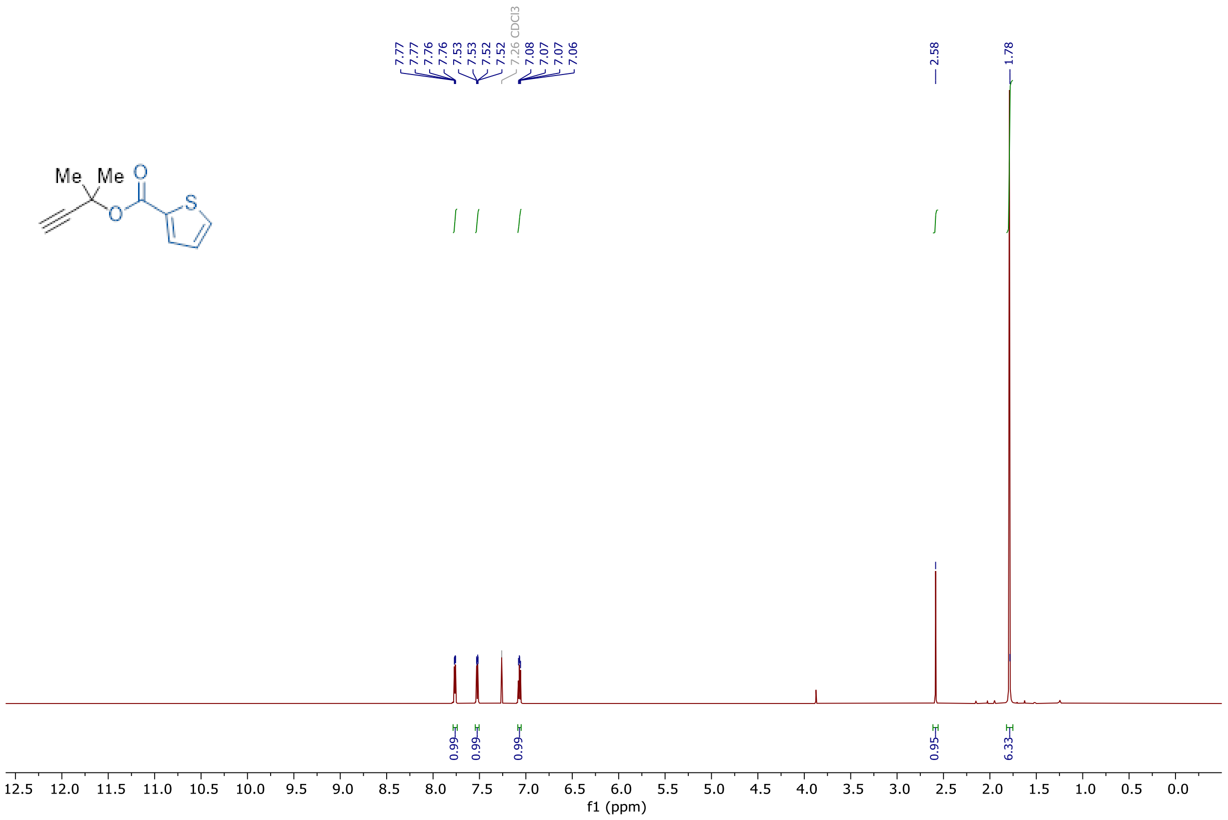

^{1}H NMR of compound **1i** (400 MHz, $CDCl_3$)

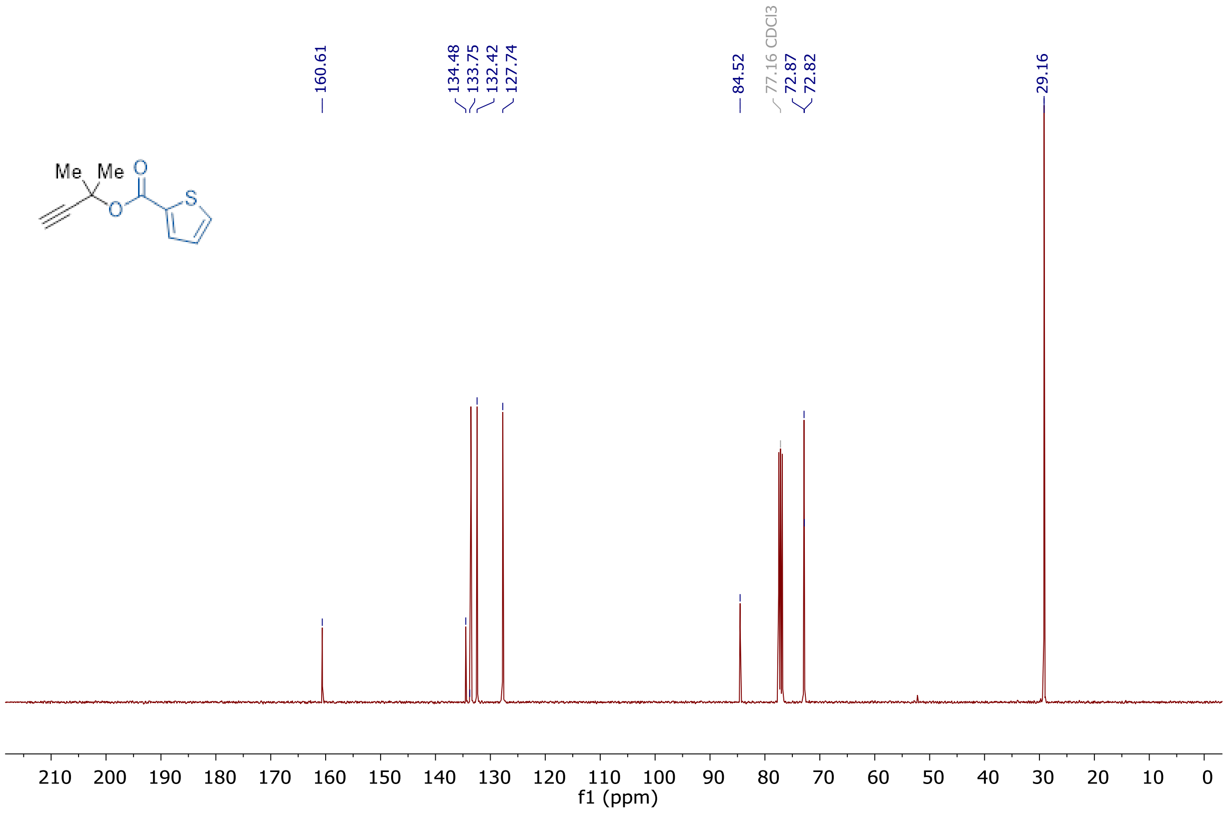

^{13}C NMR of compound **1i** (101 MHz, $CDCl_3$)

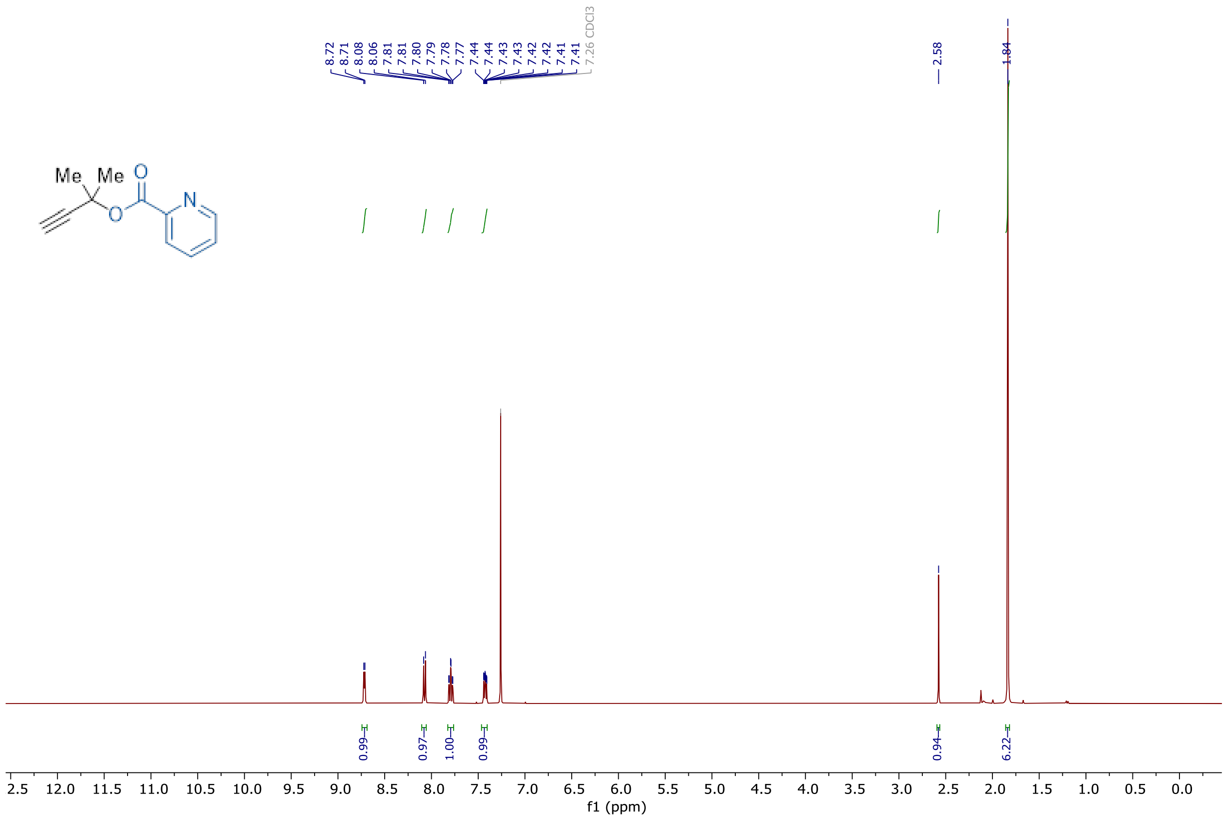

^{1}**H NMR** of compound **1j** (400 MHz, $CDCl_3$)

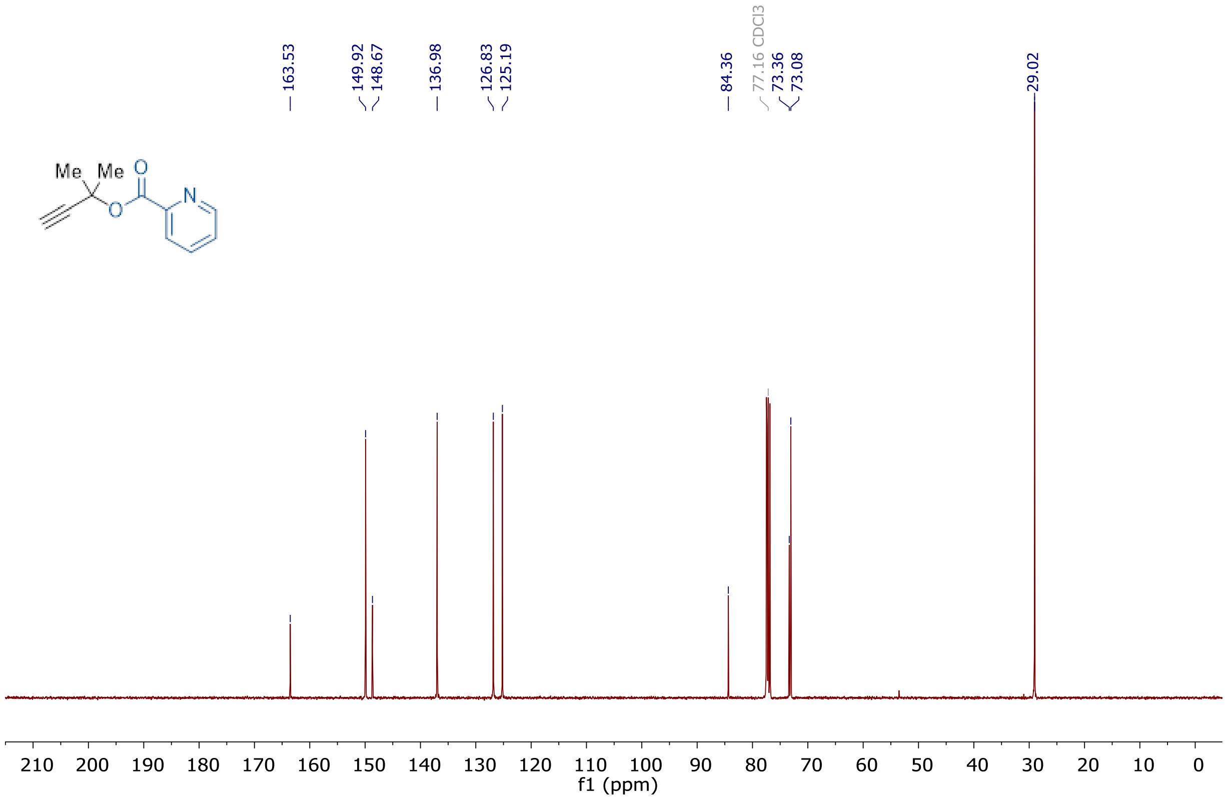

^{13}**C NMR** of compound **1j** (101 MHz, $CDCl_3$)

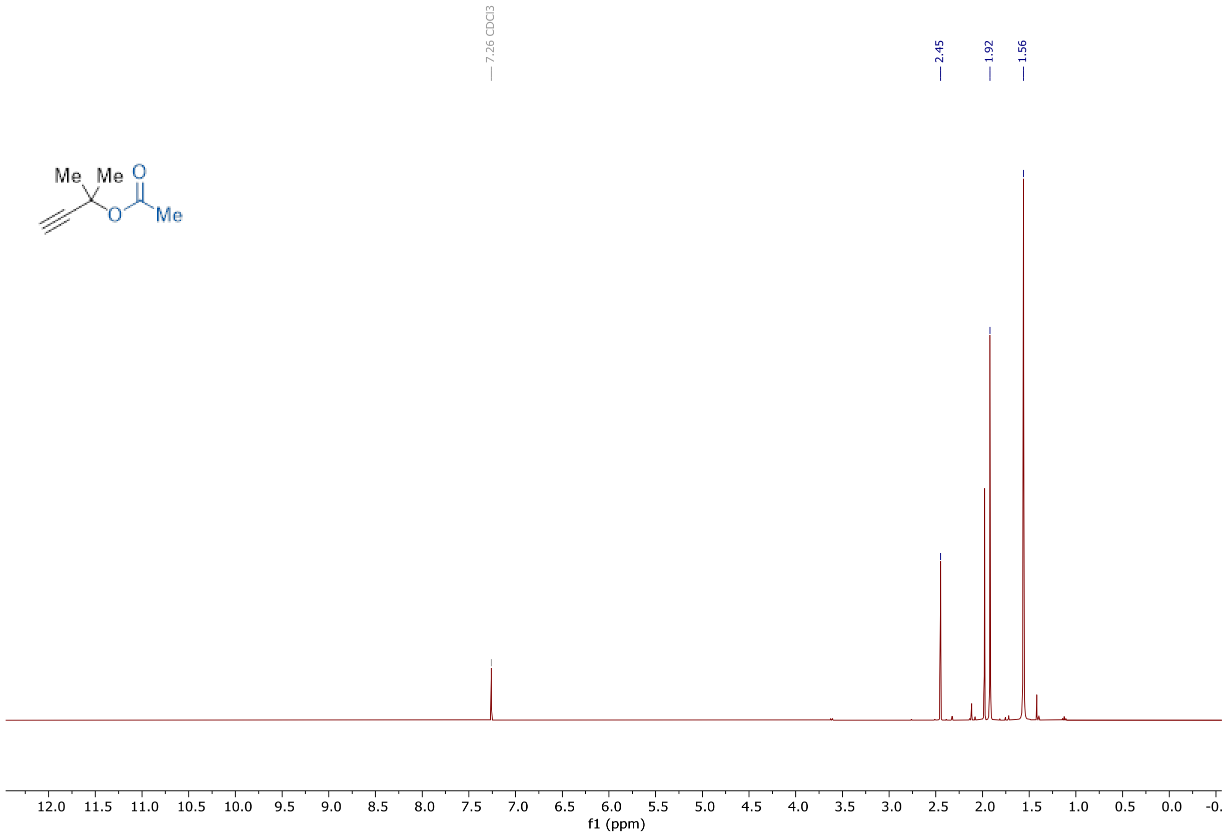

^{1}H NMR of compound **1k** (400 MHz, $CDCl_3$)

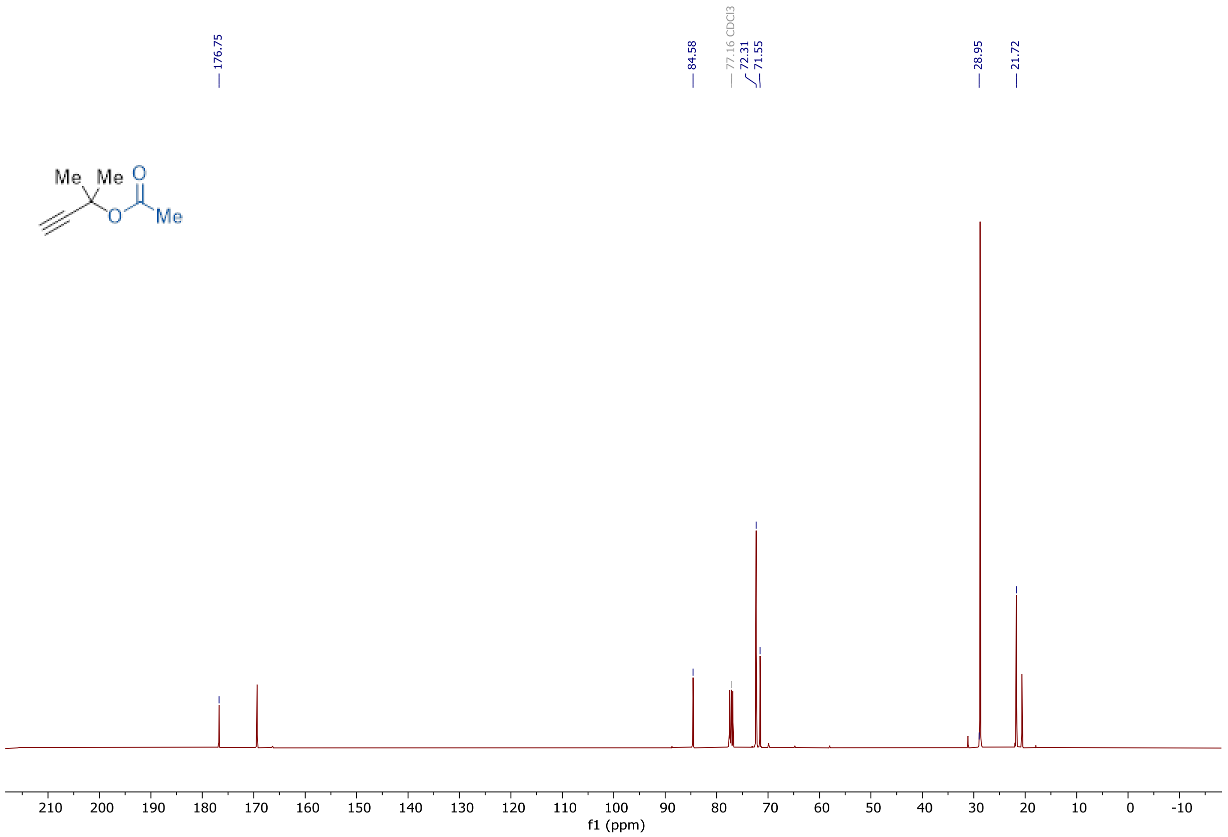

^{13}C NMR of compound **1k** (101 MHz, $CDCl_3$)

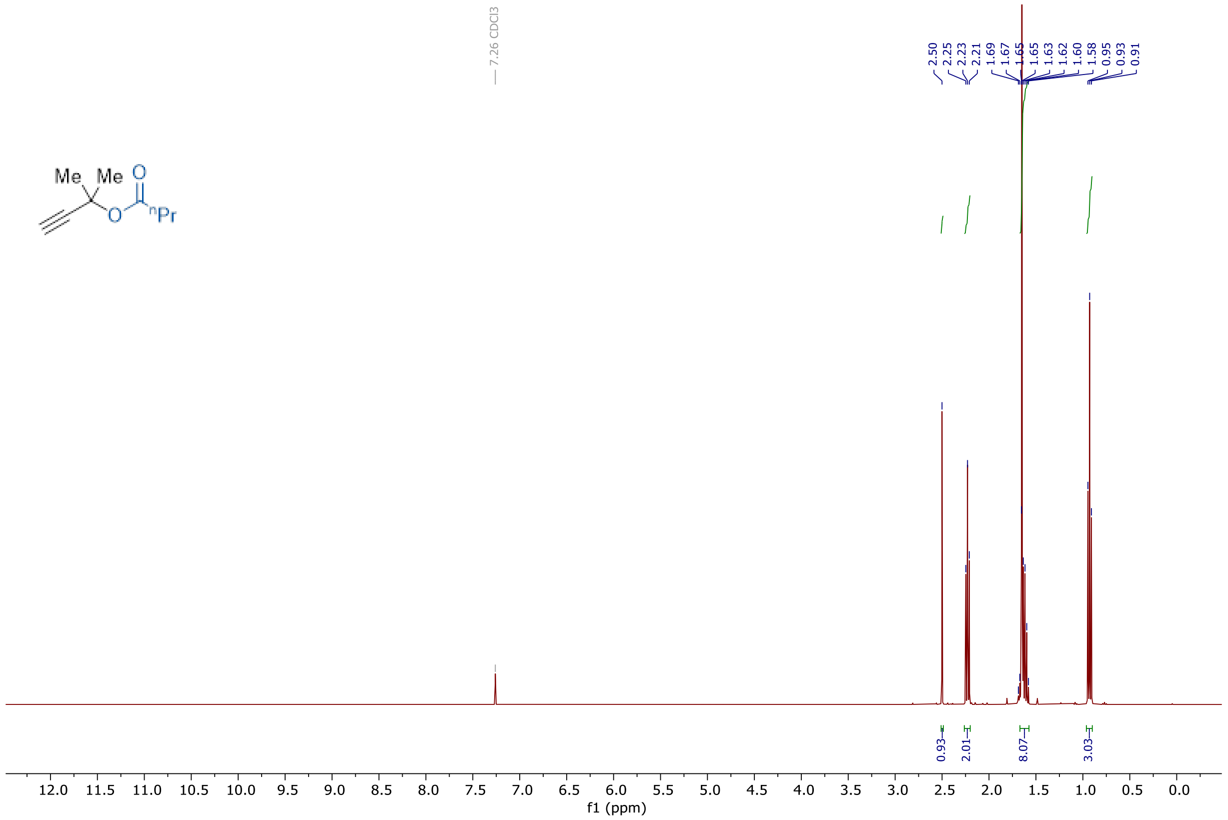

^{1}H NMR of compound **1l** (400 MHz, $CDCl_3$)

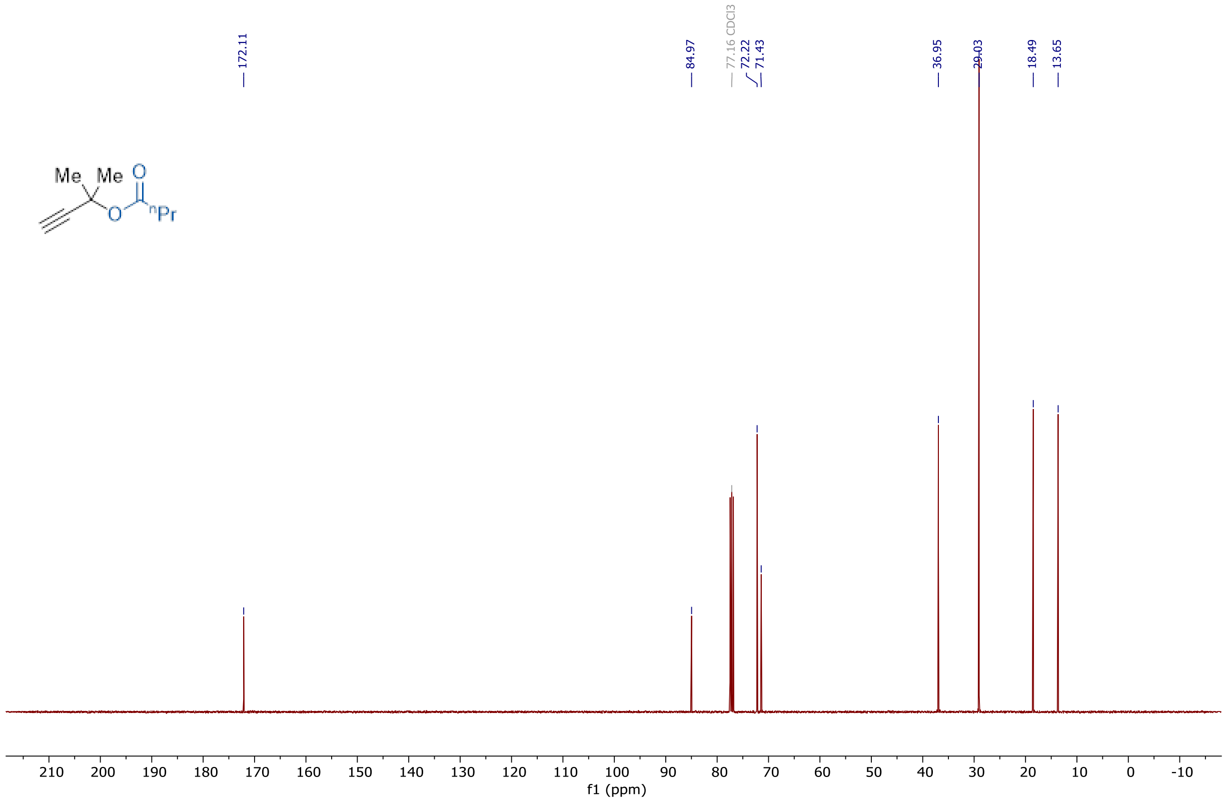

^{13}C NMR of compound **1l** (101 MHz, $CDCl_3$)

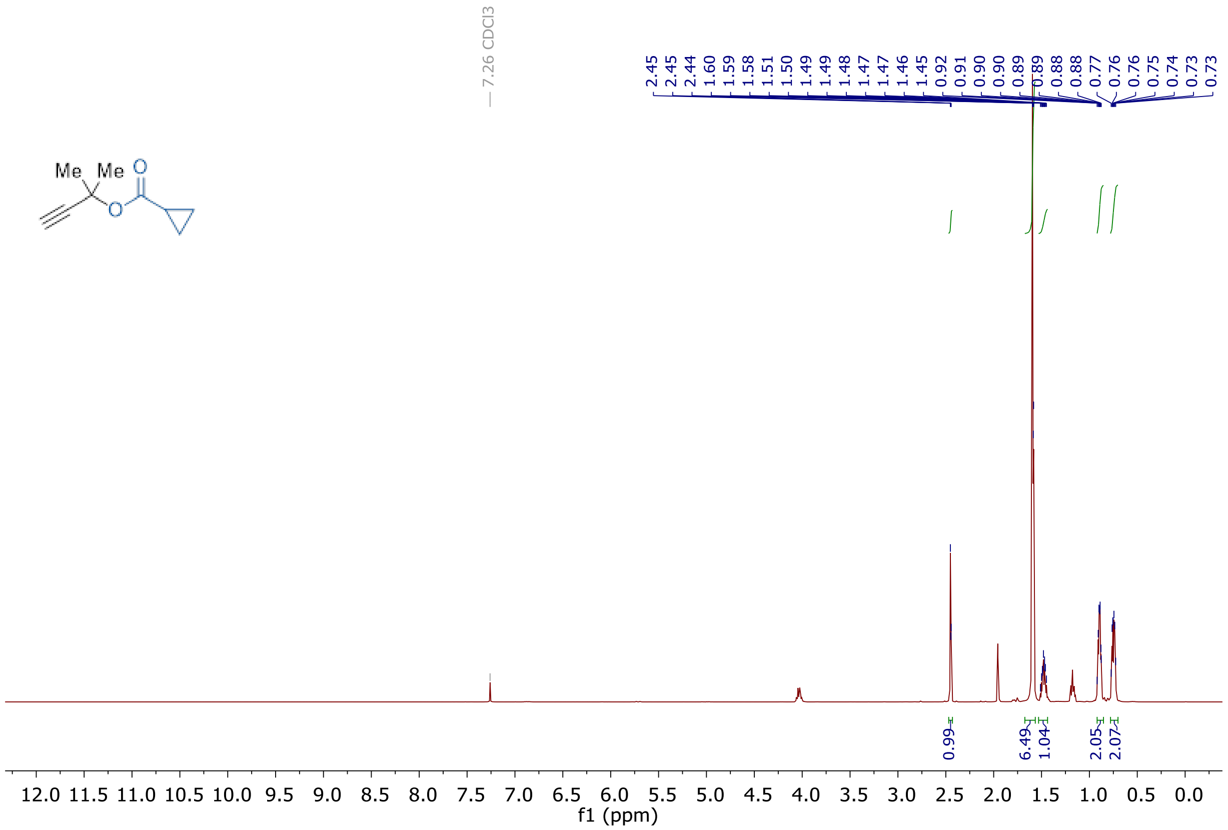

[1]**H NMR** of compound **1m** (400 MHz, $CDCl_3$)

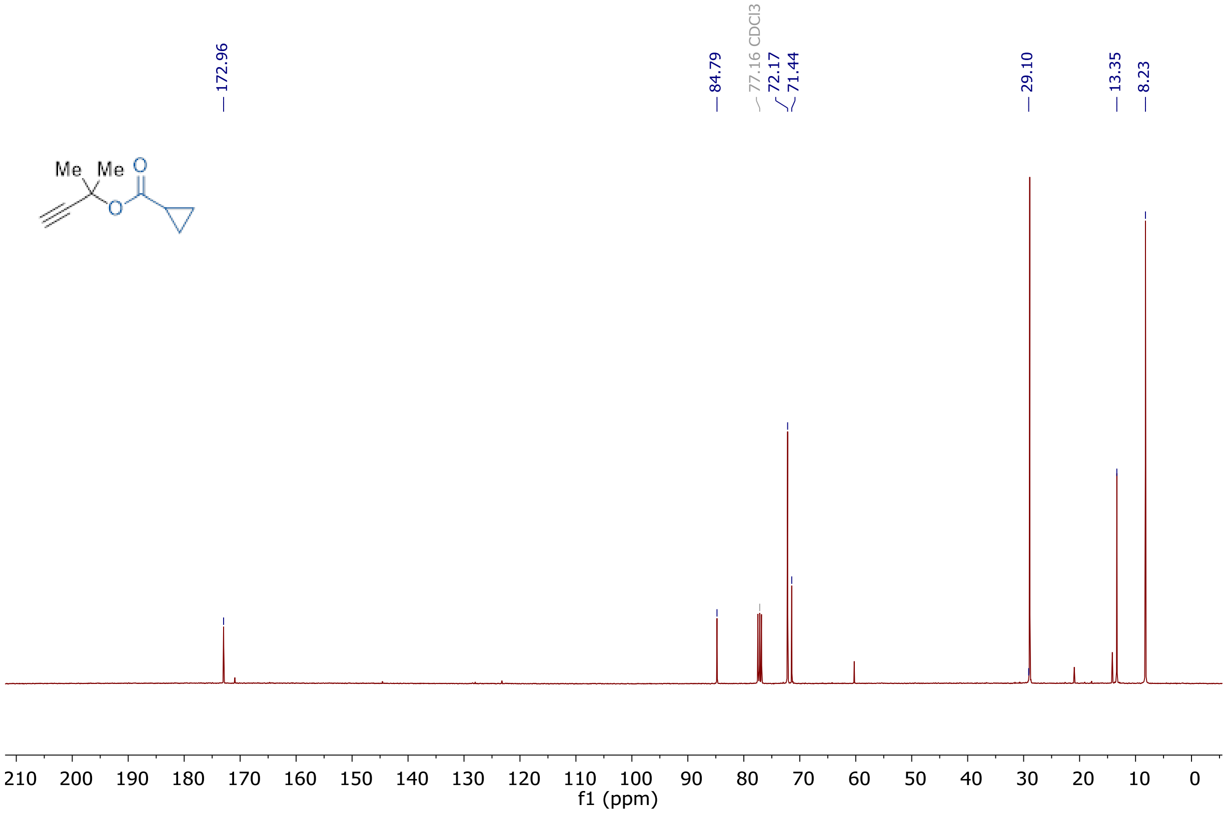

[13]**C NMR** of compound **1m** (101 MHz, $CDCl_3$)

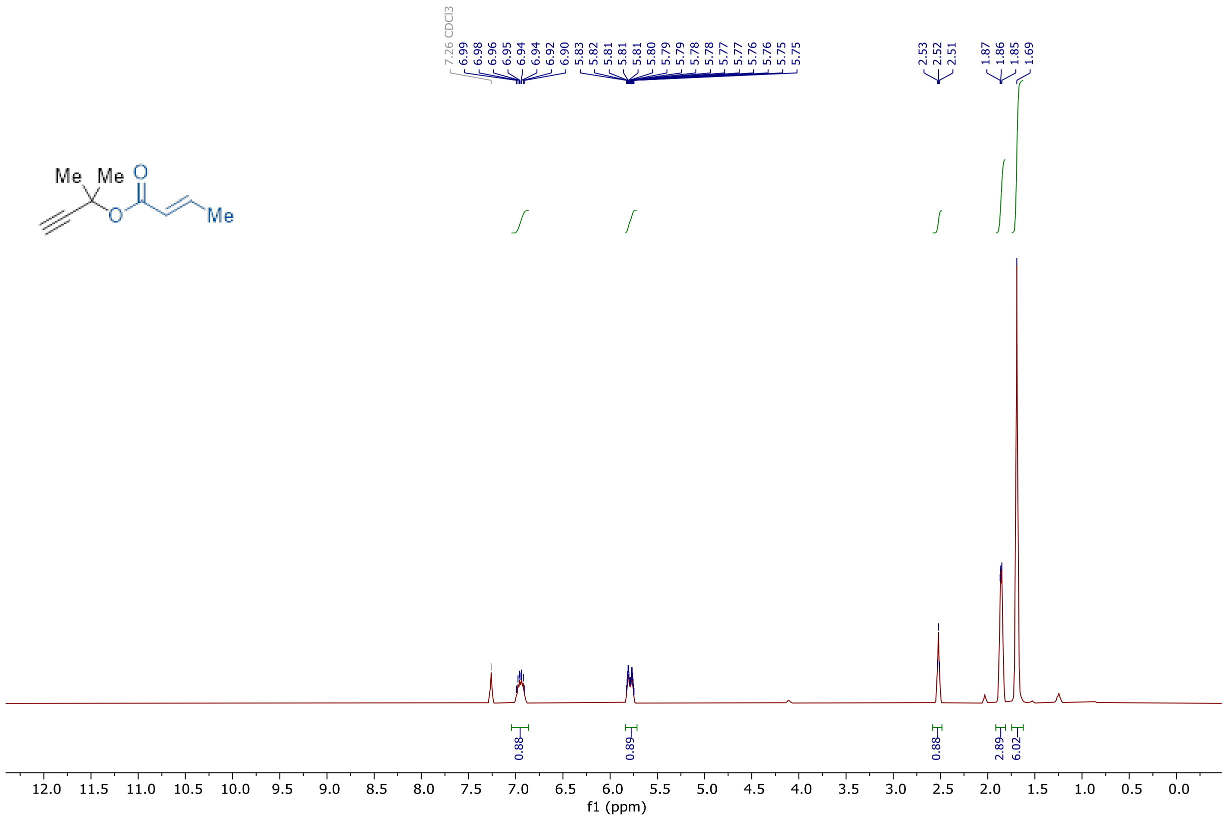

^{1}H NMR of compound **1n**(400 MHz, $CDCl_3$)

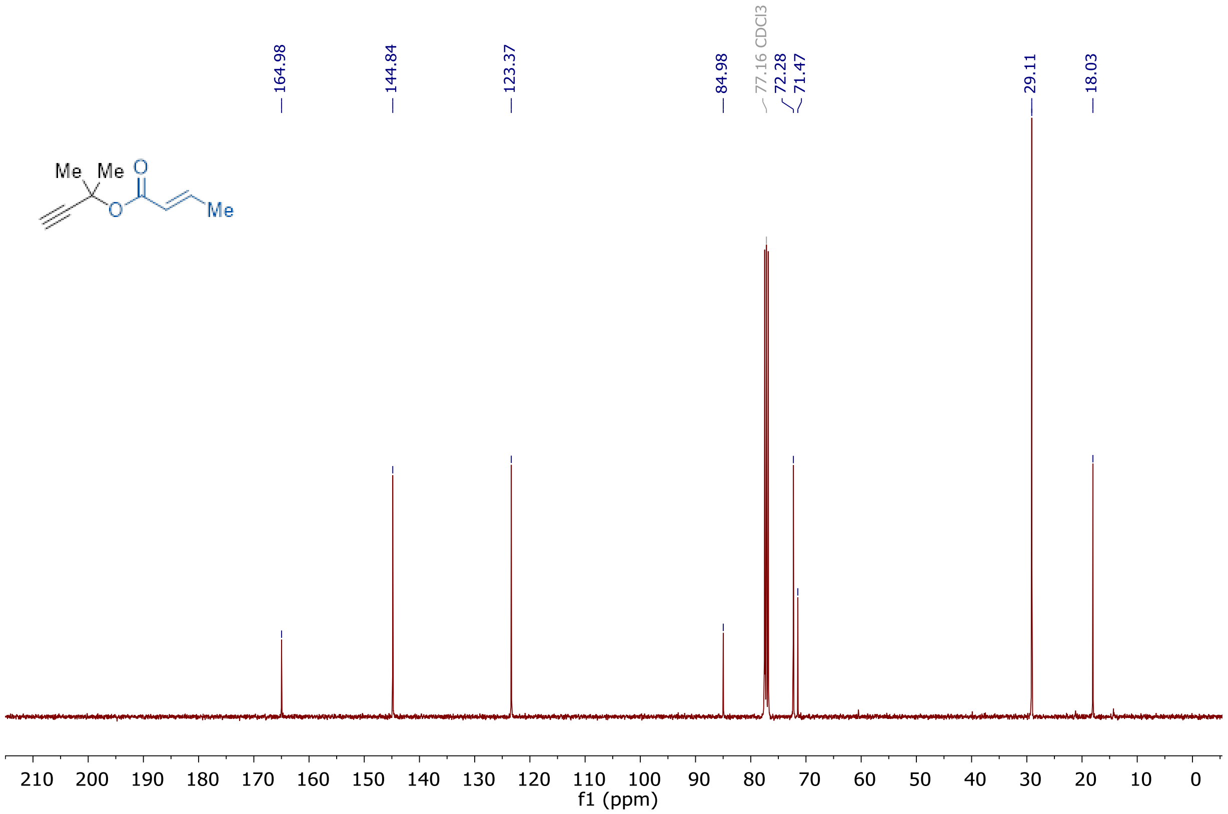

^{13}C NMR of compound **1n** (101 MHz, $CDCl_3$)

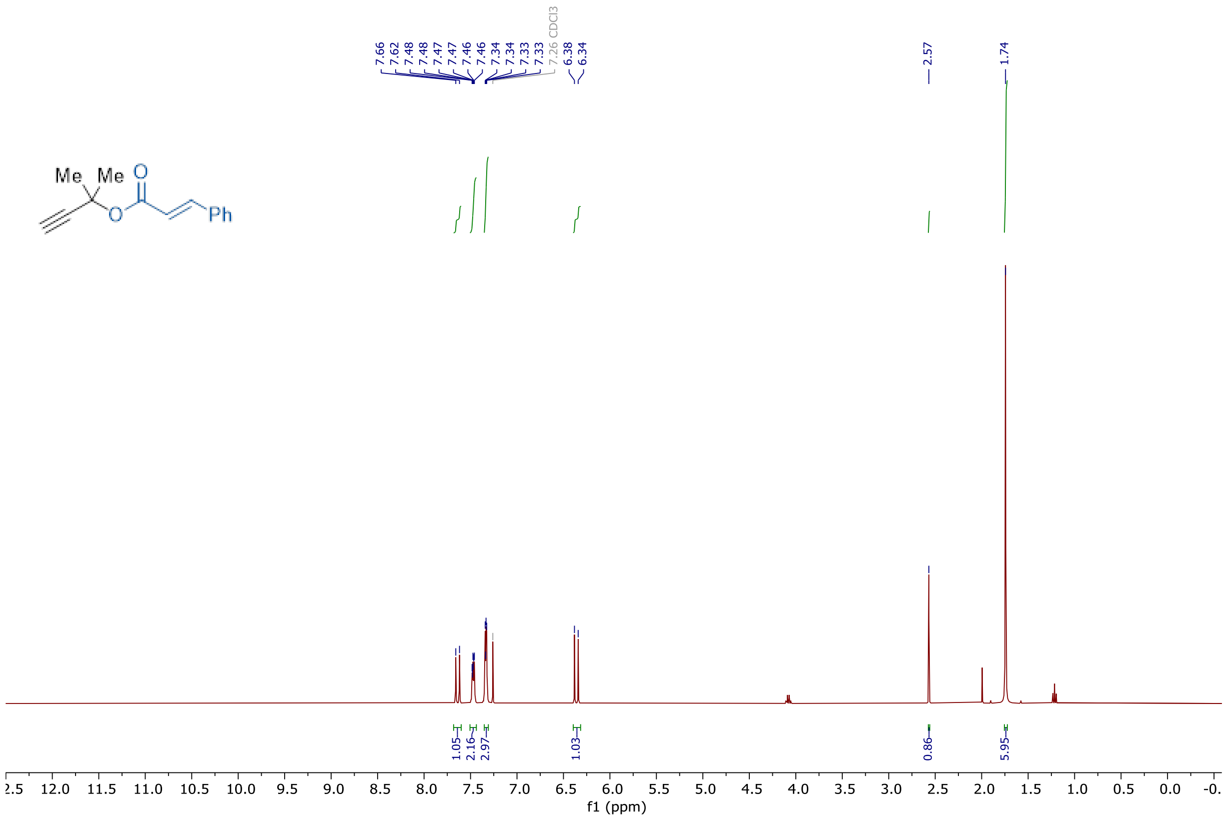

^{1}**H NMR** of compound **1o** (400 MHz, $CDCl_3$)

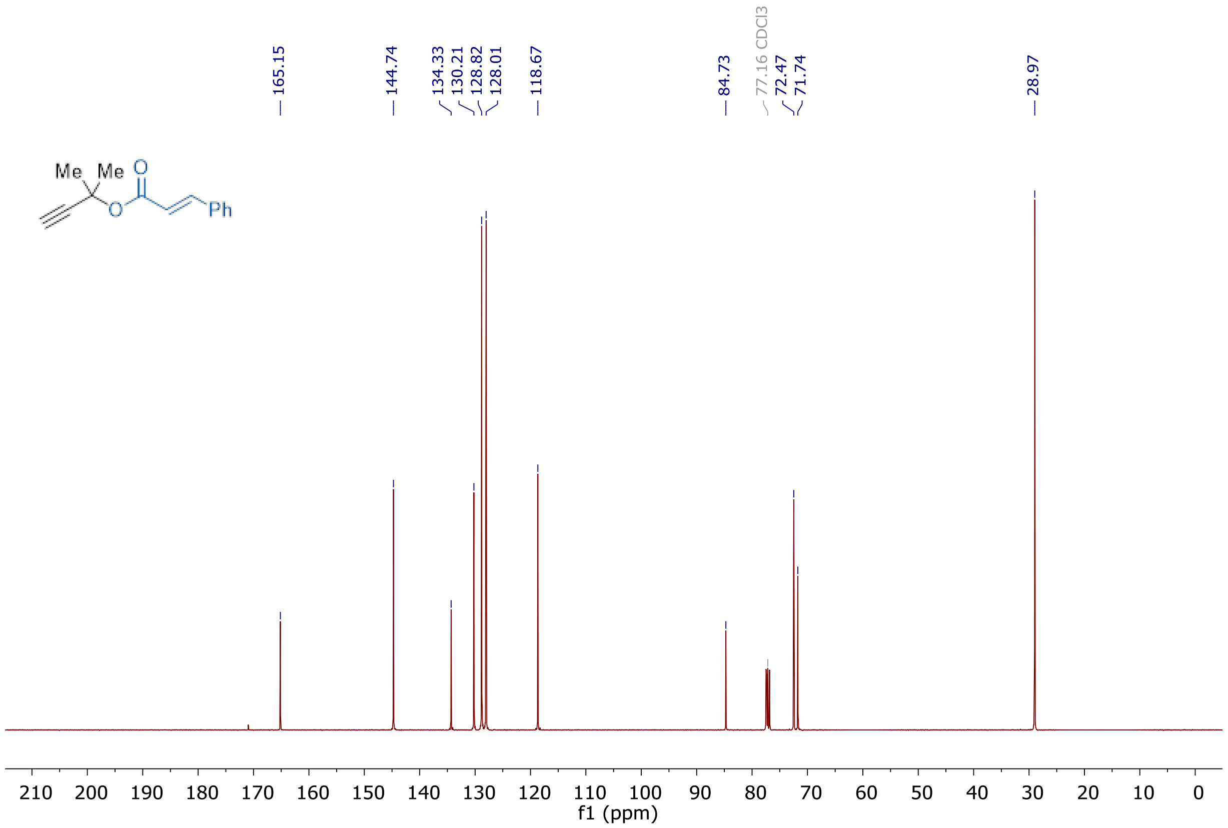

^{13}**C NMR** of compound **1o** (101 MHz, $CDCl_3$)

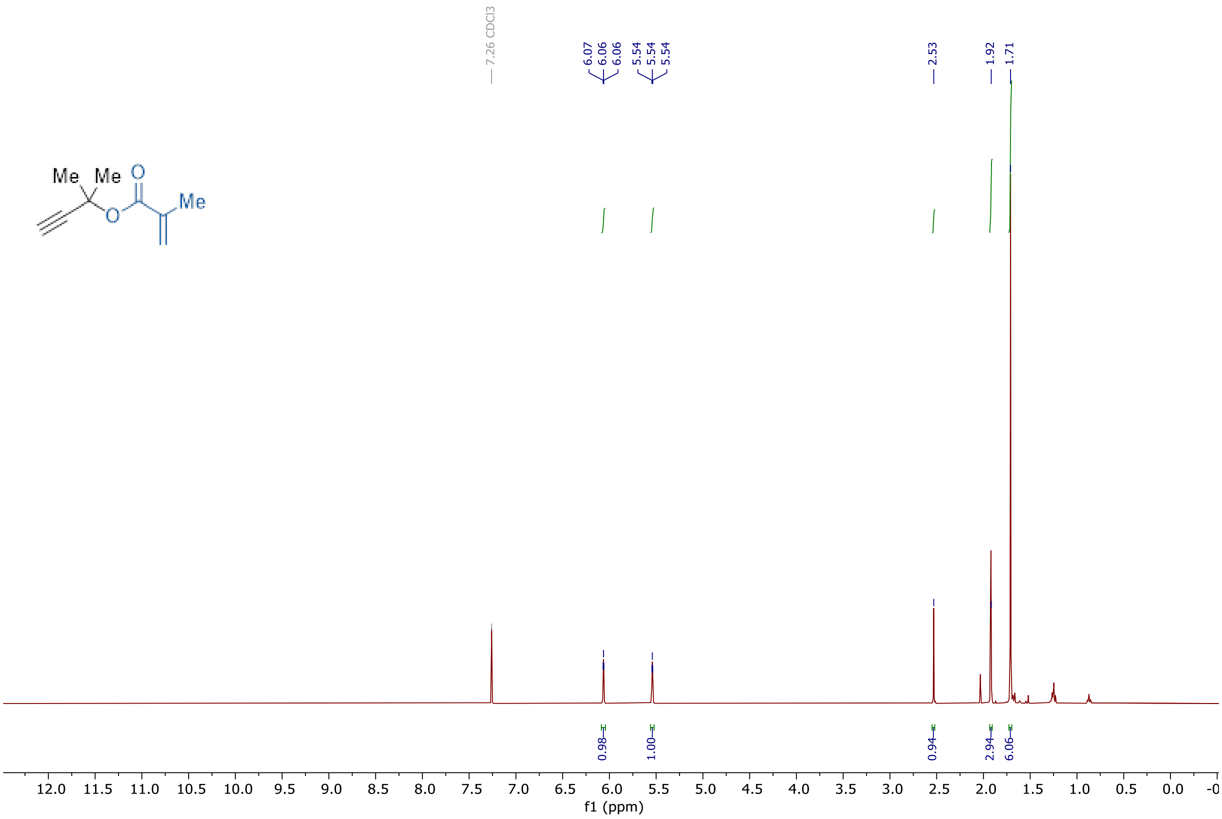

^{1}**H NMR** of compound **1p** (400 MHz, $CDCl_3$)

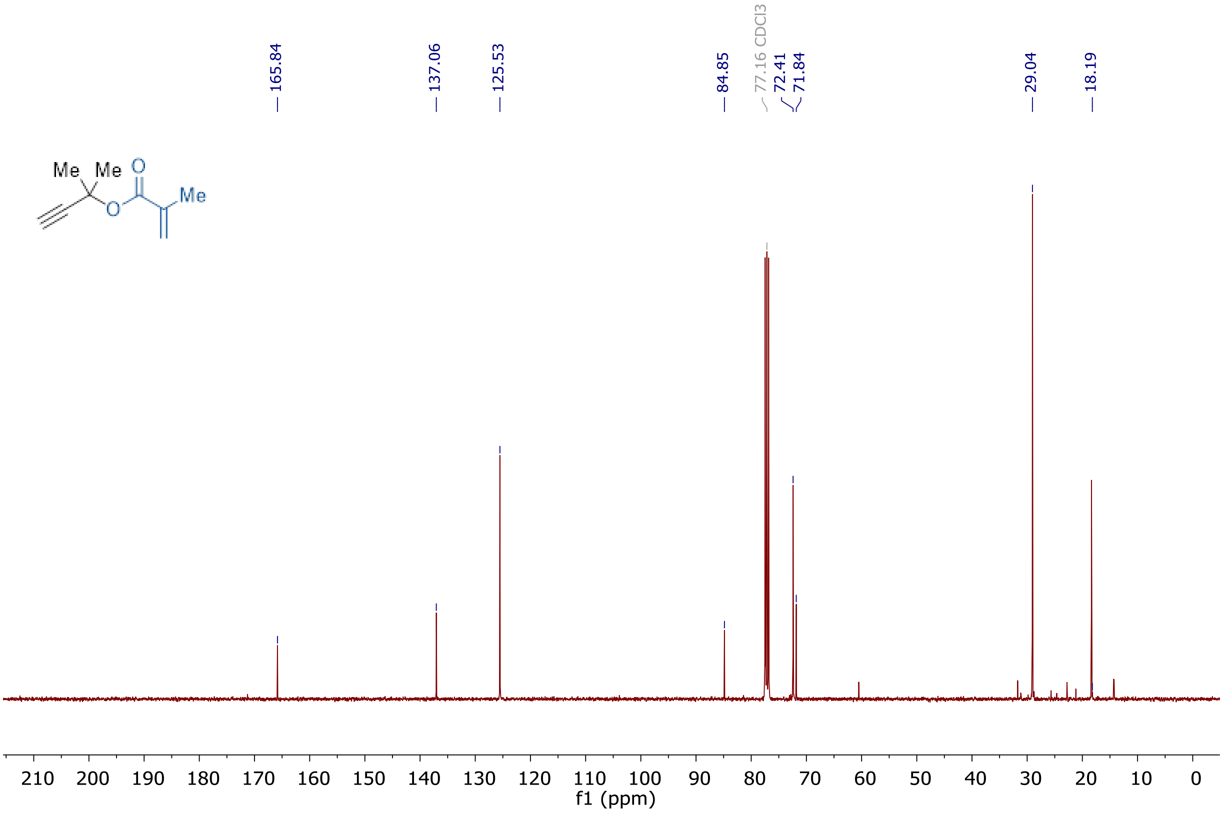

^{13}**C NMR** of compound **1p** (101 MHz, $CDCl_3$)

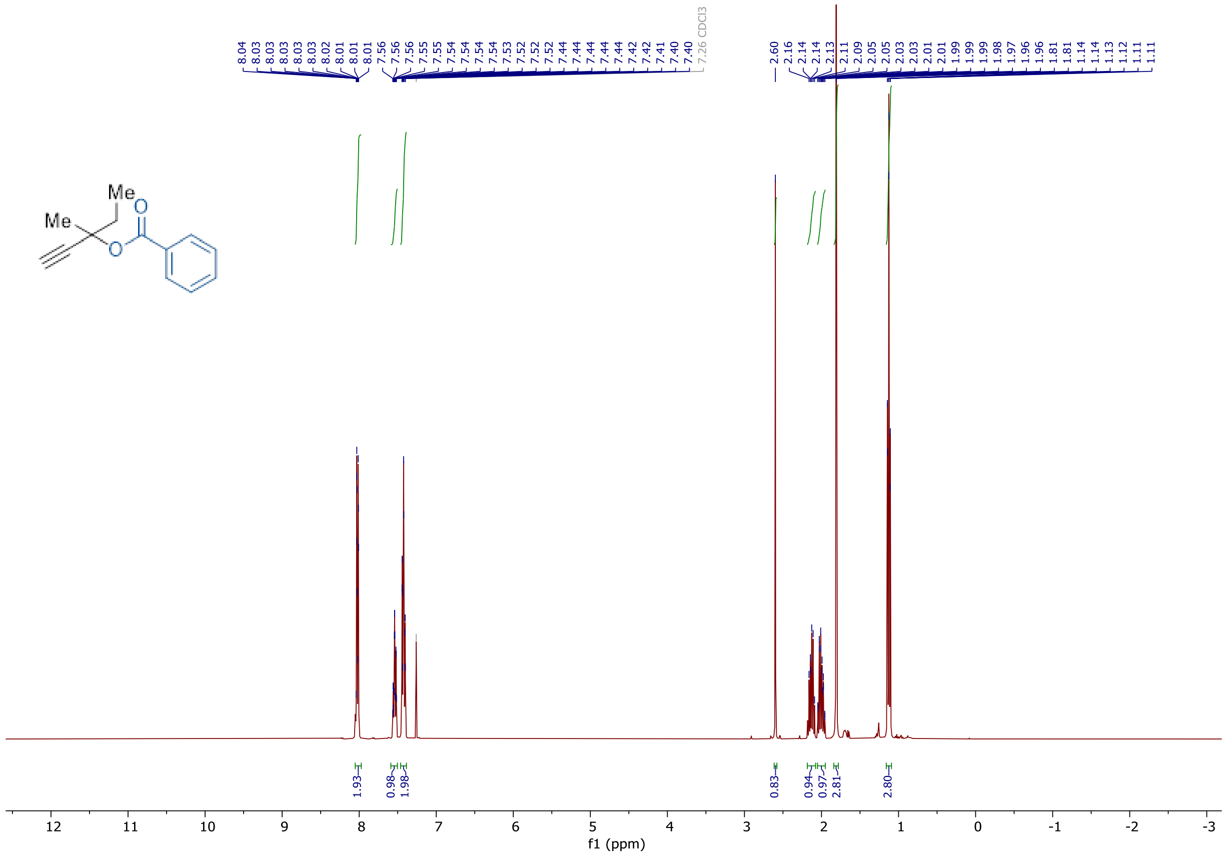

^{1}**H NMR** of compound **1q** (400 MHz, $CDCl_3$)

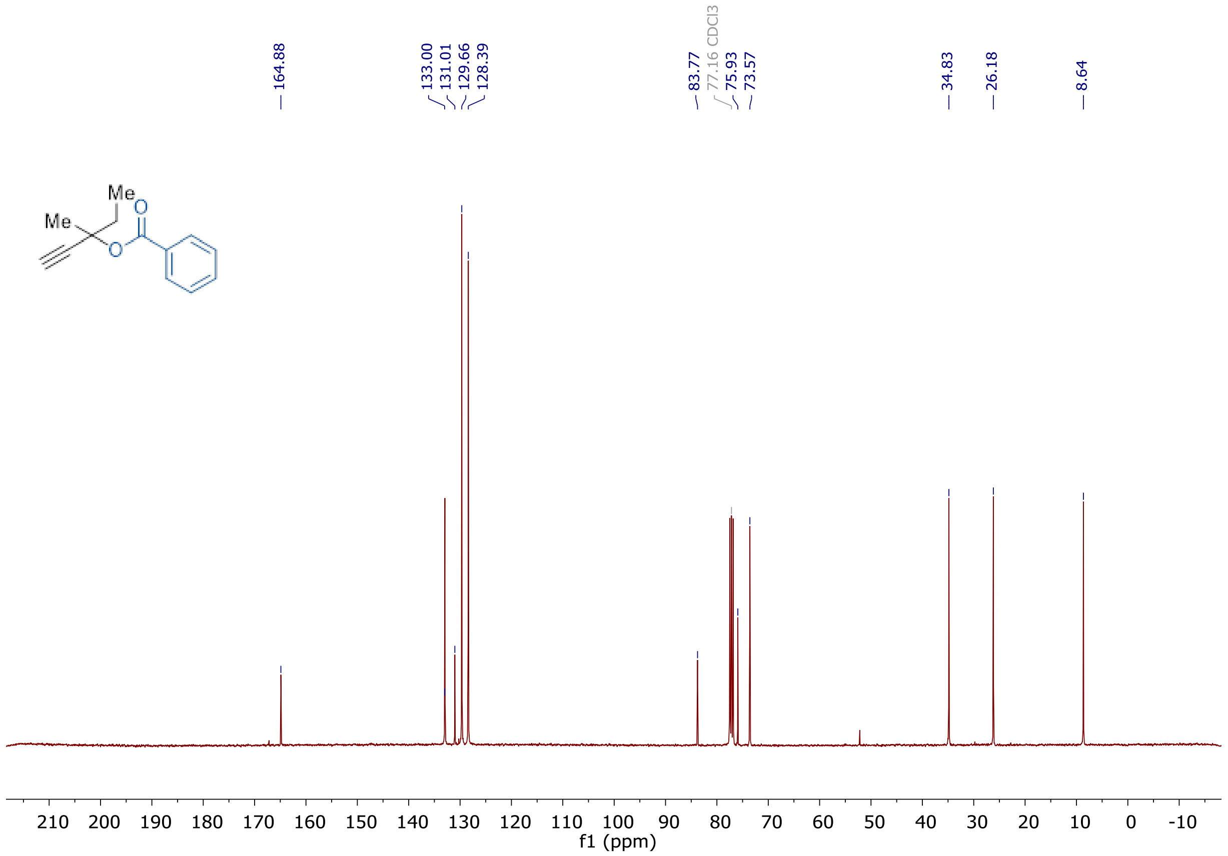

^{13}**C NMR** of compound **1q** (101 MHz, $CDCl_3$)

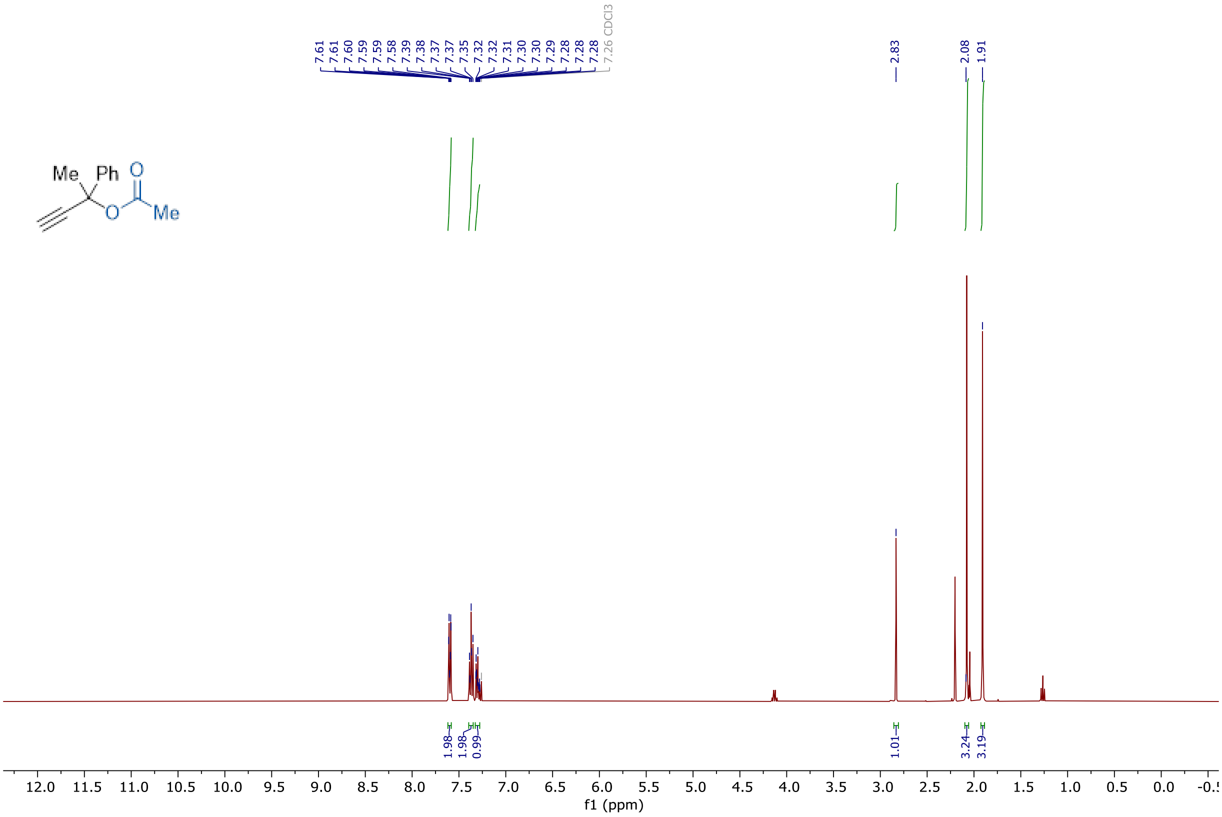

^{1}**H NMR** of compound **1r** (400 MHz, $CDCl_3$)

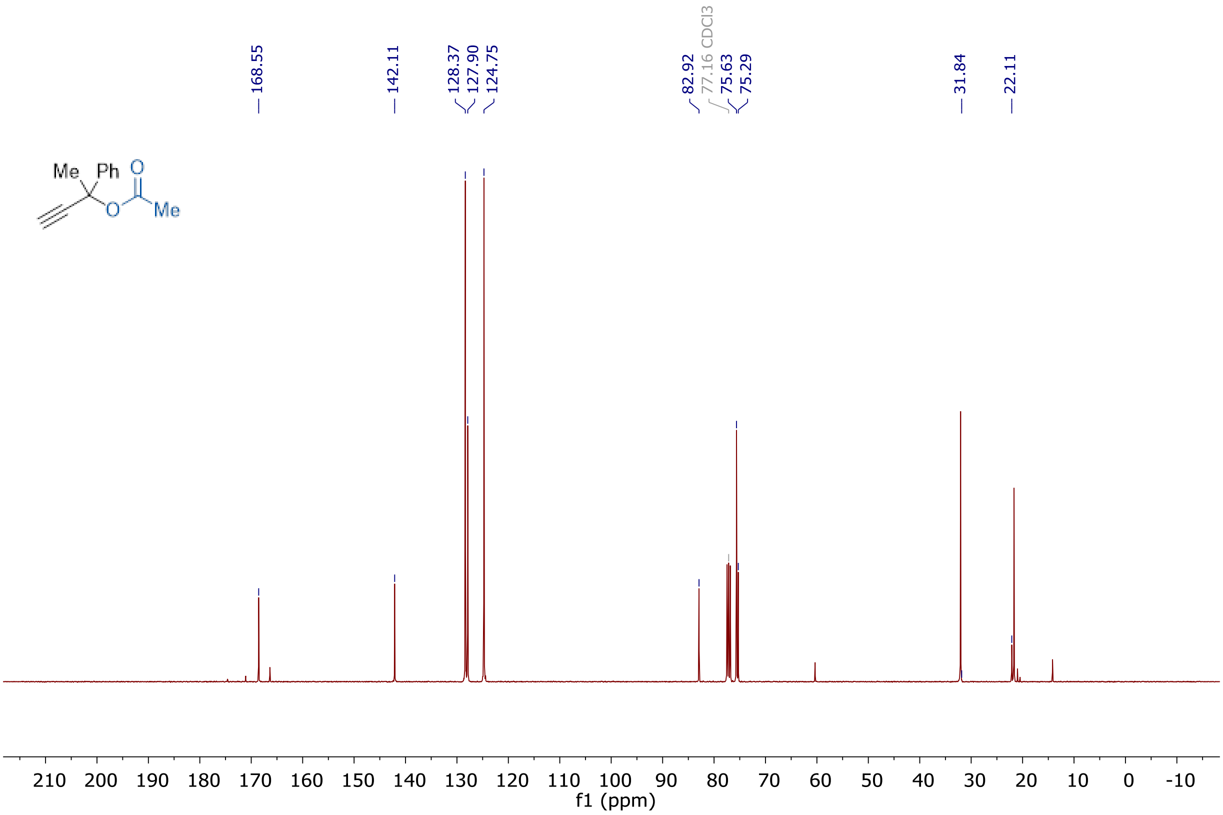

^{13}**C NMR** of compound **1r** (101 MHz, $CDCl_3$)

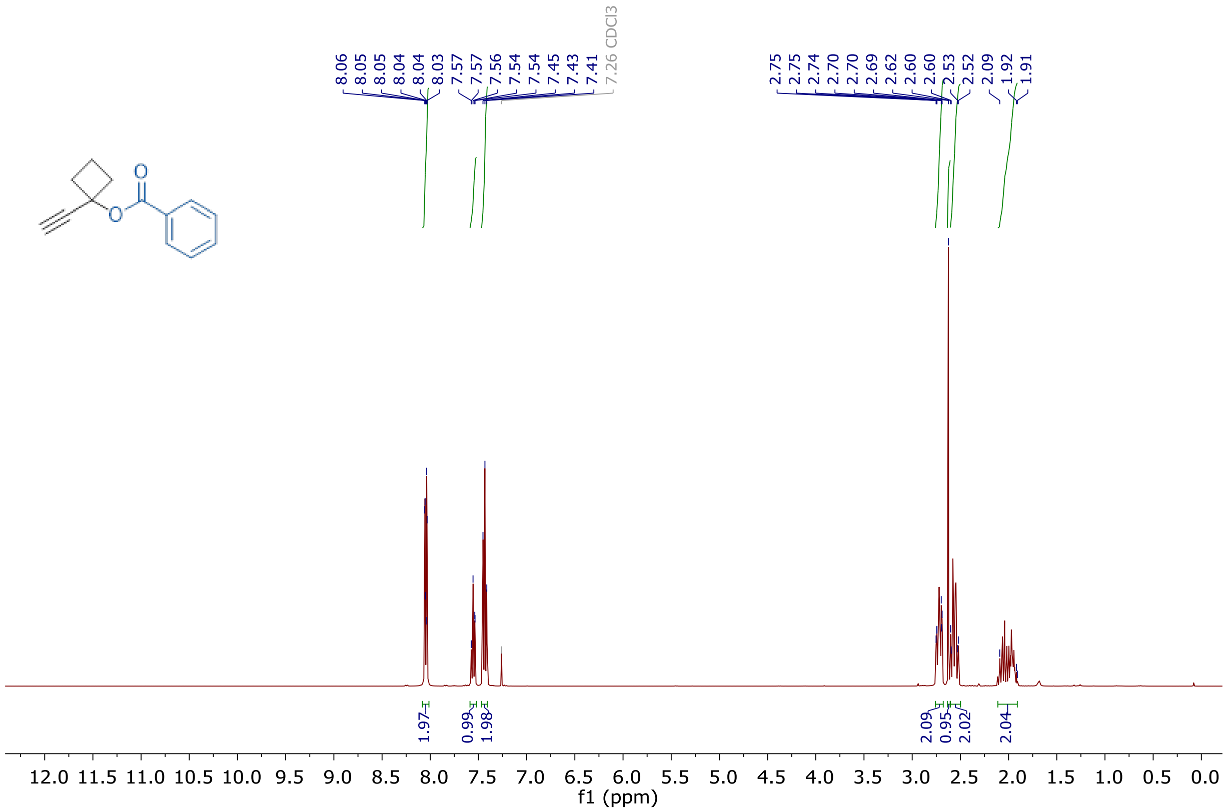

^{1}**H NMR** of compound **1s** (400 MHz, $CDCl_3$)

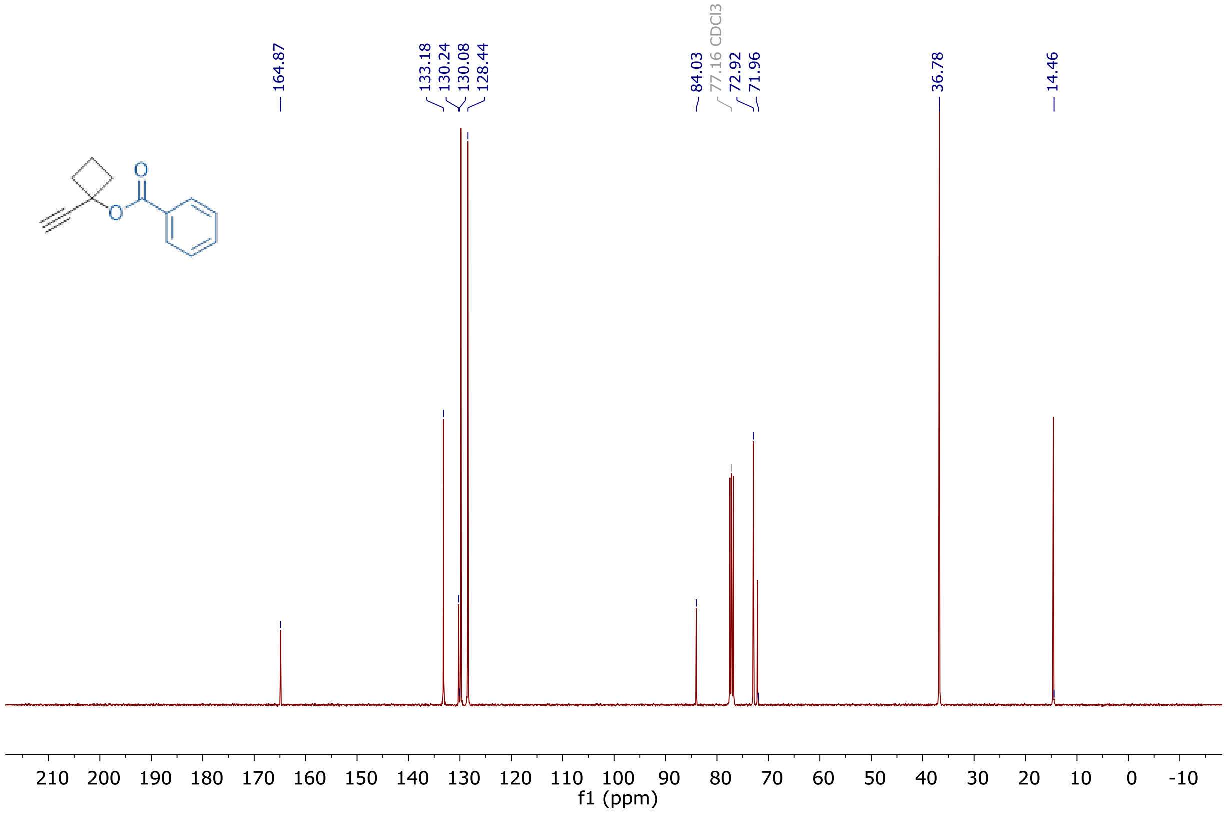

^{13}**C NMR** of compound **1s** (101 MHz, $CDCl_3$)

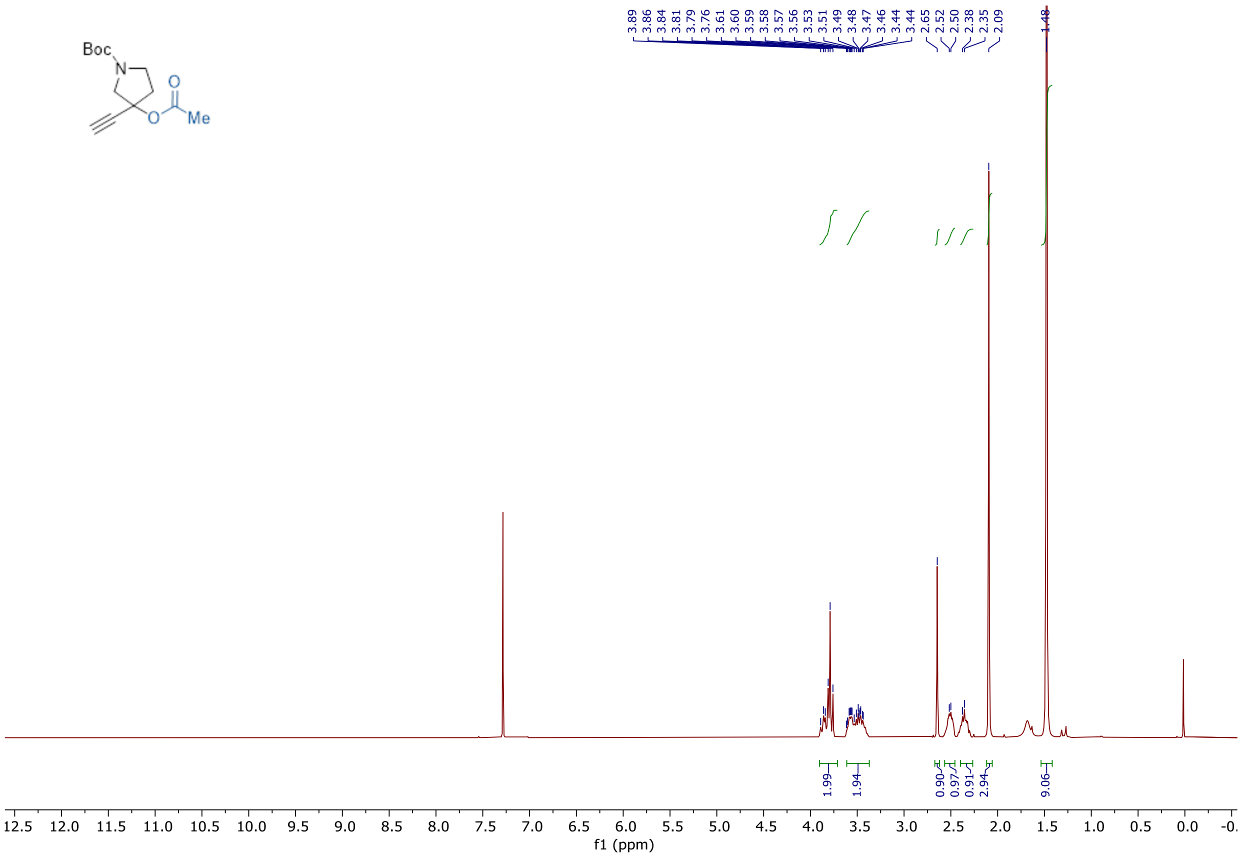

¹H NMR of compound **1t** (400 MHz, $CDCl_3$)

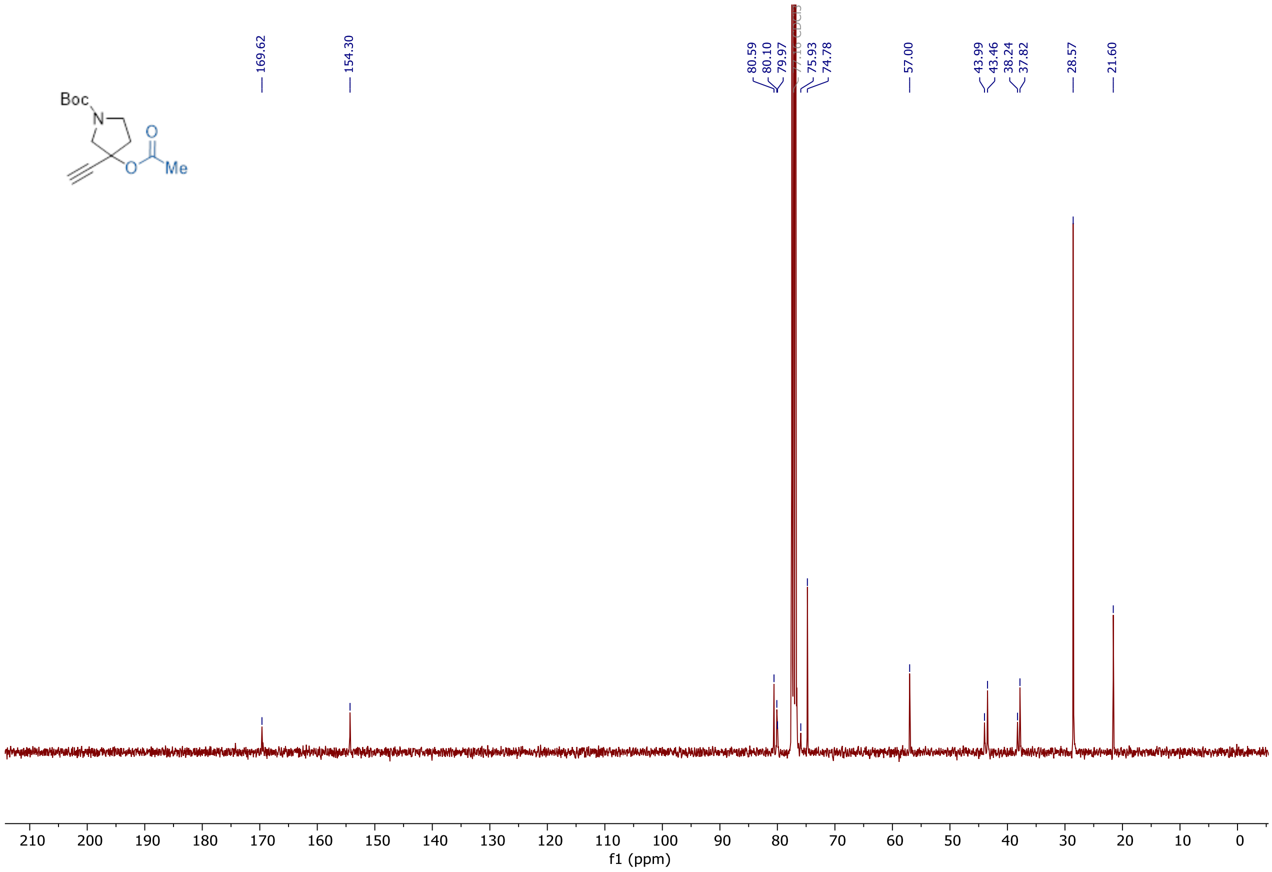

¹³C NMR of compound **1t** (101 MHz, $CDCl_3$)

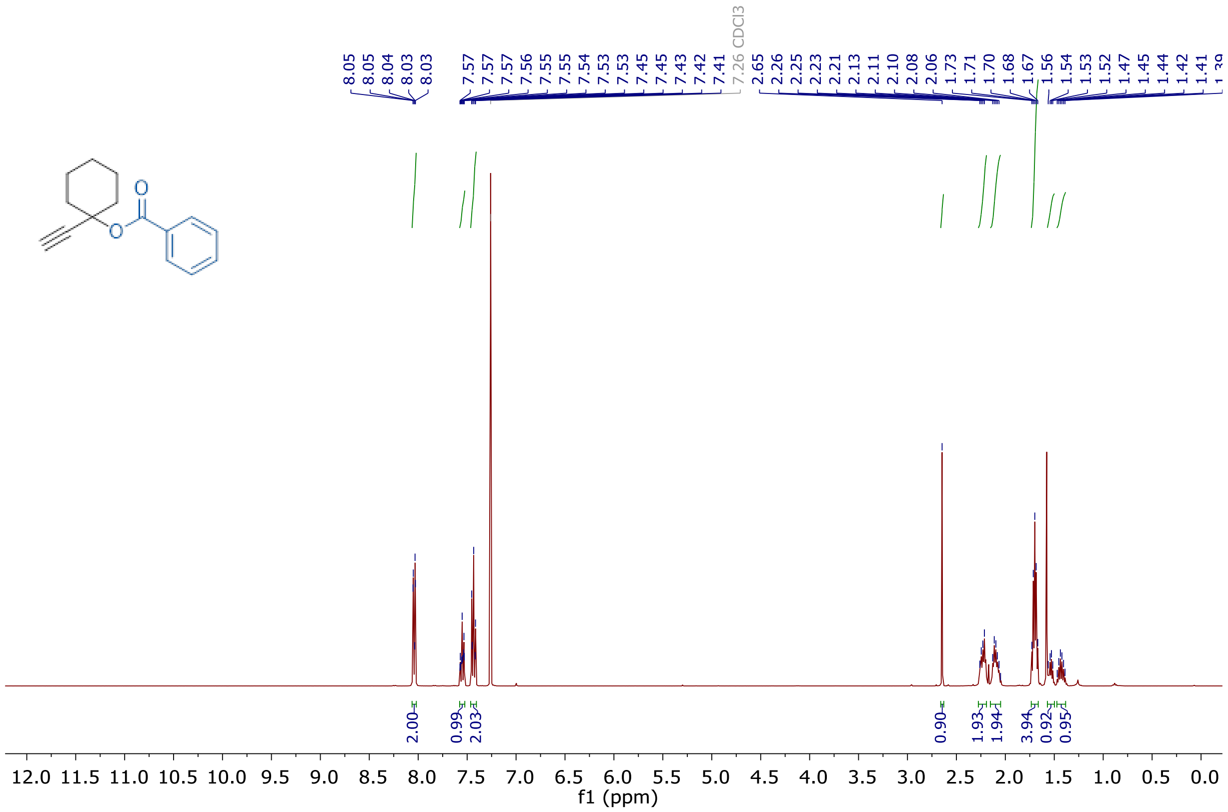

[1]**H NMR** of compound **1u** (400 MHz, $CDCl_3$)

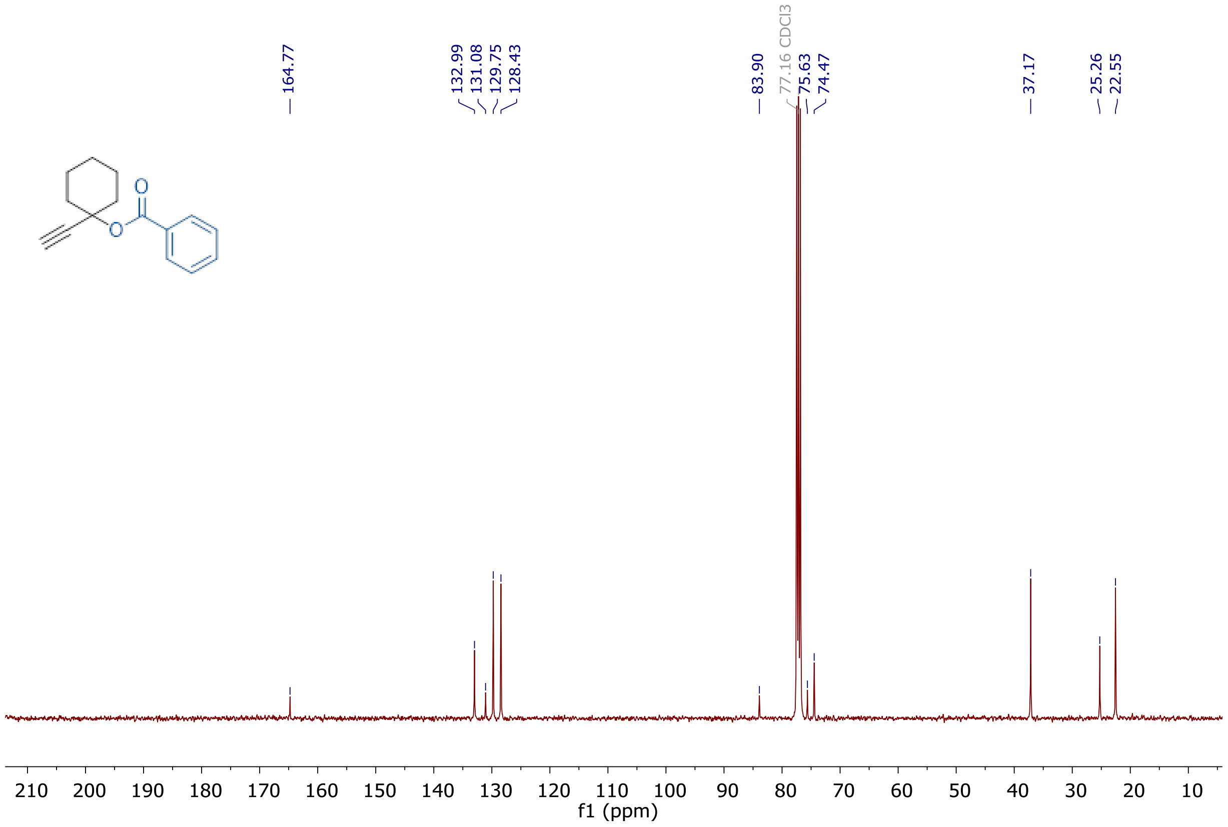

[13]**C NMR** of compound **1u** (101 MHz, $CDCl_3$)

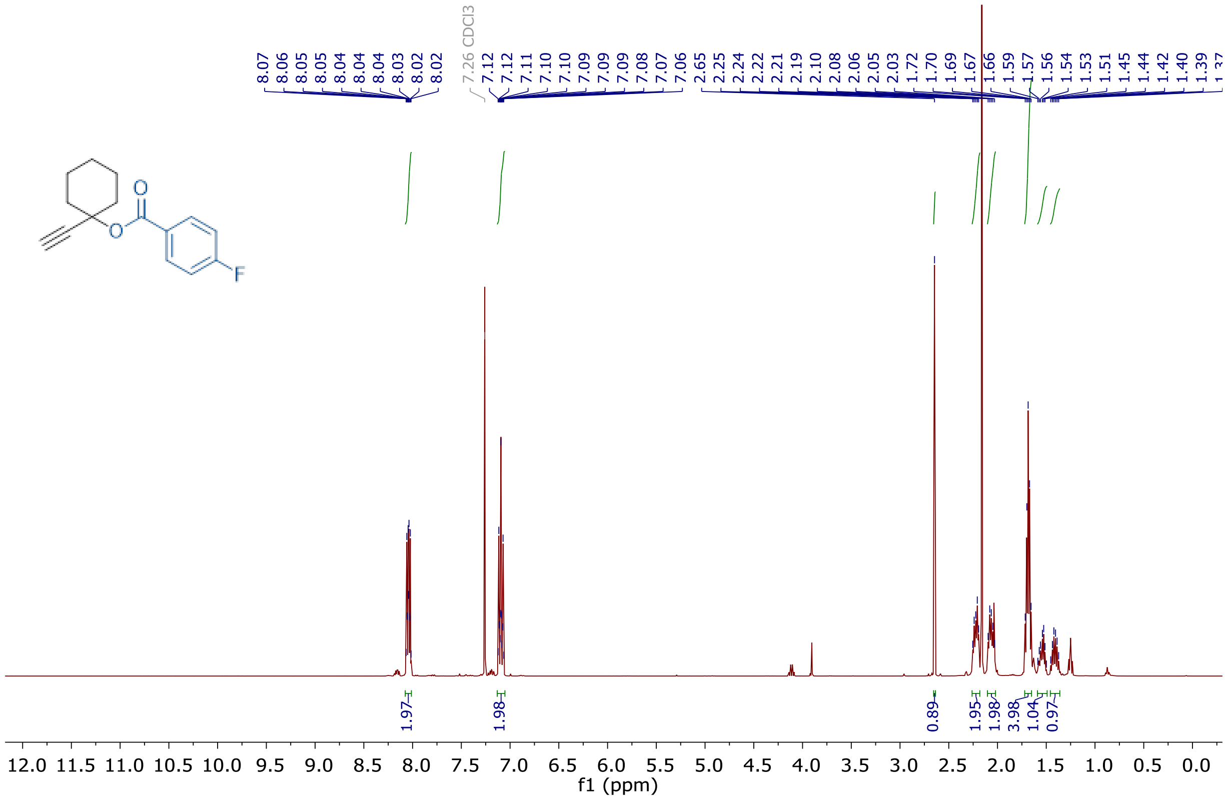

¹H NMR of compound **1v** (400 MHz, $CDCl_3$)

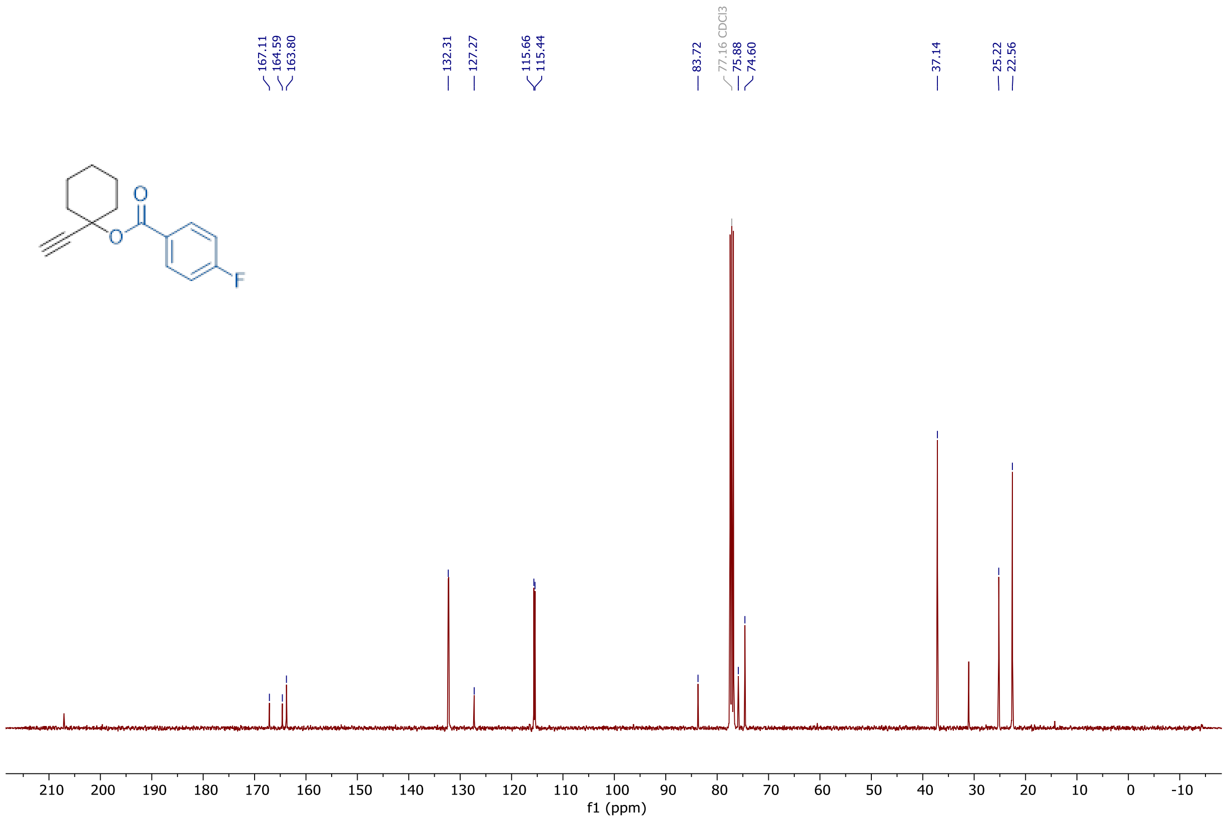

¹³C NMR of compound **1v** (101 MHz, $CDCl_3$)

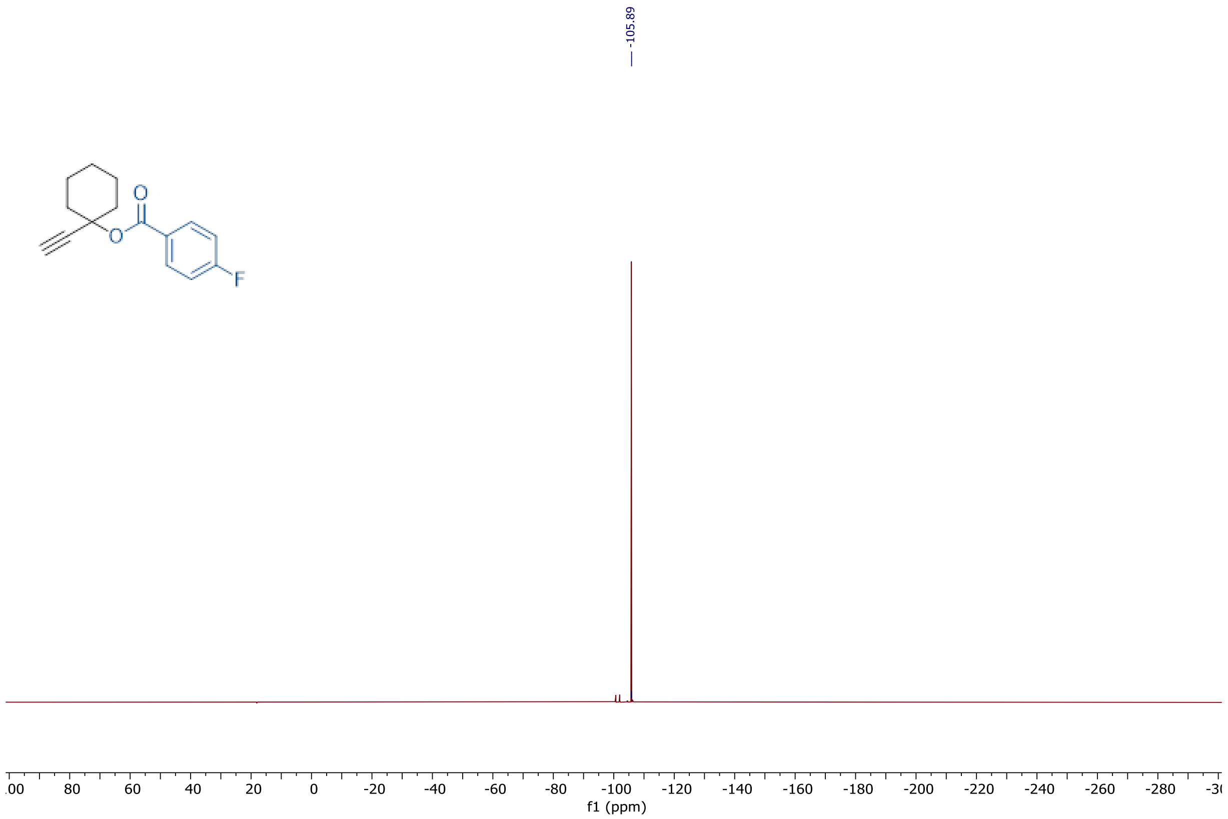

^{19}**F NMR** of compound **1v** (565 MHz, $CDCl_3$)

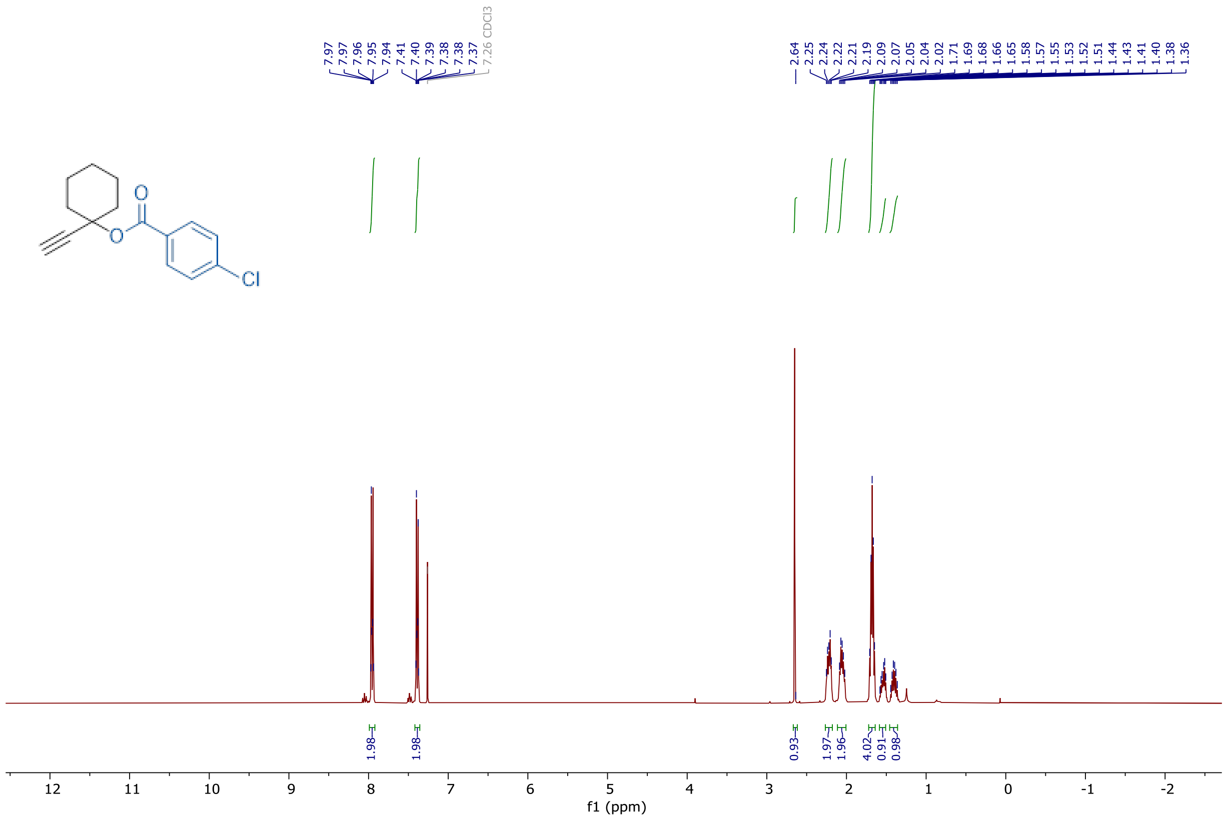

^{1}**H NMR** of compound **1w** (400 MHz, $CDCl_3$)

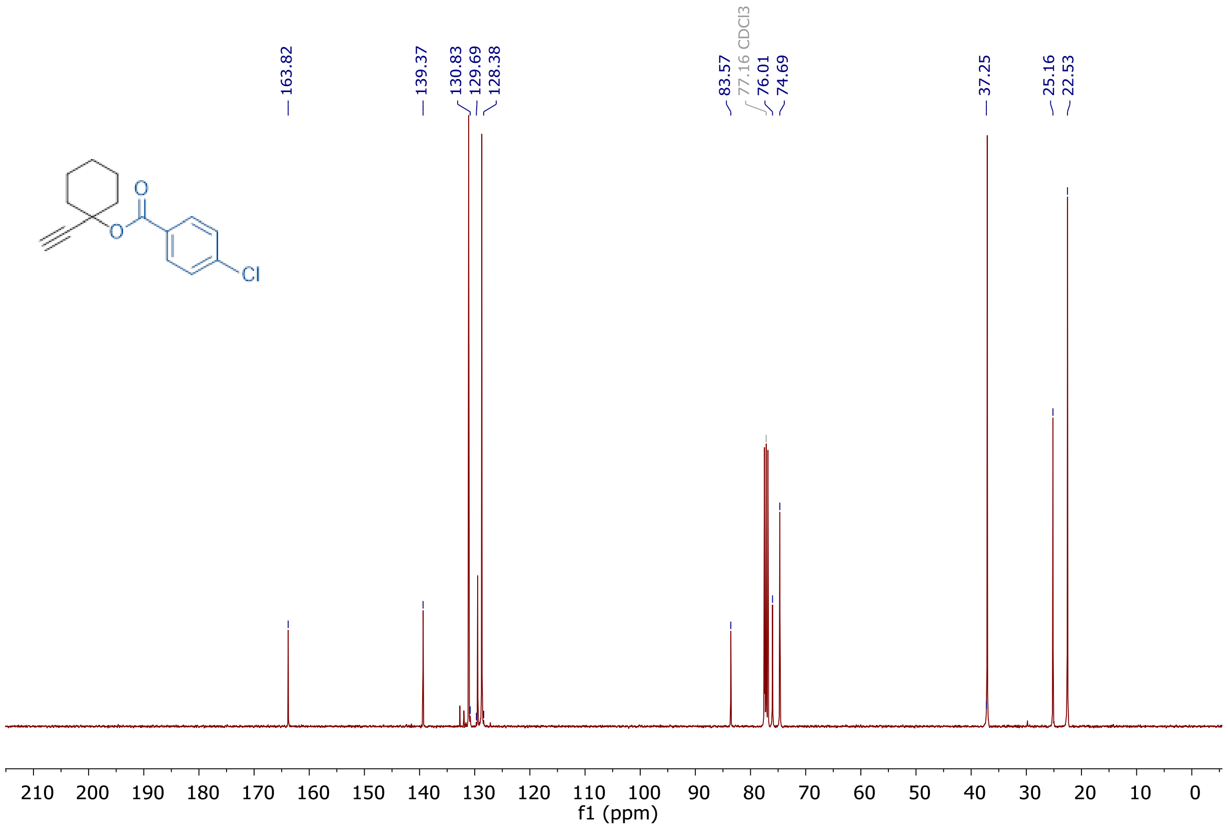

^{13}**C NMR** of compound **1w** (101 MHz, $CDCl_3$)

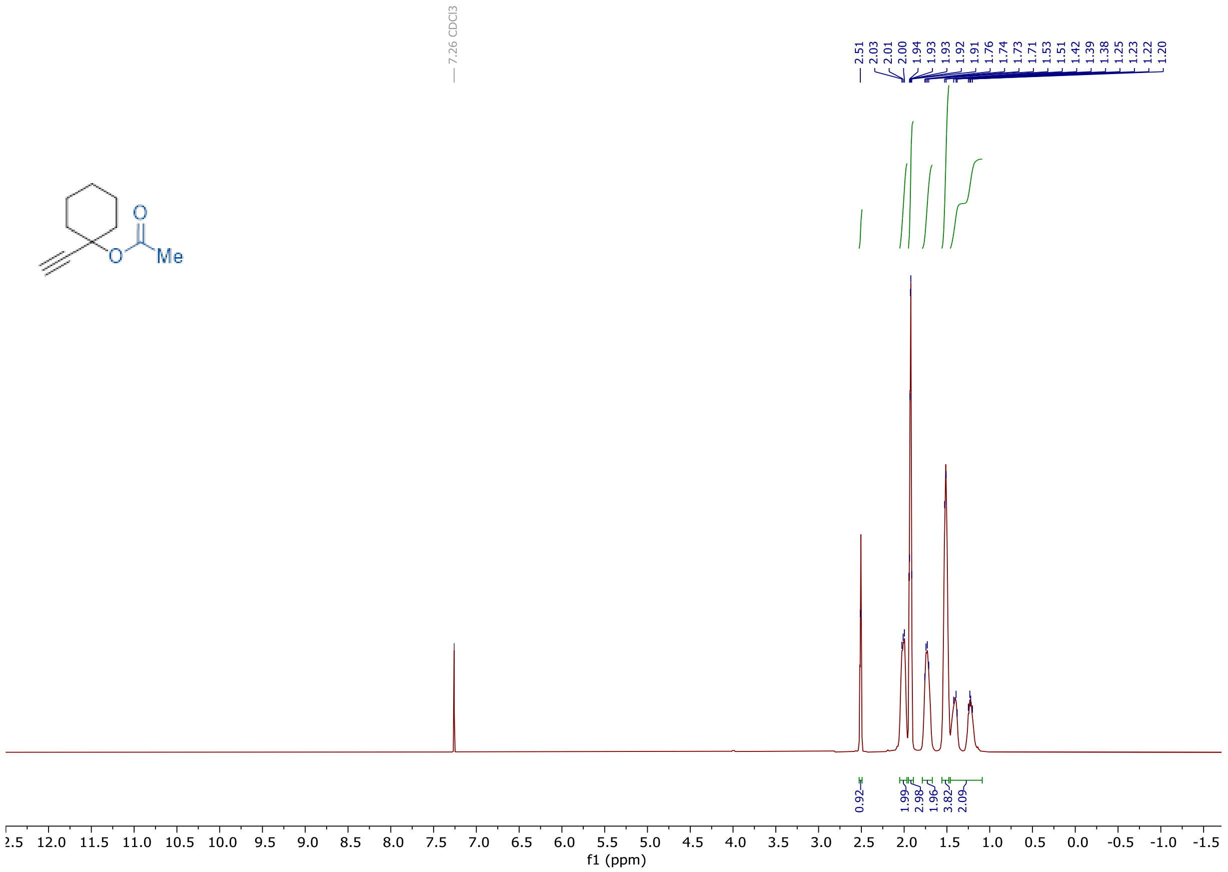

^{1}H NMR of compound **1x** (400 MHz, $CDCl_3$)

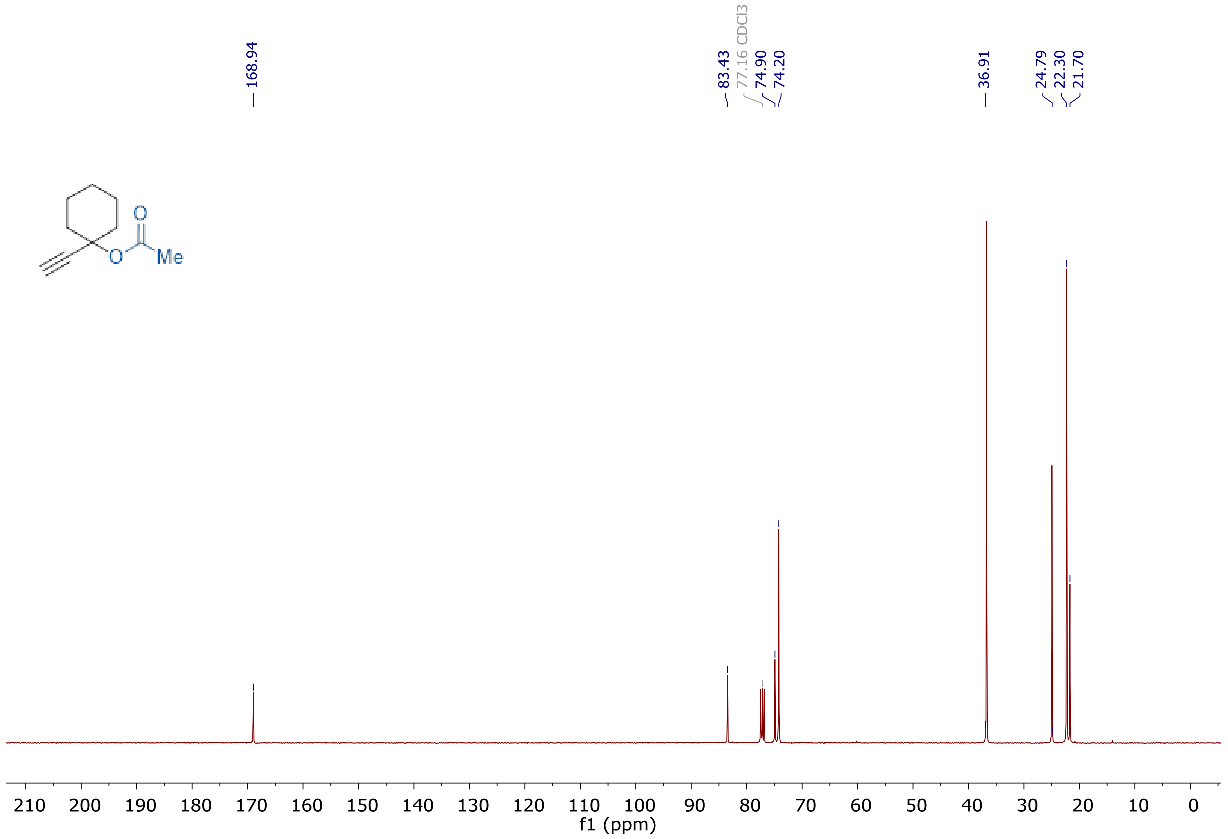

^{13}C NMR of compound **1x** (101 MHz, $CDCl_3$)

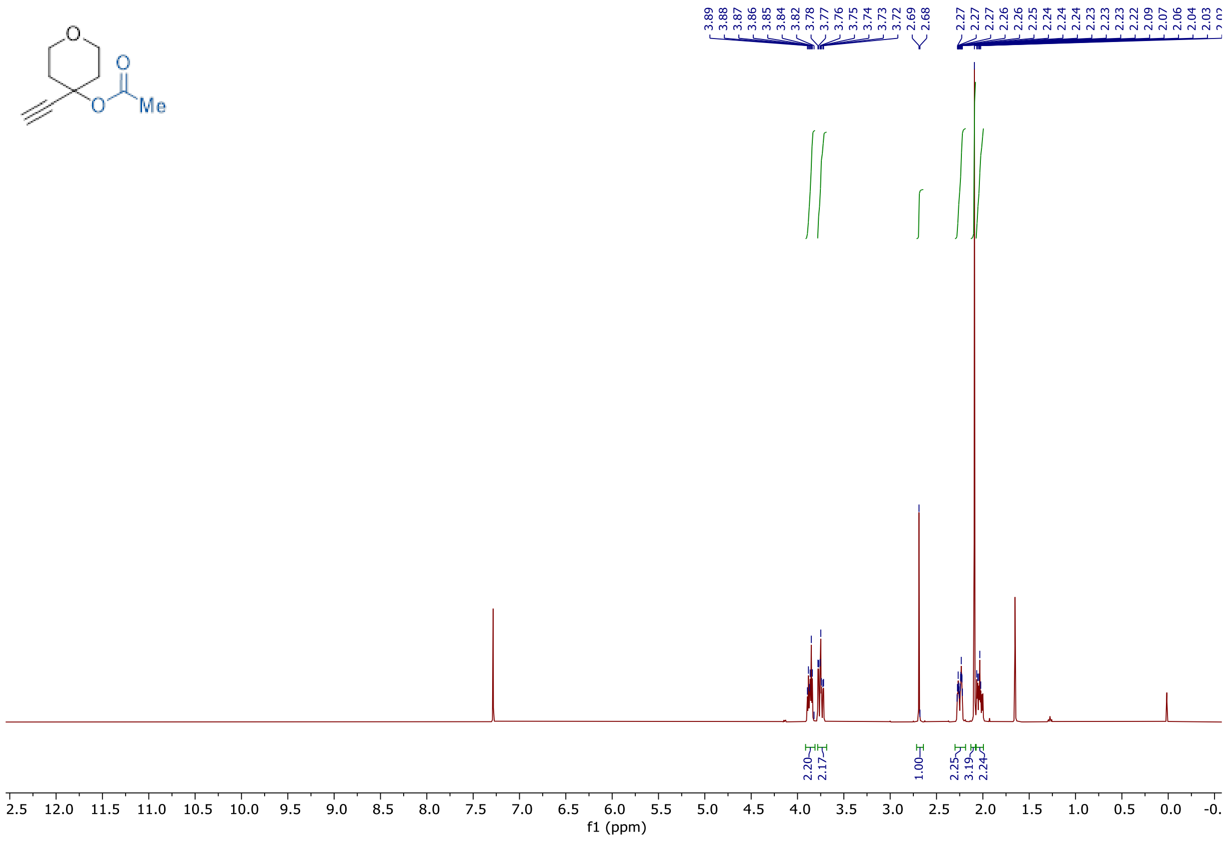

^{1}**H NMR** of compound **1y** (400 MHz, $CDCl_3$)

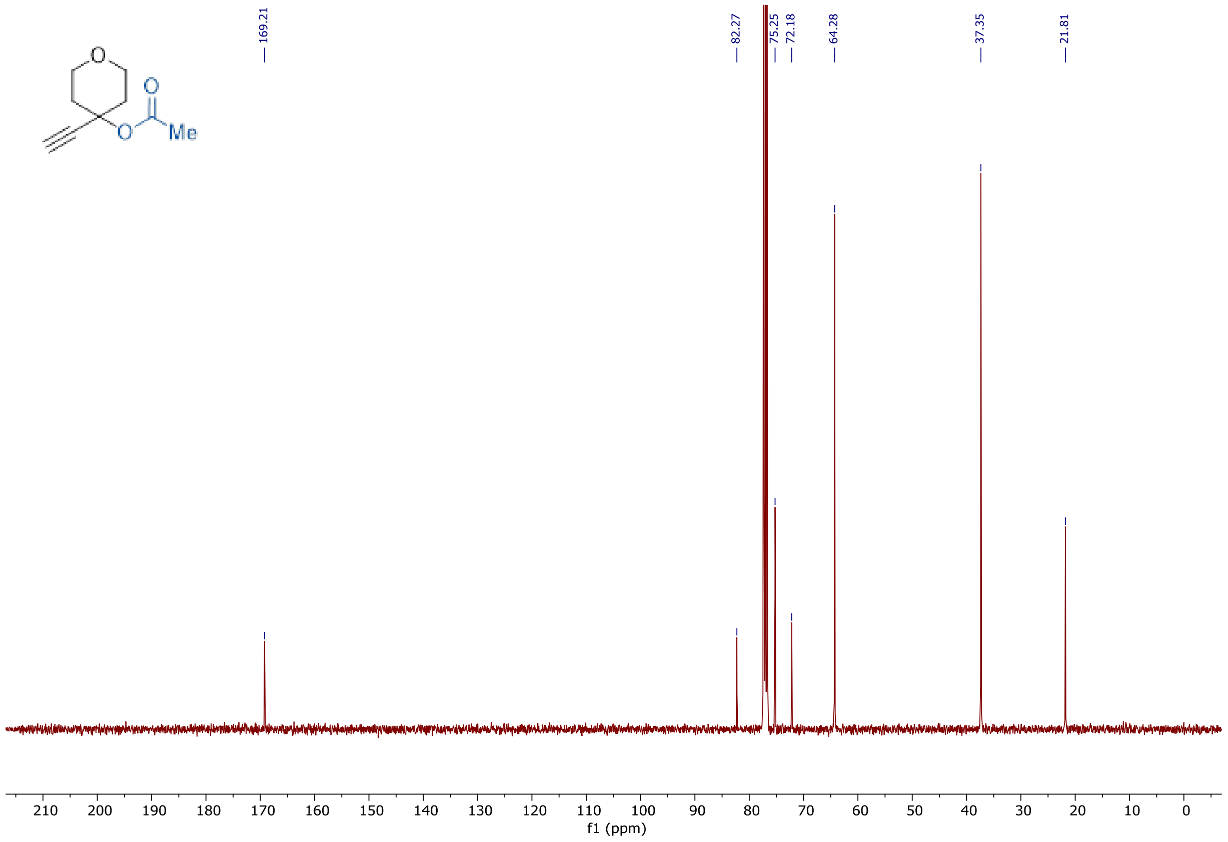

^{13}**C NMR** of compound **1y** (101 MHz, $CDCl_3$)

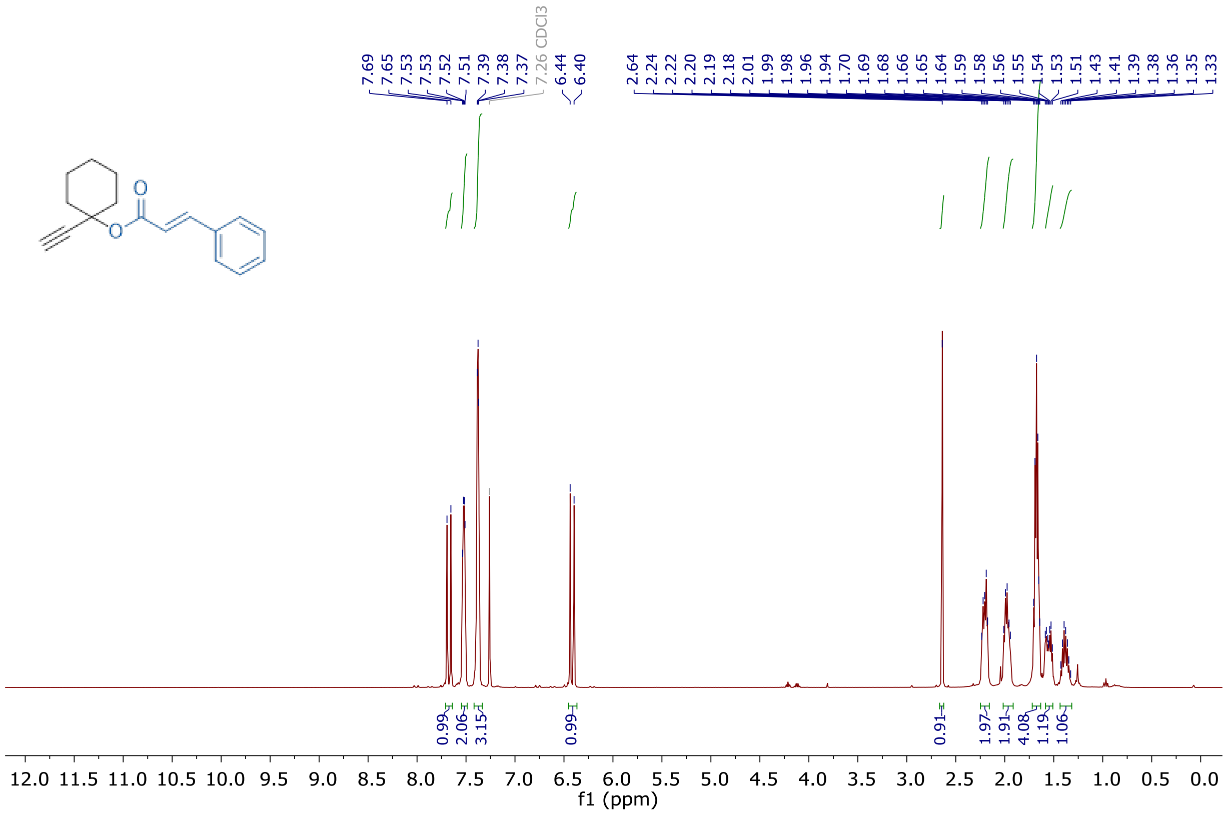

^{1}H NMR of compound **1z** (400 MHz, $CDCl_3$)

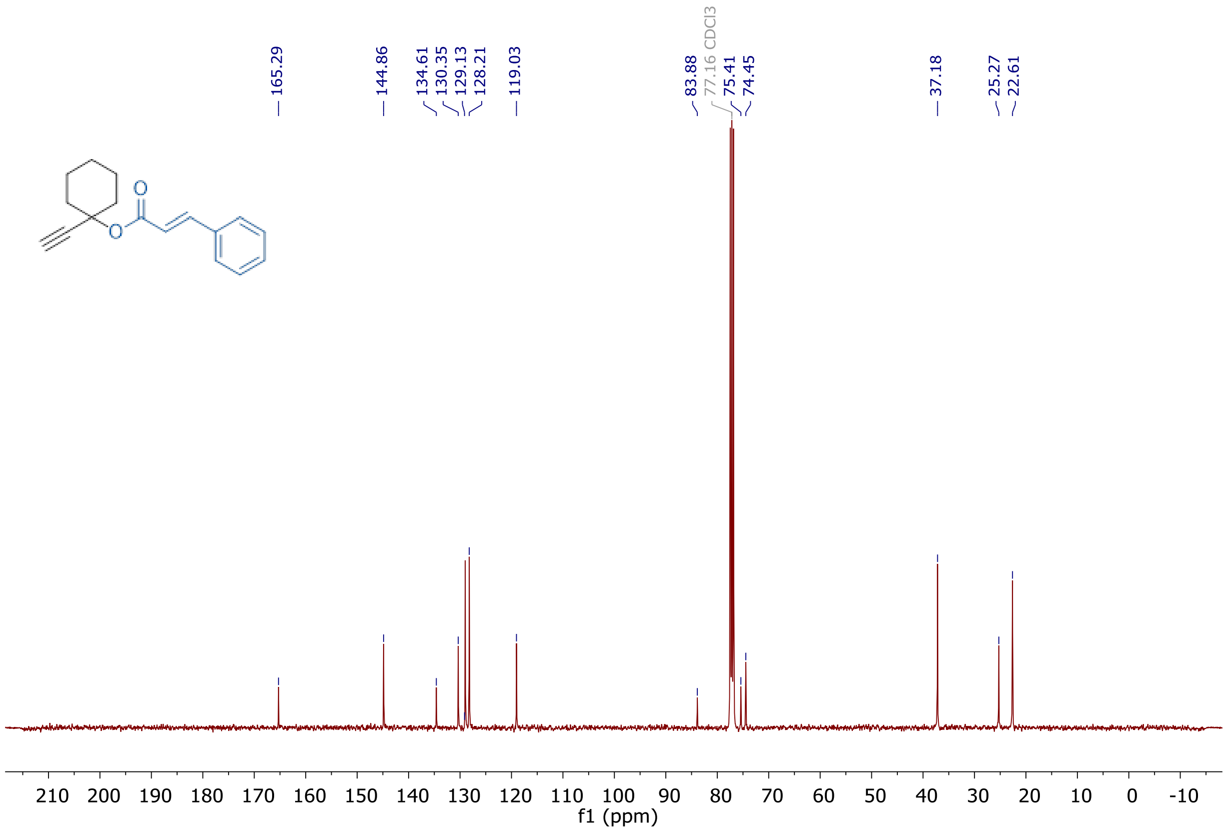

^{13}C NMR of compound **1z** (101 MHz, $CDCl_3$)

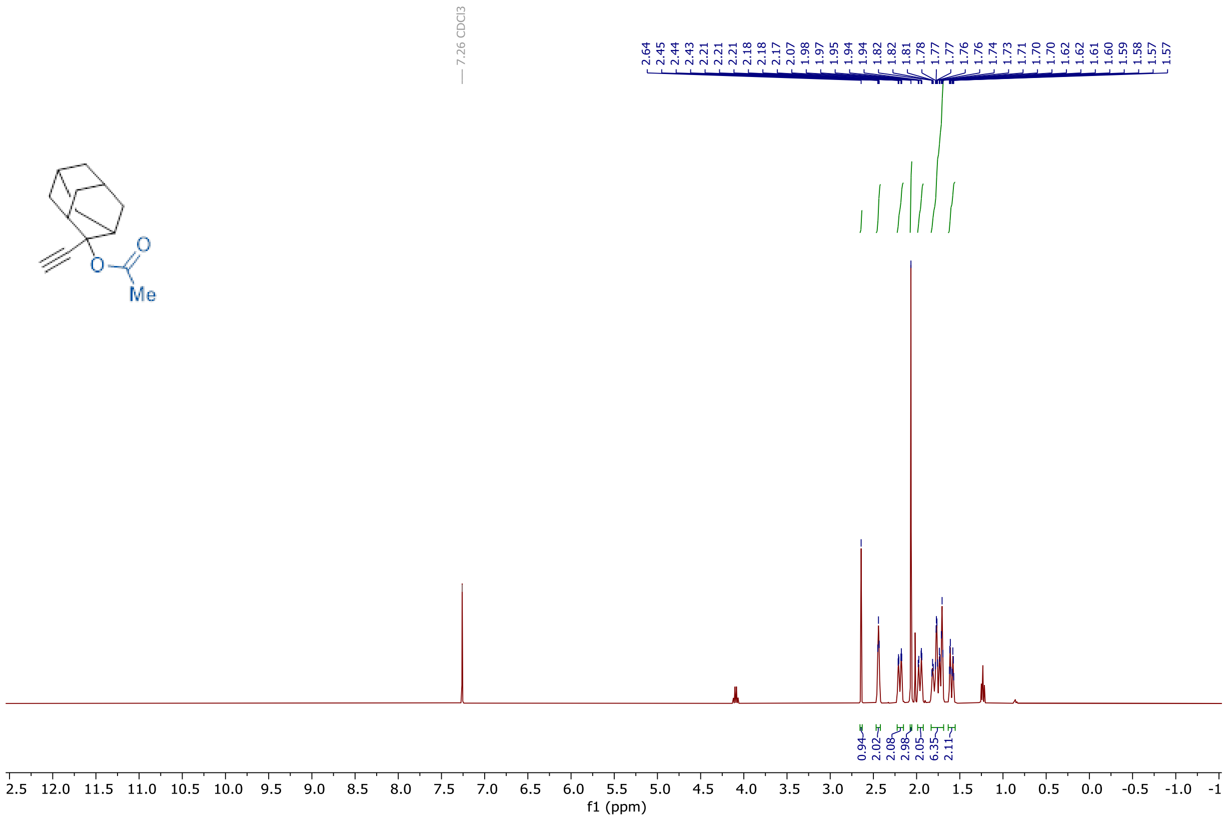

[1]**H NMR** of compound **1aa** (400 MHz, $CDCl_3$)

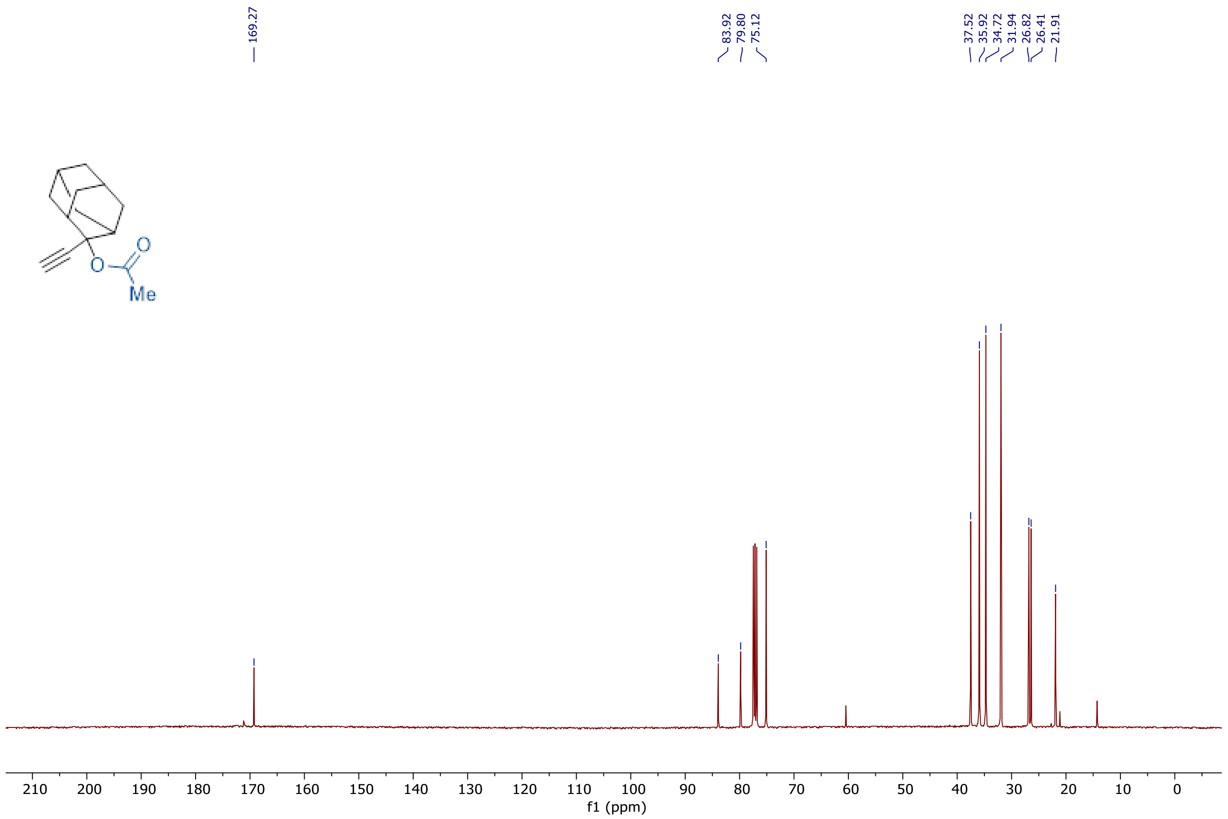

[13]**C NMR** of compound **1aa** (101 MHz, $CDCl_3$)

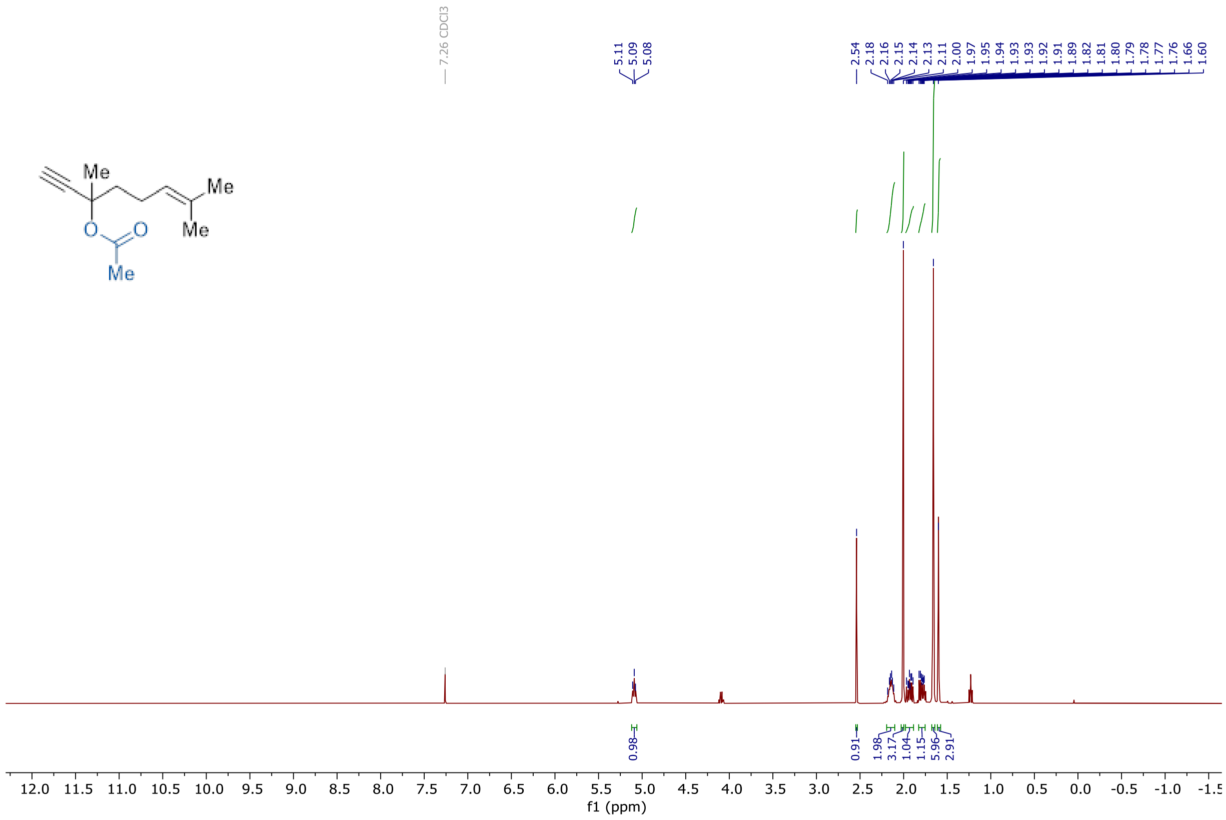

^{1}H NMR of compound **1ab** (400 MHz, $CDCl_3$)

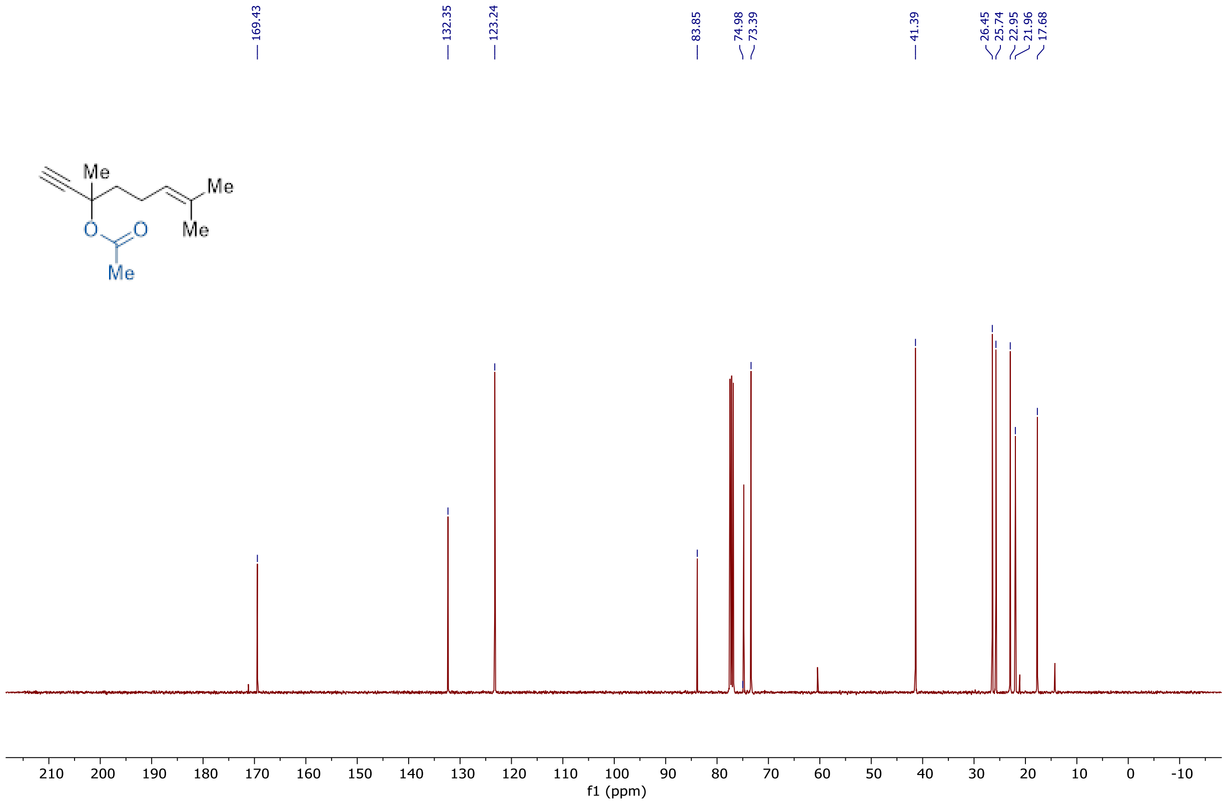

^{13}C NMR of compound **1ac** (101 MHz, $CDCl_3$)

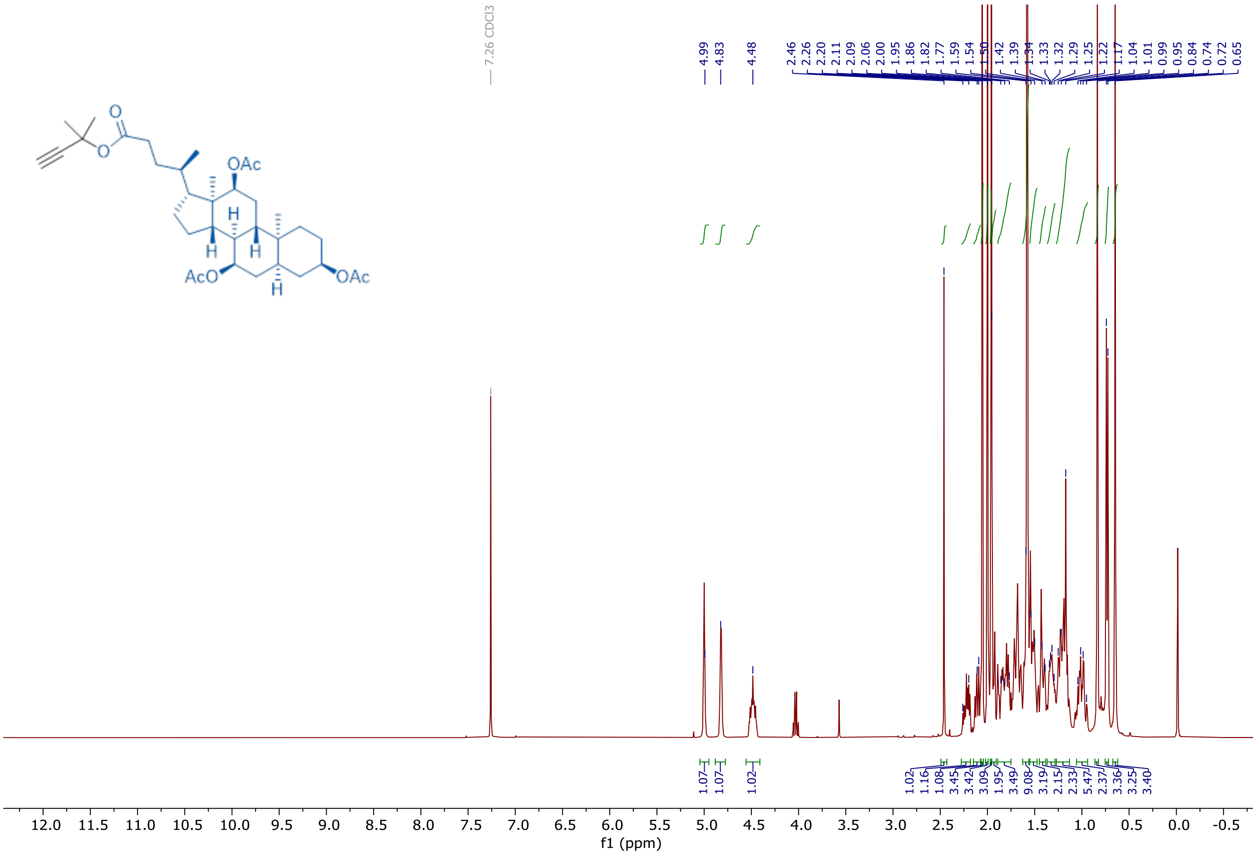

^{1}H NMR of compound **1ac** (400 MHz, $CDCl_3$)

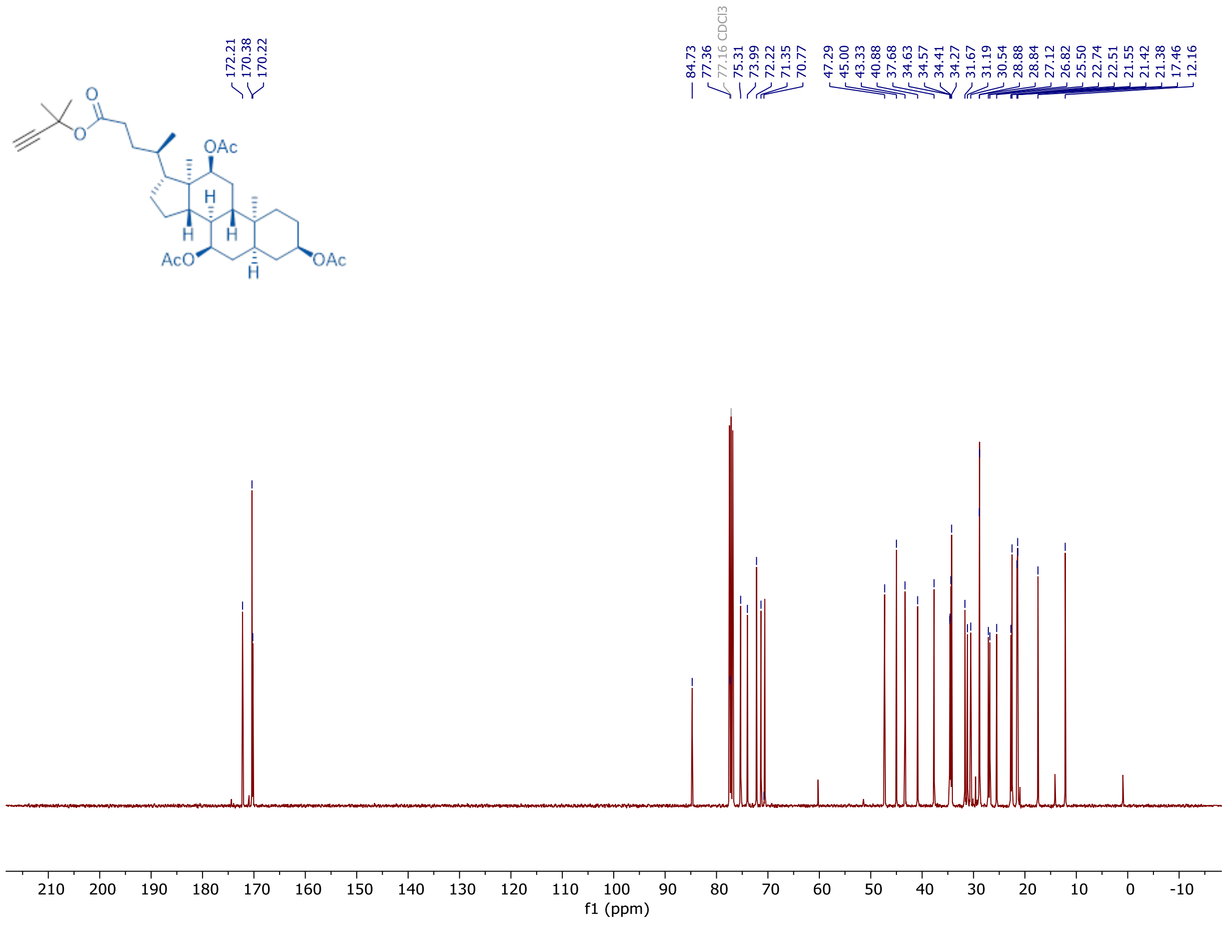

^{13}C NMR of compound **1ac** (101 MHz, $CDCl_3$)

Products **2a-ac**

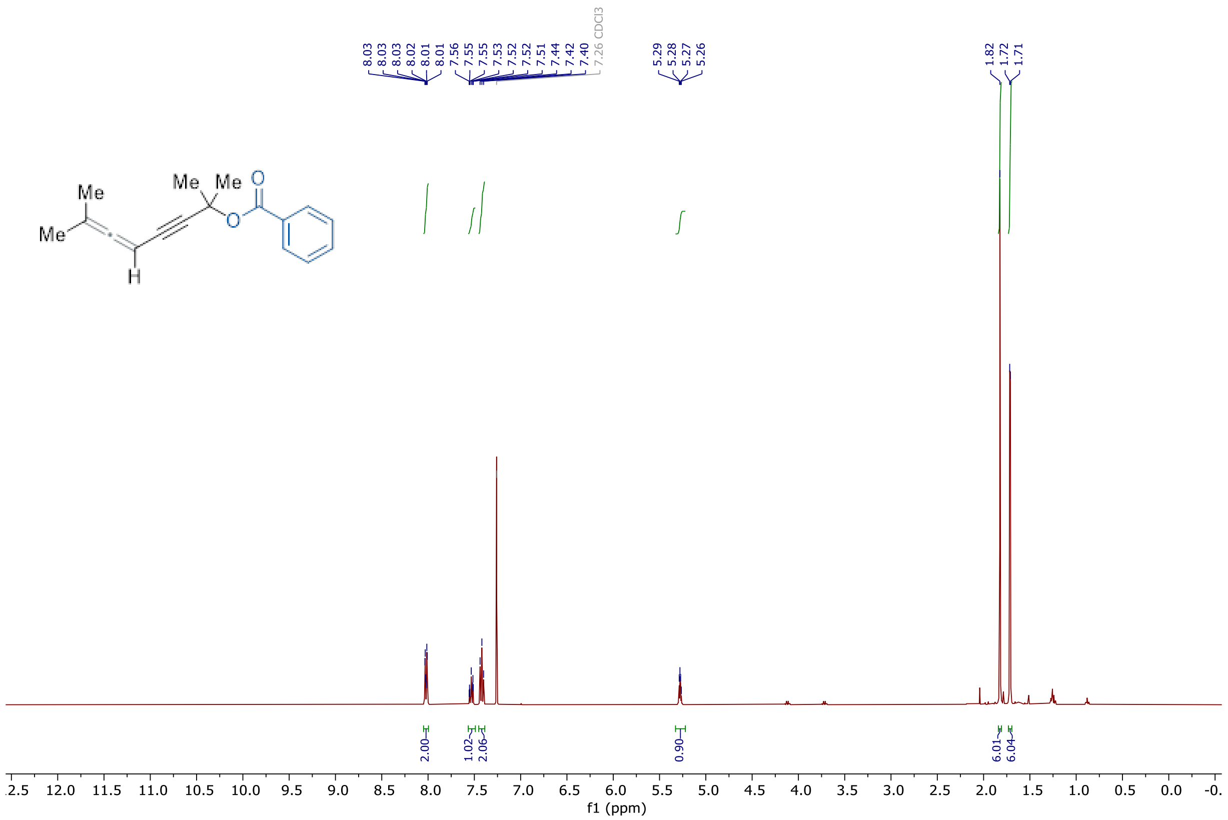

^{1}H NMR of compound **2a** (400 MHz, $CDCl_3$)

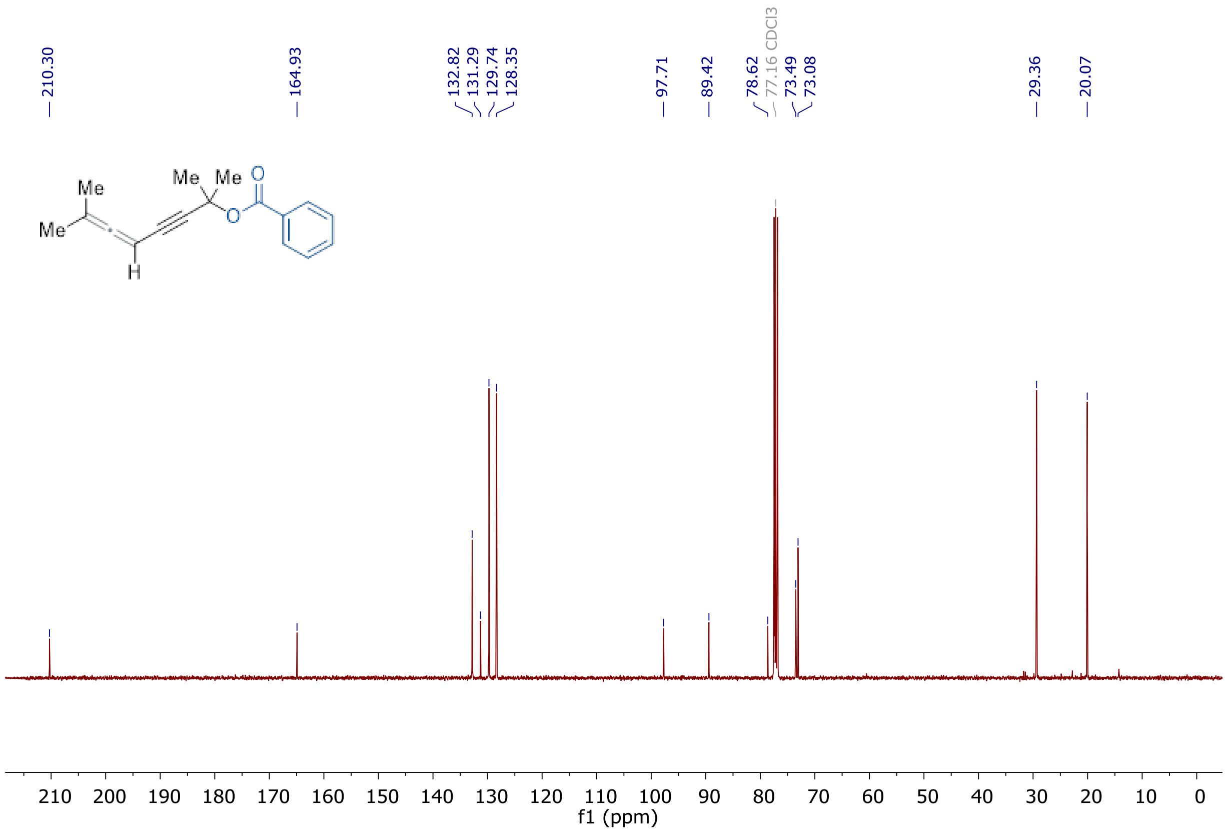

^{13}C NMR of compound **2a** (101 MHz, $CDCl_3$)

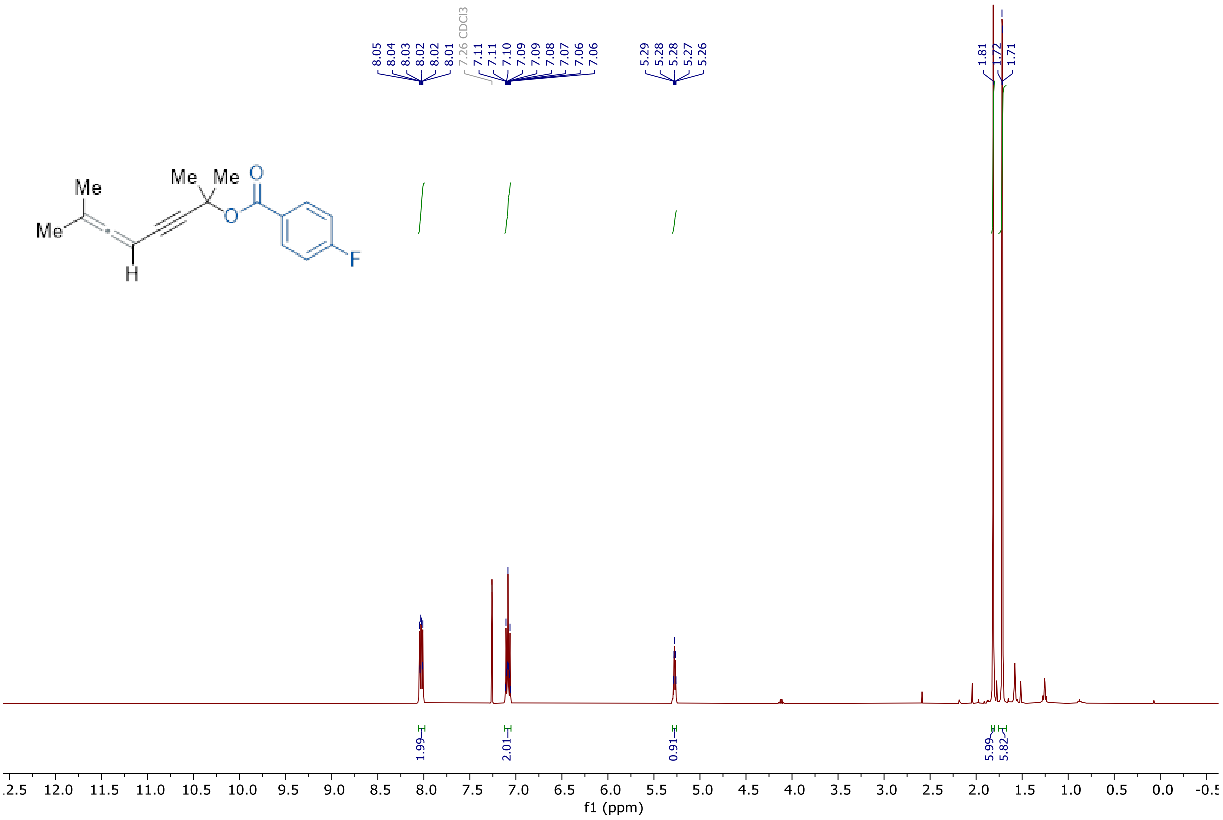

^{1}**H NMR** of compound **2b** (400 MHz, $CDCl_3$)

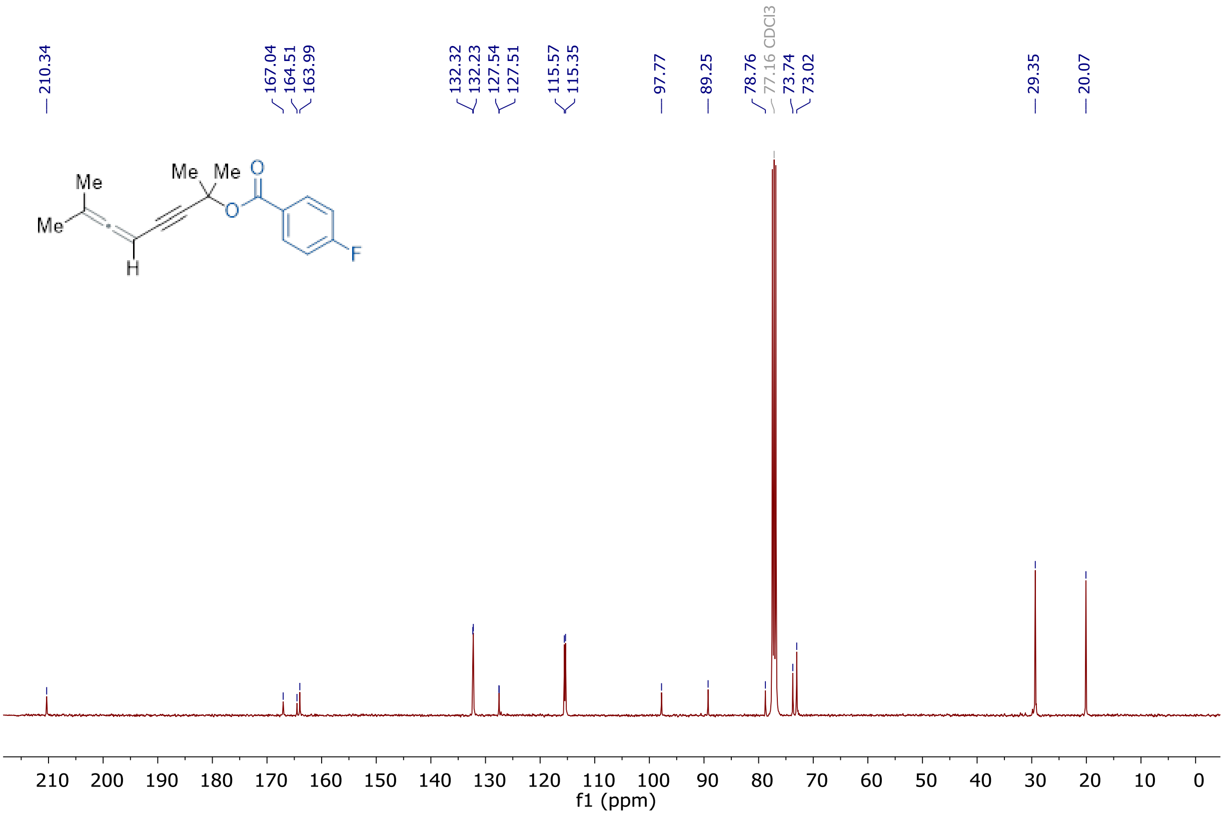

^{13}**C NMR** of compound **2b** (101 MHz, $CDCl_3$)

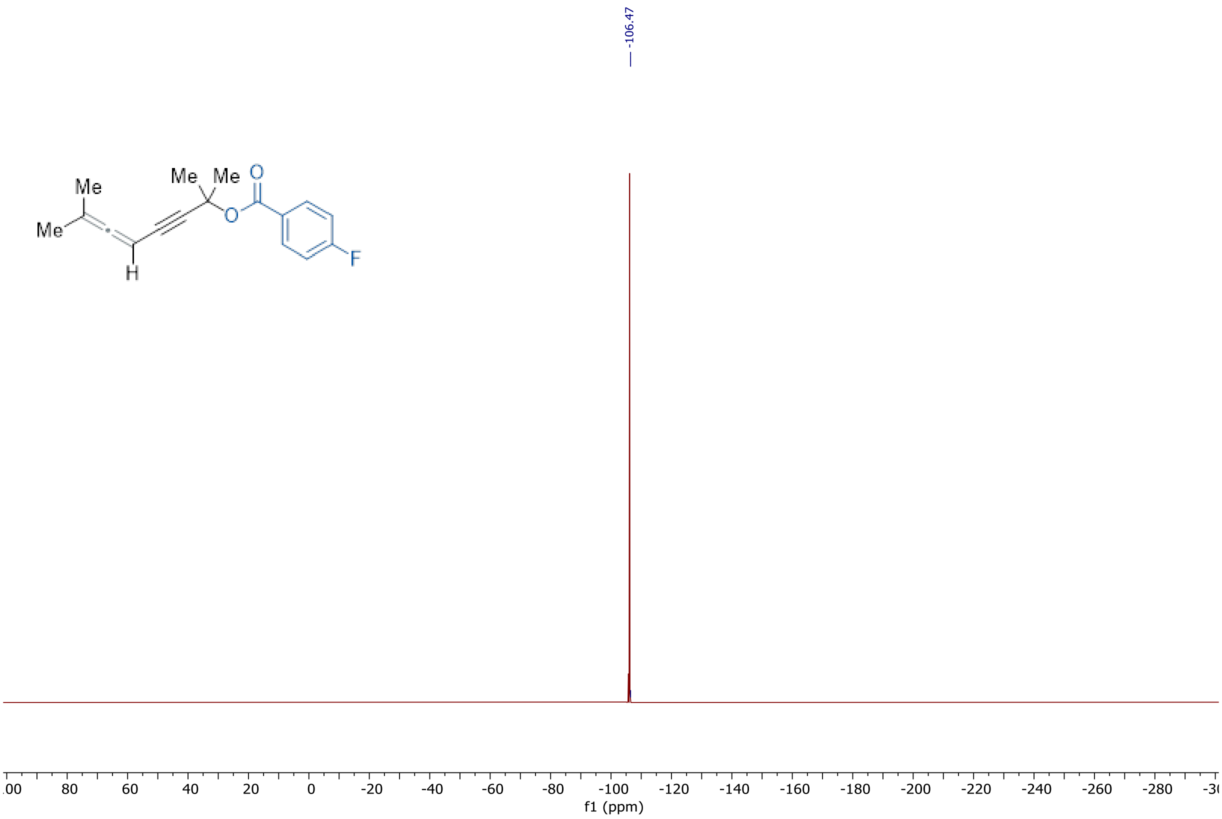

^{19}**F NMR** of compound **2b** (565 MHz, $CDCl_3$)

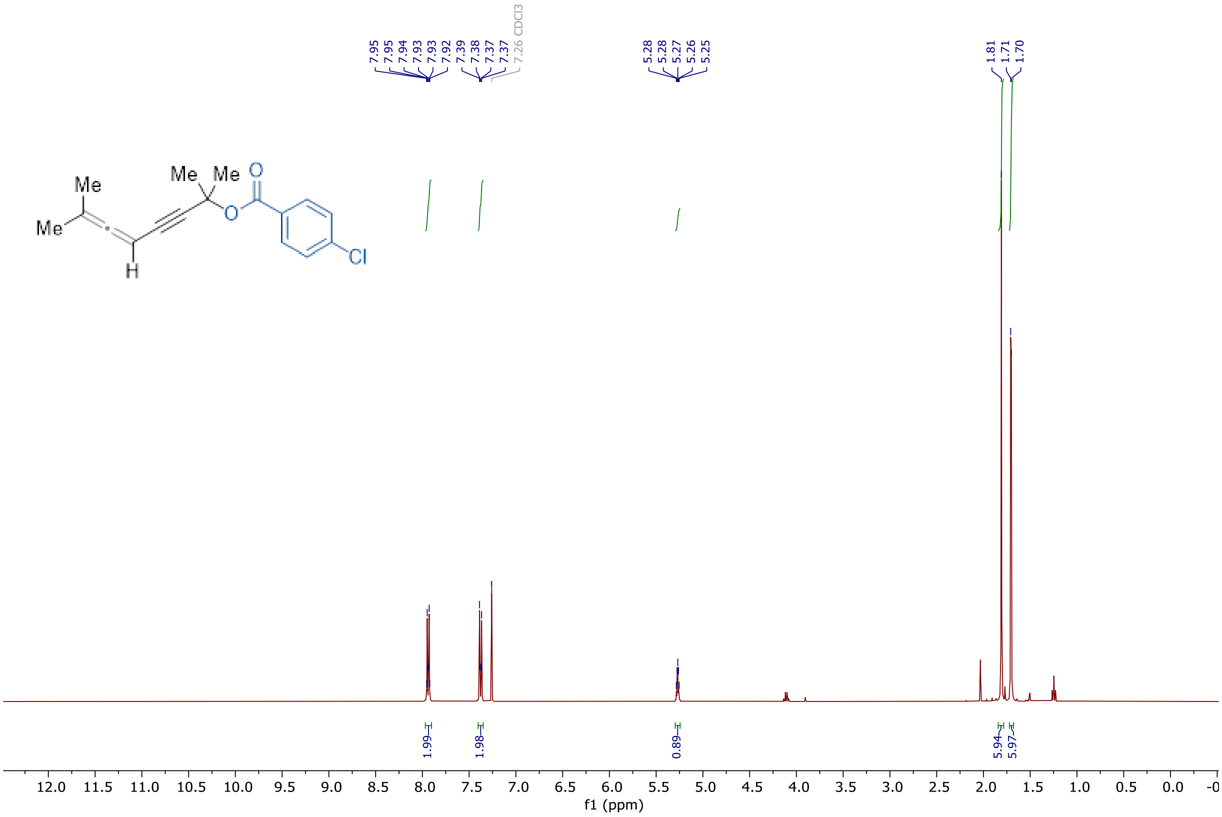

^{1}H NMR of compound **2c** (400 MHz, $CDCl_3$)

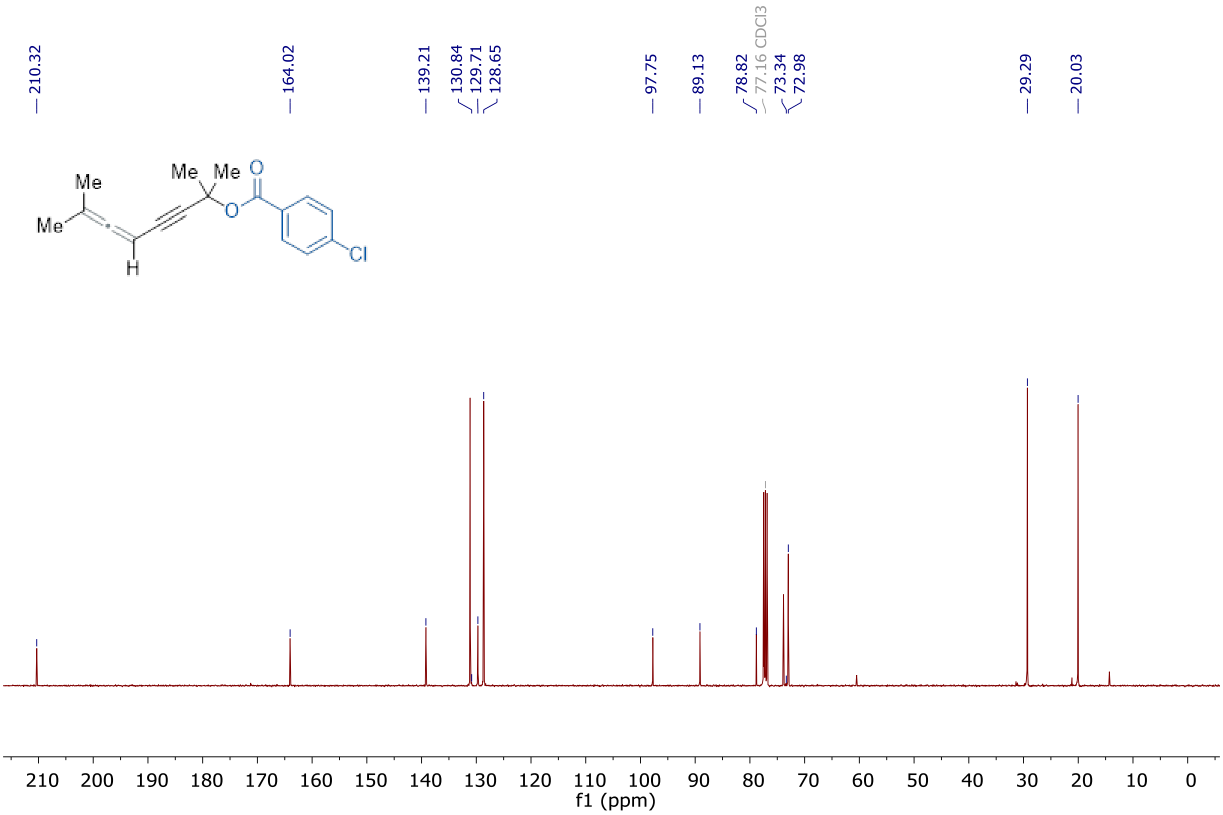

^{13}C NMR of compound **2c** (101 MHz, $CDCl_3$)

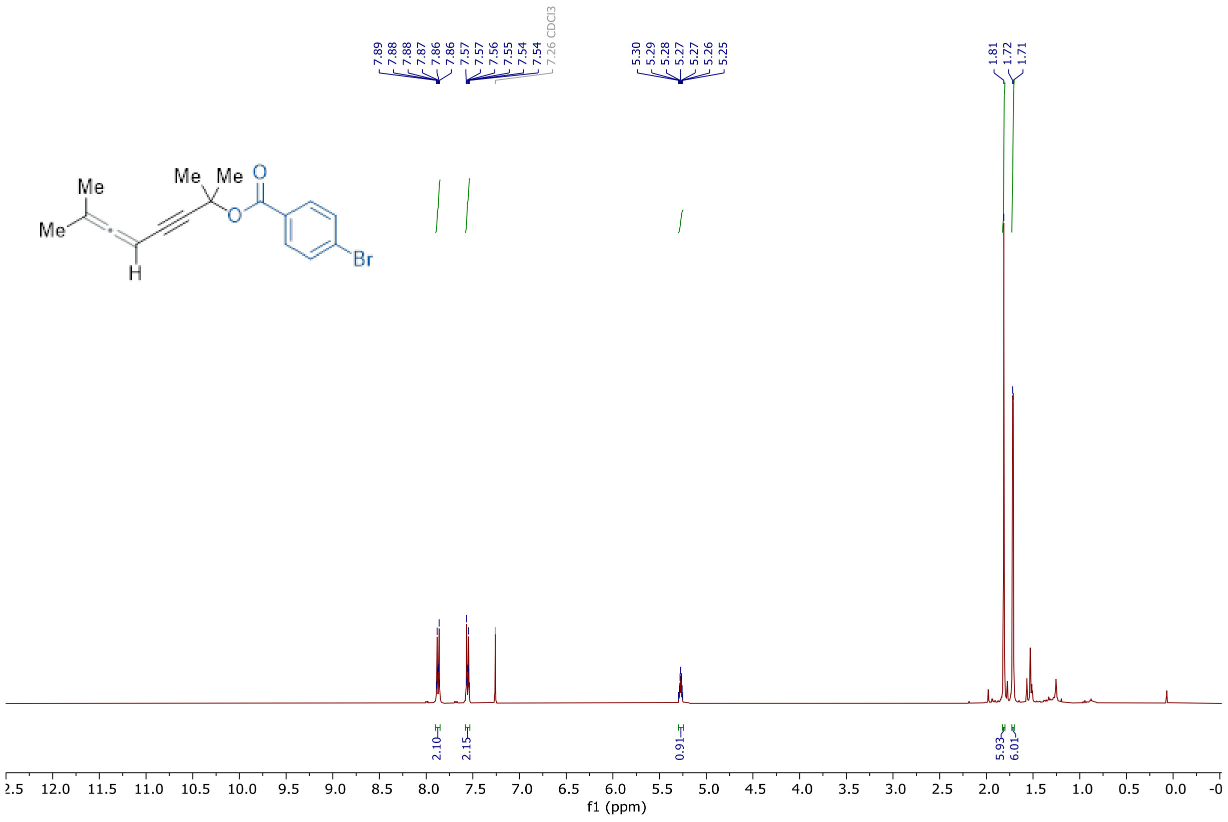

^{1}**H NMR** of compound **2d** (400 MHz, $CDCl_3$)

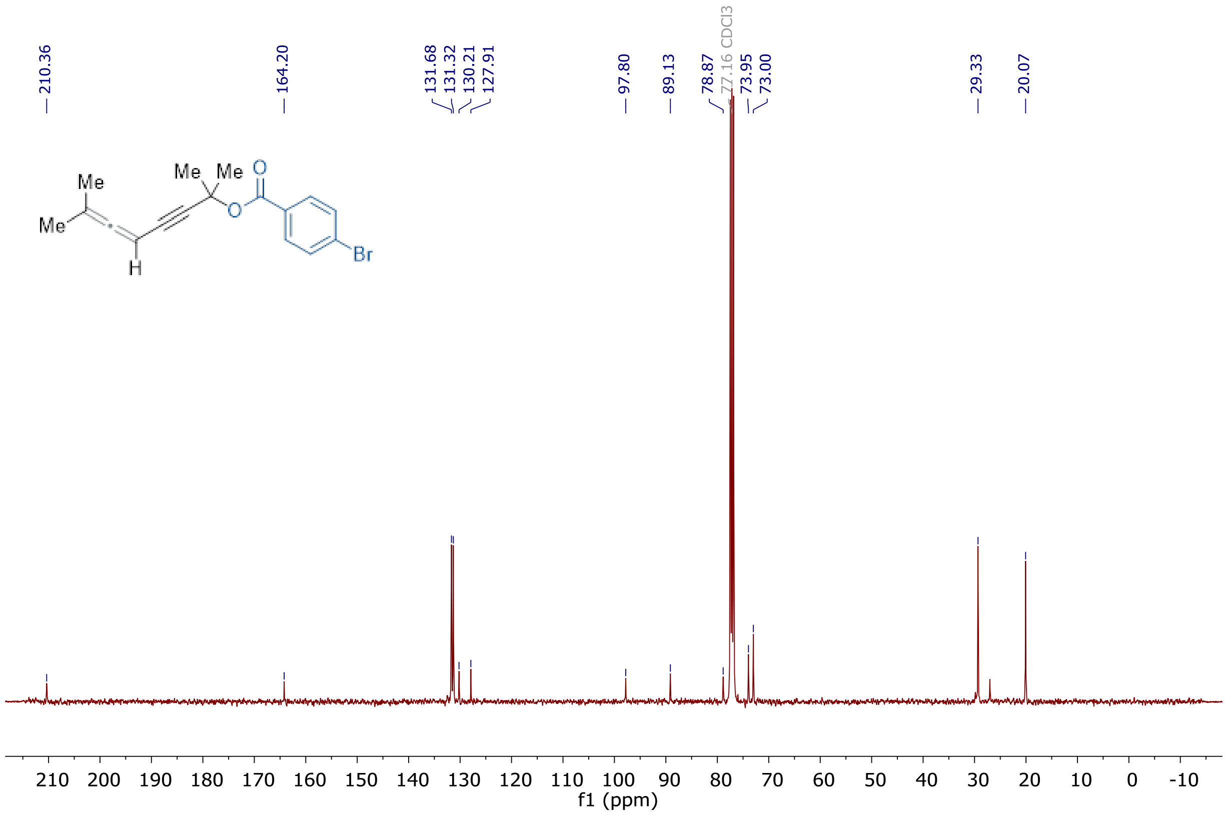

^{13}**C NMR** of compound **2d** (101 MHz, $CDCl_3$)

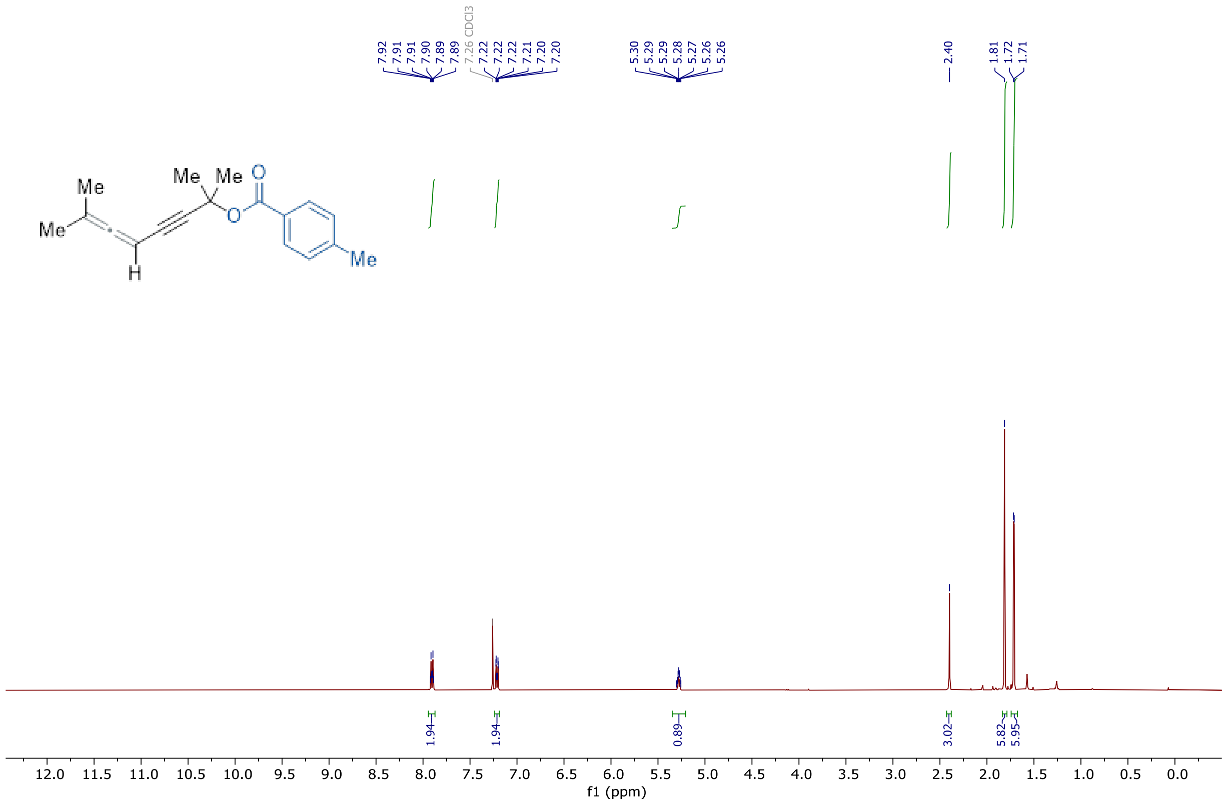

[1]**H NMR** of compound **2e** (400 MHz, $CDCl_3$)

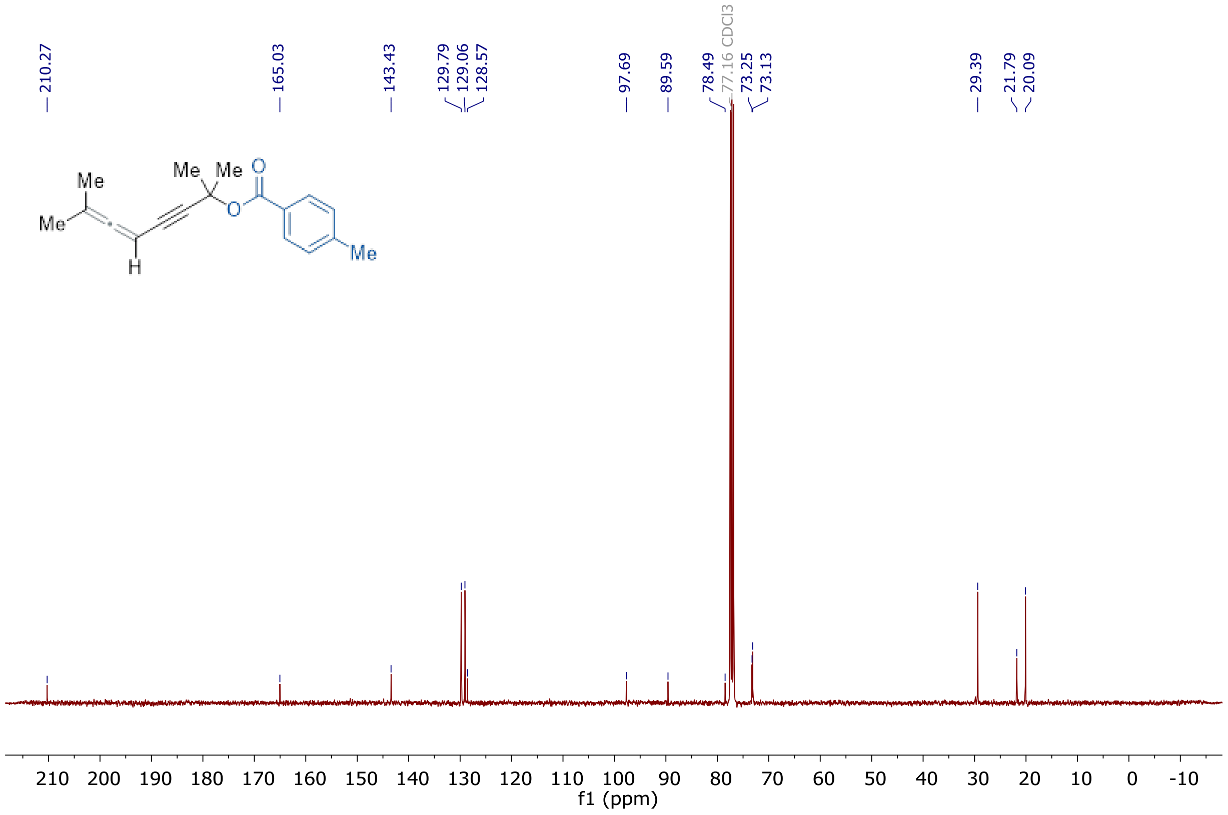

[13]**C NMR** of compound **2e** (101 MHz, $CDCl_3$)

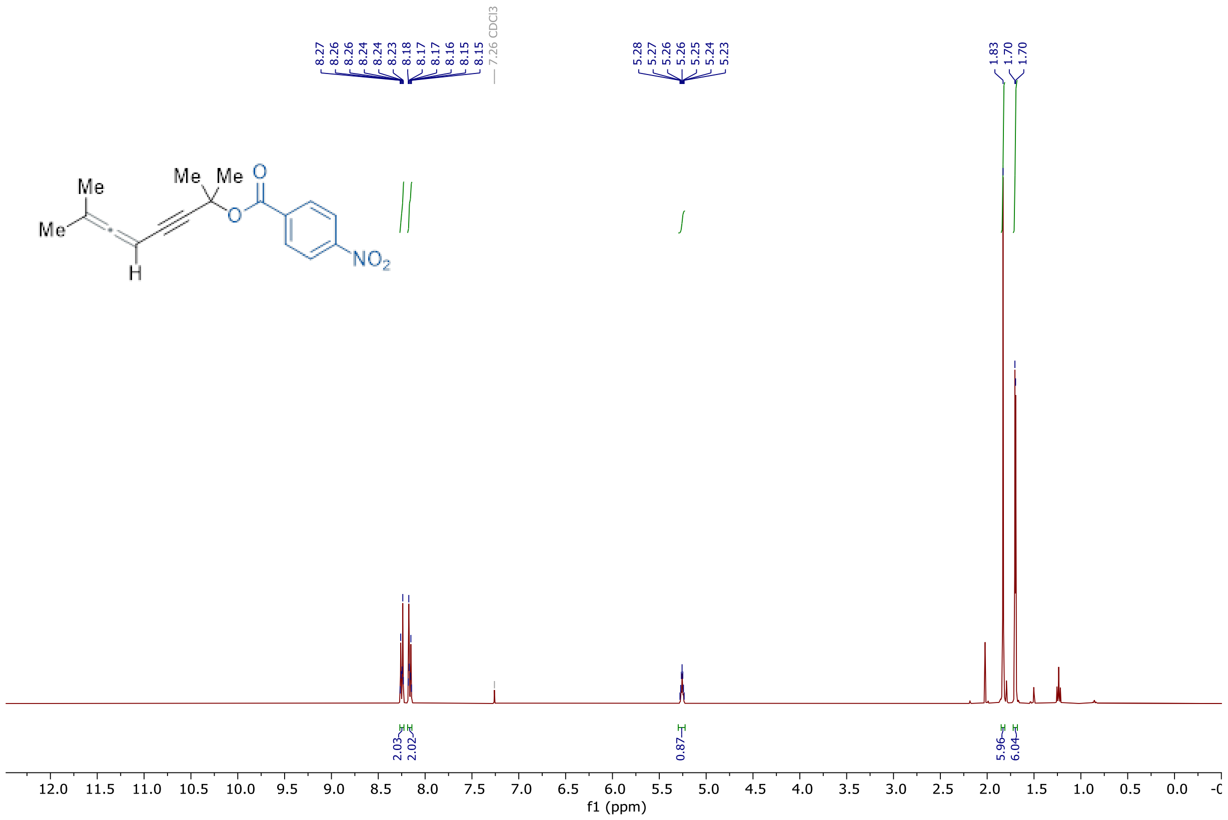

^{1}**H NMR** of compound **2f** (400 MHz, $CDCl_3$)

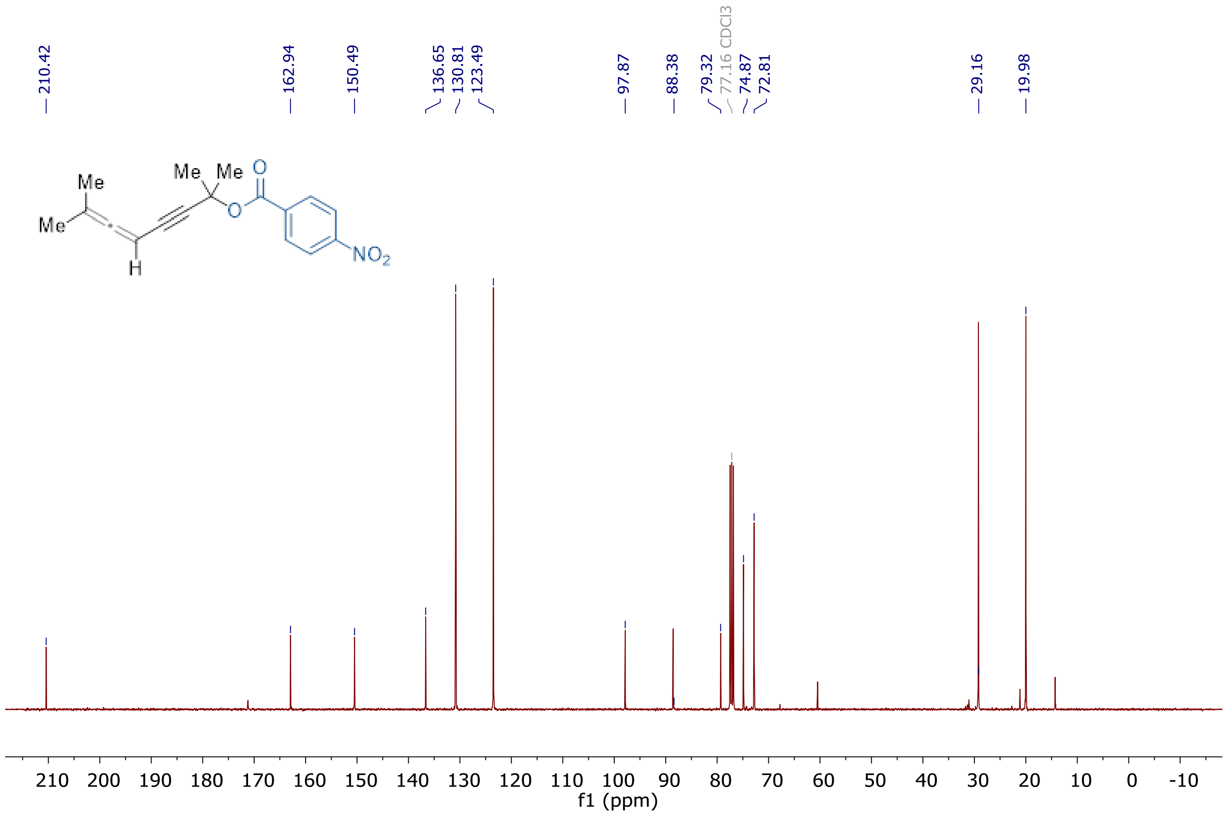

^{13}**C NMR** of compound **2f** (101 MHz, $CDCl_3$)

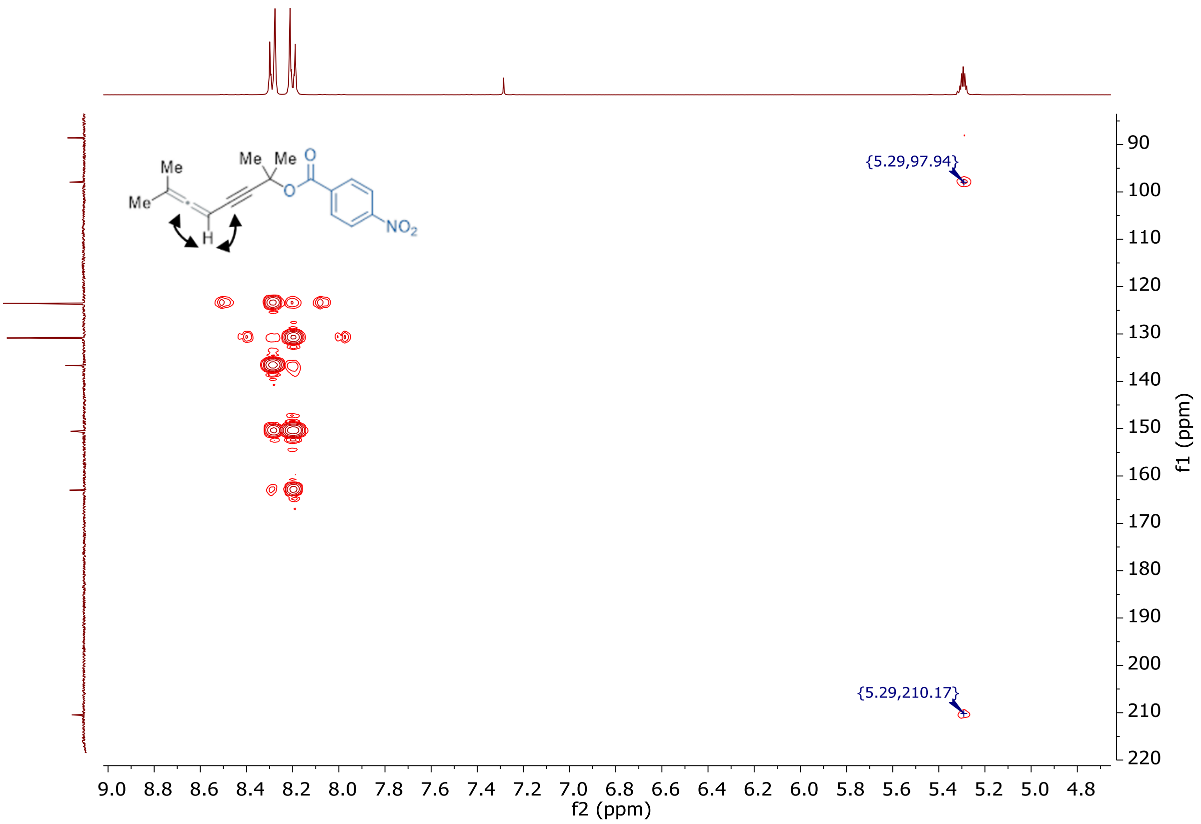

HMBC NMR of compound **2f** (101 MHz, $CDCl_3$)

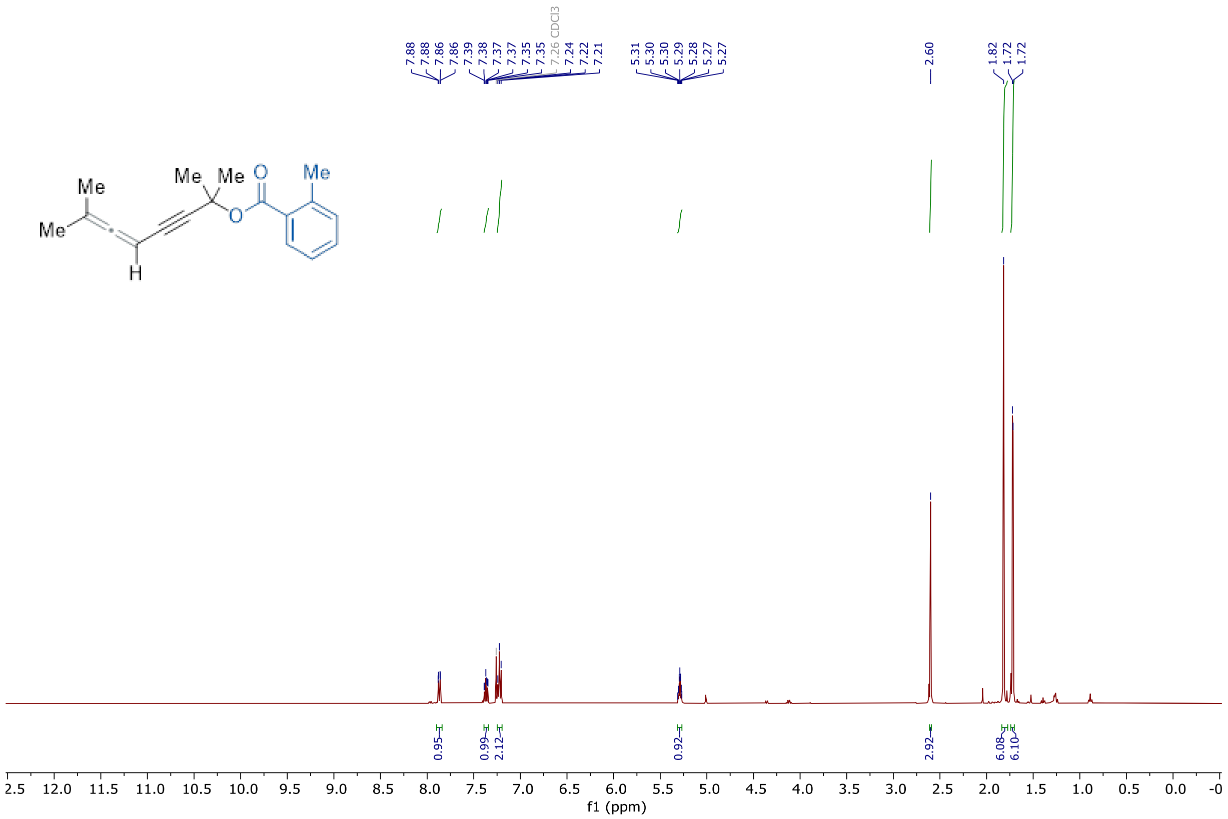

[1]**H NMR** of compound **2g** (400 MHz, $CDCl_3$)

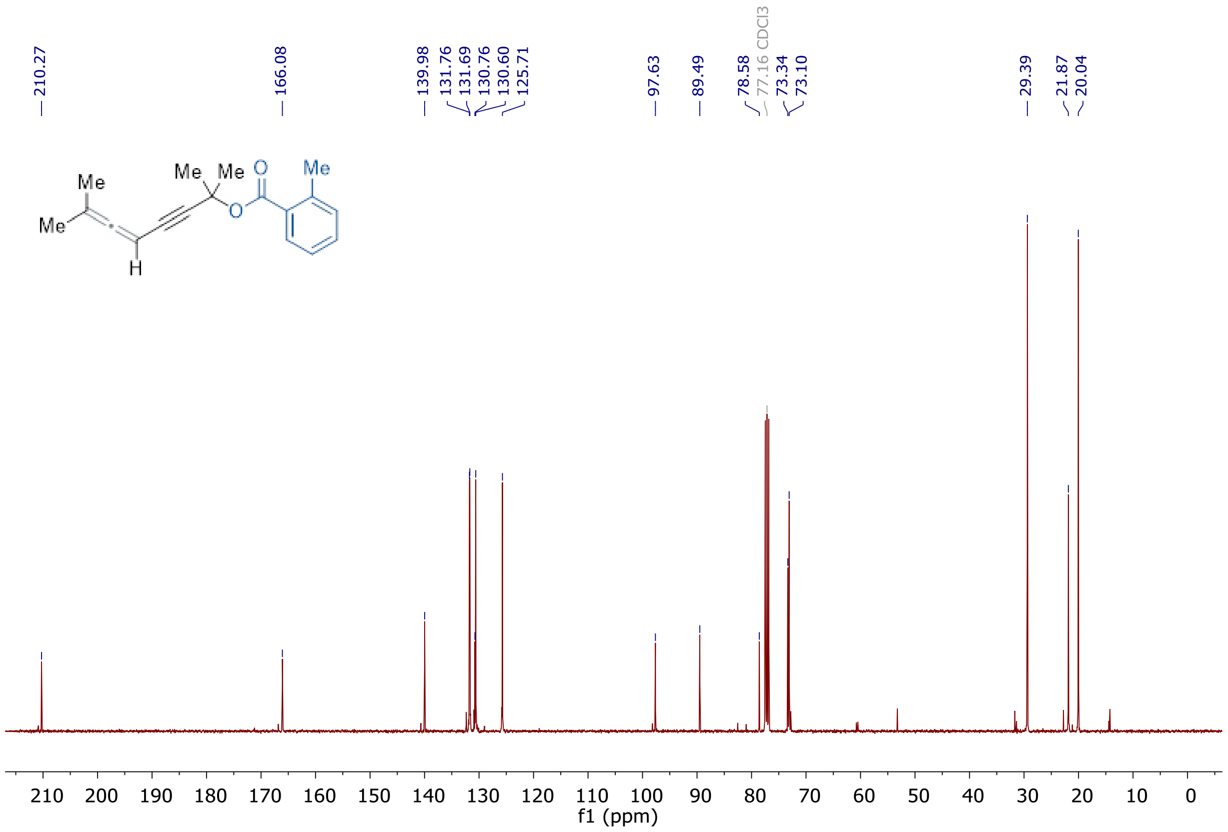

[13]**C NMR** of compound **2g** (101 MHz, $CDCl_3$)

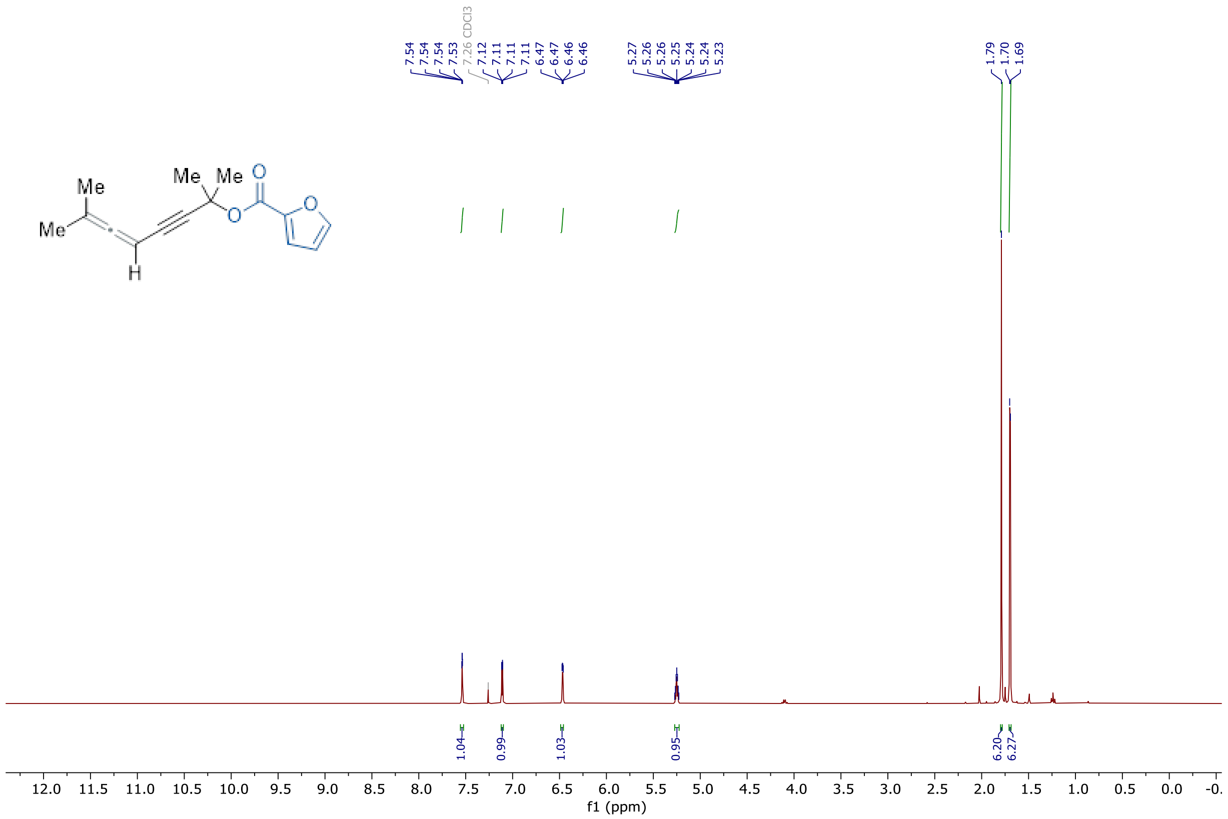

^{1}H NMR of compound **2h** (400 MHz, $CDCl_3$)

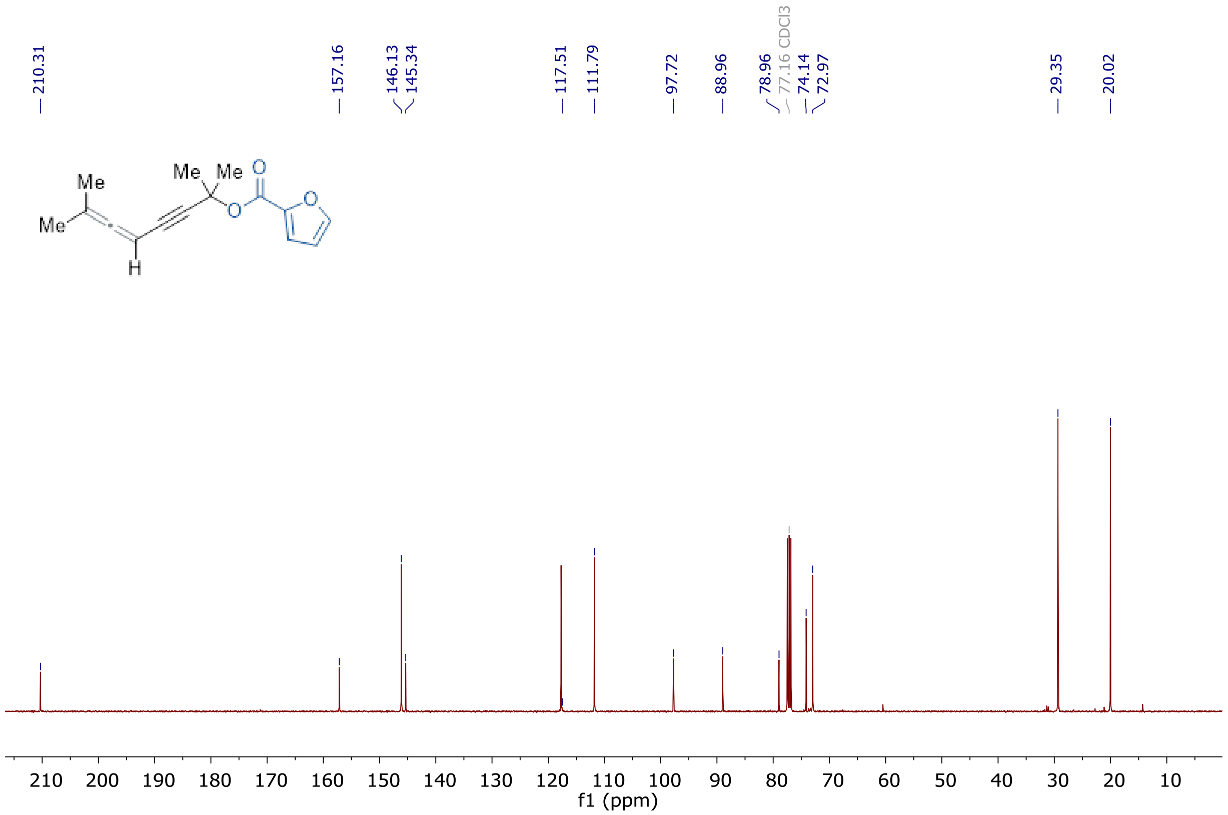

^{13}C NMR of compound **2h** (101 MHz, $CDCl_3$)

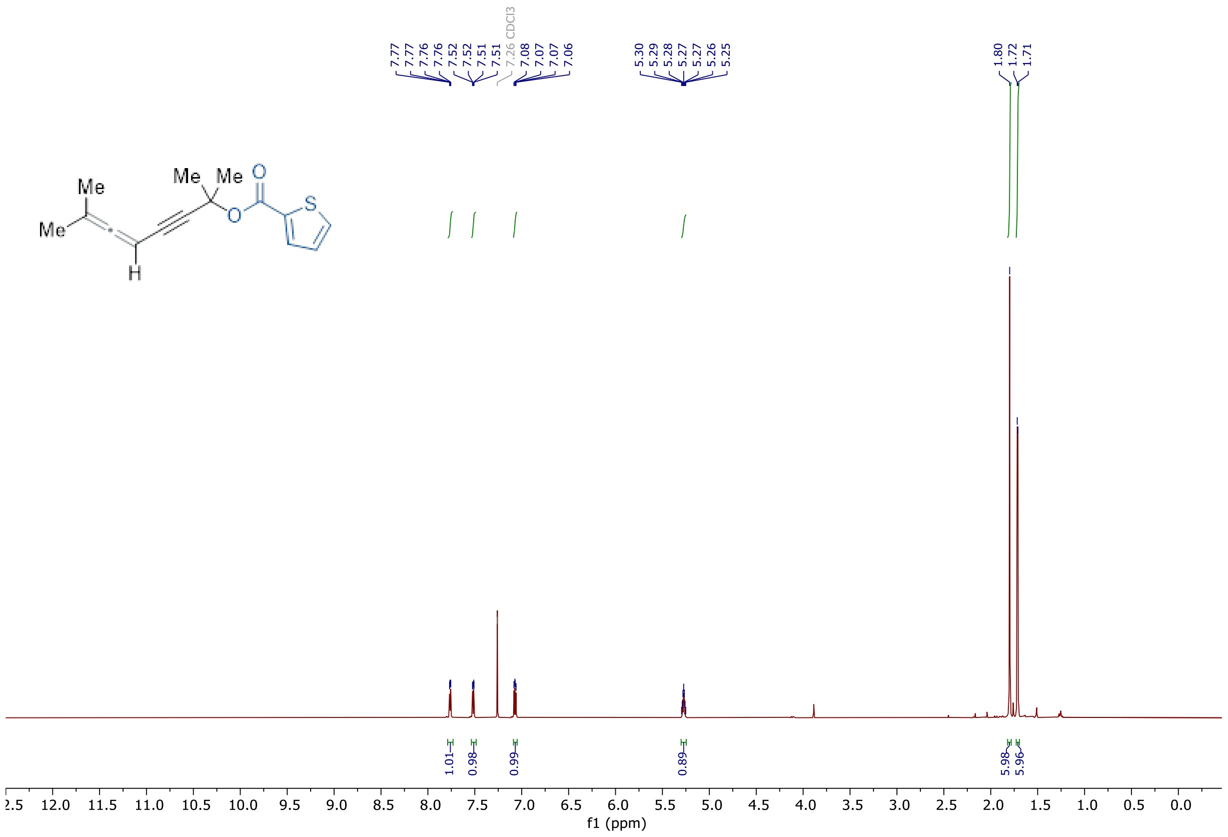

^{1}**H NMR** of compound **2i** (400 MHz, CDCl$_3$)

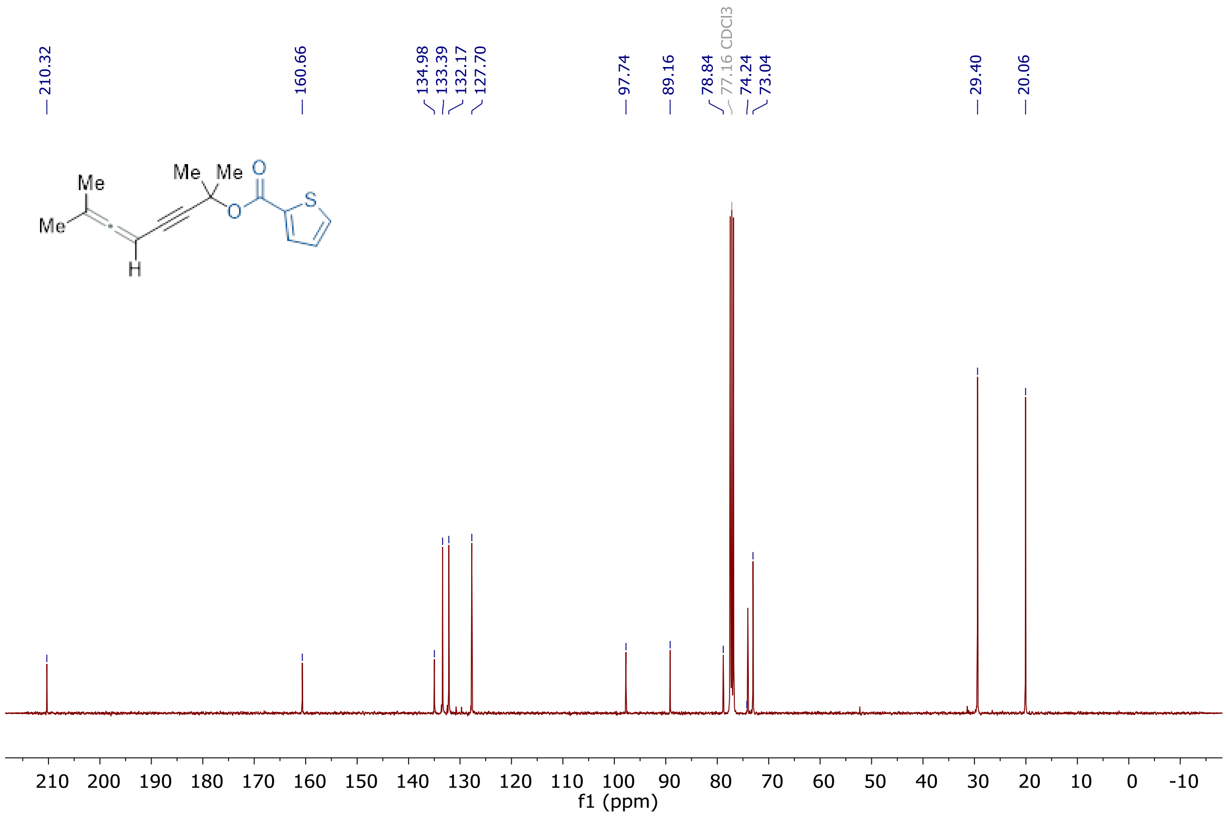

^{13}**C NMR** of compound **2i** (101 MHz, CDCl$_3$)

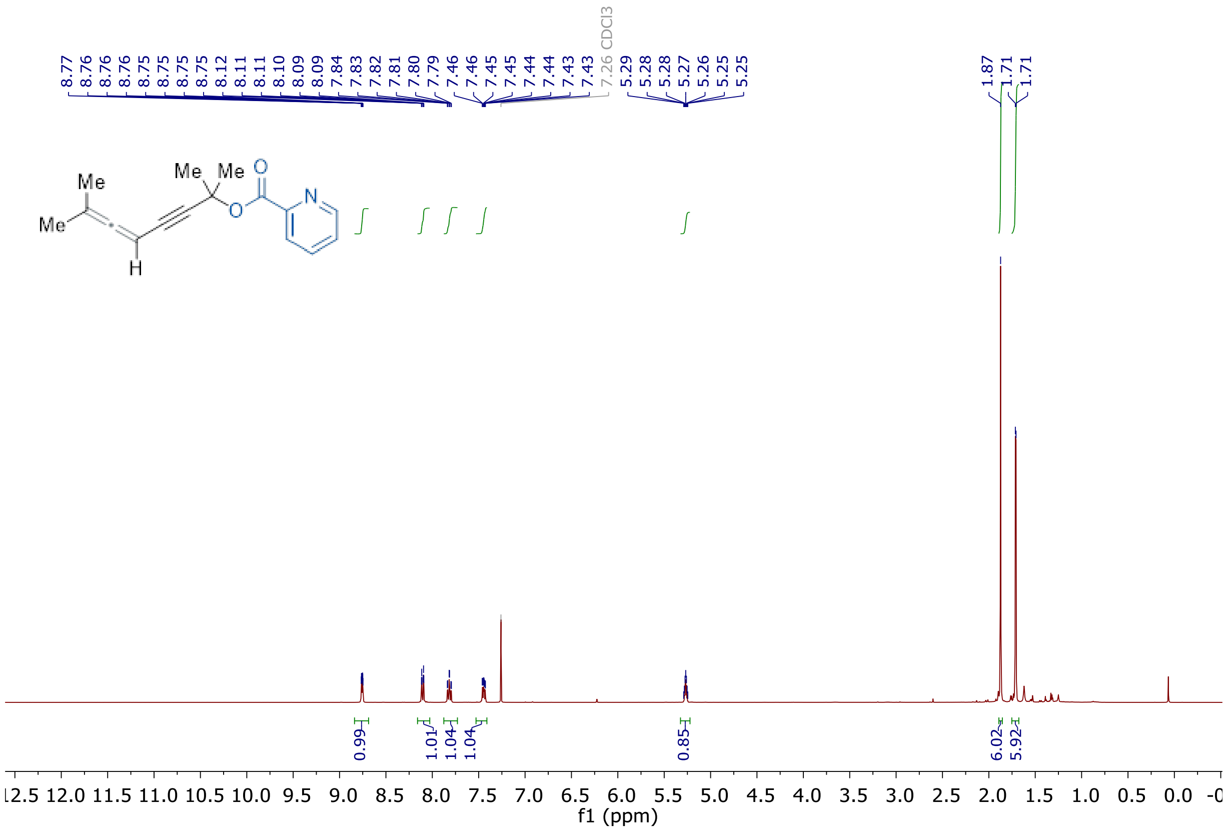

^{1}H NMR of compound 2j (400 MHz, $CDCl_3$)

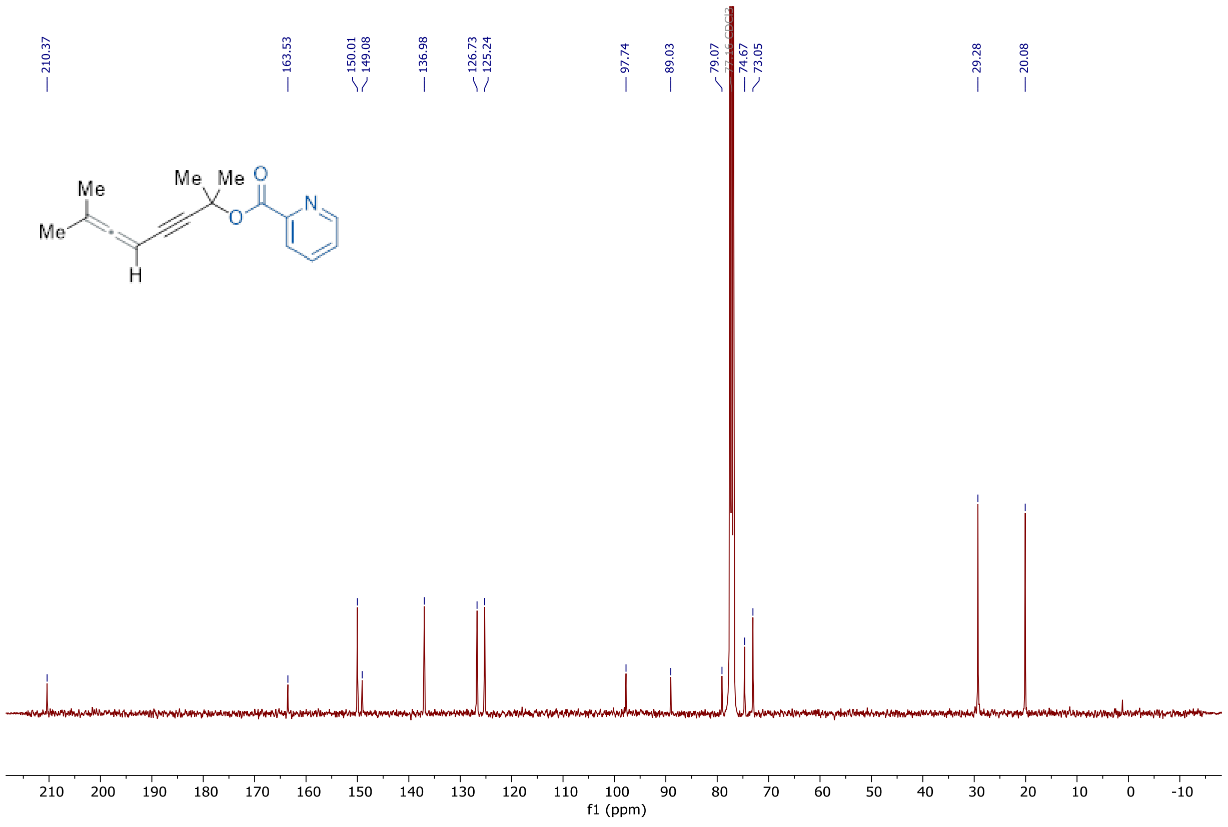

^{13}C NMR of compound 2j (101 MHz, $CDCl_3$)

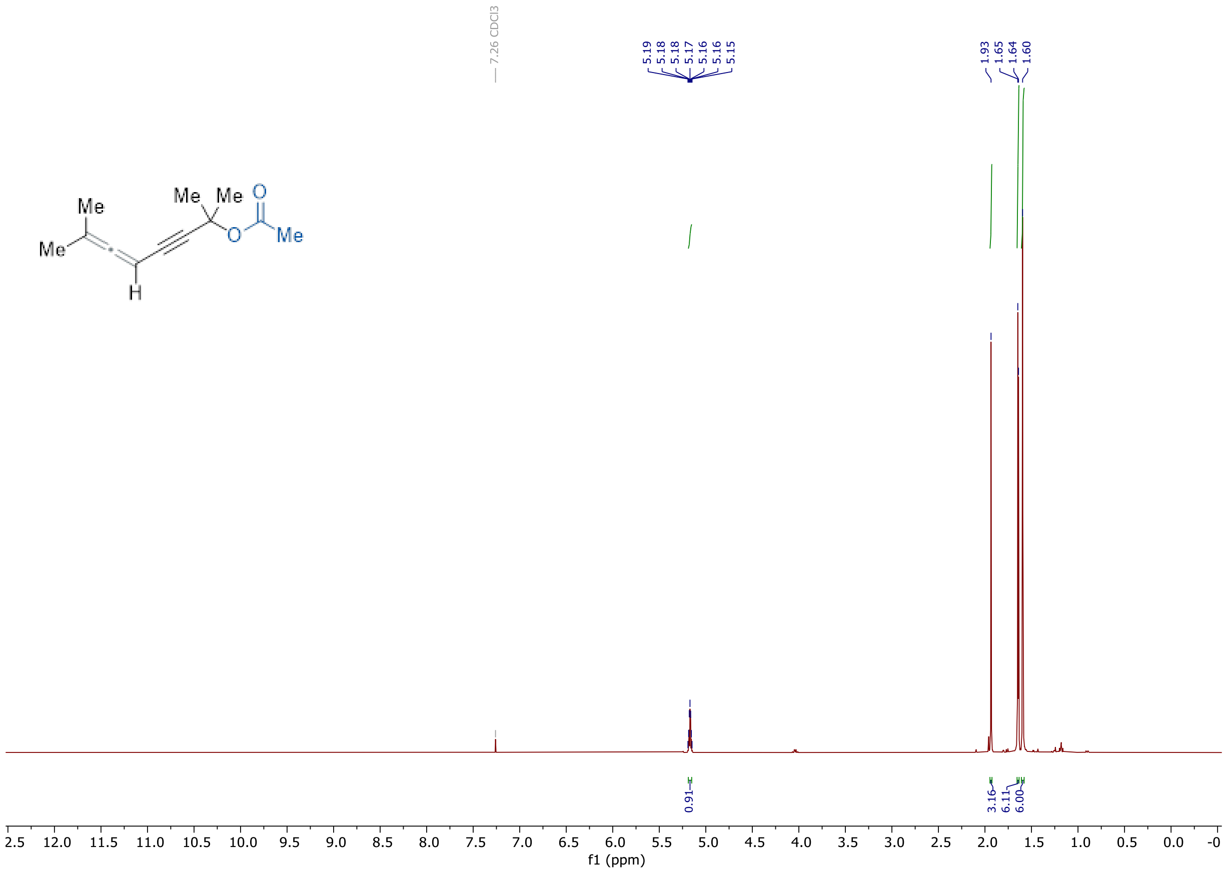

^{1}**H NMR** of compound **2k** (400 MHz, $CDCl_3$)

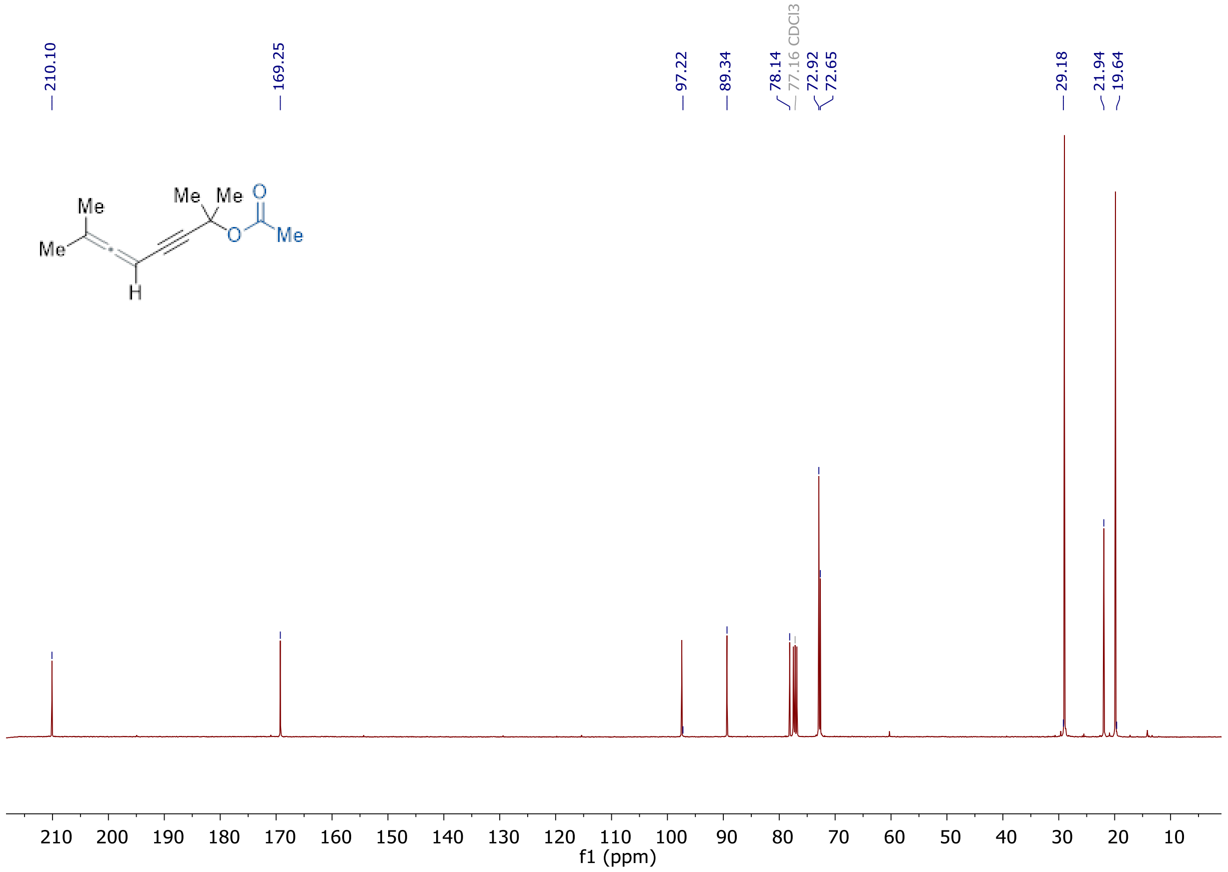

^{13}**C NMR** of compound **2k** (101 MHz, $CDCl_3$)

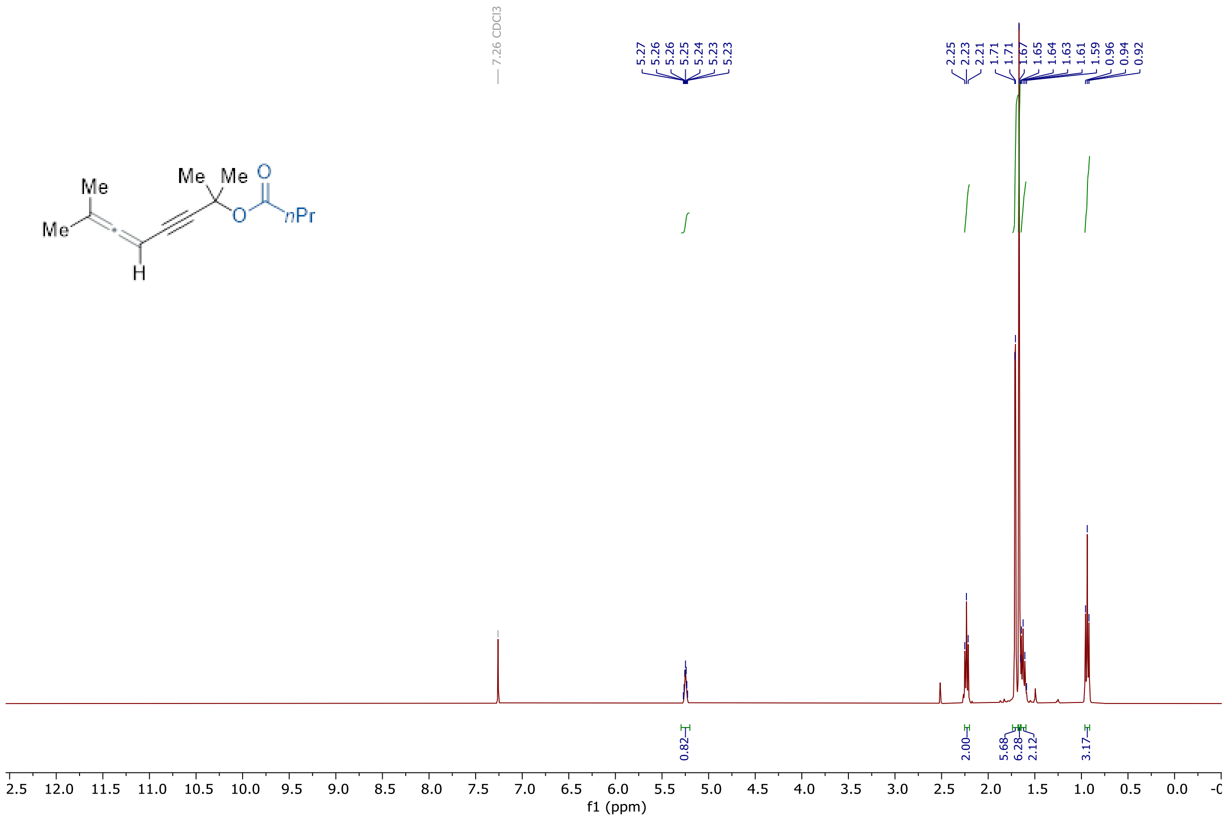

^{1}**H NMR** of compound **2l** (400 MHz, $CDCl_3$)

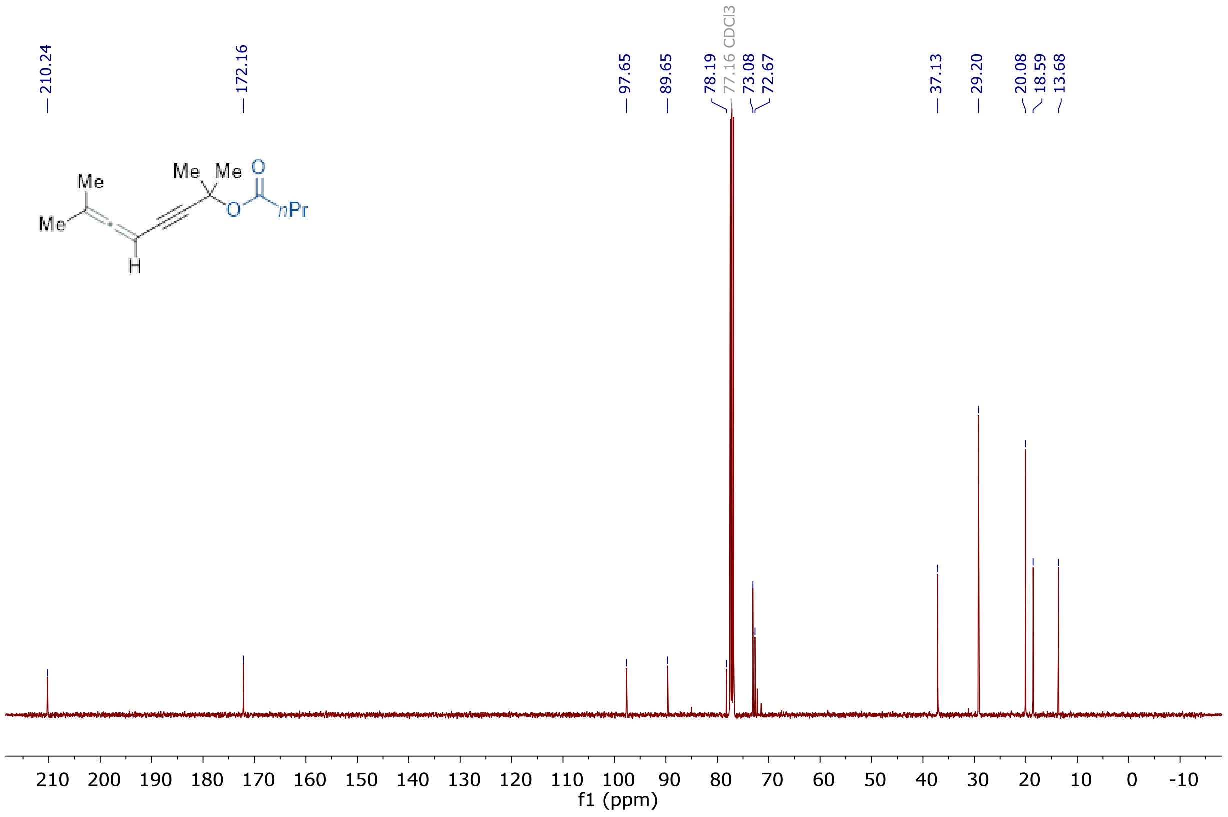

^{13}**C NMR** of compound **2l** (101 MHz, $CDCl_3$)

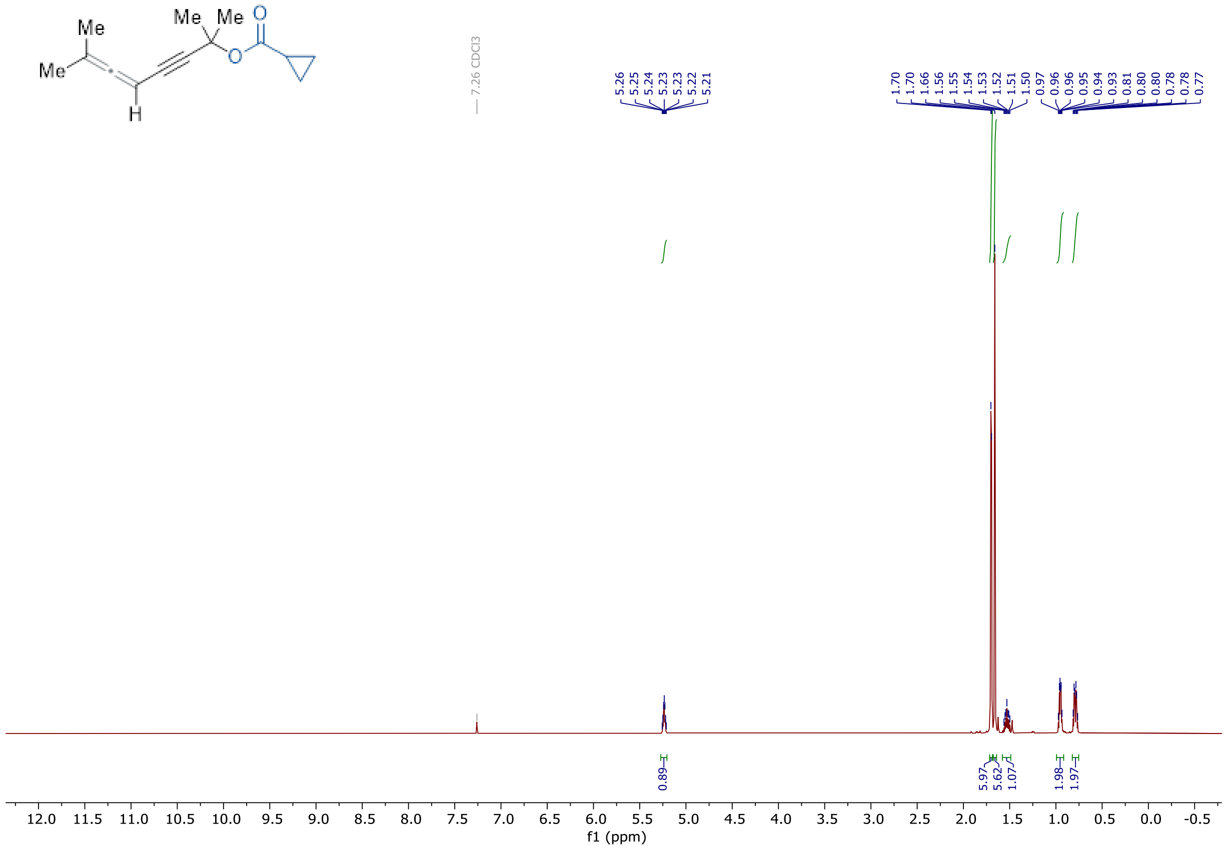

^{1}H NMR of compound **2m** (400 MHz, $CDCl_3$)

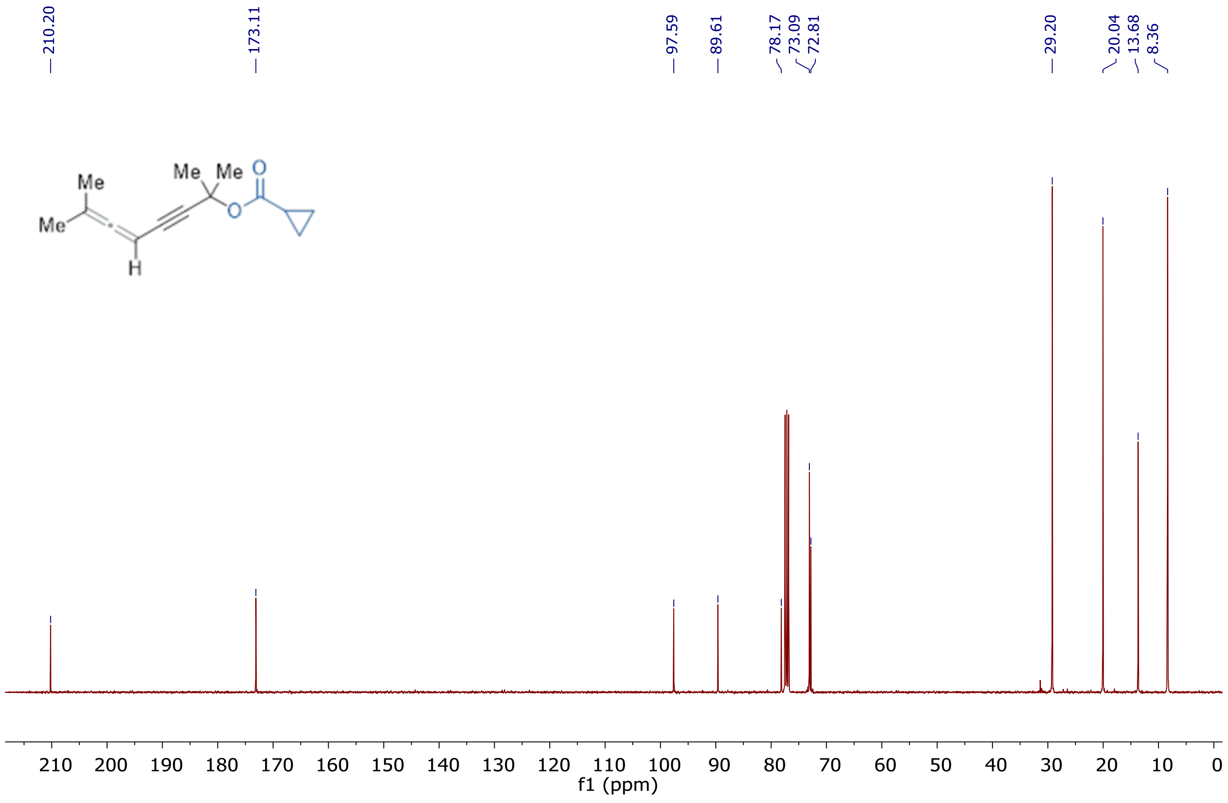

^{13}C NMR of compound **2m** (101 MHz, $CDCl_3$)

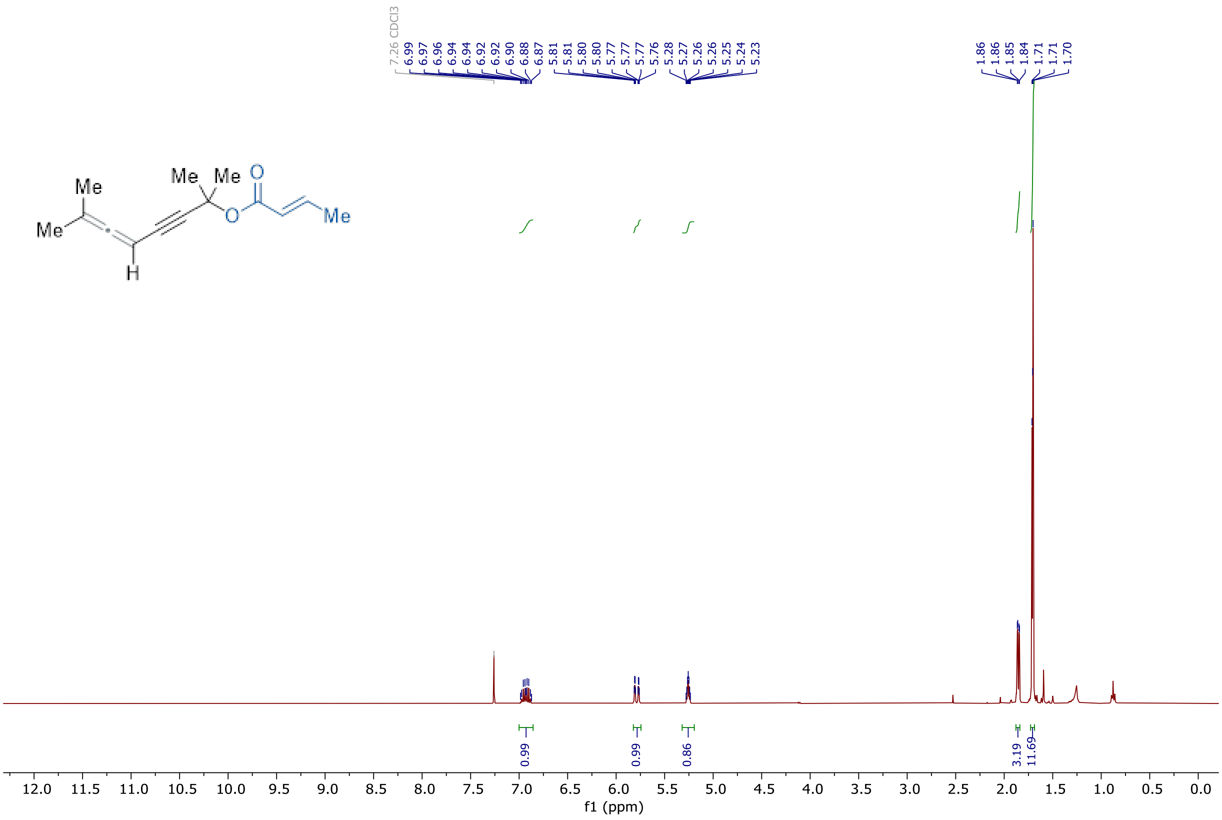

^{1}H NMR of compound **2n** (400 MHz, $CDCl_3$)

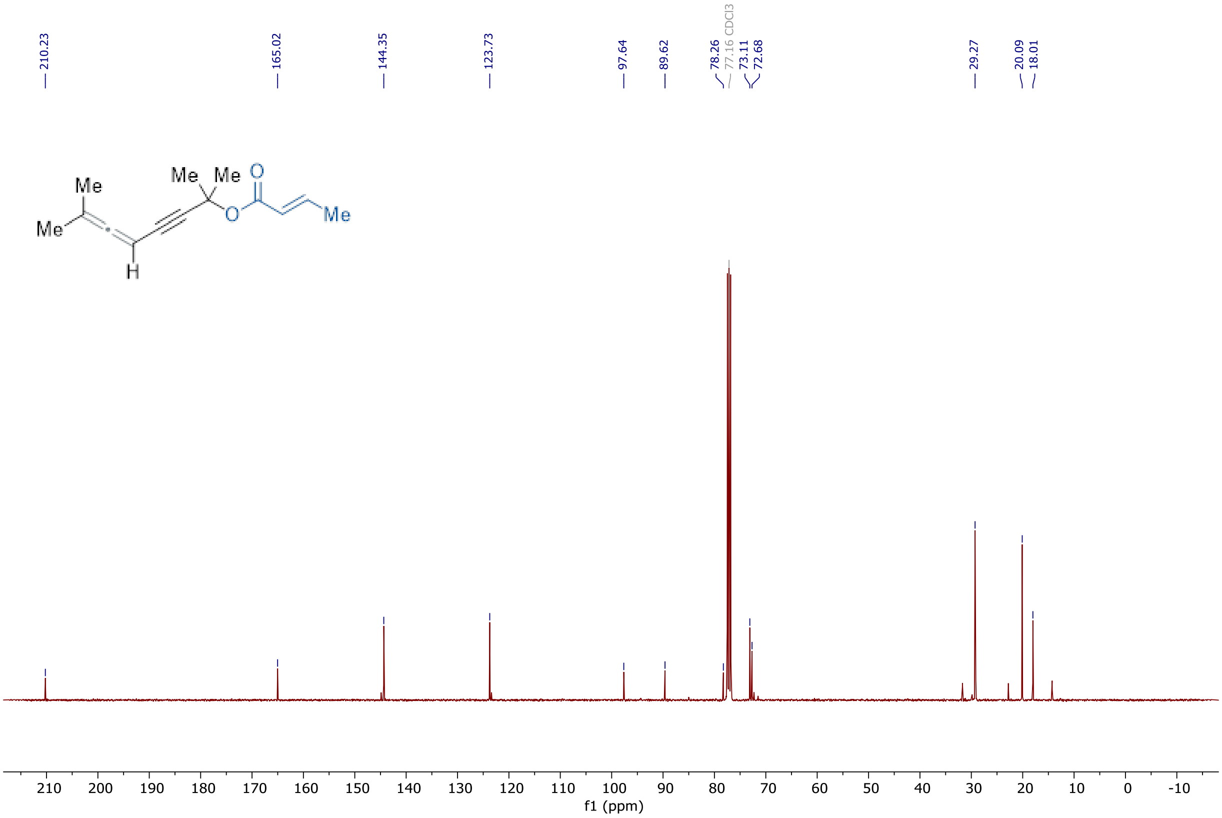

^{13}C NMR of compound **2n** (101 MHz, $CDCl_3$)

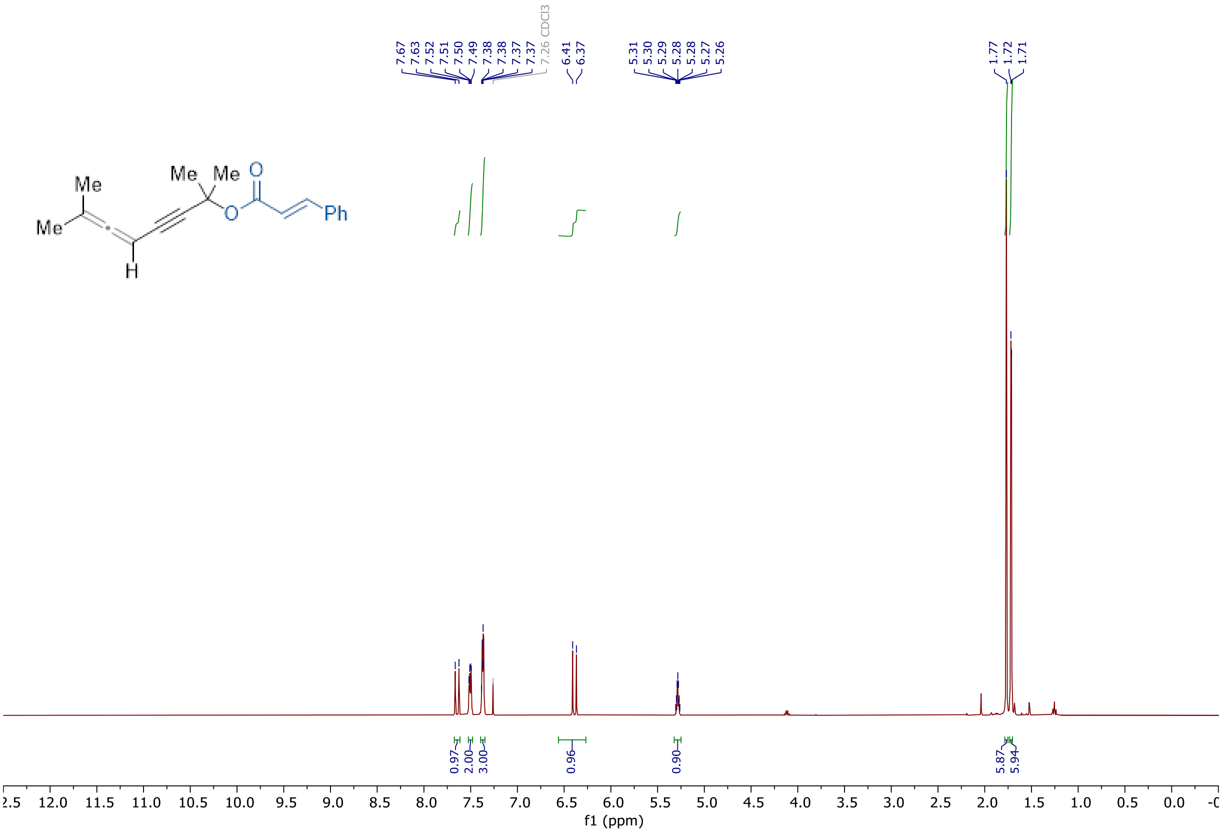

^{1}H NMR of compound **2o** (400 MHz, $CDCl_3$)

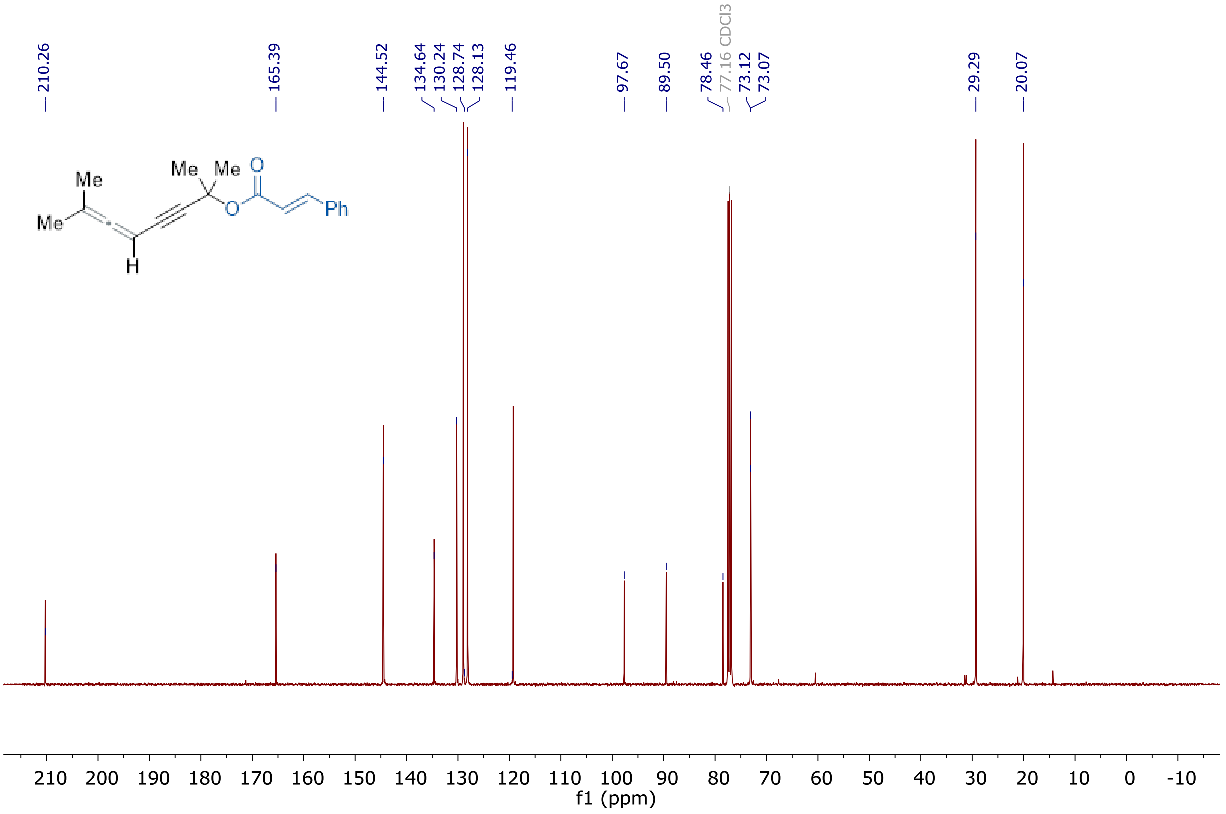

^{13}C NMR of compound **2o** (101 MHz, $CDCl_3$)

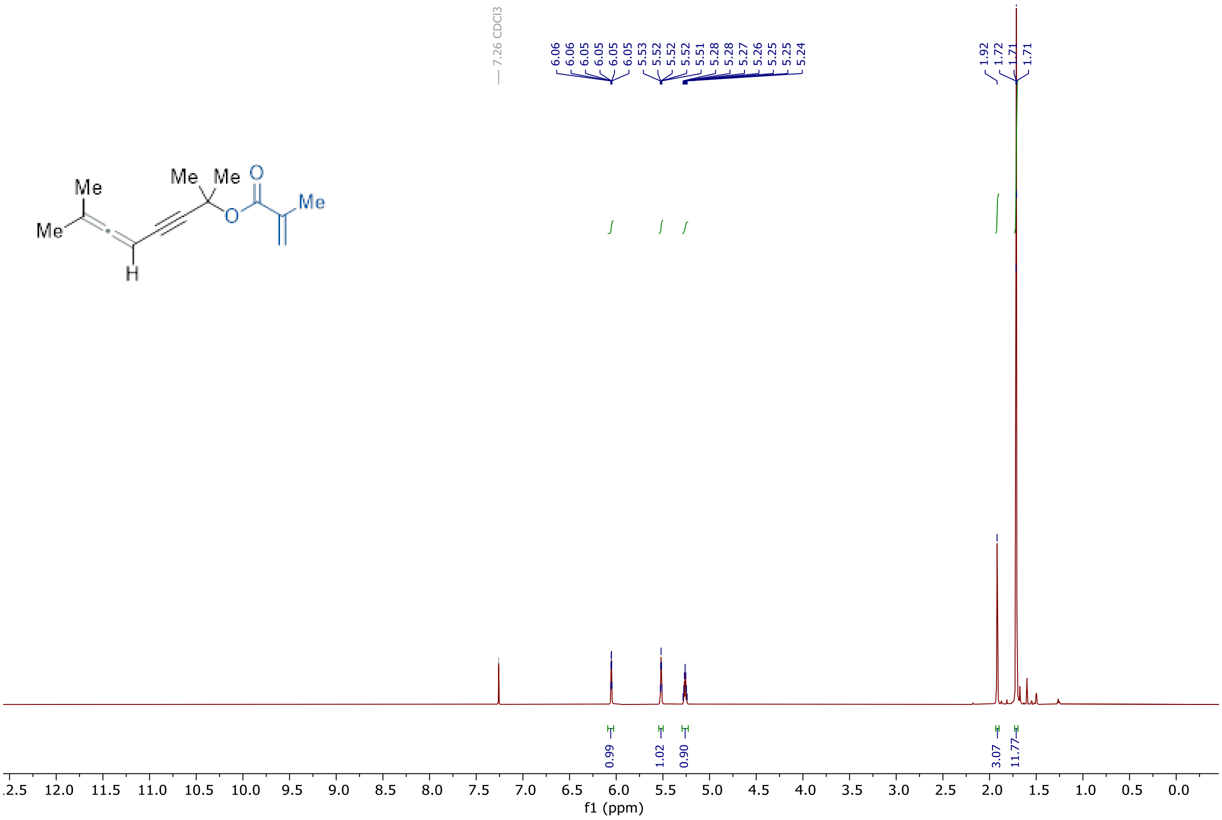

^{1}H NMR of compound **2p** (400 MHz, $CDCl_3$)

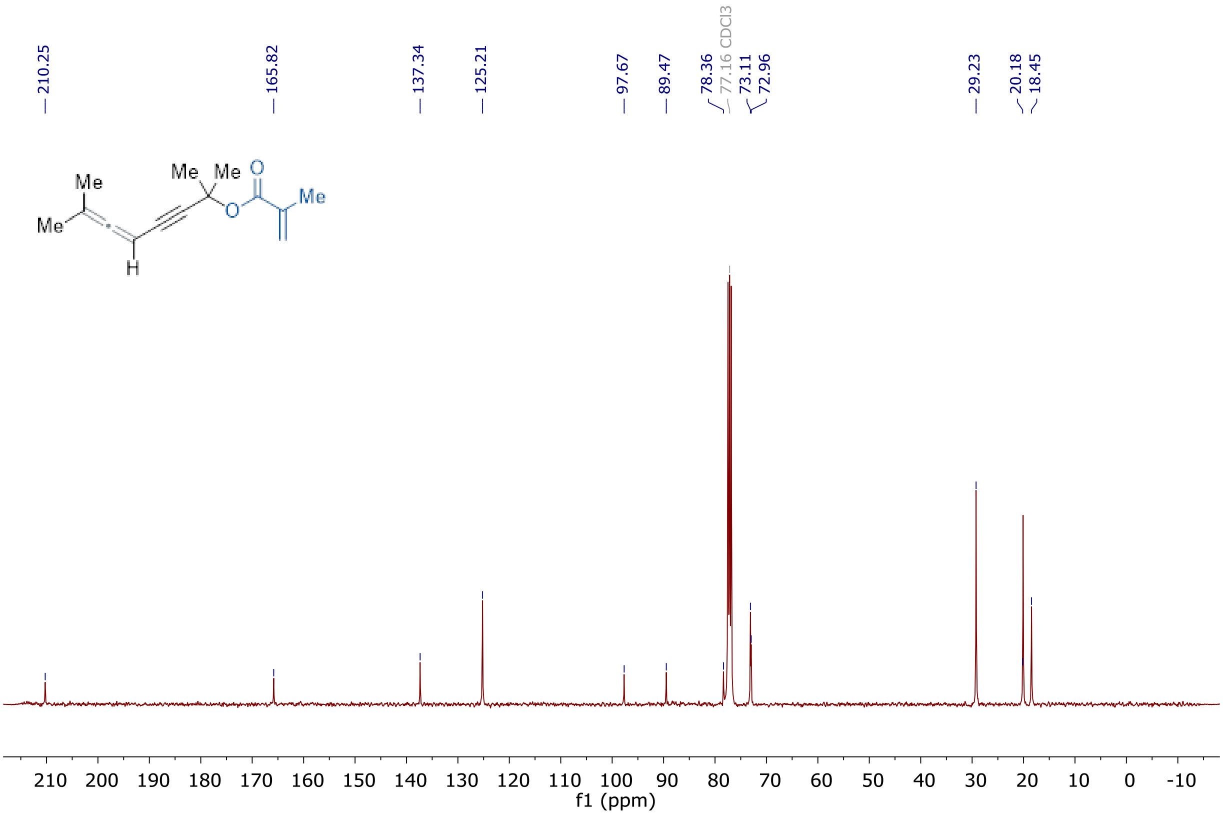

^{13}C NMR of compound **2p** (101 MHz, $CDCl_3$)

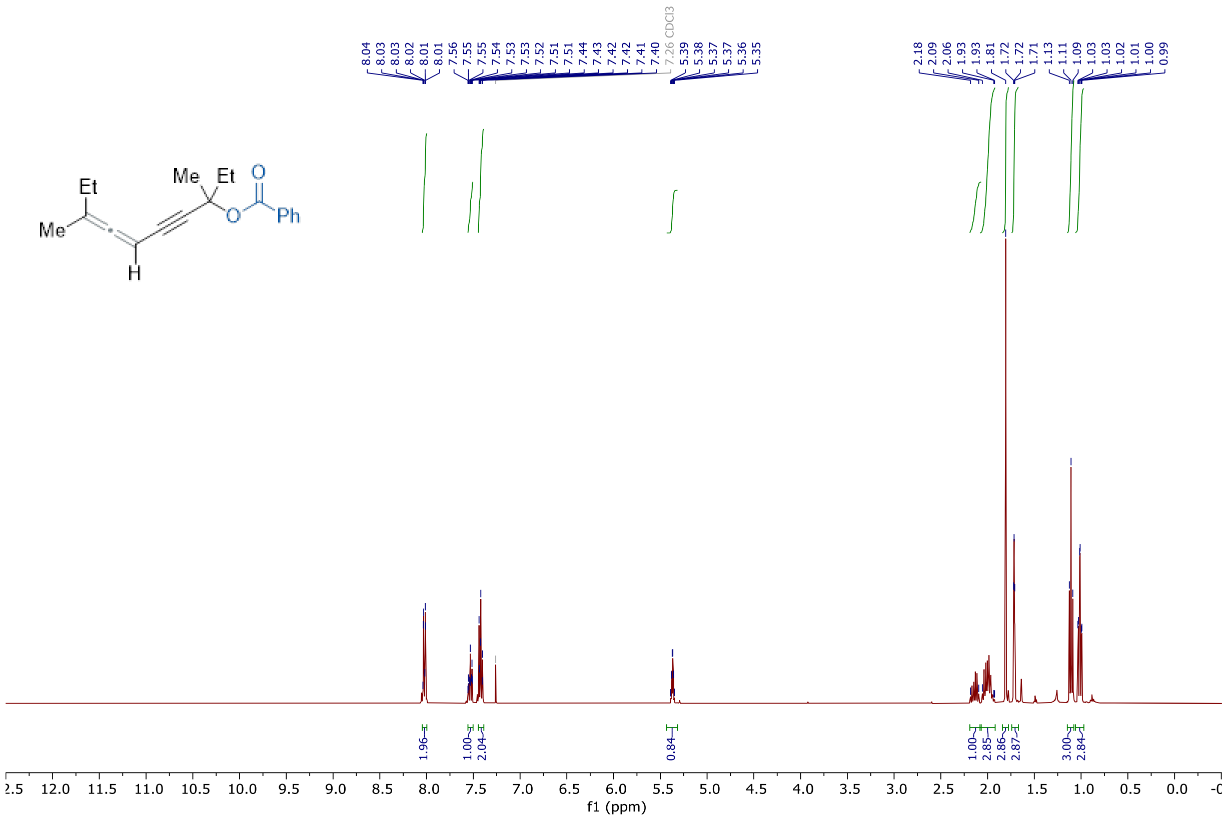

[1]**H NMR** of compound **2q** (400 MHz, $CDCl_3$)

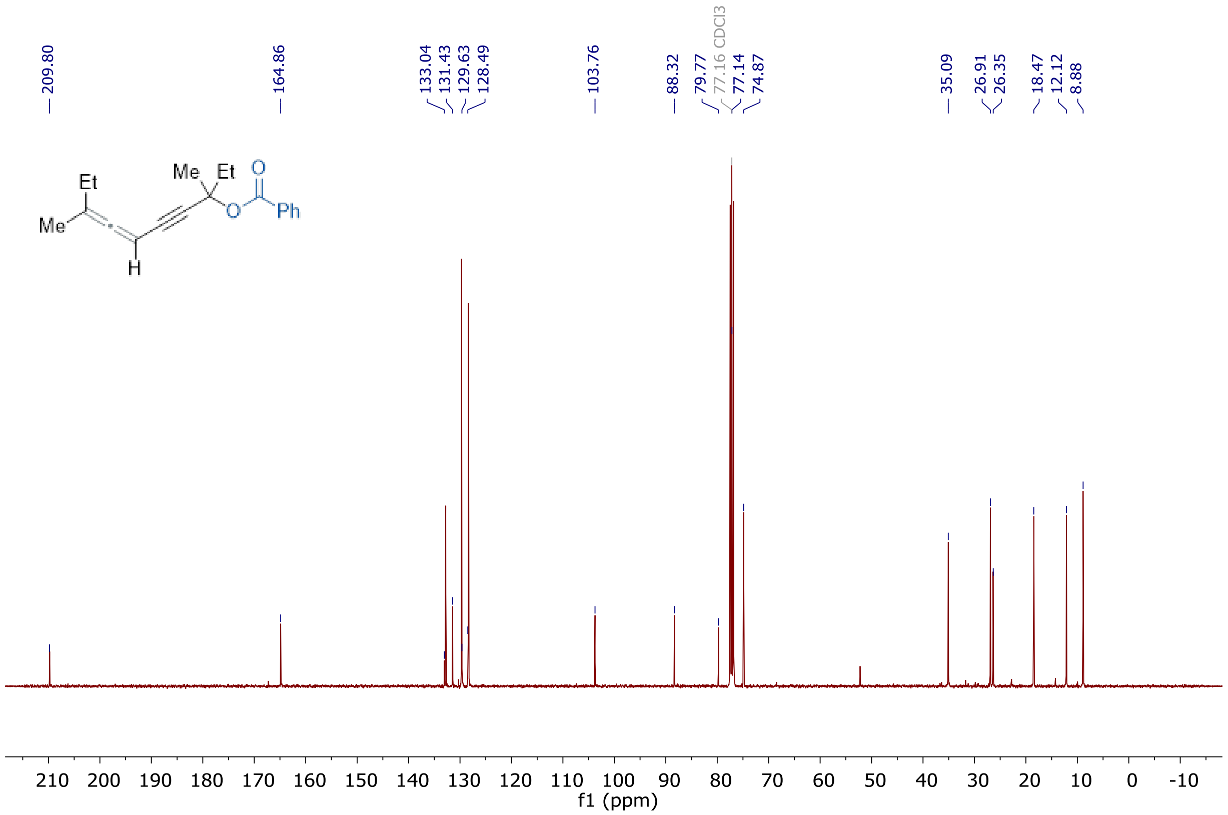

[13]**C NMR** of compound **2q** (101 MHz, $CDCl_3$)

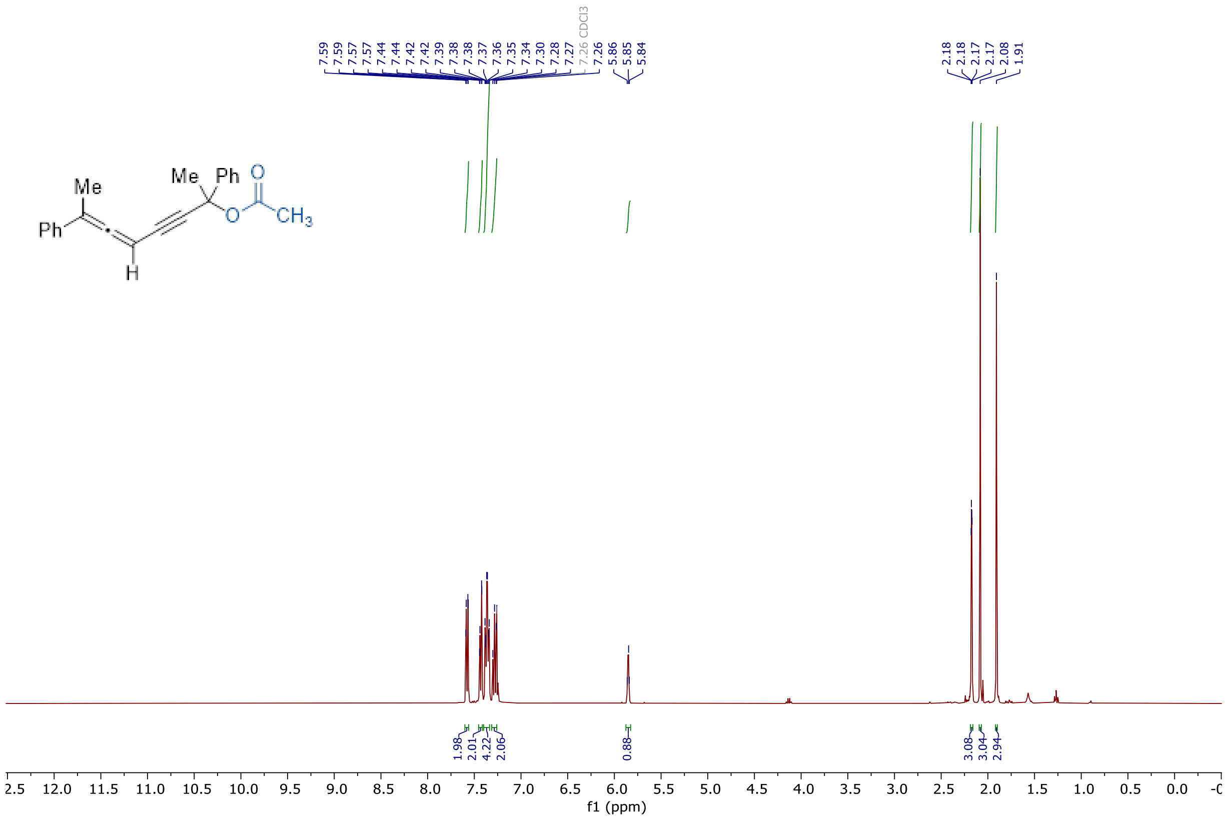

^{1}**H NMR** of compound **2r** (400 MHz, $CDCl_3$)

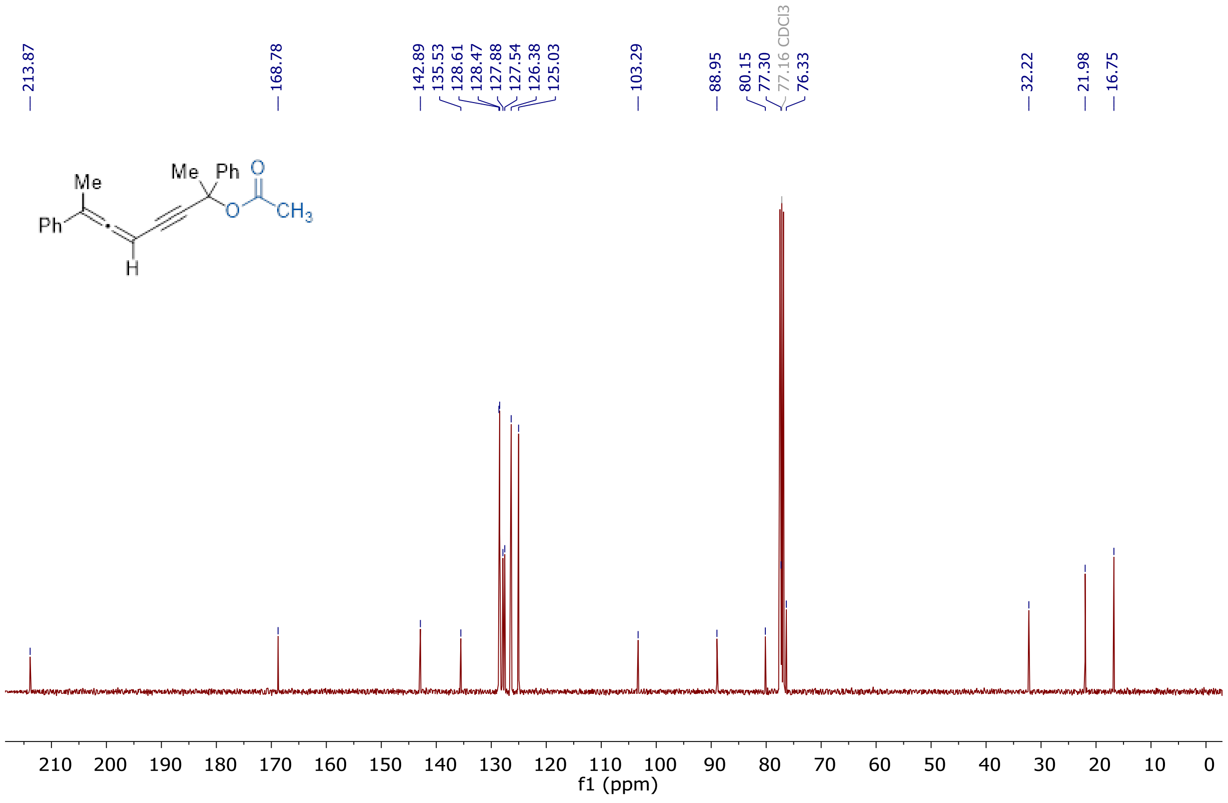

^{13}**C NMR** of compound **2r** (101 MHz, $CDCl_3$)

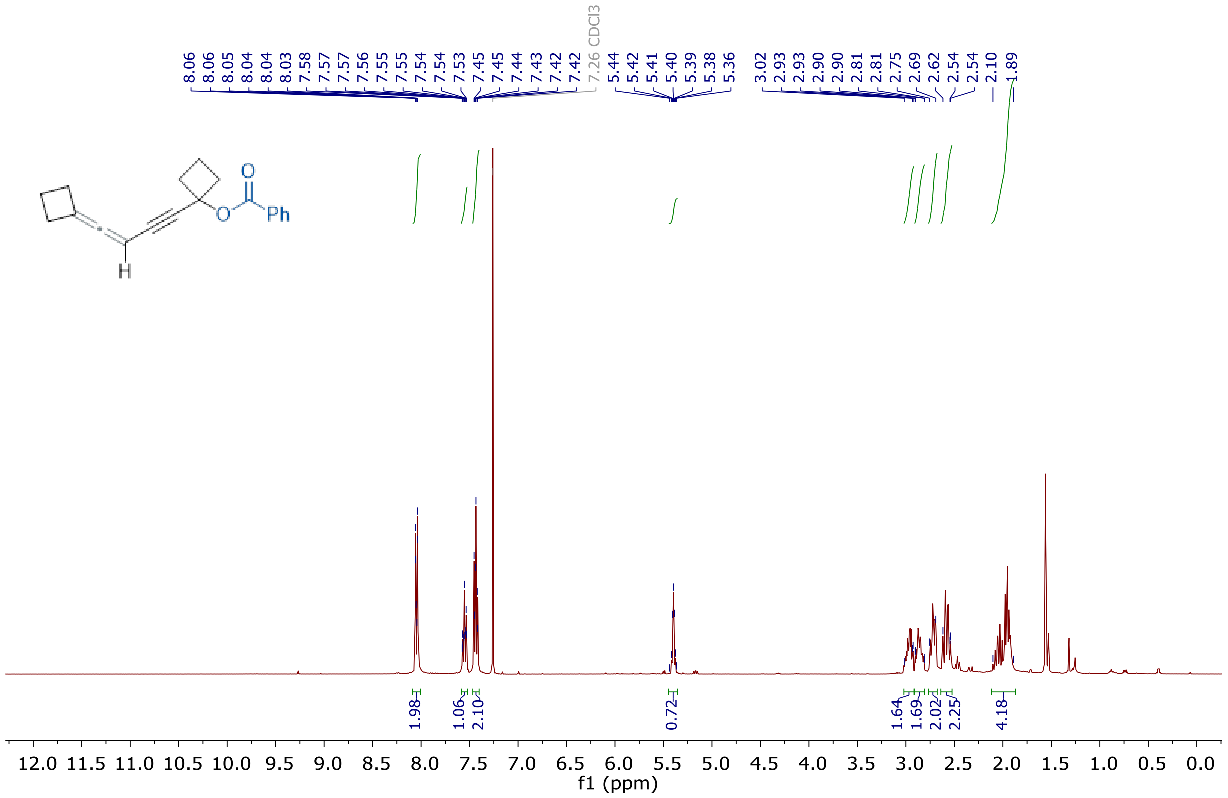

^{1}**H NMR** of compound **2s** (400 MHz, $CDCl_3$)

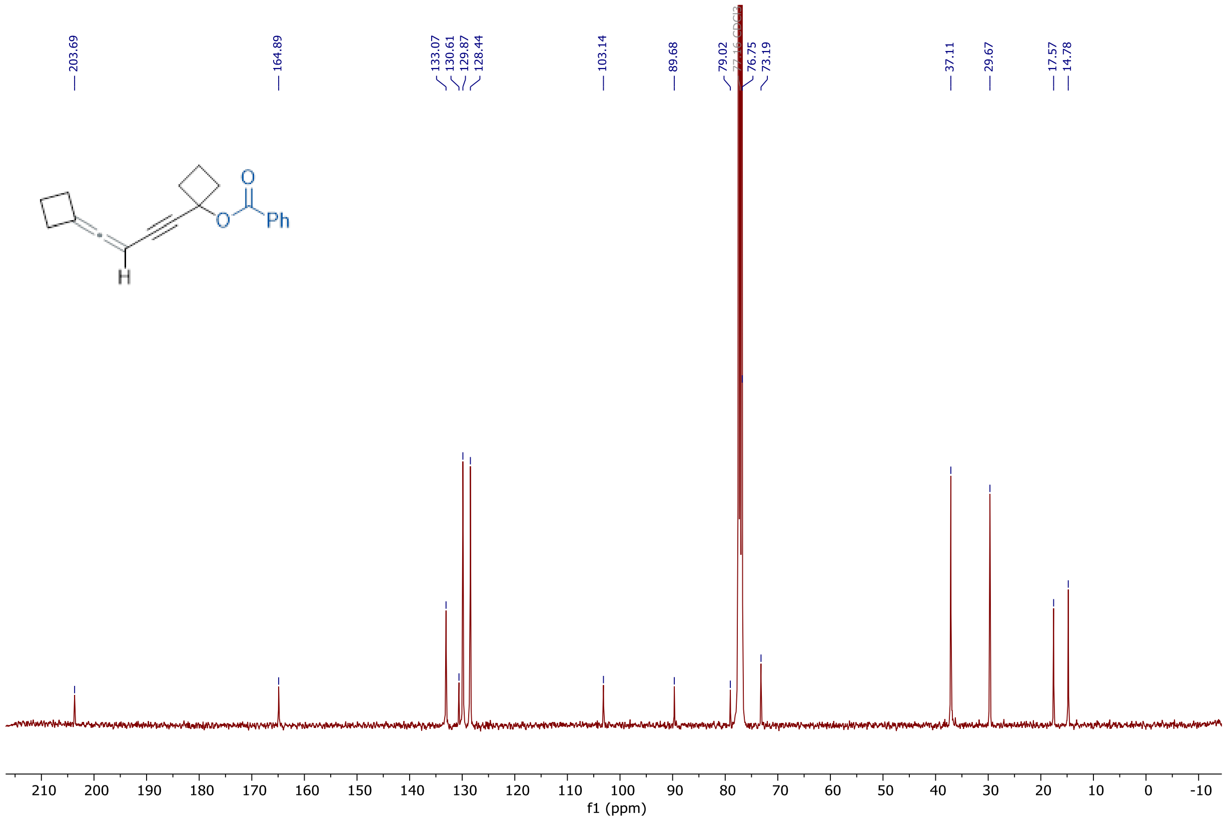

^{13}**C NMR** of compound **2s** (101 MHz, $CDCl_3$)

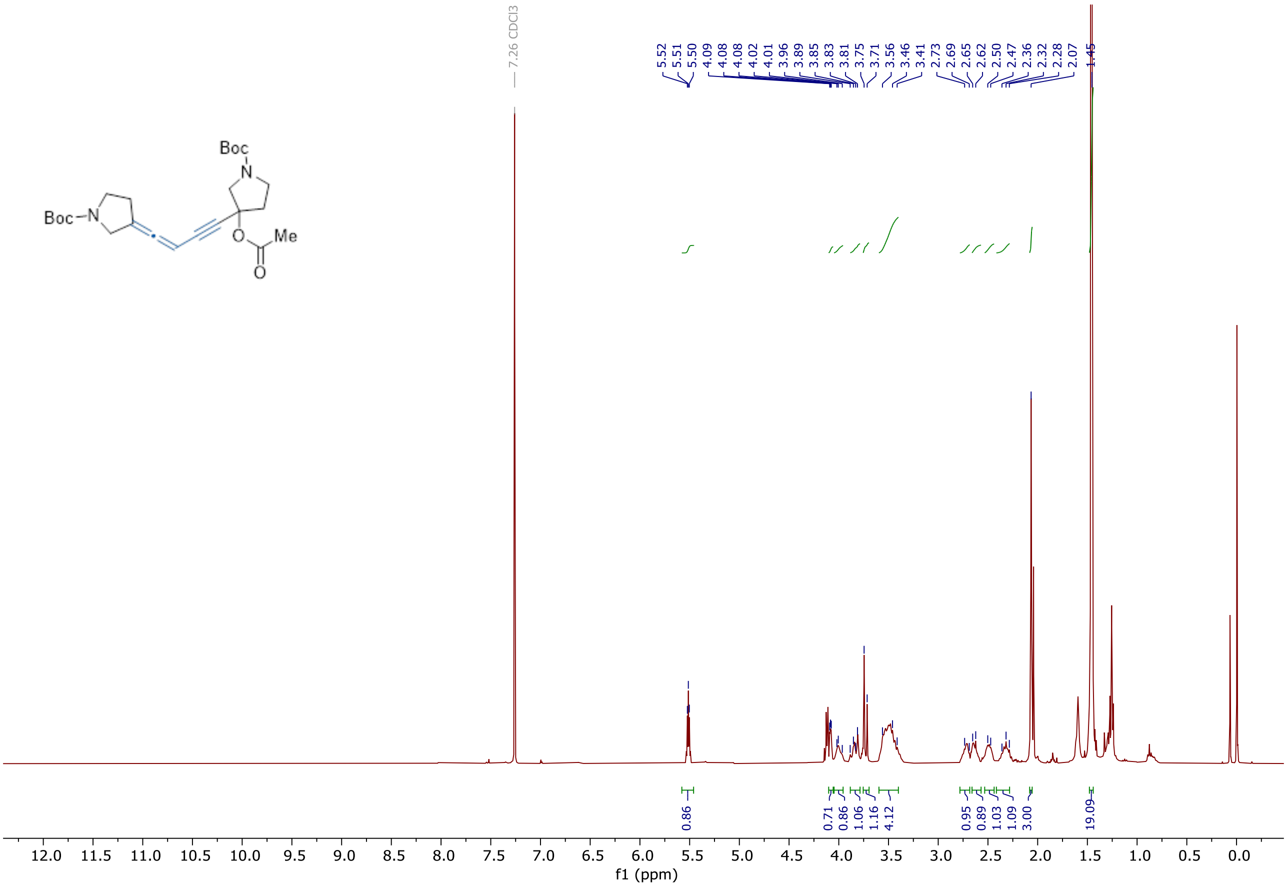

[1]**H NMR** of compound **2t** (400 MHz, $CDCl_3$)

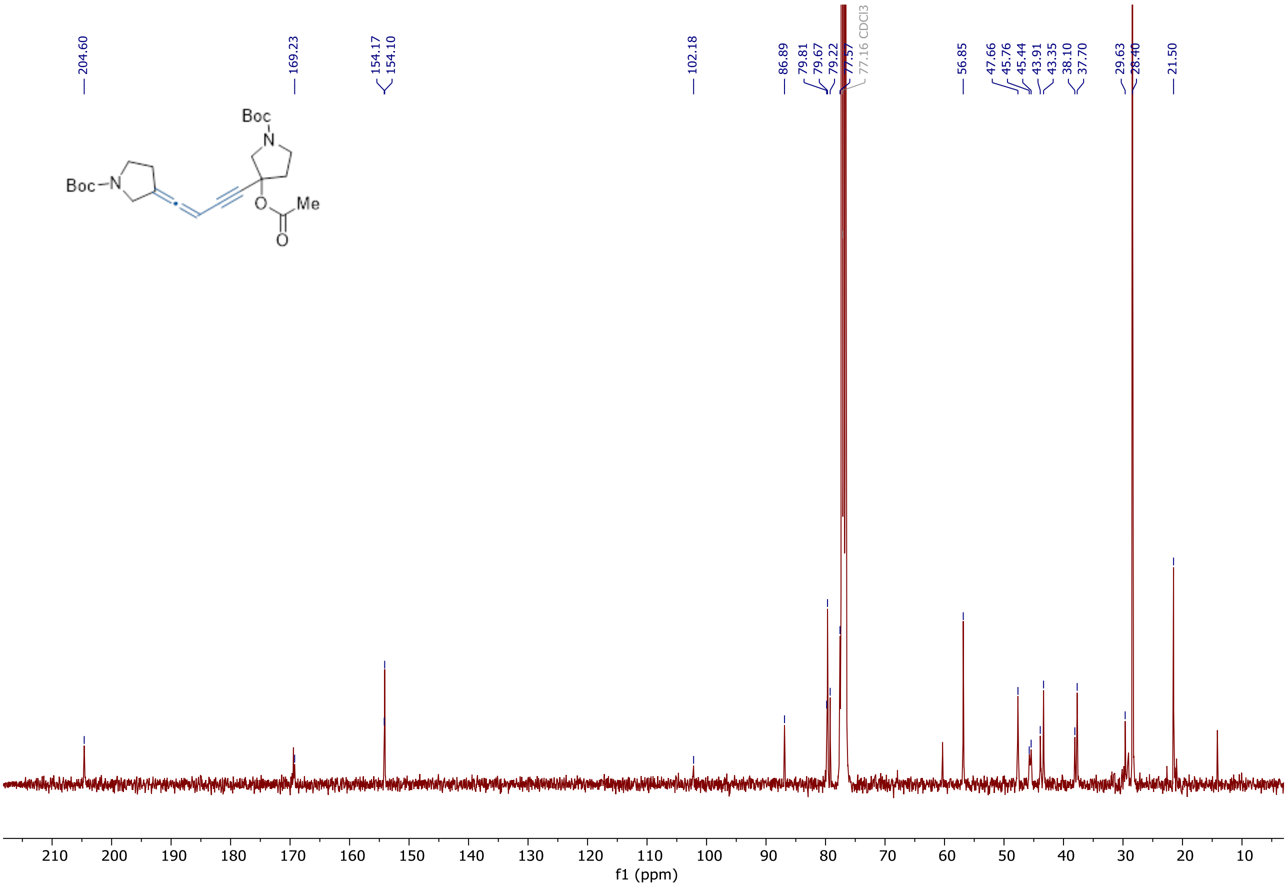

[13]**C NMR** of compound **2t** (101 MHz, $CDCl_3$)

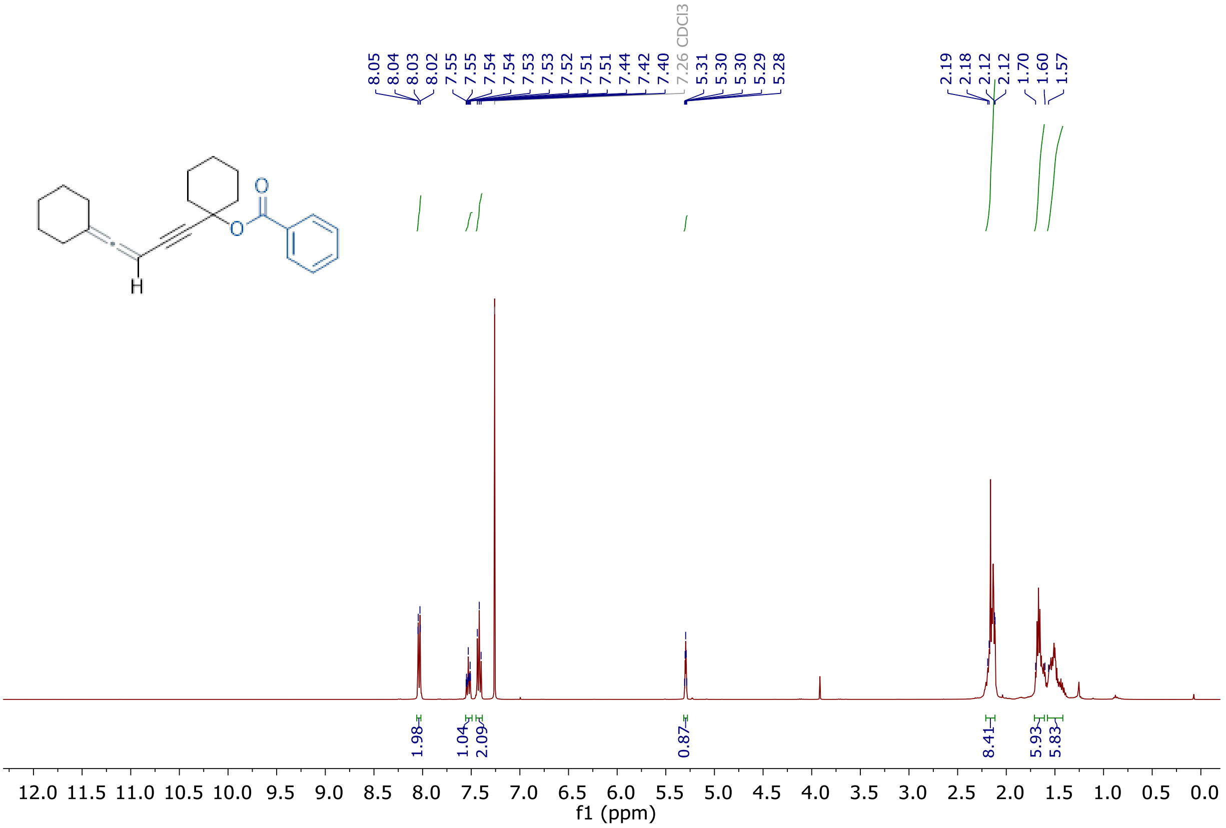

^{1}**H NMR** of compound **2u** (400 MHz, $CDCl_3$)

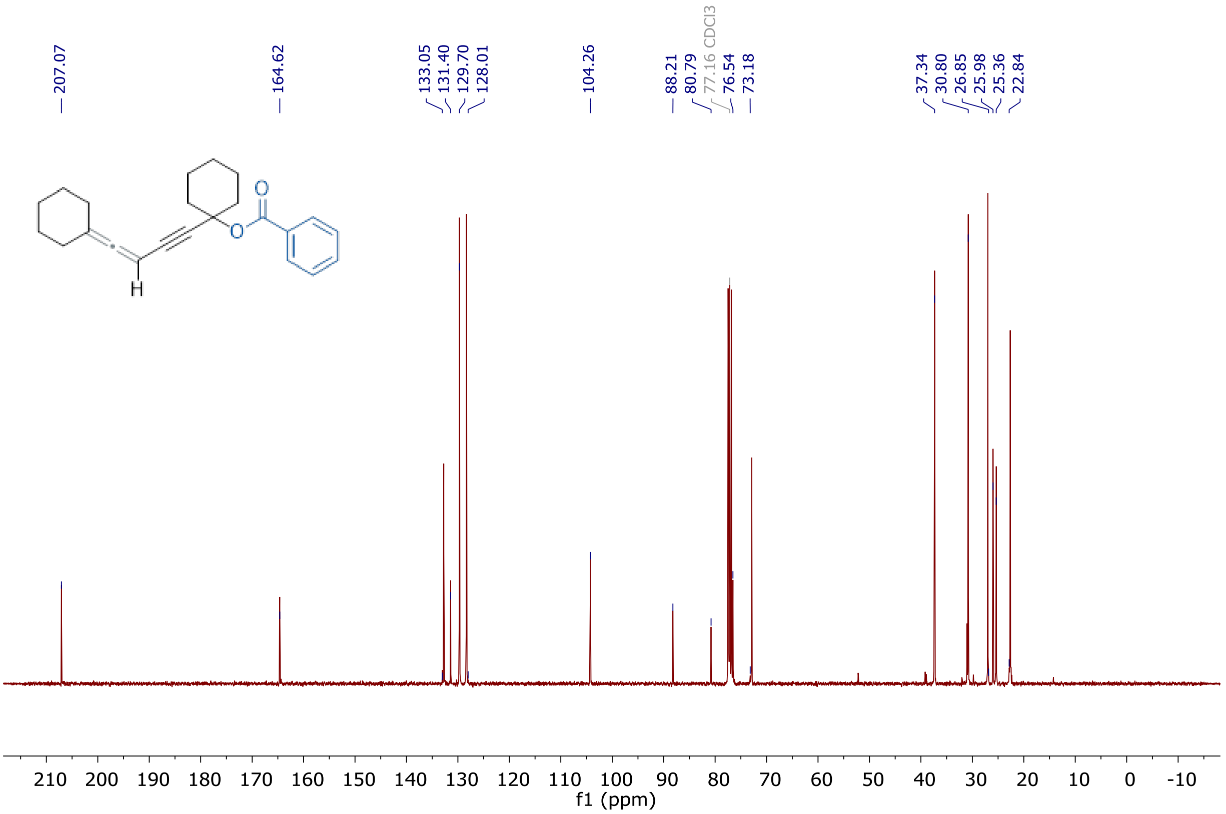

^{13}**C NMR** of compound **2u** (101 MHz, $CDCl_3$)

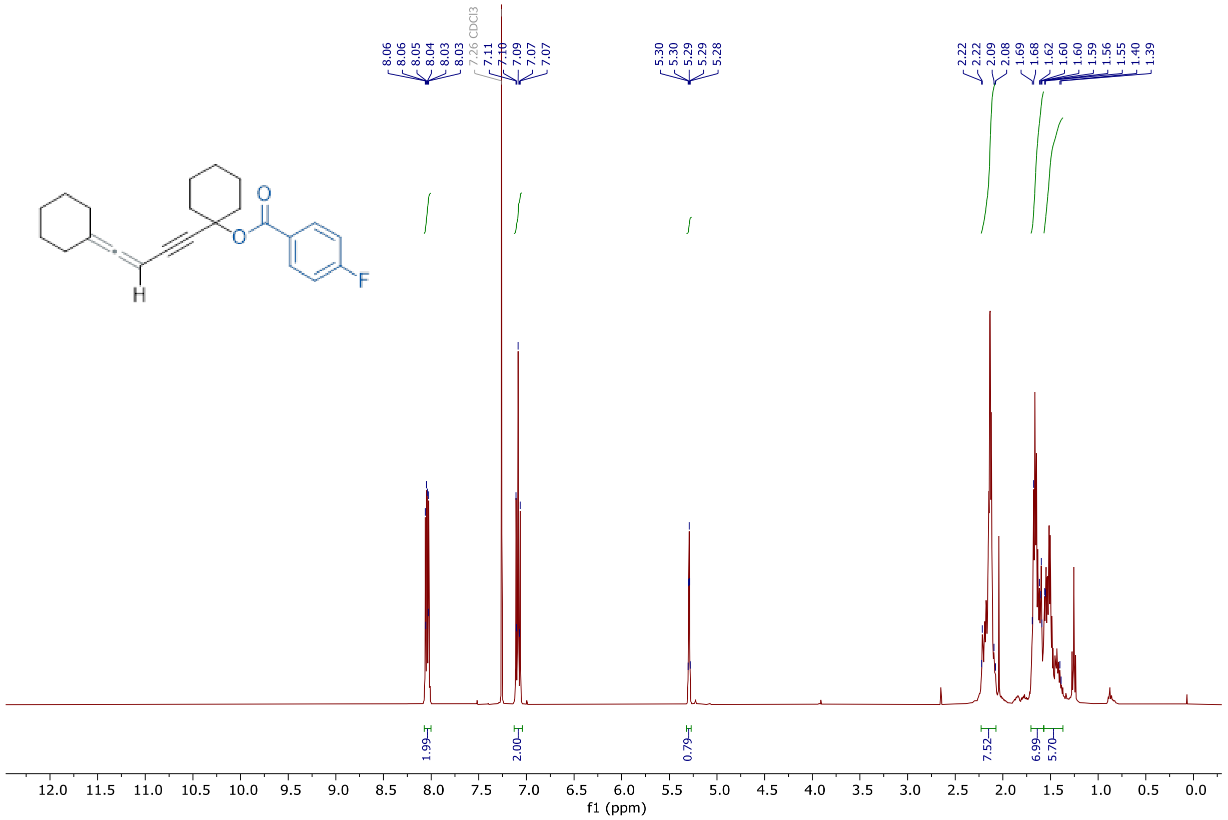

^{1}H NMR of compound **2v** (400 MHz, $CDCl_3$)

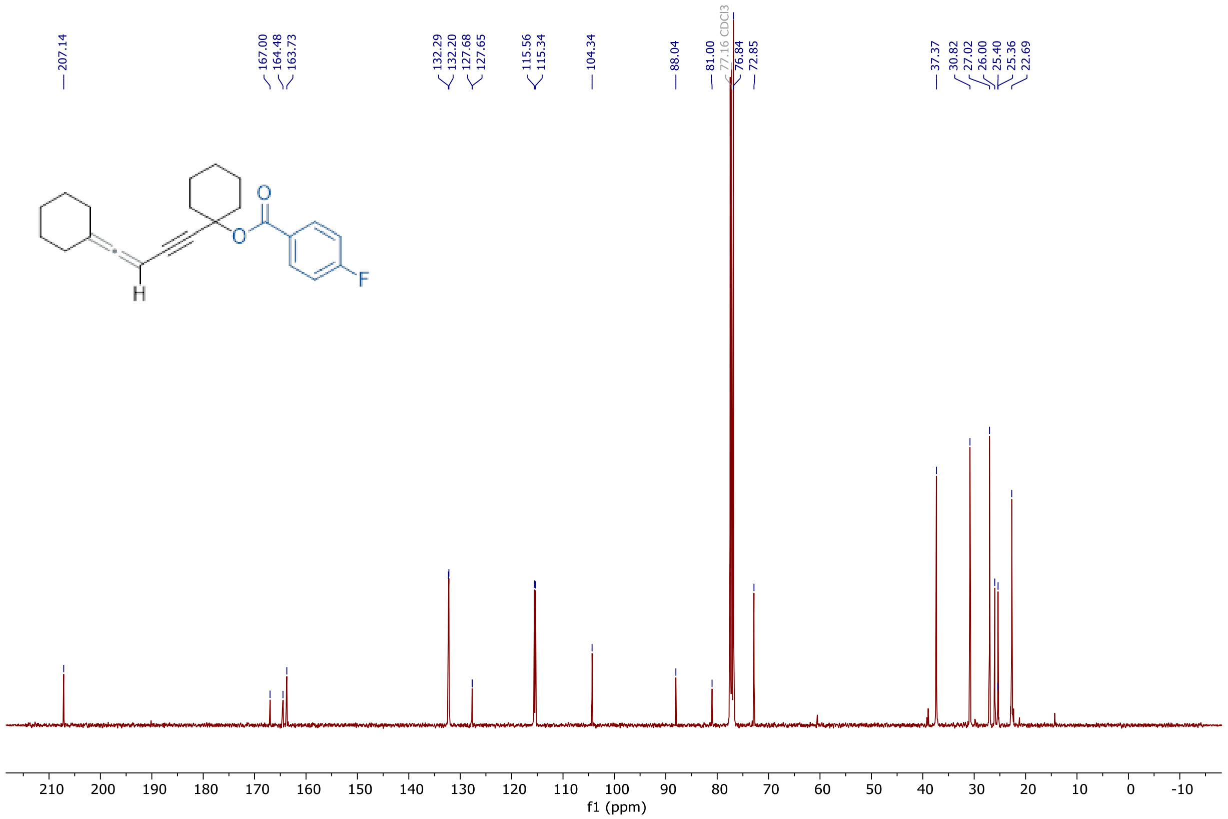

^{13}C NMR of compound **2v** (101 MHz, $CDCl_3$)

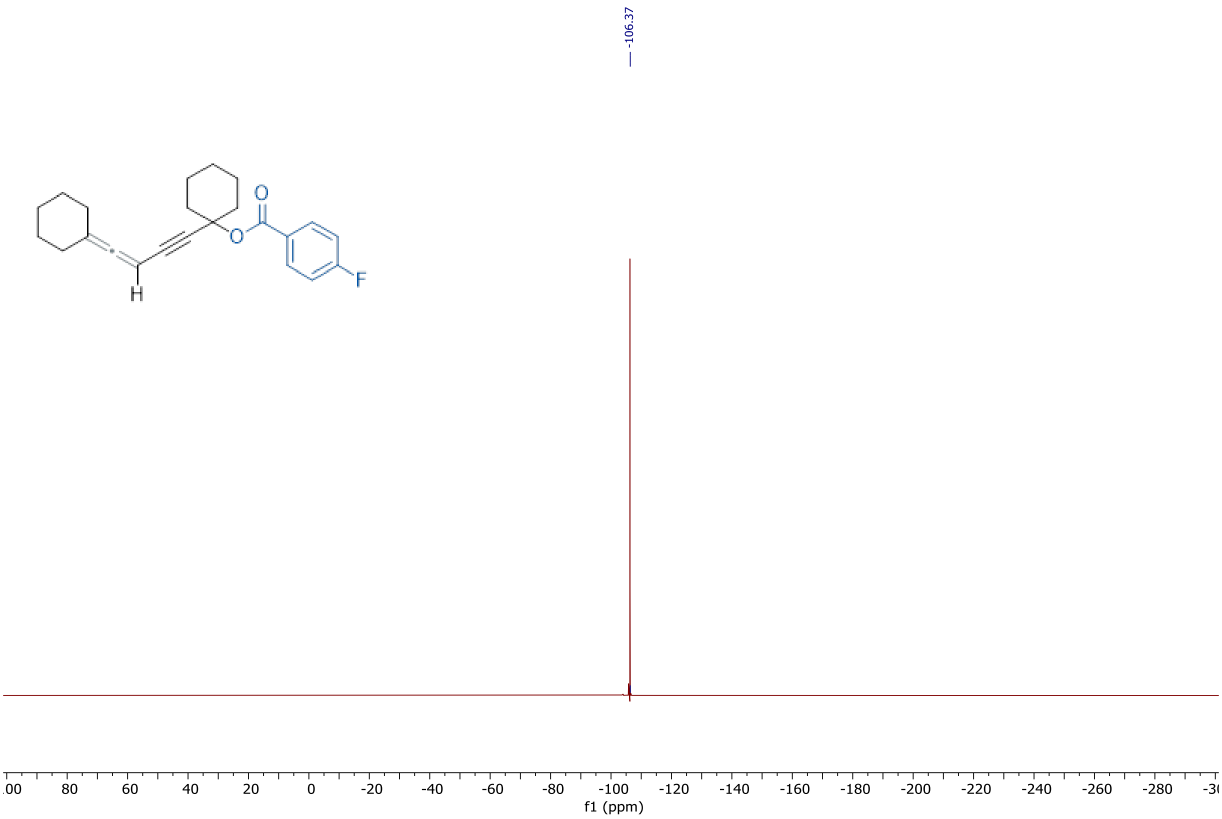

^{19}**F NMR** of compound **2v** (565 MHz, $CDCl_3$)

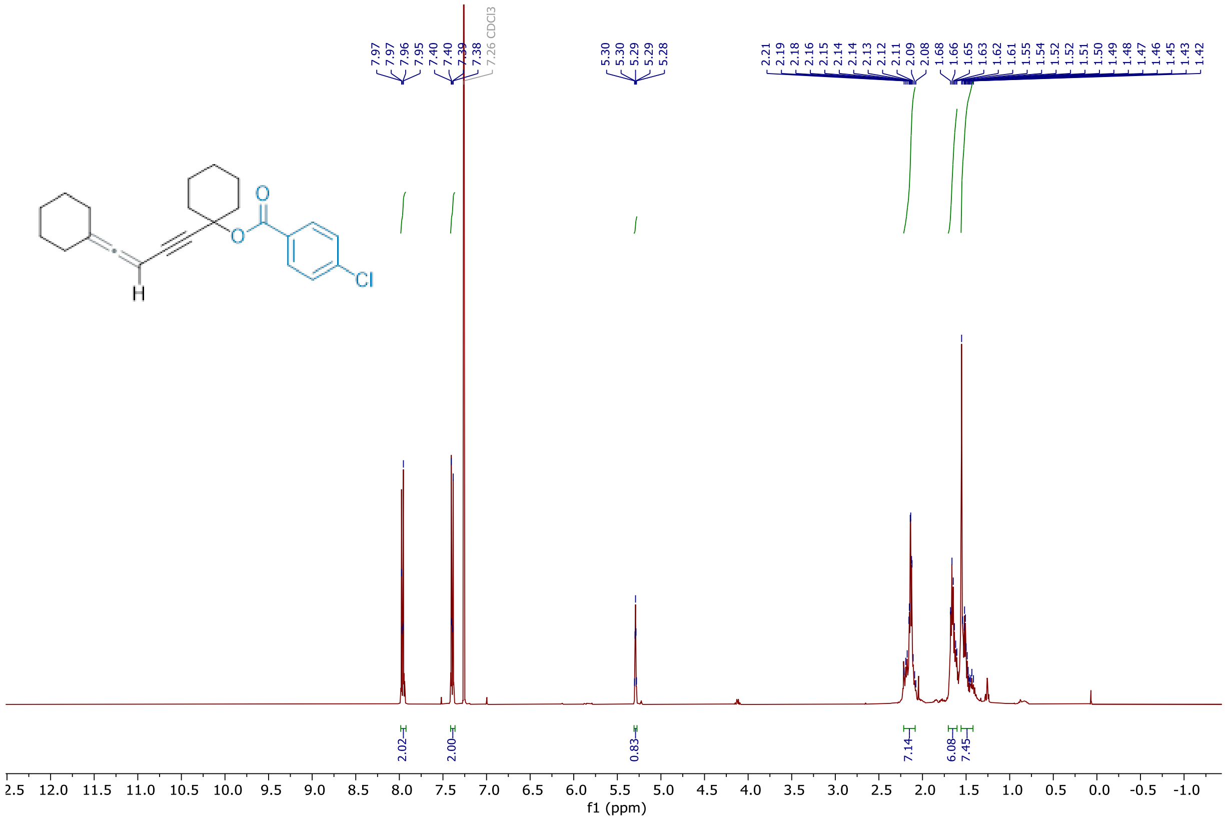

¹H NMR of compound **2w** (400 MHz, $CDCl_3$)

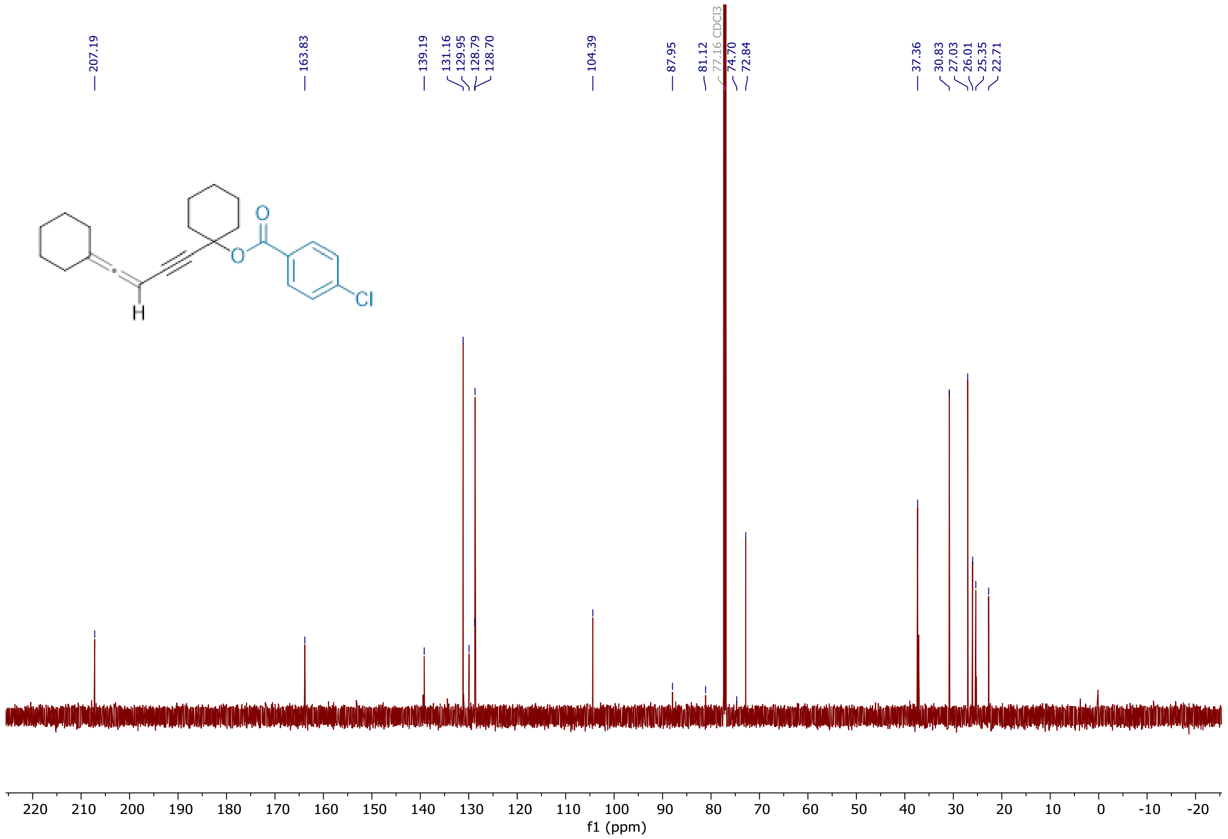

¹³C NMR of compound **2w** (151 MHz, $CDCl_3$)

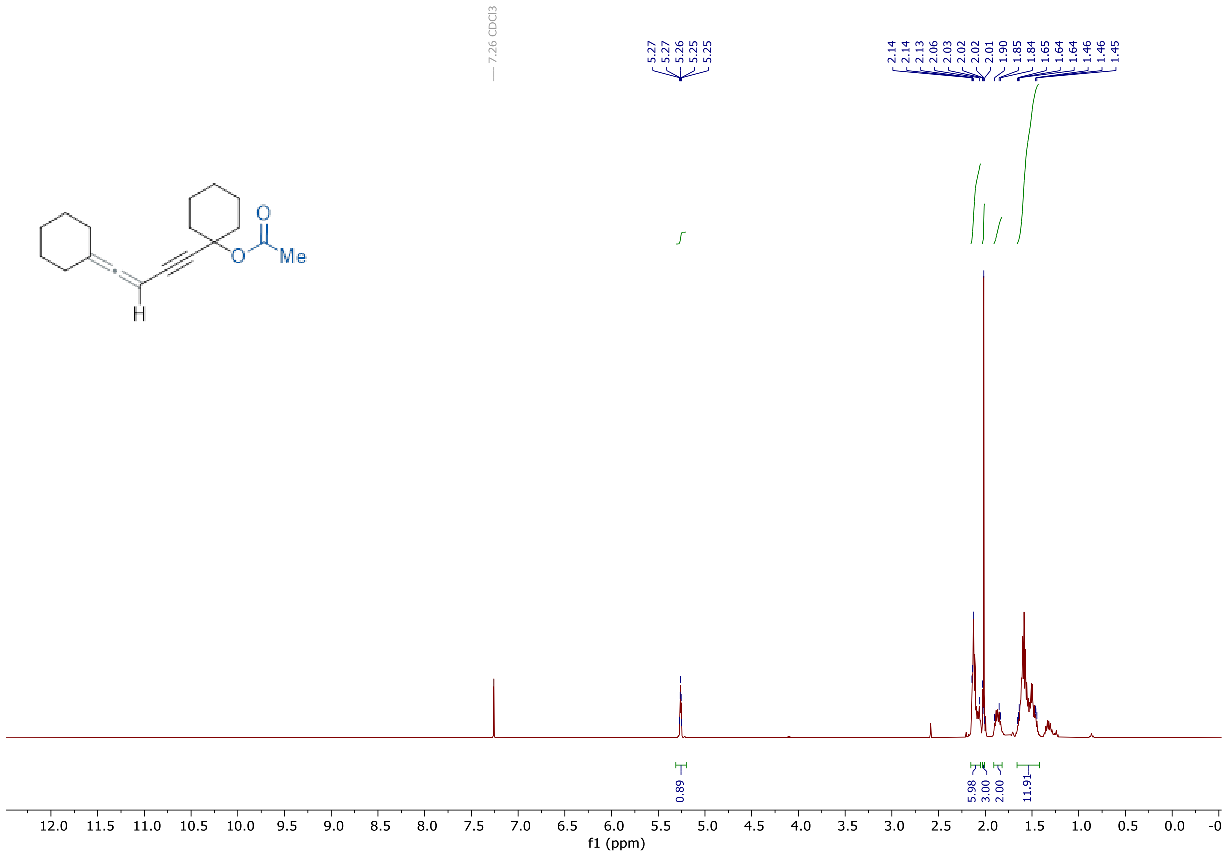

^{1}**H NMR** of compound **2x** (400 MHz, $CDCl_3$)

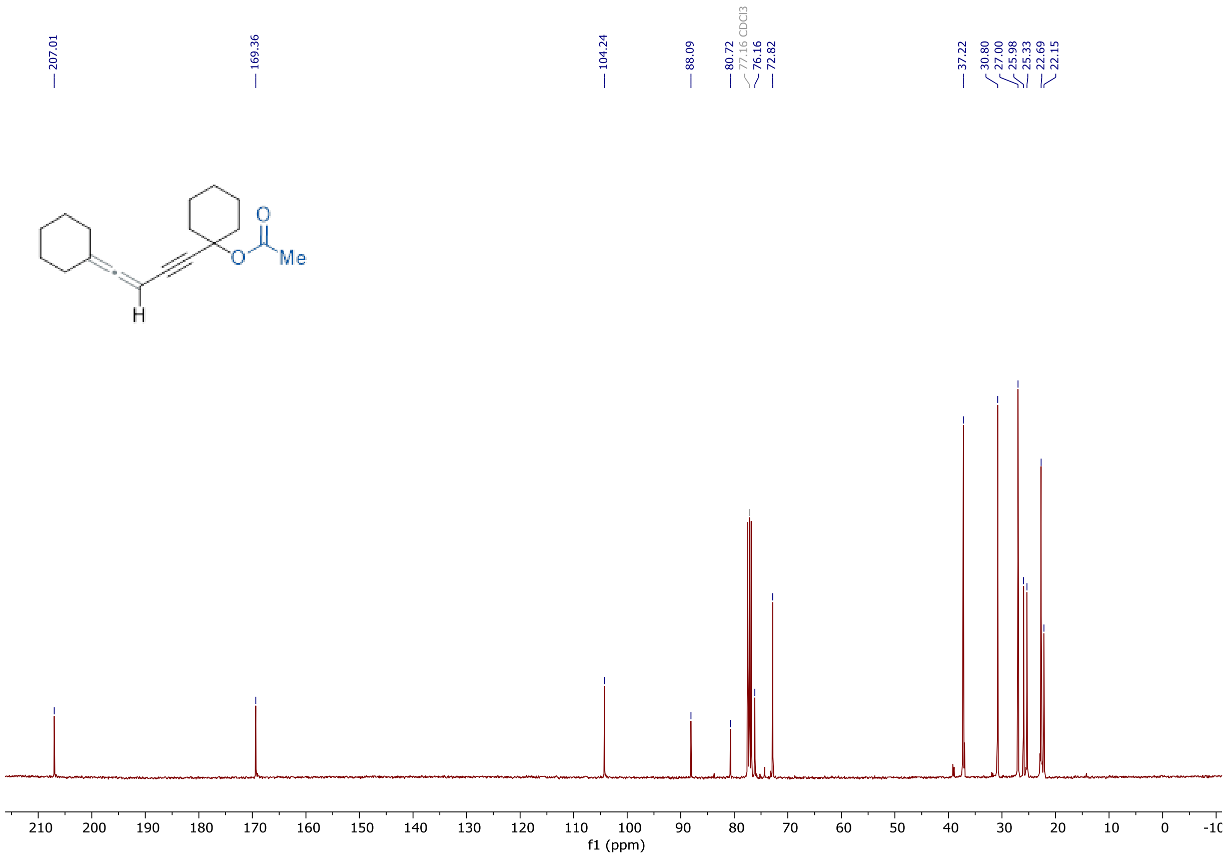

^{13}**C NMR** of compound **2x** (101 MHz, $CDCl_3$)

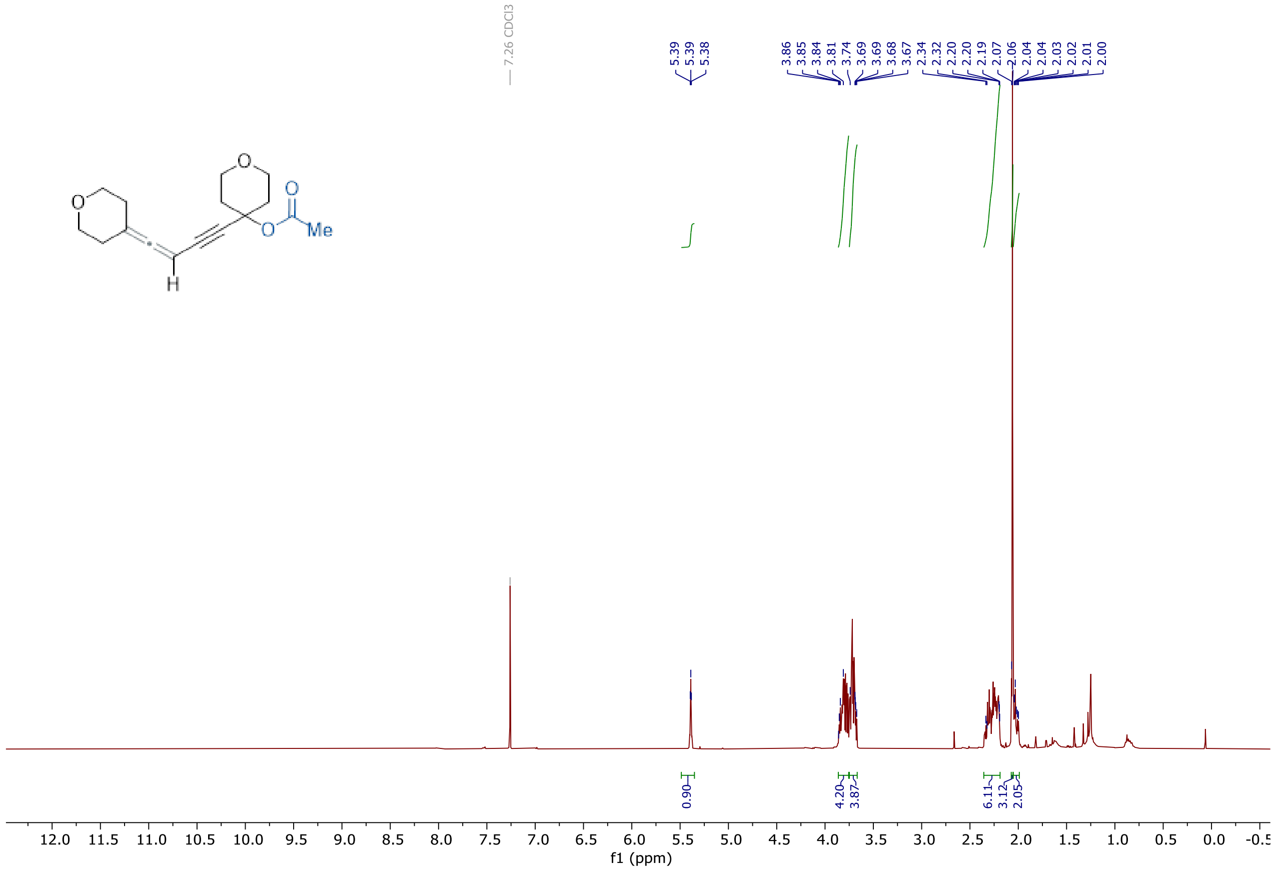

[1]**H NMR** of compound **2y** (400 MHz, CDCl₃)

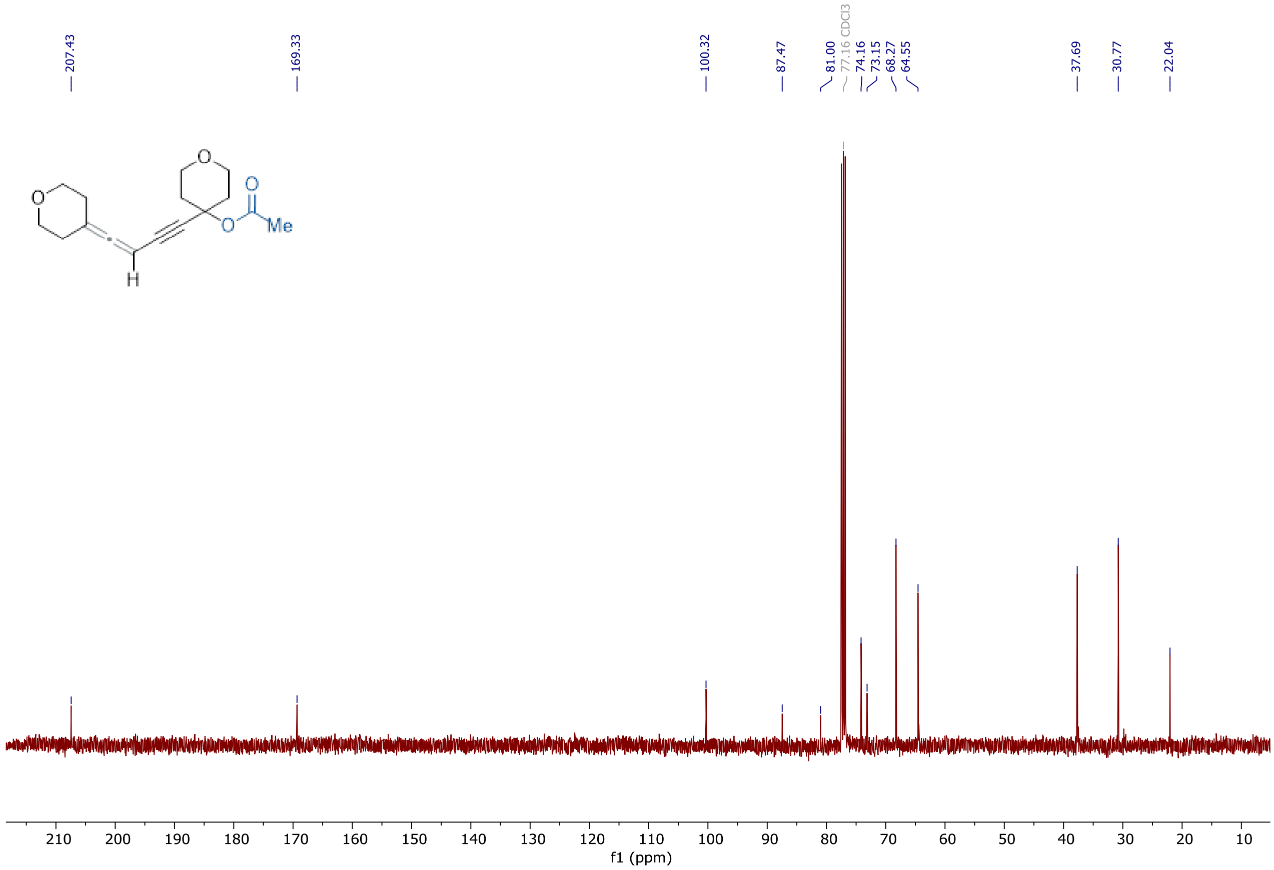

[13]**C NMR** of compound **2y** (101 MHz, $CDCl_3$)

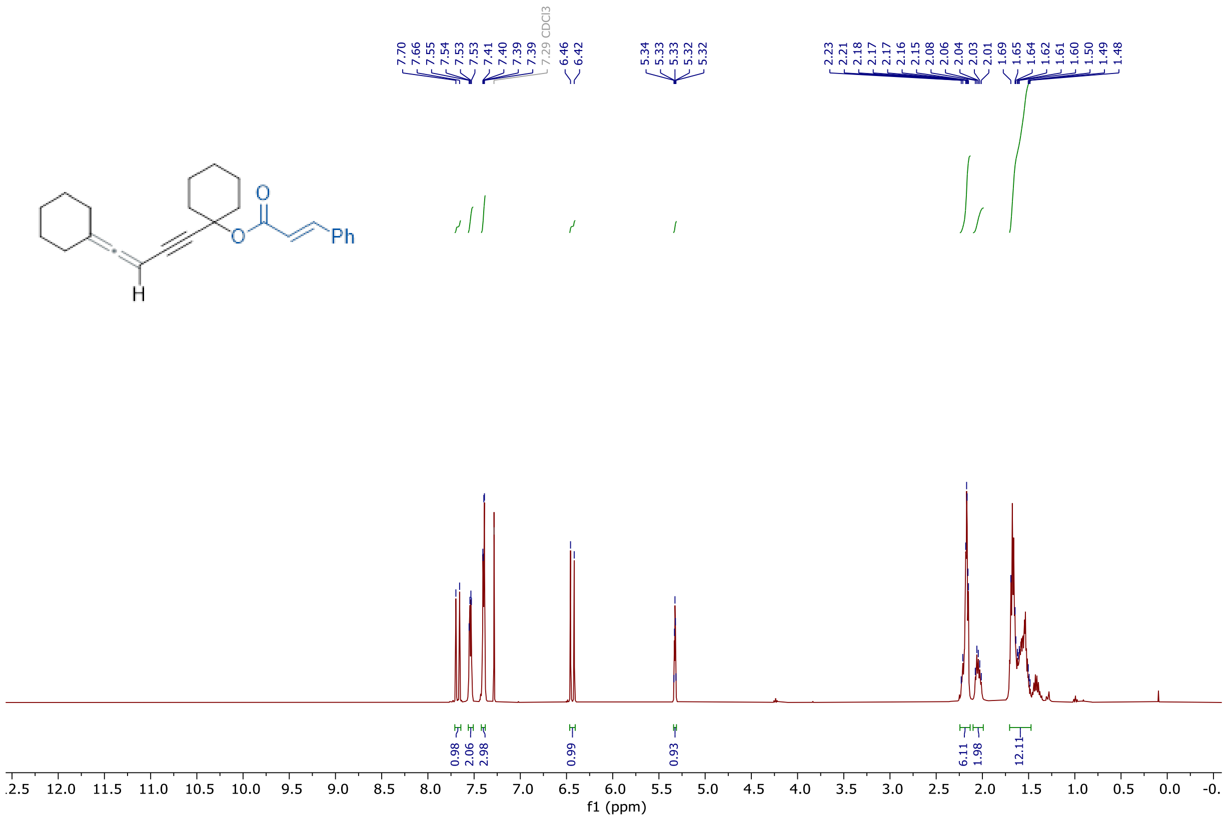

^{1}H NMR of compound **2z** (400 MHz, $CDCl_3$)

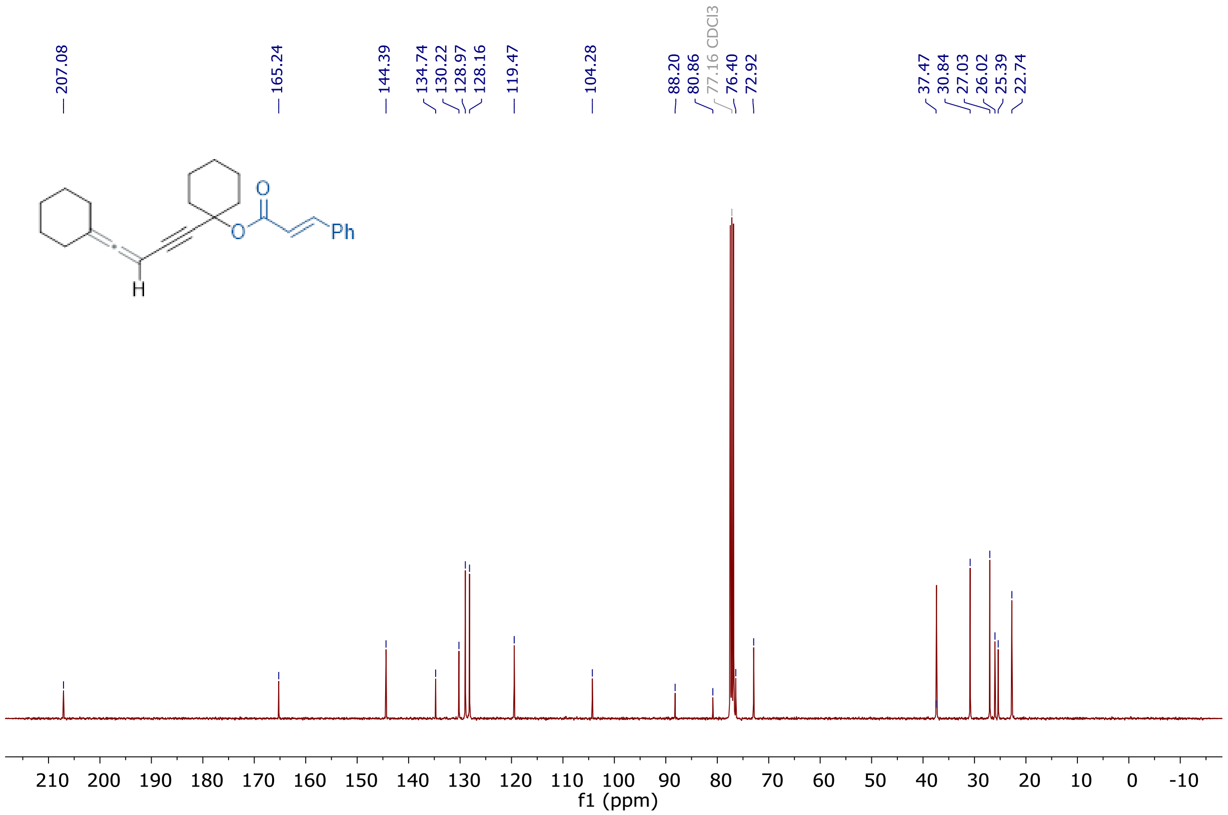

^{13}C NMR of compound **2z** (101 MHz, $CDCl_3$)

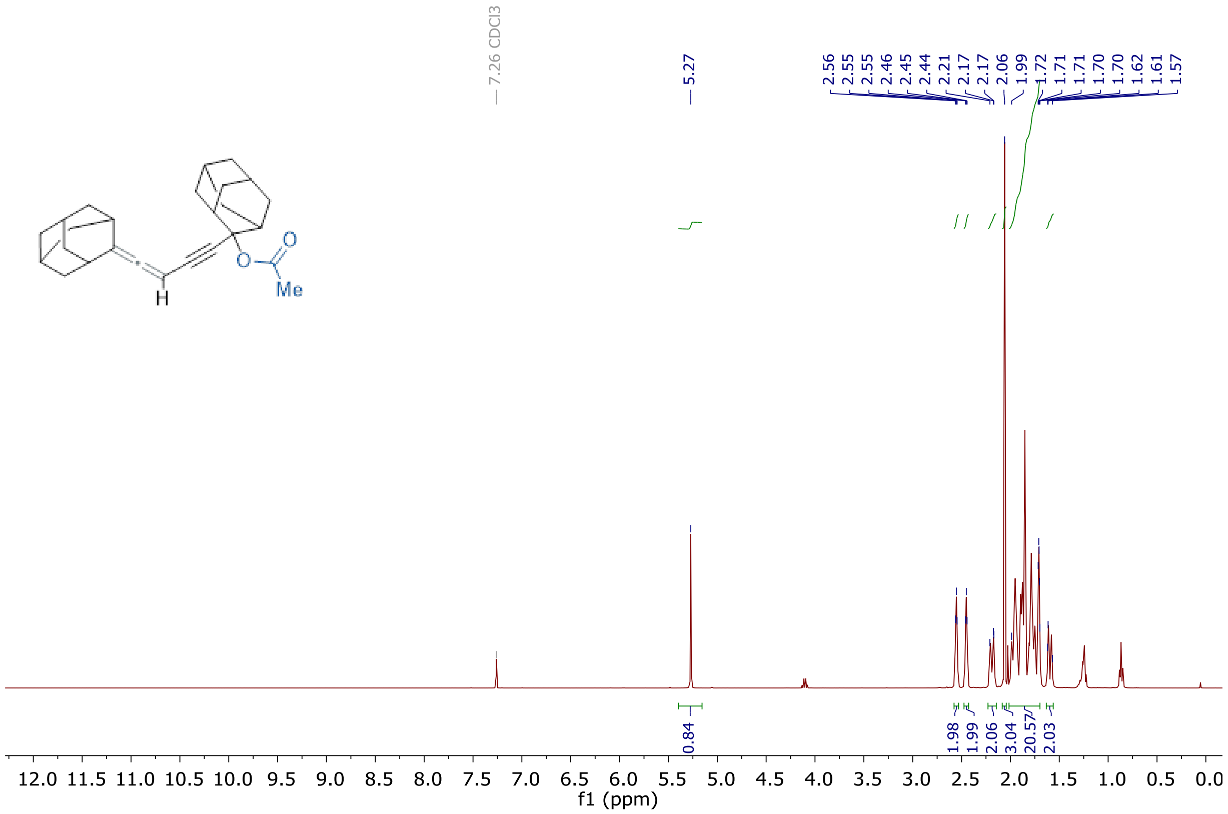

^{1}H NMR of compound **2aa** (400 MHz, $CDCl_3$)

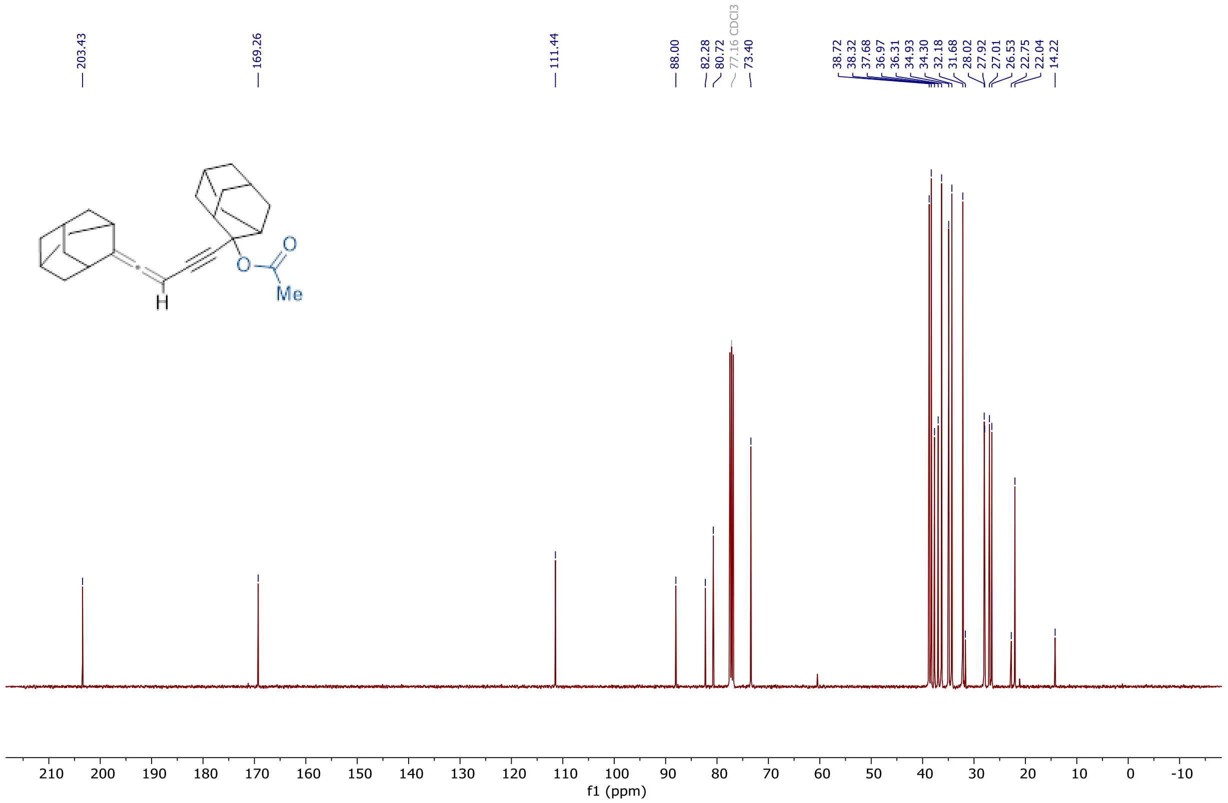

^{13}C NMR of compound **2aa** (101 MHz, $CDCl_3$)

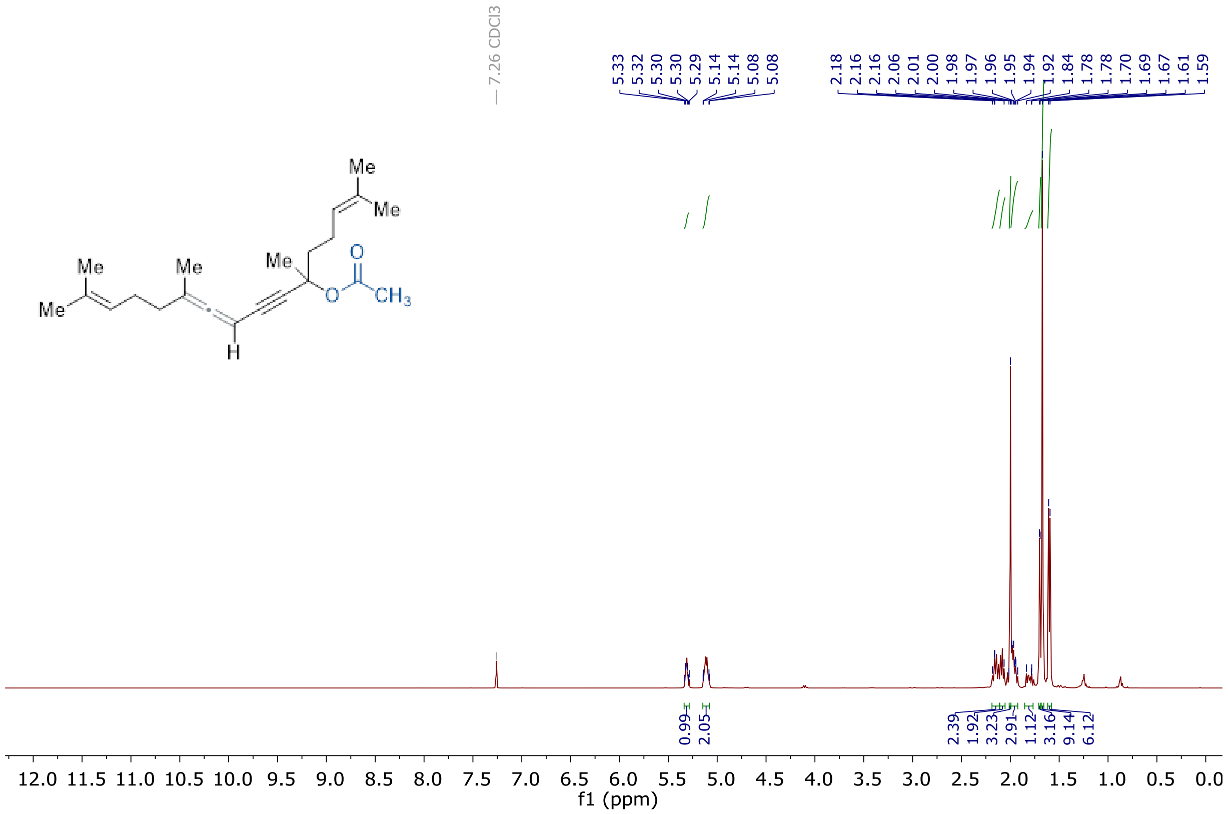

^{1}H NMR of compound **2ab** (400 MHz, $CDCl_3$)

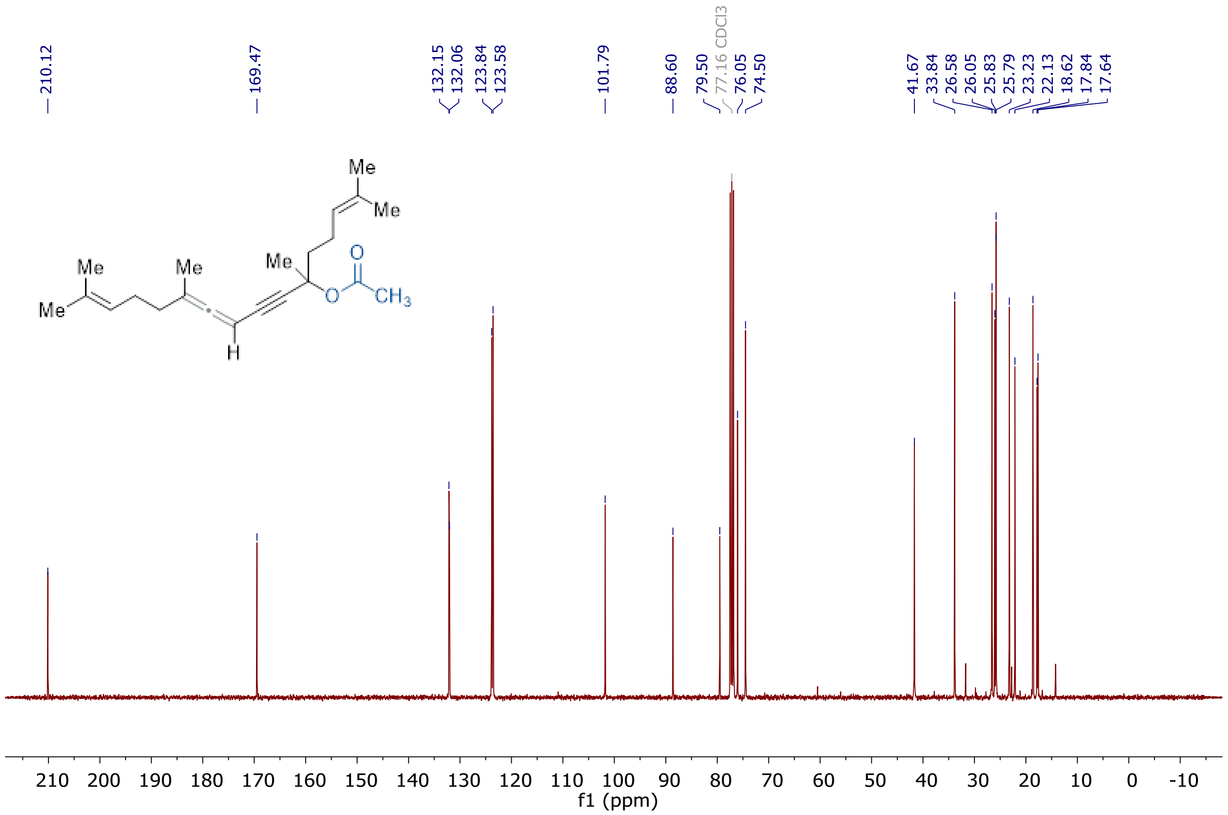

^{13}C NMR of compound **2ab** (101 MHz, $CDCl_3$)

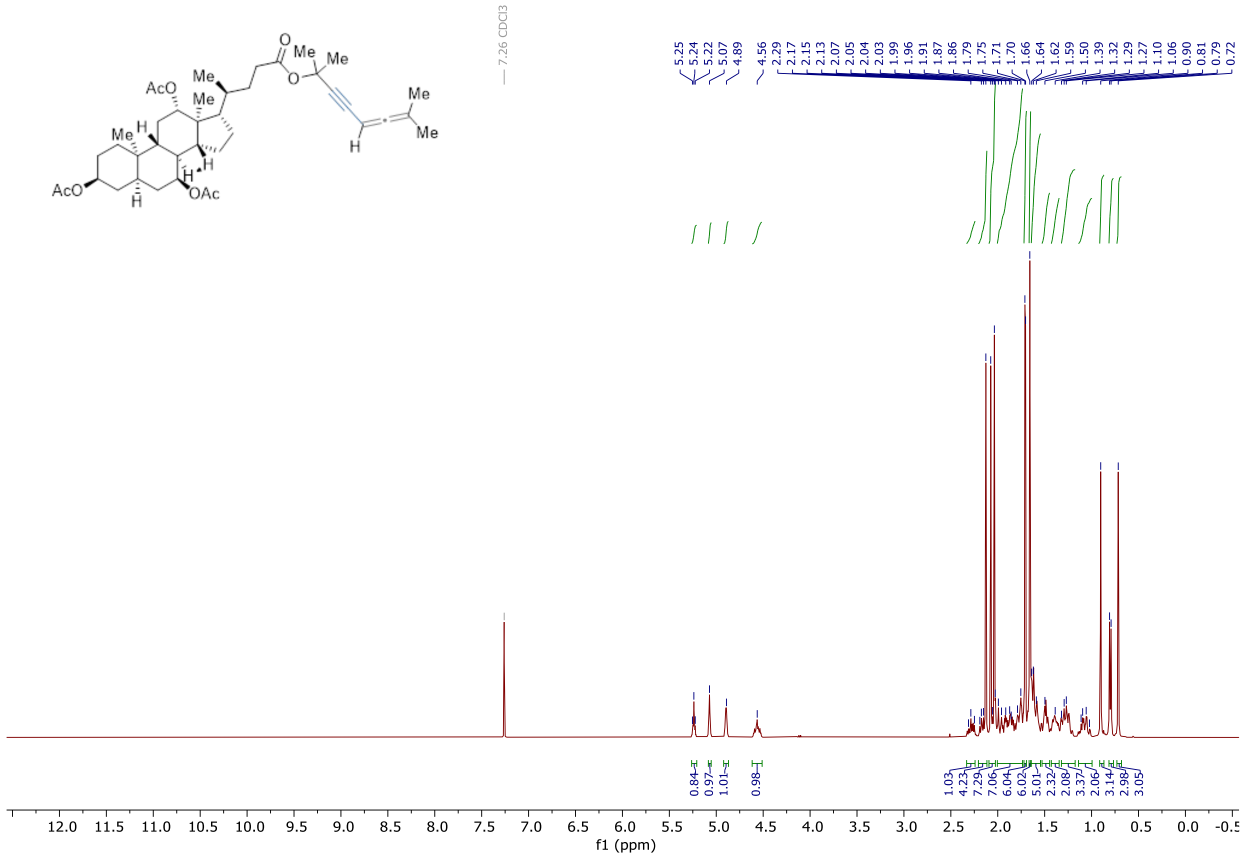

¹H NMR of compound **2ac** (400 MHz, $CDCl_3$)

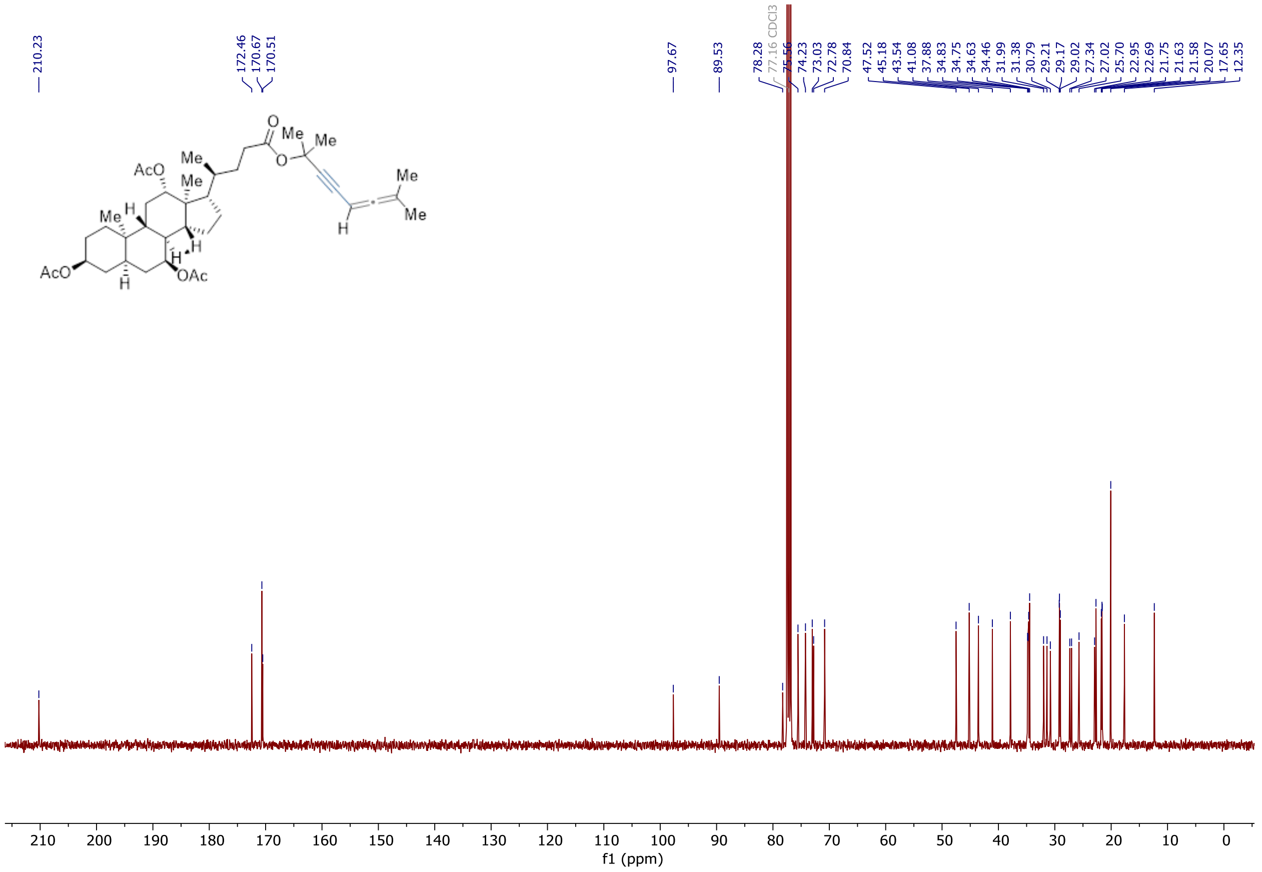

¹³C NMR of compound **2ac**(101 MHz, $CDCl_3$)

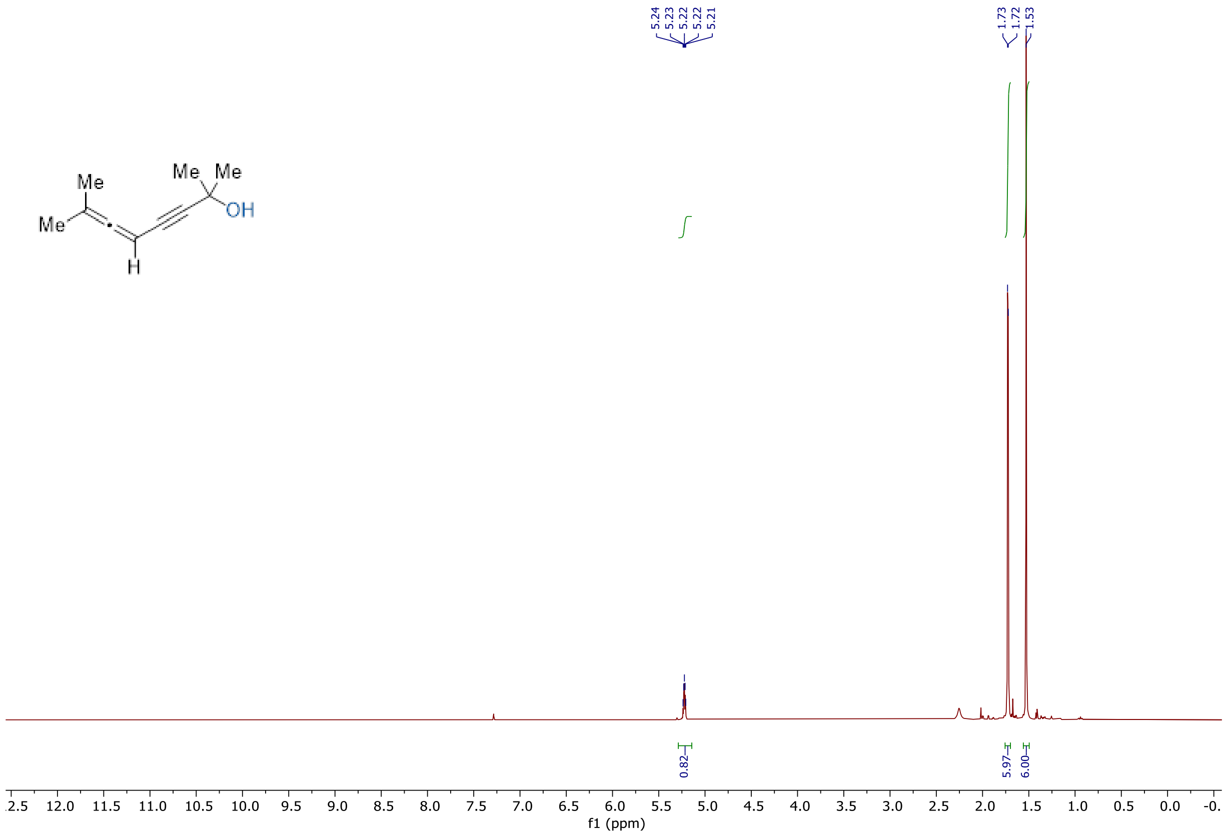

^{1}**H NMR** of compound **3** (400 MHz, $CDCl_3$)

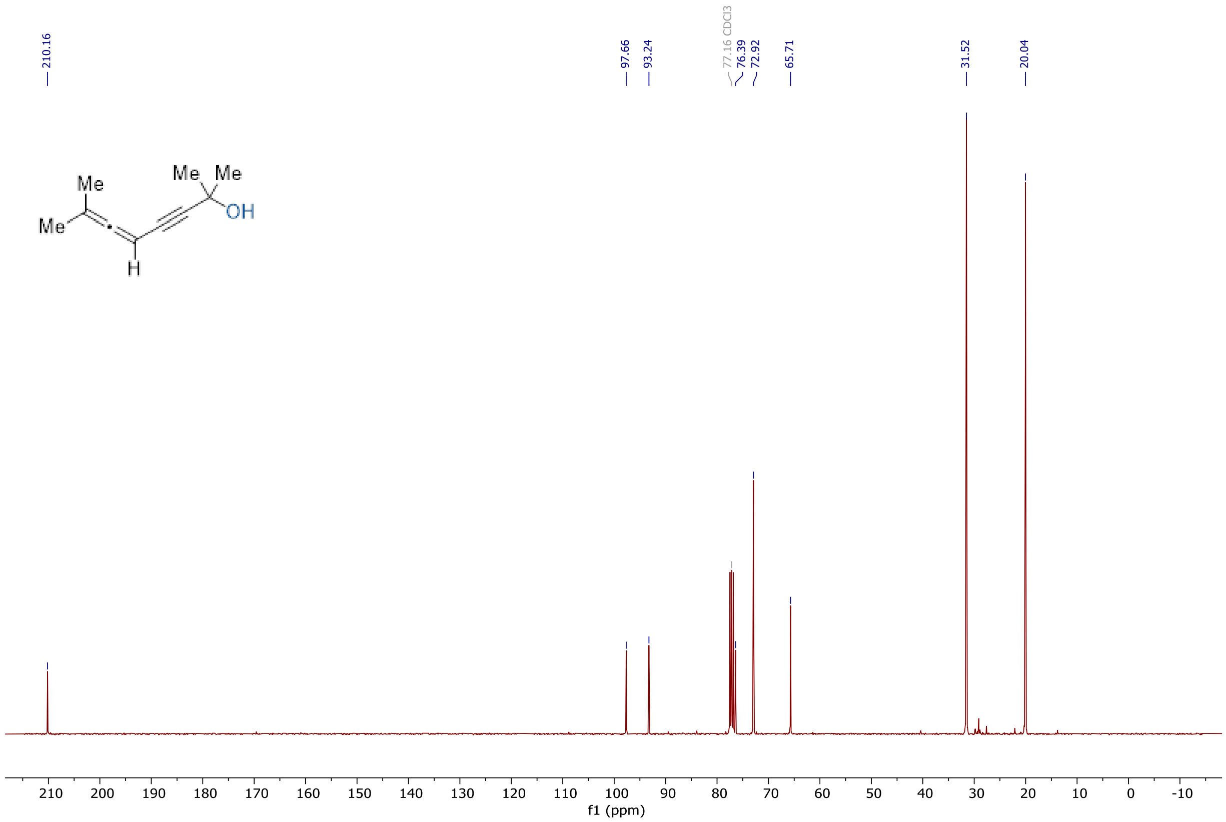

^{13}**C NMR** of compound **3** (101 MHz, $CDCl_3$)

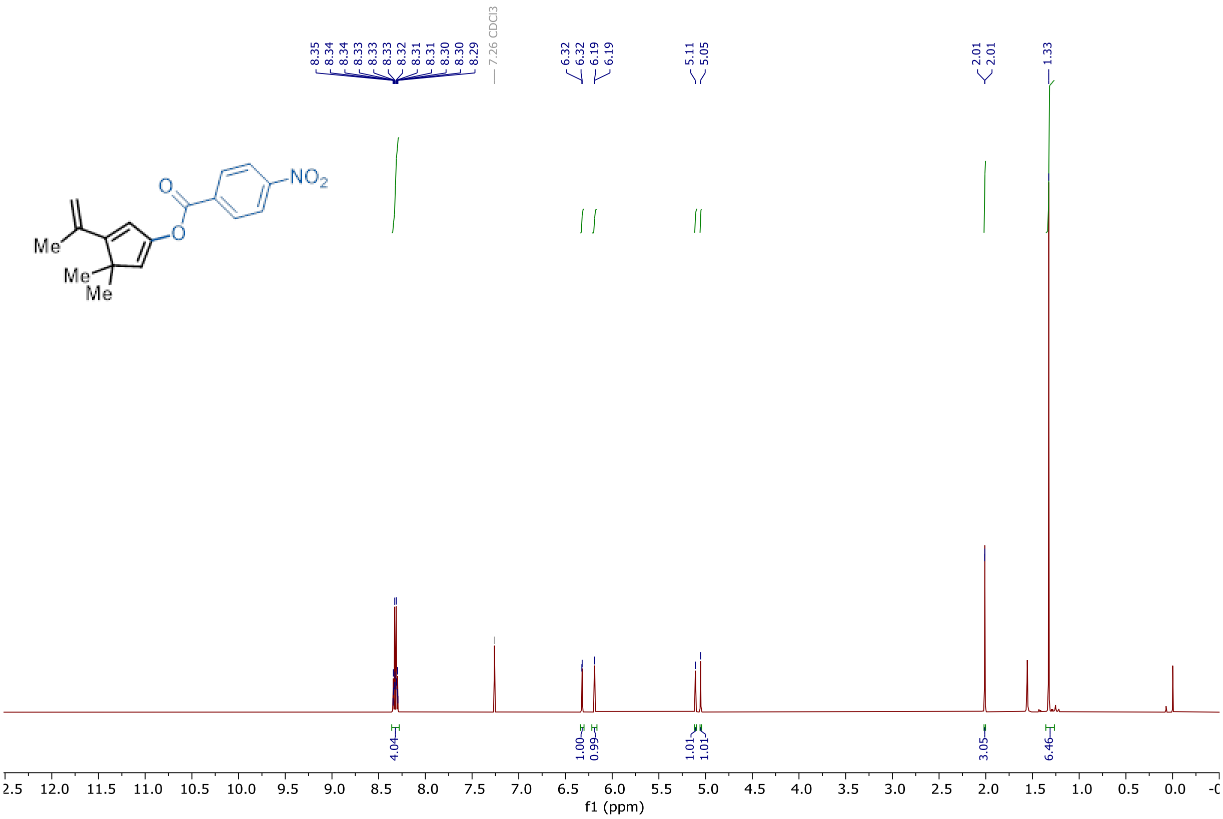

¹H NMR of compound **4** (600 MHz, $CDCl_3$)

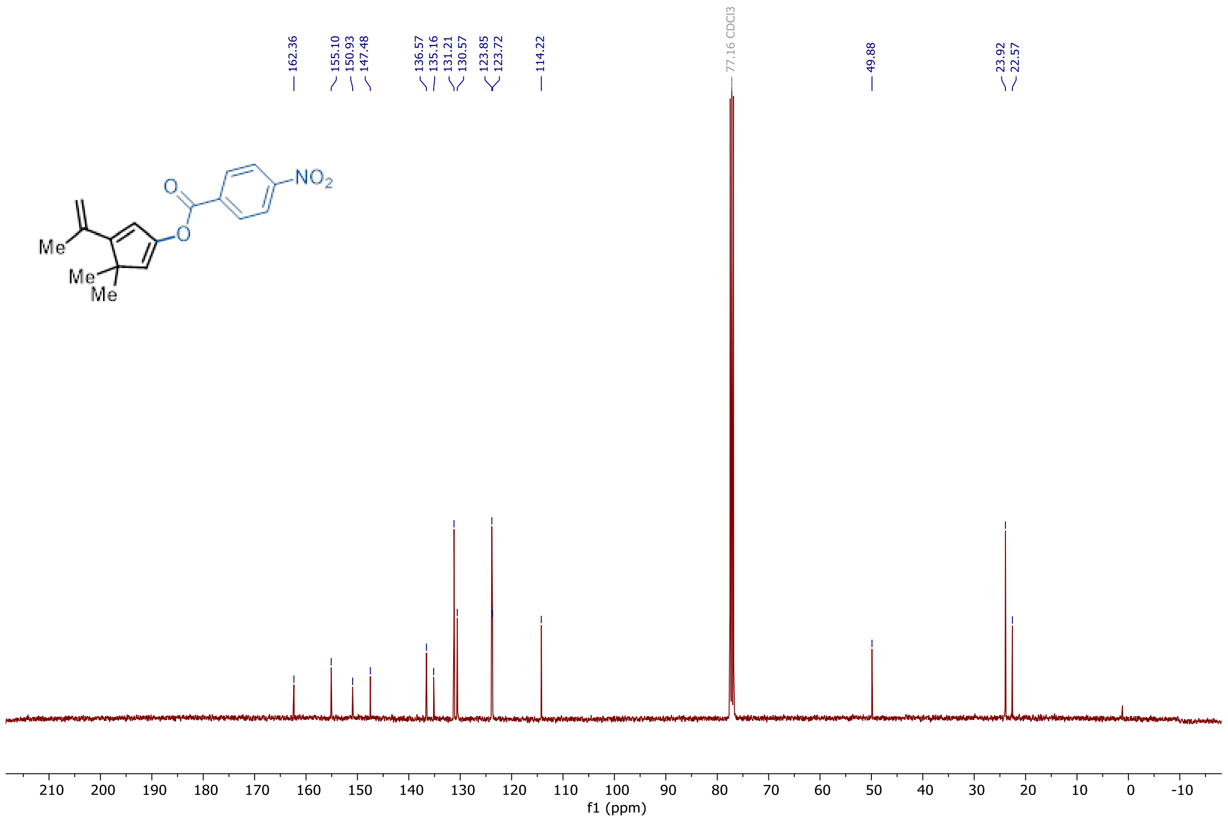

¹³C NMR of compound **4** (101 MHz, $CDCl_3$)

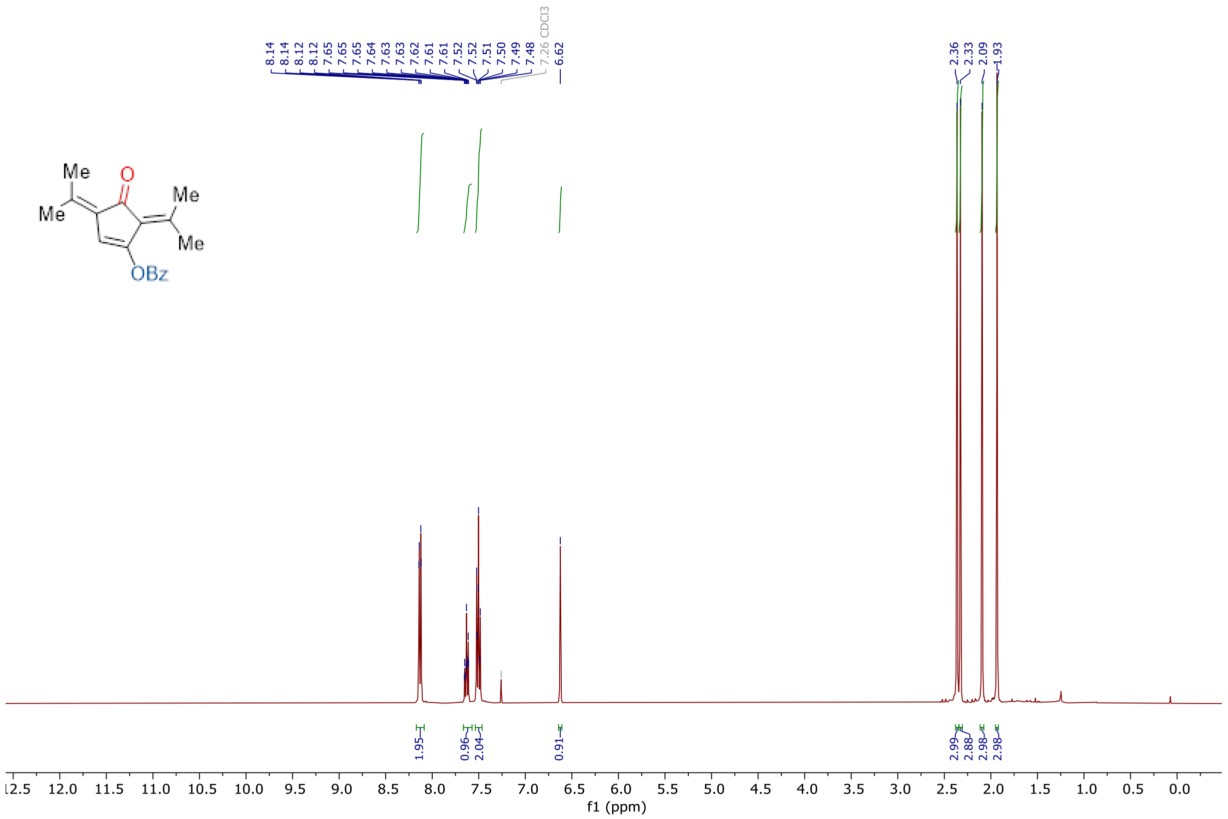

^{1}H NMR of compound **5** (400 MHz, $CDCl_3$)

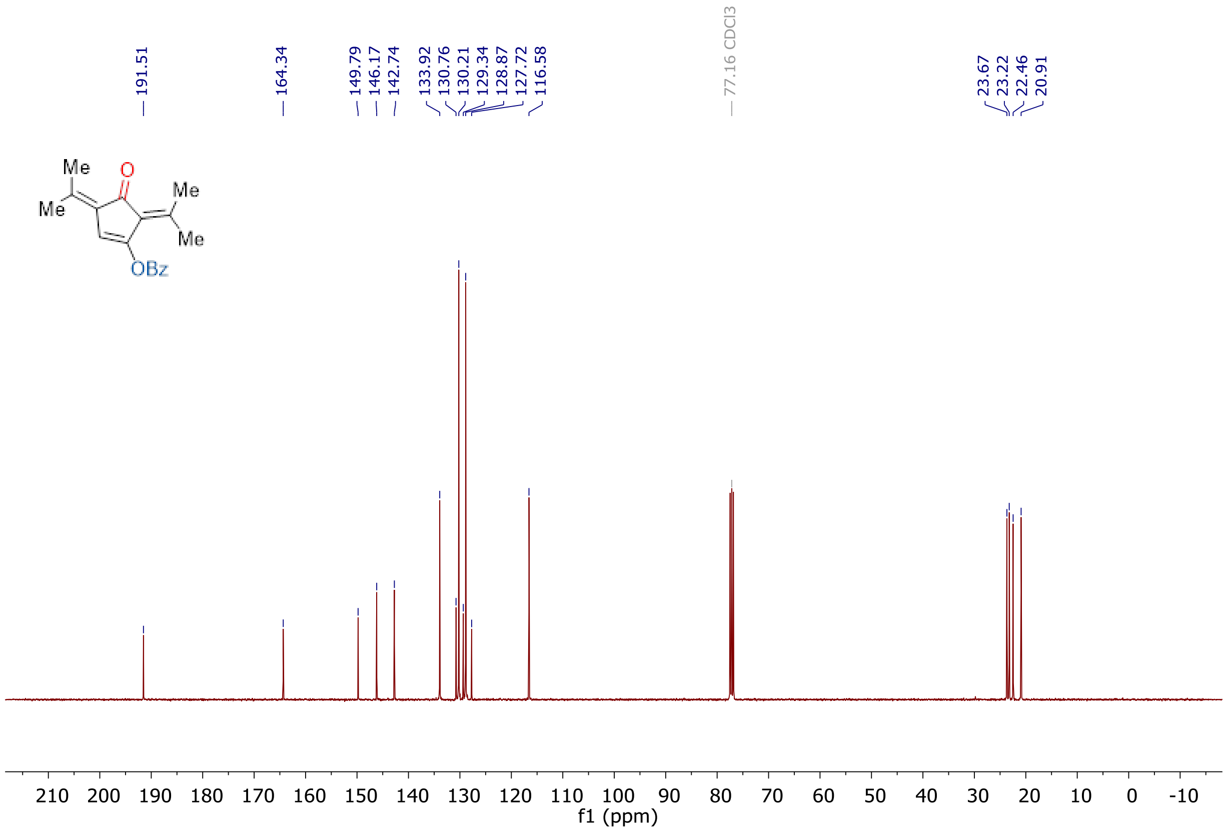

^{13}C NMR of compound **5** (101 MHz, $CDCl_3$)
